# De Novo Synthesis of α‐Ketoamides via Pd/TBD Synergistic Catalysis

**DOI:** 10.1002/advs.202404266

**Published:** 2024-07-10

**Authors:** Jia‐He Chen, Li‐Ren Zhang, Zhang‐Yang Wang, Lu‐Jie Liu, Li‐Ping Tu, Yun Zhang, Yong‐Zheng Chen, Wen‐Yong Han

**Affiliations:** ^1^ Key Laboratory of Biocatalysis & Chiral Drug Synthesis of Guizhou Province Generic Drug Research Center of Guizhou Province Green Pharmaceuticals Engineering Research Center of Guizhou Province School of Pharmacy, Zunyi Medical University No. 6 West Xuefu Rd. Zunyi 563006 China; ^2^ Key Laboratory of Basic Pharmacology of Ministry of Education and Joint International Research Laboratory of Ethnomedicine of Ministry of Education Zunyi Medical University No. 6 West Xuefu Rd. Zunyi 563006 China

**Keywords:** α‐ketoamides, double isocyanide insertion, palladium catalyst, synergistic catalysis, TBD

## Abstract

Precisely controlling the product selectivity of a reaction is an important objective in organic synthesis. α‐Ketoamides are vital intermediates in chemical transformations and privileged motifs in numerous drugs, natural products, and biologically active molecules. The selective synthesis of α‐ketoamides from feedstock chemicals in a safe and operationally simple manner under mild conditions is a long‐standing catalysis challenge. Herein, an unprecedented TBD‐switched Pd‐catalyzed double isocyanide insertion reaction for assembling ketoamides in aqueous DMSO from (hetero)aryl halides and pseudohalides under mild conditions is reported. The effectiveness and utility of this protocol are demonstrated by its diverse substrate scope (93 examples), the ability to late‐stage modify pharmaceuticals, scalability to large‐scale synthesis, and the synthesis of pharmaceutically active molecules. Mechanistic studies indicate that TBD is a key ligand that modulates the Pd‐catalyzed double isocyanide insertion process, thereby selectively providing the desired α‐ketoamides in a unique manner. In addition, the imidoylpalladium(II) complex and α‐ketoimine amide are successfully isolated and determined by X‐ray analysis, confirming that they are probable intermediates in the catalytic pathway.

## Graphical abstract

By utilizing synergistic palladium and 1,5,7‐triazabicyclo[4.4.0]dec‐5‐ene (TBD) catalysis, a challenge toward the construction of α‐ketoamides by palladium‐catalyzed double isocyanide insertion reaction in aqueous systems can be resolved smoothly. Mechanistic investigations revealed that TBD is a key ligand to control the product selectivity, and that imidoylpalladium(II) complex and α‐ketoimine amide are probable reaction intermediates.

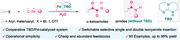

## 1 Introduction

**Figure 1:**
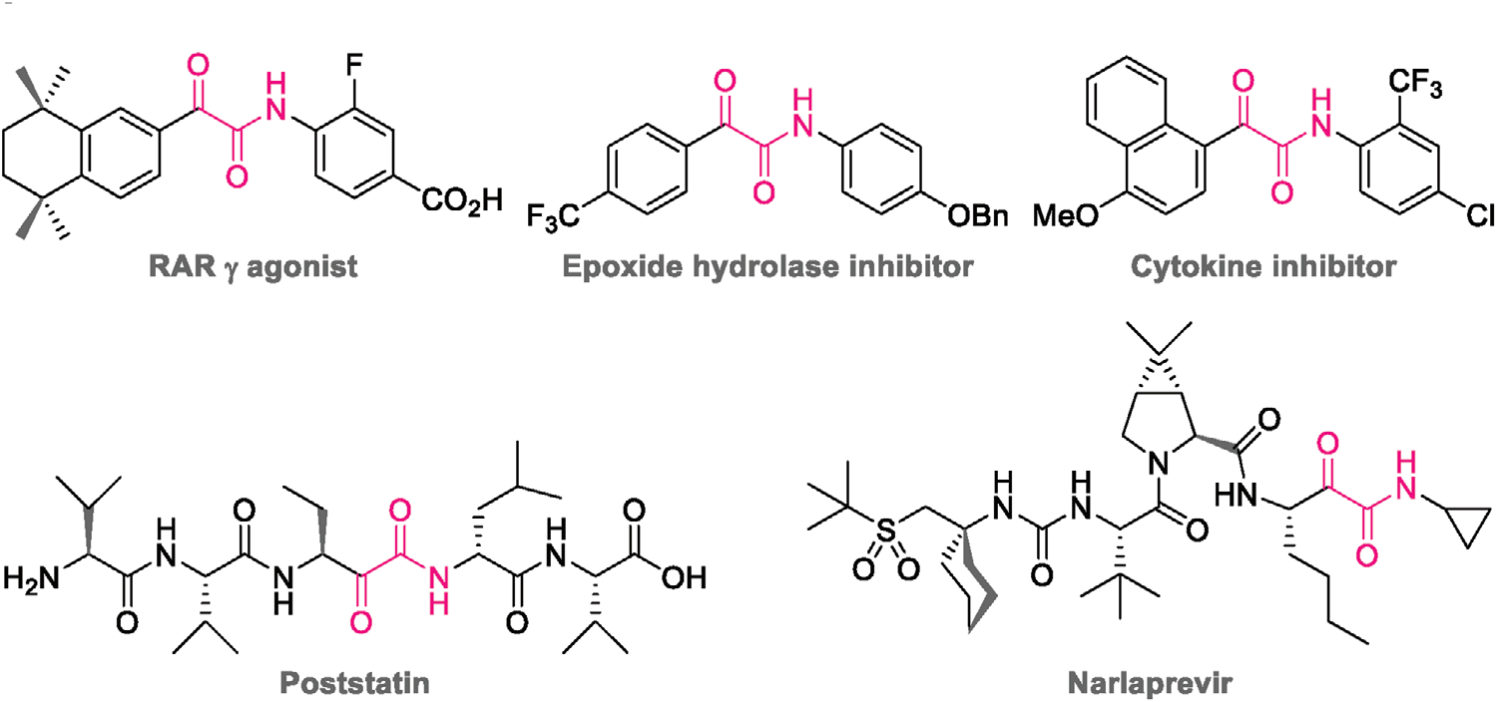
Selective examples containing α‐ketoamide moiety.

**Scheme 1:**
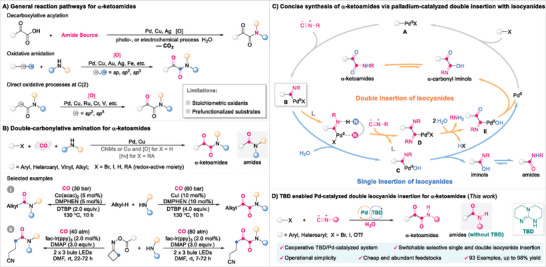
Approaches to α‐ketoamides.

α‐Ketoamides and their derivatives are important organic moieties that are prevalent in many pharmaceuticals,^[^ ^1^ ^]^ biologically relevant molecules,^[^ ^2^ ^]^ and natural products (**Figure** **1**).^[^ ^3^ ^]^ They also serve as versatile building blocks for a variety of functional group transformations owing to their structural specificities and multiple potential reaction sites.^[^ ^4^ ^]^ In this context, some general approaches, including decarboxylative acylation,^[^ ^5^ ^]^ oxidative amidation,^[^ ^6^ ^]^ direct oxidation,^[^ ^7^ ^]^ and others,^[^ ^8^ ^]^ have been rapidly developed for the synthesis of α‐ketoamides (**Scheme** **1**A).

Most α‐ketoamide synthesis methods focus on prefunctionalized substrates (e.g., 2‐hydroxyamides, α‐arylacetamides, aryl acetaldehydes, aryl methyl ketones, terminal alkynes, α‐carbonyl aldehydes, α‐ketoacids, and terminal alkenes); consequently, the development of novel approaches for the synthesis of α‐ketoamides from simple starting materials has inspired longstanding interests. Noteworthily, palladium‐catalyzed double‐carbonylative amination of a halogenated hydrocarbon in the presence of an amine under carbon monoxide (CO), which was pioneered by Yamamoto et al. in 1982,^[^ ^9^ ^]^ represents a direct and efficient strategy for the synthesis of α‐ketoamides (Scheme 1B) The scope of this elegant amino dicarbonylation chemistry was further explored by Yamamoto et al.,^[^ ^10^ ^]^ Kollár et al.,,^[^ ^11^ ^]^ Ryu et al.,,^[^ ^12^ ^]^ Jensen et al.,^[^ ^13^ ^]^ Buchwald et al.,^[^ ^13^ ^]^ Skrydstrup et al.,^[^ ^14^ ^]^ Wu et al.,^[^ ^15^ ^]^ Lei et al.,^[^ ^16^ ^]^ Zhu et al.,^[^ ^17^ ^]^ Luo et al.,^[^ ^17^ ^]^ Xiao et al.,^[^ ^18^ ^]^ Liao et al.,^[^ ^19^ ^]^ Chen et al.,^[^ ^18^, ^20^ ^]^ and others.^[^ ^21^ ^]^ In 2018, Lei and co‐workers first reported the oxidative double radical carbonylations of alkanes promoted by carbon nanofibrous microspheres (CNMs) under 60 bar of CO using stoichiometric di‐*tert*‐butyl peroxide (DTBP) as the oxidant to afford a series of α‐ketoamides in yields of 17–77%.^[^ ^16a^ ^]^ To improve this relatively low conversion efficiency, in 2022, the same group further developed tunable oxidative mono‐ and double‐carbonylation chemistry for alkanes with CO using Co and Cu catalysts, respectively (Scheme 1B‐i).^[^ ^16b^ ^]^ In the same year, Wu developed highly selective copper‐catalyzed double‐carbonylative amination chemistry involving alkyl bromides, and mono‐ and double‐carbonylation chemistry involving alkyl iodides with amines that were controlled precisely under different conditions.^[^ ^15c^ ^]^ Recently, Chen et al. illustrated a philicity‐regulation strategy that uses photocatalytic processes for the switchable single‐ and double‐carbonylation reactions of strained‐ring oxime esters at different CO pressures (Scheme 1B‐ii).^[^ ^18^ ^]^ Shortly thereafter, Liao et al. further expanded this carbonylation chemistry to include cyclic diaryliodonium salts and disclosed high‐valent Pd(II/IV)‐catalyzed asymmetric single‐ and double carbonylation reactions using a chiral sulfoxide phosphine ligand under CO to afford axially chiral biaryl compounds bearing amides or ketoamides in up to 93% yield and with up to 99% enantioselectivity.^[^ ^19^ ^]^ Quite recently, Chen's group further reported a visible‐light‐driven radical‐relayed multicomponent radical double aminocarbonylation reaction involving alkenes under 80 atm of CO to afford valuable γ‐trifluoromethyl α‐ketoamides.^[^ ^20^ ^]^ Despite the aforementioned state‐of‐the‐art approaches, surprisingly, except for rare examples that rely on 9‐methylfluorenecarbonyl chloride as a CO surrogate,^[^ ^14b^ ^]^ a safe and simple protocol for the preparation of α‐ketoamides under CO‐gas‐free conditions has, to the best of our knowledge, not yet been developed.

Isocyanides, which form a class of important reagents that are extensively utilized in multicomponent and imidoylative reactions, are easily synthesized as liquids or solids and can be used in stoichiometric quantities.^[^ ^22^ ^]^ In 2011, Jiang et al. reported palladium‐catalyzed amidation chemistry involving aryl halides and isocyanides that provides access to amides.^[^ ^23^ ^]^ Mechanistically, this reaction involves the successive oxidative addition of the aryl halide to the Pd(0) species, the single migratory insertion of the isocyanide, nucleophilic substitution, reductive elimination, and amide‐iminol tautomerism (Scheme 1C, catalytic cycle in gray and blue). While palladium‐catalyzed double isocyanide insertion reactions are well‐precedented for the syntheses of α‐iminoimidates,^[^ ^24^ ^]^ ketimine‐amidine,^[^ ^25^ ^]^ 1,2‐diketones,^[^ ^26^ ^]^ α‐ketoester,^[^ ^27^ ^]^ α‐diimines,^[^ ^28^ ^]^ α‐iminonitriles,^[^ ^29^ ^]^ 3‐amino pyrroles,^[^ ^30^ ^]^ 3‐iminoindol‐2‐amines,^[^ ^31^ ^]^ fused tetracyclic heterocycles,^[^ ^32^ ^]^ and aza‐saddle‐type frameworks,^[^ ^33^ ^]^ among others, expanding this strategy to the construction of α‐ketoamides with H_2_O as the nucleophile remains a formidable challenge, supposedly because imidoylpalladium(II) B favors nucleophilic substitution with H_2_O to give intermediate C rather than a second isocyanide insertion to form intermediate D. As part of our ongoing interests in palladium‐catalyzed domino reactions,^[^ ^34^ ^]^ as well as our recent progress in palladium‐enabled rearrangement processes involving isocyanides,^[^ ^35^ ^]^ we envisaged that a bifunctional (NH, N)‐ligand may stabilize intermediate B through coordination of the nitrogen atom to the Pd(II) center, which would lower the activation barrier for a second isocyanide insertion to give intermediate D. Subsequent partial hydrolysis of the imine moiety and nucleophilic substitution of the α‐imine palladium species with H_2_O would provide β‐carbonyl α‐imino palladium species E, which then undergoes reductive elimination and amide‐iminol tautomerism to afford the α‐ketoamide, with the Pd(0) catalyst regenerated (Scheme 1C, catalytic cycle in gray and orange). To explore this possibility, various reaction parameters, including ligands, catalysts, bases, and solvents, were examined. We found that the ligand exhibits the utmost effect on the reaction pathway toward α‐ketoamide and amide selectivity. Herein, we report palladium‐catalyzed double isocyanide insertion chemistry involving (hetero)aryl halides or pseudohalides and enabled by triazabicyclo[4.4.0]dec‐5‐ene (TBD) that provides access to α‐ketoamides (Scheme 1D). The protocol is distinguished by its operational simplicity, mild conditions, as well as inexpensive and abundant feedstocks, thereby presenting a convenient platform for the construction of valuable α‐ketoamides.

## 2 Results and Discussion

### 2.1 Search for the Optimal Reaction Conditions

Based on our mechanistic assumption (Scheme 1C), we initially evaluated an array of bifunctional (NH, N)‐ligands in the palladium‐catalyzed double isocyanide insertion reaction by subjecting bromobenzene (**1**) and *tert*‐butyl isocyanide (**2**) to PdCl_2_ (10 mol%), Cs_2_CO_3_ (1.0 equiv.), and H_2_O (100 µL) in DMSO (1.0 mL) under Ar at 90 °C for 12 h (**Table** **1**, entries 1–7).^[^ ^36^ ^]^ The desired α‐ketoamide product **3** was smoothly generated in 36% yield in the presence of 1,1,3,3‐tetramethylguanidine (**L1**, TMG) (Table 1, entry 1). Disappointingly, benzimidamide (**L2**), propionimidamide (**L3**), 1,3‐diphenylguanidine (**L4**), 1,3,5‐triazine‐2,4,6‐triamine (**L5**), and 2‐methylpyrimidine‐4,6(1*H*,5*H*)‐dione (**L6**) were ineffective in this transformation (Table 1, entries 2–6). Surprisingly, 1,5,7‐triazabicyclo[4.4.0]dec‐5‐ene (**L7**, TBD) proved to be the best cocatalyst, with **3** obtained in 49% yield, along with trace amounts of amide **4** as the single isocyanide insertion product (Table 1, entry 7). Inspired by these fascinating results, other reaction parameters, including the palladium catalyst, base, and solvent, among others, were investigated in the presence of TBD (Table 1, entries 8–18). Replacing PdCl_2_ with Pd_2_(dba)_3_ resulted in a 63% yield of **3** (Table 1, entry 12), whereas Pd(OAc)_2_, Pd(TFA)_2_, Pd(OPiv)_2_, and Pd(PPh_3_)_4_ afforded the product in slightly lower yields (Table 1, entries 8–11). However, none of the inorganic bases screened led to improved conversions (Table 1, entries 13–15). A survey of solvents revealed that DMSO was always the best choice (Table 1, entry 12 vs entries 16–18); trace amounts of the product were obtained using MeCN or PhMe as the reaction medium (Table 1, entries 17 and 18). To our delight, the yield of **3** promptly increased from 63% to 81% when the reaction was performed using 2.5 equiv. of **2** and 2.0 equiv. of Cs_2_CO_3_ (Table 1, entry 19). As expected, a lower yield of **3** was obtained when less Pd_2_(dba)_3_ was used (Table 1, entry 20). Of particularly note, different product selectivity was observed when the reaction was performed under classic palladium‐catalysis conditions, with amide **4** obtained in 74% yield without any α‐ketoamide **3** observed (Table 1, entry 21).^[^ ^23^ ^]^

**Table 1:** Search for the optimal reaction conditions.

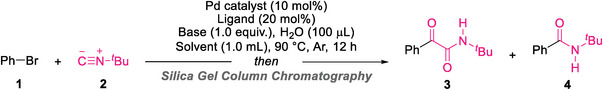						
Entry^a)^	Ligand	Pd catalyst	Base	Solvent	Yield (%)^b)^	
					3	4
1	**L1**	PdCl_2_	Cs_2_CO_3_	DMSO	36	Trace
2	**L2**	PdCl_2_	Cs_2_CO_3_	DMSO	NR	—
3	**L3**	PdCl_2_	Cs_2_CO_3_	DMSO	NR	—
4	**L4**	PdCl_2_	Cs_2_CO_3_	DMSO	NR	—
5	**L5**	PdCl_2_	Cs_2_CO_3_	DMSO	NR	—
6	**L6**	PdCl_2_	Cs_2_CO_3_	DMSO	NR	—
7	**L7**	PdCl_2_	Cs_2_CO_3_	DMSO	49	Trace
8	**L7**	Pd(OAc)_2_	Cs_2_CO_3_	DMSO	32	Trace
9	**L7**	Pd(TFA)_2_	Cs_2_CO_3_	DMSO	37	Trace
10	**L7**	Pd(OPiv)_2_	Cs_2_CO_3_	DMSO	41	Trace
11	**L7**	Pd(PPh_3_)_4_	Cs_2_CO_3_	DMSO	34	Trace
12	**L7**	Pd_2_(dba)_3_	Cs_2_CO_3_	DMSO	63	Trace
13	**L7**	Pd_2_(dba)_3_	CsF	DMSO	18	Trace
14	**L7**	Pd_2_(dba)_3_	K_2_CO_3_	DMSO	32	Trace
15	**L7**	Pd_2_(dba)_3_	*^t^* BuOK	DMSO	22	Trace
16	**L7**	Pd_2_(dba)_3_	Cs_2_CO_3_	DMF	16	Trace
17	**L7**	Pd_2_(dba)_3_	Cs_2_CO_3_	MeCN	trace	—
18	**L7**	Pd_2_(dba)_3_	Cs_2_CO_3_	PhMe	trace	—
19^c)^	**L7**	Pd_2_(dba)_3_	Cs_2_CO_3_	DMSO	81	Trace
20^c)^, ^d)^	**L7**	Pd_2_(dba)_3_	Cs_2_CO_3_	DMSO	63	Trace
21^e)^	**L8**	PdCl_2_	Cs_2_CO_3_	DMSO	0	74
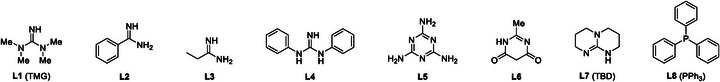						

^a)^ Unless otherwise noted, all reactions were performed with **1** (0.2 mmol, 1.0 equiv.), **2** (0.6 mmol, 3.0 equiv.), H_2_O (100 µL), Pd catalyst (10 mol%), ligand (20 mol%), base (0.2 mmol, 1.0 equiv.) in 1.0 mL of solvent under Ar atmosphere at 90 °C for 12 h, and then purified by silica gel column chromatography;

^b)^ Isolated yield based on **1**;

^c)^ The reaction was performed with 2.5 equiv. of **2** and 2.0 equiv. of Cs_2_CO_3_;

^d)^ The reaction was performed with 5 mol% of Pd_2_(dba)_3_;

^e)^ The reaction was performed with 5 mol% of PdCl_2_ and 10 mol% of PPh_3_. NR = no reaction.

### 2.2 (Hetero)aryl Bromide Scope

**Scheme 2:**
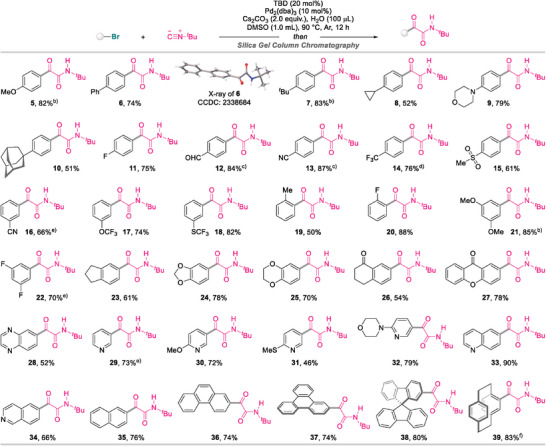
Scope of (hetero)aryl bromides^a)^. a) Reaction conditions: bromoarenes (0.2 mmol), *tert*‐butyl isocyanide (0.5 mmol), TBD (0.04 mmol), Pd_2_(dba)_3_ (0.02 mmol), Cs_2_CO_3_ (0.4 mmol), and H_2_O (100 µL) in 1.0 mL of DMSO under an Ar atmosphere at 90 °C for 12 h. Isolated yields are given. b) Performed with 20 mol% of Pd_2_(dba)_3_ and 40 mol% of TBD. c) Performed at 90 °C for 3 h. d) Performed at 90 °C for 2 h. e) Performed with 3.0 equiv. of Cs_2_CO_3_. f) Performed at 110 °C for 24 h.

Having established the optimal reaction conditions for the formation of α‐ketoamides, the generality of the current protocol with regard to the (hetero)aryl bromide was initially examined, as outlined in **Scheme** **2**. Reactions proceeded smoothly, irrespective of whether the *ortho*‐, *meta*‐, or *para*‐substituent on the bromoarene is electron‐donating or ‐withdrawing, to deliver the desired α‐ketoamides **5**–**20** in good yields (50–88%).^[^ ^37^ ^]^ In addition, this approach can be extended to disubstituted bromoarenes bearing electron‐rich or electron‐poor groups, with the corresponding products **21**–**26** obtained in good yields. Given the importance of heteroarenes in the pharmaceutical industry, the scope of the isocyanide double‐insertion event toward heteroaryl bromides was further evaluated using the established methodology. To our delight, α‐ketoamides with incorporated xanthone (**27**), quinoxaline (**28**), pyridine (**29**–**32**), quinoline (**33**), and isoquinoline (**34**) moieties were smoothly accessed in up to 90% yield. Bromoarenes featuring π‐extended systems also proved compatible and provided the corresponding α‐ketoamides **35**–**37** in good results. Moreover, this TBD‐switchable assembly platform was successfully applied to sterically hindered 2‐bromo‐9,9′‐spirobi[fluorene] and 4‐bromo[2.2]paracyclophane to furnish desired products **38** and **39** in yields of 80% and 83%, respectively.

### 2.3 (Hetero)aryl Sulfonate Scope

**Scheme 3:**
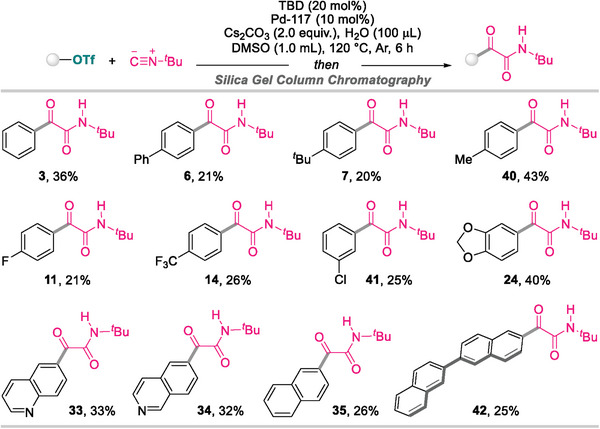
Scope of (hetero)aryl sulfonates^a)^. a) Performed on 0.2 mmol scale at 120 °C for 6 h. Isolated yields are given.

The generality of the current methodology was investigated using pseudohalides, as depicted in **Scheme** **3**.^[^ ^36^ ^]^ Notably, the double isocyanide insertion process appears to be laborious for (hetero)aryl sulfonates, which required a higher temperature (120 °C) to install the desired α‐ketoamides in low yields.

### 2.4 (Hetero)aryl Iodide Scope

**Scheme 4:**
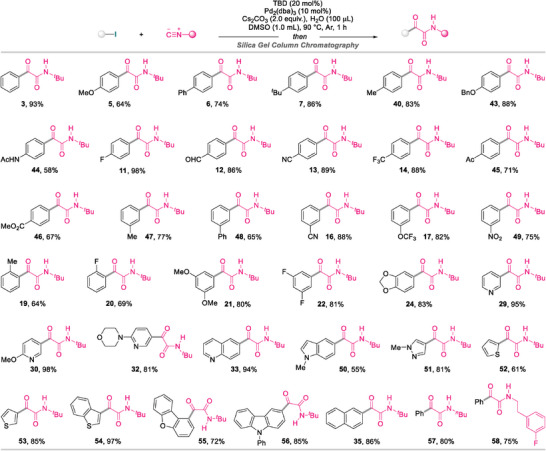
Scope of (hetero)aryl iodides^a)^. a) All reactions were performed with iodoarenes (0.2 mmol), isocyanides (0.5 mmol), TBD (0.04 mmol), Pd_2_(dba)_3_ (0.01 mmol), Cs_2_CO_3_ (0.4 mmol), and H_2_O (100 µL) in 1.0 mL of DMSO under an Ar atmosphere at 90 °C for 1 h. Isolated yields were given.

We next explored highly active iodoarenes in TBD‐enabled Pd‐catalyzed double insertion chemistry involving isocyanides (**Scheme** **4**).^[^ ^36^ ^]^ Incontrovertibly, aryl iodides with diversified functional groups reacted efficiently with isocyanides and H_2_O to afford desired products **3**, **5**–**7**, **11**–**14**, **16**, **17**, **19**–**22**, **24**, **40**, and **43**–**49** in good‐to‐excellent yields within 1 h. Notably, some sensitive functional groups, including ─NHAc, ─CN, and ─CO_2_Me, also tolerated the current conditions without hydrolysis observed. The substrate scope was successfully extended to heteroaryl iodides, which enabled the corresponding α‐ketoamides with incorporated pyridine (**29**, **30**, and **32**), quinoline (**33**), indole (**50**), pyrazole (**51**), thiophene (**52** and **53**), benzo[*b*]thiophene (**54**), dibenzo[*b*,*d*]furan (**55**), and carbazole (**56**) moieties to be formed in a highly efficient manner (in up to 98% yield). No desired α‐ketoamide was obtained when straight‐chain halogenated hydrocarbons, such as 1‐iodopropane, 1‐bromobutane, 1‐iodopentane, 1‐iodo‐3‐phenylpropane, or 1‐bromo‐4‐phenylbutane, were subjected to the current catalytic conditions, which is possibly ascribable to facile β‐H elimination from the C(sp^3^)‒Pd(II) intermediate. Less sterically hindered alkyl isocyanides, such as *n*‐butyl isocyanide and 1‐fluoro‐2‐(2‐isocyanoethyl)benzene, were compatible with the developed methodology to afford **57** and **58** in good yields, whereas cyclohexyl isocyanide, 2‐isocyano‐2,4,4‐trimethylpentane, and benzyl isocyanide were ineffective, which is possibly attributable to sterically hindered isonitriles interfering with the double insertion process under the current catalytic conditions. Additionally, aryl isocyanides were poor substrates, with the corresponding products not observed, presumably due to facile dimerization of the aryl isocyanide.

### 2.5 Gram Scale Reactions

Four gram‐scale reactions were performed to demonstrate the scalability of the protocol (**Table** **2**). The transformations of bromobenzene and iodobenzene under their respective reaction conditions were amenable to the scaled‐up production of **3**, albeit less efficiently (Table 2, entries 1–3). Pleasingly, a good result (1.4963 g of **3** in 73% yield) was also obtained using only 5 mol% of the palladium catalyst (Table 2, entry 4).

**Table 2:** Gram scale reactions.

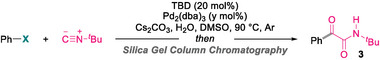					
Entry^a)^	Ph‐X		Pd_2_(dba)_3_	**3**	Yield^b)^
1	X = Br	1.5701 g	10 mol%	1.0378 g	51%
2	X = Br	3.1402 g	10 mol%	2.0629 g	50%
3	X = I	2.0401 g	10 mol%	1.5790 g	77%
4	X = I	2.0401 g	5 mol%	1.4963 g	73%

^a)^ For details, see the Supporting Information;

^b)^ Isolated yields.

### 2.6 Late‐Stage Modifications of Pharmaceuticals and Their Derivatives

**Figure 2:**
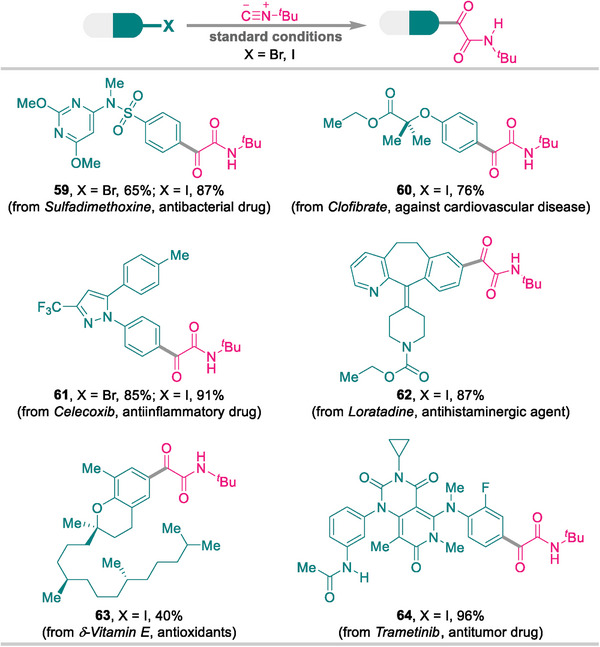
Late‐stage modification of pharmaceuticals and their derivatives.

The application of the developed protocol to the late‐stage modifications of pharmaceuticals and their derivatives further demonstrates the inherent value of this approach. As outlined in **Figure** **2**, drug‐like haloarenes derived from sulfadimethoxine, clofibrate, celecoxib, loratadine, *δ*‐vitamin E, and trametinib reacted smoothly to furnish the corresponding α‐ketoamides **59**–**64** in good‐to‐excellent yields, with only one exception, namely **63**, which was obtained in slightly lower yield.

### 2.7 Synthesis of Pharmaceutically Active Molecules

**Scheme 5:**
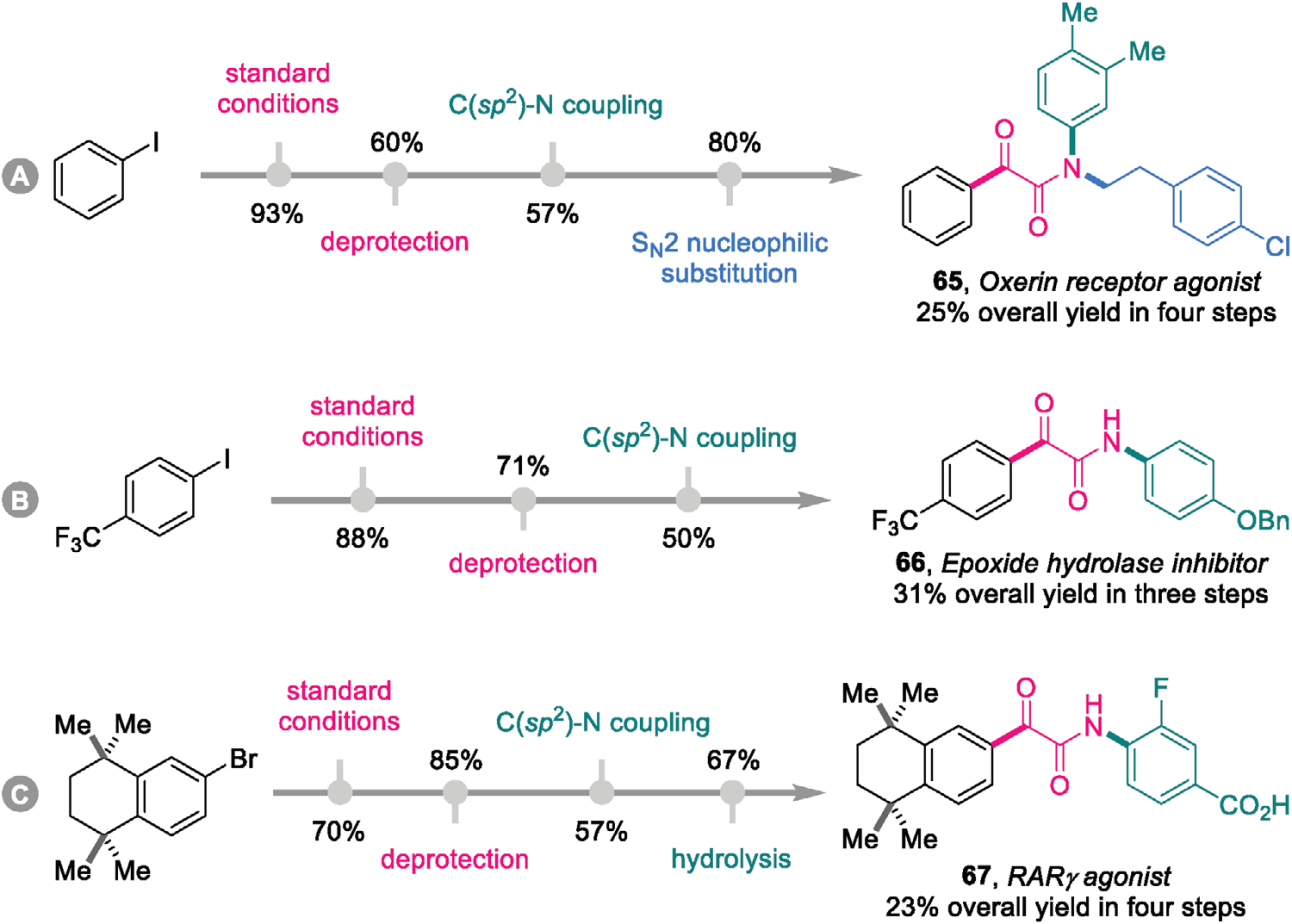
Synthesis of pharmaceutically active molecules^a)^. a) For details, see the Supporting Information.

Considering the frequency of the α‐ketoamide motif in pharmaceutical structures, the developed protocol provides an alternative drug discovery and development methodology. The ligand‐switchable assembly platform was successfully applied to the *de novo* syntheses of three pharmaceutically active molecules. As depicted in **Scheme** **5**, oxerin receptor agonist **65**, epoxide hydrolase inhibitor **66**, and RARγ agonist **67** were smoothly forged in 3–4 steps from safe, inexpensive, and commercially available feedstock chemicals.

### 2.8 Mechanism of the TBD‐Enabled Pd‐Catalyzed Isocyanide Double Insertion Reaction

**Figure 3:**
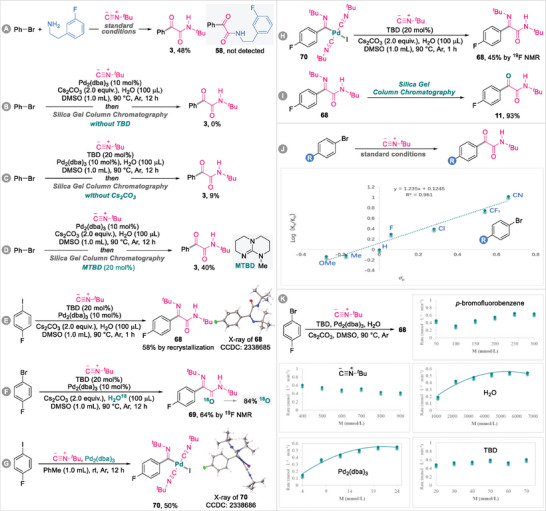
Mechanistic investigation on the TBD enabled Pd‐catalyzed double insertion of isocyanides.

The proposed reaction pathway was investigated using mechanistic experiments. First, cross‐product **58** was not detected when 2‐(3‐fluorophenyl)ethan‐1‐amine was included in the model reaction (**Figure** **3**A), which indicates that acylpalladium species are not involved in the current catalytic system. Compound **3** was not detected in the absence of TBD (Figure 3B), and the desired product was obtained in only 9% yield in the absence of Cs_2_CO_3_ (Figure 3C), which implies that TBD does not act as a base, but likely participates in the reaction as a ligand. To verify this conjecture, lower activity was observed in a control experiment involving MTBD, prepared by methylating the N─H group of TBD (Figure 3D), which is possibly ascribable to steric hindrance or the eliminated hydrogen bond to the imine moiety of the imidoylpalladium(II) intermediate due to the incorporated methyl group. Surprisingly, α‐ketoimine amide **68** was obtained in 58% yield by recrystallization (Figure 3E).^[^ ^37^ ^]^ In addition, ^18^O‐labeled α‐ketoimine amide **69** was generated in 64% yield, which confirms that the oxygen atom in the amide moiety is derived from the H_2_O in the reaction system (Figure 3F). To our delight, we were able to isolate linearly di‐coordinated palladium complex **70** and determine its structure by single‐crystal X‐ray diffractometry (Figure 3G).^[^ ^37^ ^]^ We also attempted but failed to obtain a single crystal of the TBD‐coordinated palladium intermediate, possibly because ring strain rendered it thermodynamically unstable. Similarly, α‐ketoimine amide **68** was obtained in 45% yield when imidoylpalladium complex **70** was subjected to the reaction protocol (Figure 3H). α‐Ketoimine amide **68** was subjected to silica‐gel column chromatography using 20:1 (v/v) petroleum ether/ethyl acetate as the eluant, which afforded hydrolysis product **11** in 93% yield (Figure 3I), and suggests that compounds **68** and **70** are possible intermediates involved in the formation of target product **11**, and that the oxygen atom in the ketone carbonyl group comes from the silica‐gel stationary phase. The influence of electronic effects on the TBD‐enabled Pd‐catalyzed isocyanide double insertion reaction was examined using seven bromoarenes bearing *para*‐substituents (─OMe, ─Me, ─H, ─F, ─Cl, ─CF_3_, and ─CN) under the optimal conditions. As shown in Figure 3J, a linear Hammett plot (*R* ^2^ = 0.961) with a positive *ρ* value of 1.235 was obtained, which indicates that the isocyanide double insertion reaction is clearly facilitated by electron‐withdrawing groups. We finally surveyed the initial‐rate orders of the five individual components [*p*‐bromofluorobenzene, *tert*‐butyl isocyanide, H_2_O, Pd_2_(dba)_3_, and TBD] in the reaction (Figure 3K), which revealed that the reaction is zero‐order with respect to *p*‐bromofluorobenzene, *tert*‐butyl isocyanide, and TBD, suggesting that the oxidative addition of the haloarene to the Pd(0) species, migratory isocyanide insertion, or coordination of the imidoylpalladium(II) intermediate to TBD is not the rate‐limiting step. Remarkably, the kinetic experiment involving H_2_O did not show a simple zero‐ or first‐order relationship, rather saturation kinetics was observed in which the hydrolysis pre‐equilibrium of Cs_2_CO_3_ is involved, which implies that halogen exchange with OH^−^ is possibly the rate‐limiting step. Similarly, a pre‐equilibrium process involving Pd_2_(dba)_3_/[Pd(CN*^t^* Bu)_2_] ligand exchange was determined based on the saturation dependence of Pd_2_(dba)_3_, consistent with a previously reported the mechanistic study into palladium‐catalyzed imidoylative couplings using isocyanides as both substrates and ligands.^[^ ^38^ ^]^

### 2.9 Proposed Mechanism

**Scheme 6:**
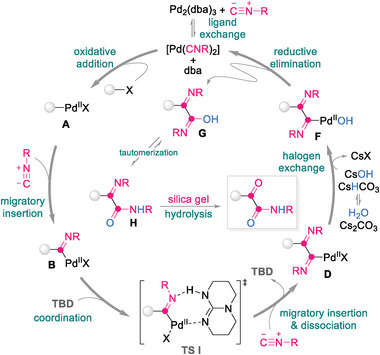
Proposed mechanism (ligands are omitted for clarity).

Based on the experimental results presented above and related literature,^[^ ^24^, ^25^, ^26^, ^27^, ^28^, ^29^, ^30^, ^31^, ^32^, ^33^, ^38^ ^]^ we propose a revised mechanism for the TBD‐enabled Pd‐catalyzed isocyanide double insertion chemistry developed herein (**Scheme** **6**). The [Pd(CNR)_2_] complex is proposed to initially form by Pd_2_(dba)_3_/isocyanide (CNR) ligand exchange, after which imidoylpalladium(II) B is smoothly produced by the oxidative addition of [Pd(CNR)_2_] to the haloarene and the migratory insertion of the isocyanide. Coordination of intermediate B to TBD subsequently forms a chelating seven‐membered ring transition state I (TS I), in which the N─H moiety is associated with the imine of B and the N(sp^2^) atom in TBD is coordinated to the Pd(II) center. The second migratory insertion of the isocyanide is followed by dissociation of the TBD to give α‐ketoimine imidoylpalladium(II) D, which is trapped by OH^−^ generated in situ from Cs_2_CO_3_ and H_2_O to deliver palladium(II) complex F. The α‐ketoamides is finally formed through successive reductive elimination and amide‐iminol tautomerism, followed by silica‐gel‐assisted hydrolysis, while the Pd(0) species is regenerated for participation in the next catalytic cycle.

## 3 Conclusion

We developed a protocol for the selective synthesis of α‐ketoamides from commercially available haloarenes and isocyanides that involves TBD/Pd cooperative catalysis under mild conditions. The efficiency and applicability of this ligand‐switchable assembly platform were demonstrated by its substrate scope, compatibility with late‐stage pharmaceutical modifications, gram‐scale reactions, and the synthesis of pharmaceutically active molecules. Additionally, the reaction mechanism was investigated using crossover reactions, control experiments, an ^18^O‐labeled experiment, by identifying key intermediates, and kinetic studies. Given the flexibility and operational simplicity of the current protocol for forging α‐ketoamides that are of interest in organic synthesis and medicinal chemistry, we anticipate that this strategy will provide new avenues for advancing precision chemistry for assembling molecules.

## Conflict of Interest

The authors declare no conflict of interest.

## Supporting information

Supporting Information

## Data Availability

The data that support the findings of this study are available in the supplementary material of this article.
