# Supplementary material for: De Novo Synthesis of α‐Ketoamides via Pd/TBD Synergistic Catalysis
Source: Adv Sci (Weinh). 2024 Jul 10;11(34):2404266. doi: 10.1002/advs.202404266 (PMC11425860; doi:10.1002/advs.202404266)
Supplement: Supplementary file 1 — Supporting Information [file ADVS-11-2404266-s001.pdf]

ADVANCED SCIENCE
Open Access

Supporting Information

for *Adv. Sci.*, DOI 10.1002/advs.202404266

De Novo Synthesis of α-Ketoamides via Pd/TBD Synergistic Catalysis

*Jia-He Chen, Li-Ren Zhang, Zhang-Yang Wang, Lu-Jie Liu, Li-Ping Tu, Yun Zhang, Yong-Zheng Chen and Wen-Yong Han**

De Novo Synthesis of α-Ketoamides via Pd/TBD Synergistic Catalysis

Jia-He Chen,[a] Li-Ren Zhang,[a] Zhang-Yang Wang,[a] Lu-Jie Liu,[a] Li-Ping Tu,[a] Yun Zhang,[a,b] Yong-Zheng Chen,[a,b] and Wen-Yong Han*[a,b]

[a] Key Laboratory of Biocatalysis & Chiral Drug Synthesis of Guizhou Province, Generic Drug Research Center of Guizhou Province, Green Pharmaceuticals Engineering Research Center of Guizhou Province, School of Pharmacy, Zunyi Medical University, No. 6 West Xuefu Rd. Zunyi 563006 (China)

[b] Key Laboratory of Basic Pharmacology of Ministry of Education and Joint International Research Laboratory of Ethnomedicine of Ministry of Education Zunyi Medical University, No. 6 West Xuefu Rd. Zunyi 563006 (China)

E-mail: hanwy@126.com, hanwy@zmu.edu.cn

Supporting Information

Table of Contents

1. General information S1

2. Optimization of reaction conditions S2

    2.1 Synthesis of **3** from bromobenzene S2

    2.2 Synthesis of **3** from iodobenzene S5

    2.3 Synthesis of **3** from phenyl trifluoromethanesulfonate S9

3. Procedures for the preparation of substrates S11

    3.1 Preparation of **1a** S11

    3.2 Preparation of **1b** S12

    3.3 Preparation of **1c** S13

    3.4 Preparation of **1d** S13

    3.5 Preparation of **1e** S14

    3.6 Preparation of **1f** S14

    3.7 Preparation of **1g** S15

    3.8 Preparation of **1h** S16

    3.9 Preparation of isocyanides **2a** S16

4. Representative procedure for the synthesis of **3** S18

5. Procedures for the synthesis of **65-67** S20

    5.1 Synthesis of **65** from **3** S20

    5.2 Synthesis of **66** from **14** S21

    5.3 Synthesis of **67** from **67-1** S22

    5.4 Synthesis of **68** and **69** S24

    5.5 Synthesis of **70** S24

6. Characterization data of **3-70** S26

7. Gram-scale experiments S52

8. Mechanistic studies S55

    8.1 Control experiment S55

    8.2 Isotope labeling experiment S57

8.3 Control experiment with imidoylpalladium complex S58
8.4 Control experiment with α-ketoimine amide S59
8.5 Hammett's correlation S59
8.6 Kinetic Studies under standard reaction conditions S61
9. Single crystal X-ray diffraction S69
9.1 Cultivation of single crystals S69
9.2 Crystal measurement S69
9.3 X-ray crystallographic data of **6**, **68** and **70** S70
10. References S73
11. Copies of ^{1}H-NMR, ^{13}C-NMR and ^{19}F-NMR spectra S75

1. General information

Unless otherwise noted, all commercially available reagents were used without further purification. All of the solvents were treated according to known methods. Silica gel (300-400 mesh) was purchased from Energy Chemical (China). ^{1}H-NMR (400 MHz) chemical shifts were reported in ppm (*δ*) relative to tetramethylsilane (TMS) with the solvent resonance employed as the internal standard. ^{13}C-NMR (101 MHz) chemical shifts were reported in ppm (*δ*) from tetramethylsilane (TMS) with the solvent resonance as the internal standard. Data were reported as follows: chemical shift, multiplicity (s = singlet, d = doublet, t = triplet, q = quartet, dd = doublet of doublets, td = triplet of doublets, m = multiplet, br s = broad single), coupling constants (Hz) and integration. HRMS measurements were obtained on a Orbitrap analyzer. High-resolution mass spectra were recorded on a Waters Xevo G2-XS QTof spectrometer and Thermo Q Exactive Focus. Melting points were uncorrected. X-ray crystallographic data were collected by a diffractometer Rigaku Oxford Diffraction Supernova Dual Source.

2. Optimization of reaction conditions

2.1 Synthesis of **3** from bromobenzene

Table S1. Screening of ligands, Pd catalysts, solvents and base[a]

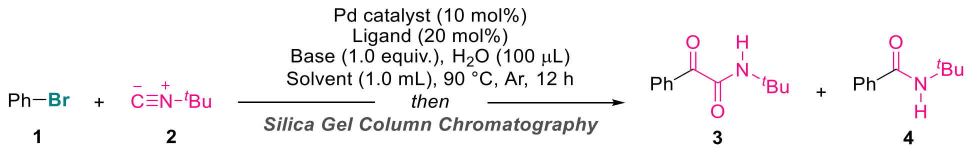

Entry	Ligand	Pd catalyst	Solvent	Base	Yield (%)[b]	
					**3**	**4**
1	L1	$PdCl_2$	DMSO	Cs_2CO_3	36	trace
2	L2	$PdCl_2$	DMSO	Cs_2CO_3	NR	—
3	L3	$PdCl_2$	DMSO	Cs_2CO_3	NR	—
4	L4	$PdCl_2$	DMSO	Cs_2CO_3	NR	—
5	L5	$PdCl_2$	DMSO	Cs_2CO_3	NR	—
6	L6	$PdCl_2$	DMSO	Cs_2CO_3	NR	—
7	L7	$PdCl_2$	DMSO	Cs_2CO_3	49	trace
8[c]	L8	$PdCl_2$	DMSO	Cs_2CO_3	0	74
9	L9	$PdCl_2$	DMSO	Cs_2CO_3	24	trace
10	L10	$PdCl_2$	DMSO	Cs_2CO_3	38	trace
11	L11	$PdCl_2$	DMSO	Cs_2CO_3	NR	—
12	L12	$PdCl_2$	DMSO	Cs_2CO_3	27	trace
13	L7	$Pd(OAc)_2$	DMSO	Cs_2CO_3	32	trace
14	L7	PdI_2	DMSO	Cs_2CO_3	40	—
15	L7	$Pd(TFA)_2$	DMSO	Cs_2CO_3	37	trace
16	L7	$PdCl_2$(dppb)	DMSO	Cs_2CO_3	32	—
17	L7	$PdCl_2$(nbd)	DMSO	Cs_2CO_3	34	—
18	L7	[(π-cinnamyl)PdCl]$_2$	DMSO	Cs_2CO_3	trace	—
19	L7	$PdCl_2$(dippp)	DMSO	Cs_2CO_3	28	—
20	L7	$Pd(OTF)_2$(dippp)	DMSO	Cs_2CO_3	35	—
21	L7	Pd-117	DMSO	Cs_2CO_3	41	—
22	L7	[Pd(allyl)Cl]$_2$	DMSO	Cs_2CO_3	trace	—
**23**	**L7**	**$Pd_2(dba)_3$**	**DMSO**	**Cs_2CO_3**	**63**	**trace**
24	L7	$PdCl_2(CH_3CN)_2$	DMSO	Cs_2CO_3	trace	—
25	L7	$PdCl_2(CH_2CN)_4(BF_4$	DMSO	Cs_2CO_3	33	—
26	L7	$Pd(PPh_3)_4$	DMSO	Cs_2CO_3	34	trace

27	L7	$Pd(OPiv)_2$	DMSO	Cs_2CO_3	41	trace
28	L7	$Pd_2(dba)_3$	DMF	Cs_2CO_3	16	trace
29	L7	$Pd_2(dba)_3$	DMA	Cs_2CO_3	19	—
30	L7	$Pd_2(dba)_3$	1,4-dioxane	Cs_2CO_3	trace	—
31	L7	$Pd_2(dba)_3$	PhMe	Cs_2CO_3	trace	—
32	L7	$Pd_2(dba)_3$	mesitylene	Cs_2CO_3	trace	—
33	L7	$Pd_2(dba)_3$	MeCN	Cs_2CO_3	trace	—
34	L7	$Pd_2(dba)_3$	DCE	Cs_2CO_3	trace	—
35	L7	$Pd_2(dba)_3$	DMSO	CsF	18	trace
36	L7	$Pd_2(dba)_3$	DMSO	KOH	19	—
37	L7	$Pd_2(dba)_3$	DMSO	K_2CO_3	32	trace
38	L7	$Pd_2(dba)_3$	DMSO	^{t}BuOK	22	trace
39	L7	$Pd_2(dba)_3$	DMSO	HCOON	trace	—
40	L7	$Pd_2(dba)_3$	DMSO	Et_3N	trace	—

[a] Reaction conditions: Bromobenzene (**1**, 0.2 mmol), *tert*-butyl isocyanide (**2**, 3.0 equiv., 0.6 mmol), ligand (20 mol%), Pd catalyst (10 mol%), base (1.0 equiv., 0.2 mmol), and H_2O (100 μL) in 1.0 mL of dry solvent under an Ar atmosphere at 90 ºC for 12 h. [b] Isolated yield of **3** and **4** based on bromobenzene is given. [c] Performed with 5 mol% of $PdCl_2$ and 10 mol% of PPh_3. DMSO = dimethyl sulfoxide. DMF = *N*,*N*-dimethylformamide. DMA = *N,N*-Dimethylacetamide. DCE = 1,2-dichloroethane. NR = no reaction.

Table S2. Screening of other reaction parameters[a]

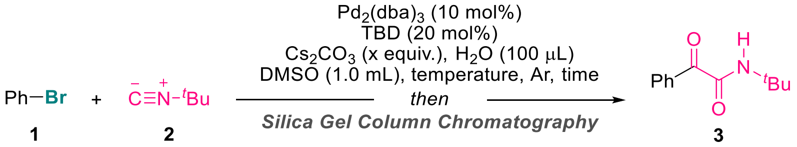

Entry	Cs_2CO_3 (x equiv.)	**2** (y equiv.)	Temperature (ºC)	Time	Yield (%)[b]
1	1.0	3.0	90	12	63
2	2.0	3.0	90	12	77
3	3.0	3.0	90	12	60
4	2.0	2.0	90	12	56
**5**	**2.0**	**2.5**	**90**	**12**	**81**
6	2.0	3.5	90	12	69
7	2.0	4.0	90	12	65
8	2.0	2.5	120	12	37
9	2.0	2.5	90	3	49
10	2.0	2.5	90	6	70
11	2.0	2.5	90	18	76
12[c]	2.0	2.5	90	12	63

[a] Reaction conditions: Bromobenzene (**1**, 0.2 mmol), *tert*-butyl isocyanide (**2**, y equiv., 0.2y mmol), TBD (20 mol%), $Pd_2(dba)_3$ (10 mol%), Cs_2CO_3 (x equiv., 0.2x mmol), and H_2O (100 μL) in dry DMSO (1.0 mL) under an Ar atmosphere at 90−120 ºC for 3−18 h. [b] Isolated yield of **3** based on bromobenzene is given. [c] Performed with 5 mol% of $Pd_2(dba)_3$.

2.2 Synthesis of **3** from iodobenzene

Table S3. Screening of ligands, Pd-catalysts, solvents and base[a]

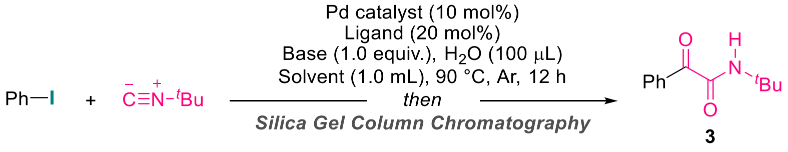

Entry	Ligand	Pd catalyst	Solvent	Base	Yield (%)[b]
1	L1	$PdCl_2$	DMSO	Cs_2CO_3	31
2	L2	$PdCl_2$	DMSO	Cs_2CO_3	trace
3	L3	$PdCl_2$	DMSO	Cs_2CO_3	NR
4	L4	$PdCl_2$	DMSO	Cs_2CO_3	NR
5	L5	$PdCl_2$	DMSO	Cs_2CO_3	NR
6	L6	$PdCl_2$	DMSO	Cs_2CO_3	NR
7	L7	$PdCl_2$	DMSO	Cs_2CO_3	62
8	L8	$PdCl_2$	DMSO	Cs_2CO_3	NR
9	L9	$PdCl_2$	DMSO	Cs_2CO_3	53
10	L10	$PdCl_2$	DMSO	Cs_2CO_3	44
11	L11	$PdCl_2$	DMSO	Cs_2CO_3	NR
12	L12	$PdCl_2$	DMSO	Cs_2CO_3	43
13	L13	$PdCl_2$	DMSO	Cs_2CO_3	43
14	L14	$PdCl_2$	DMSO	Cs_2CO_3	trace
15	L15	$PdCl_2$	DMSO	Cs_2CO_3	trace
16	L16	$PdCl_2$	DMSO	Cs_2CO_3	NR
17	L17	$PdCl_2$	DMSO	Cs_2CO_3	trace
18	L7	$Pd(OAc)_2$	DMSO	Cs_2CO_3	36
19	L7	$PdBr_2$	DMSO	Cs_2CO_3	37
20	L7	$PdCl_2(PPh_3)_2$	DMSO	Cs_2CO_3	49
21	L7	$Pd(TFA)_2$	DMSO	Cs_2CO_3	53
22	L7	$PdCl_2$(nbd)	DMSO	Cs_2CO_3	65
23	L7	[(π-cinnamyl)PdCl]$_2$	DMSO	Cs_2CO_3	29
24	L7	$PdCl_2$(dppb)	DMSO	Cs_2CO_3	36
25	L7	Pd(dppf)$_2$Cl$_2$	DMSO	Cs_2CO_3	39
26	L7	$PdCl_2$(dippp)	DMSO	Cs_2CO_3	trace
27	L7	PdI_2	DMSO	Cs_2CO_3	30
28	L7	$PdCl_2(CH_3CN)_2$	DMSO	Cs_2CO_3	50

29	L7	$PdCl_2(CH_2CN)_4(BF_4)_2$	DMSO	Cs_2CO_3	51
30	L7	Pd-117	DMSO	Cs_2CO_3	51
31	L7	$Pd(PPh_3)_4$	DMSO	Cs_2CO_3	52
**32**	**L7**	**$Pd_2(dba)_3$**	**DMSO**	**Cs_2CO_3**	70
33	L7	$Pd(OPiv)_2$	DMSO	Cs_2CO_3	25
34	L7	$Pd(OTf)_2$(dippp)	DMSO	Cs_2CO_3	26
35	L7	1,3-bis[2,6-bis(1-methyl ethyl)phenyl]-1,3-dihydro-2*H*-imid azol-2-ylidene]chloro (η3-2-propen-1-yl)palladium	DMSO	Cs_2CO_3	52
36	L7	dichloro[1,3-bis(diisopropylphenyl)-2-imidazolidinylidene]palladium(II)dimer	DMSO	Cs_2CO_3	trace
37	L7	$[PdCl(2\text{-}(dimethyl\ amino)methylphenyl)]_2$	DMSO	Cs_2CO_3	67
38	L7	$Pd_2(dba)_3$	DMF	Cs_2CO_3	23
39	L7	$Pd_2(dba)_3$	DMA	Cs_2CO_3	24
40	L7	$Pd_2(dba)_3$	1,4-dioxane	Cs_2CO_3	trace
41	L7	$Pd_2(dba)_3$	NMP	Cs_2CO_3	24
42	L7	$Pd_2(dba)_3$	HMPA	Cs_2CO_3	22
43	L7	$Pd_2(dba)_3$	toluene	Cs_2CO_3	trace
44	L7	$Pd_2(dba)_3$	benzotrifluoride	Cs_2CO_3	trace
45	L7	$Pd_2(dba)_3$	mesitylene	Cs_2CO_3	trace
46	L7	$Pd_2(dba)_3$	MeCN	Cs_2CO_3	trace
47	L7	$Pd_2(dba)_3$	DCE	Cs_2CO_3	trace
48	L7	$Pd_2(dba)_3$	cyclohexane	Cs_2CO_3	NR
49	L7	$Pd_2(dba)_3$	DMSO	CsF	12
50	L7	$Pd_2(dba)_3$	DMSO	KOH	24
51	L7	$Pd_2(dba)_3$	DMSO	K_2CO_3	21
52	L7	$Pd_2(dba)_3$	DMSO	^{t}BuOK	17

[a] Reaction conditions: Iodobenzene (0.2 mmol), *tert*-butyl isocyanide (3.0 equiv., 0.6 mmol), Pd catalyst (10 mol%), base (1.0 equiv., 0.2 mmol), and H_2O (100 μL) in 1.0 mL of dry solvent under an Ar atmosphere at 90 ºC for 12 h. [b] Isolated yield of **3** based on iodobenzene is given. DMSO = dimethyl sulfoxide. DMF = *N*,*N*-dimethylformamide. DMA = *N,N*-Dimethylacetamide. NMP = *N*-methylpyrrolidone. HMPA = Hexamethylphosphoramide. DCE = 1,2-dichloroethane. NR = no reaction.

Table S4. Screening of other reaction parameters[a]

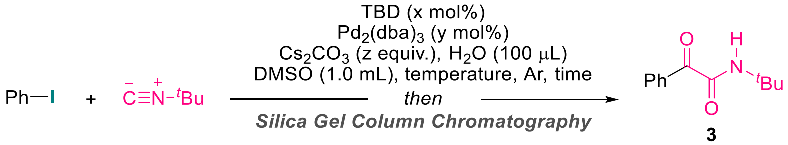

Entry	Time	Isocyanide (w equiv.)	Cs_2CO_3 (z equiv.)	$Pd_2(dba)_3$ (y mol%)	TBD (x mol%)	Temperature (°C)	Yield (%)[b]
1	12 h	3.0	1.0	10	20	90	70
2	4 h	3.0	1.0	10	20	90	64
3	1 h	3.0	1.0	10	20	90	74
4	50 min	3.0	1.0	10	20	90	65
5	30 min	3.0	1.0	10	20	90	58
6	20 min	3.0	1.0	10	20	90	33
7	10 min	3.0	1.0	10	20	90	21
8	1 h	2.0	1.0	10	20	90	70
9	1 h	2.5	1.0	10	20	90	75
10	1 h	3.0	1.0	10	20	90	77
11	1 h	3.5	1.0	10	20	90	77
12	1 h	2.5	1.5	10	20	90	84
**13**	**1 h**	**2.5**	**2.0**	**10**	**20**	**90**	**93**
14	1 h	2.5	2.5	10	20	90	84
15	1 h	2.5	2.0	5	20	90	54
16	1 h	2.5	2.0	10	10	90	46

17	1 h	2.5	2.0	10	20	80	72
18	1 h	2.5	2.0	10	20	70	34

[a] Reaction conditions: Iodobenzene (0.2 mmol), *tert*-butyl isocyanide (w equiv., 0.2w mmol), TBD (x mol%), $Pd_2(dba)_3$ (y mol%), Cs_2CO_3 (z equiv., 0.2z mmol), and H_2O (100 µL) in dry DMSO (1.0 mL) under an Ar atmosphere at 70−90 ºC for 10 min−12 h. [b] Isolated yield of **3** based on iodobenzene is given.

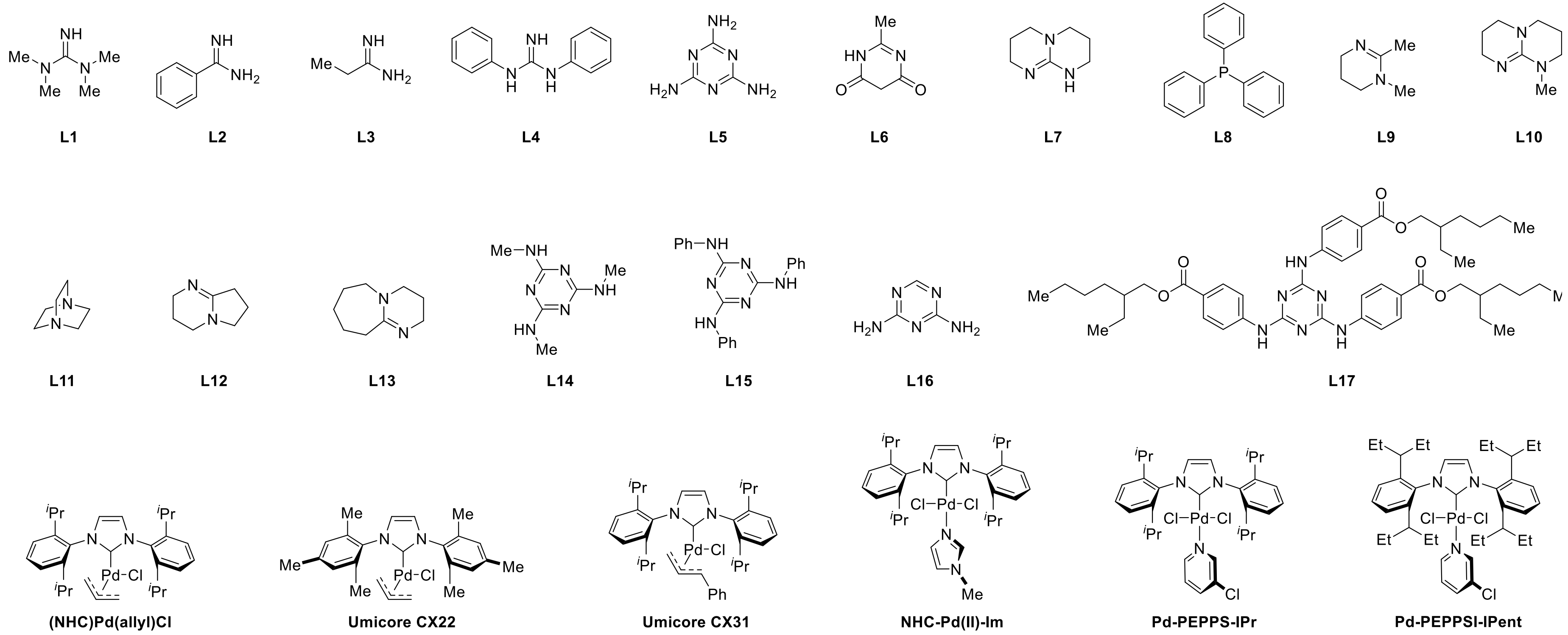

Figure S1. Ligands and palladium complexes examined in the optimization of reaction conditions for the synthesis of **3**

2.3 Synthesis of **3** from phenyl trifluoromethanesulfonate

Table S5. Screening of temperature, time, Pd-catalysts and base[a]

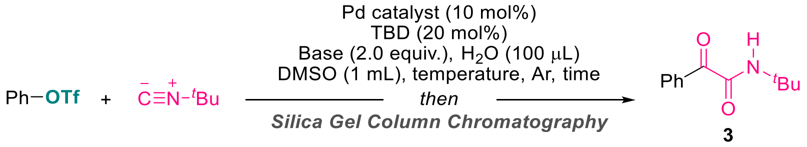

Entry	Temperature (ºC)	Time (h)	Pd catalyst	Base	Yield (%)[b]
1	90	6	$Pd_2(dba)_3$	Cs_2CO_3	8
2	90	12	$Pd_2(dba)_3$	Cs_2CO_3	9
3	120	6	$Pd_2(dba)_3$	Cs_2CO_3	13
4	120	12	$Pd_2(dba)_3$	Cs_2CO_3	13
5	150	6	$Pd_2(dba)_3$	Cs_2CO_3	11
6	150	12	$Pd_2(dba)_3$	Cs_2CO_3	10
7	120	6	$Pd(dba)_2$	Cs_2CO_3	13
8	120	6	$PdCl_2$	Cs_2CO_3	25
9	120	6	$Pd(OAc)_2$	Cs_2CO_3	12
10	120	6	$PdBr_2$	Cs_2CO_3	8
11	120	6	$PdCl_2(PPh_3)_2$	Cs_2CO_3	trace
12	120	6	$Pd(TFA)_2$	Cs_2CO_3	trace
13	120	6	$PdCl_2$(nbd)	Cs_2CO_3	9
14	120	6	$[(\pi\text{-cinnamyl})PdCl]_2$	Cs_2CO_3	20
15	120	6	$PdCl_2$(dppb)	Cs_2CO_3	trace
16	120	6	$Pd(dppf)_2Cl_2$	Cs_2CO_3	trace
17	120	6	$PdCl_2$(dippp)	Cs_2CO_3	trace
18	120	6	PdI_2	Cs_2CO_3	18
19	120	6	$PdCl_2(CH_3CN)_2$	Cs_2CO_3	trace
20	120	6	$PdCl_2(CH_2CN)_4(BF_4)_2$	Cs_2CO_3	trace
**21**	**120**	**6**	**Pd-117**	**Cs_2CO_3**	**36**
22	120	6	$Pd(PPh_3)_4$	Cs_2CO_3	trace
23	120	6	$Pd(OPiv)_2$	Cs_2CO_3	trace
24	120	6	$Pd(OTF)_2$(dippp)	Cs_2CO_3	16
25	120	6	(NHC)Pd(allyl)Cl	Cs_2CO_3	trace
26	120	6	Umicore CX22	Cs_2CO_3	trace
27	120	6	Umicore CX31	Cs_2CO_3	trace
28	120	6	NHC-Pd(II)-Im	Cs_2CO_3	trace

29	120	6	Pd-PEPPS-IPr	Cs_2CO_3	trace
30	120	6	Pd-PEPPSI-IPent	Cs_2CO_3	trace
31	120	6	Pd-117	CsF	trace
32	120	6	Pd-117	KOH	trace
33	120	6	Pd-117	K_2CO_3	trace
34	120	6	Pd-117	^{t}BuOK	trace
35	120	6	Pd-117	Et_3N	trace
36	120	6	Pd-117	$NaOCH_3$	trace
37	120	6	Pd-117	CsOPiv	trace
38	120	6	Pd-117	K_3PO_4	trace
39	120	6	Pd-117	NaCNO	trace
40	120	6	Pd-117	DBU	trace
41	120	6	Pd-117	BABCO	trace
42	120	6	Pd-117	CsOH	trace
43	120	6	Pd-117	CsF	trace
44	120	6	Pd-117	KOH	trace
45	120	6	Pd-117	K_2CO_3	trace
46	120	6	Pd-117	^{t}BuOK	trace

[a] Reaction conditions: Phenyl trifluoromethanesulfonate (0.2 mmol), *tert*-butyl isocyanide (2.5 equiv., 0.5 mmol), TBD (20 mol%), Pd catalyst (10 mol%), base (2.0 equiv., 0.4 mmol), and H_2O (100 μL) in dry DMSO (1.0 mL) under an Ar atmosphere at 90−150 ºC for 6−12 h. [b] Isolated yield of **3** based on phenyl trifluoromethanesulfonate is given.

3. Procedures for the preparation of substrates

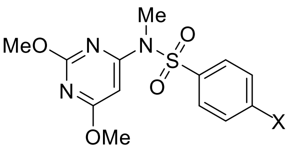

1a, X = Br
1b, X = I

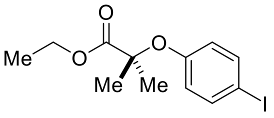

1c

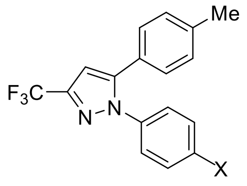

1d, X = Br
1e, X = I

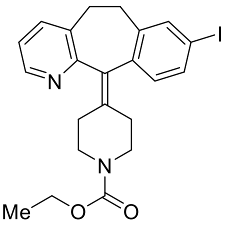

1f

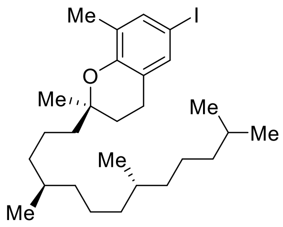

1g

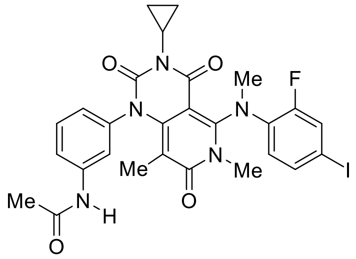

1h

3.1 Preparation of **1a**

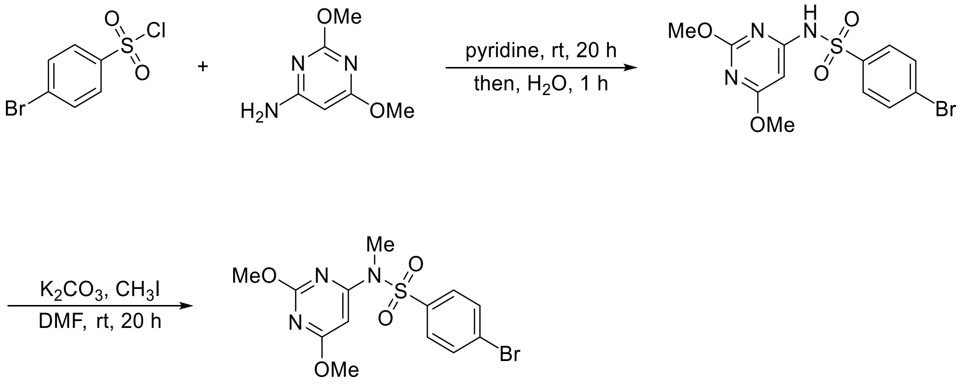

1a

A mixture of 4-bromobenzenesulfonyl chloride (2.55 g, 10.0 mmol, 1.0 equiv.) and 2,6-dimethoxypyrimidin-4-amine (1.62 g, 10.5 mmol, 1.05 equiv.) in pyridine (25 mL) was stirred for 20 hours at room temperature. To the solution was then added H_2O (50 mL) to produce a slurry. After stirring for 1 hour, the slurry was filtered, and the filter cake was washed with water (25 mL), after which the solid was dried in vacuo to yield the compound as a white solid (2.61 g, 70% yield).

A mixture of 4-bromo-*N*-(2,6-dimethoxypyrimidin-4-yl)benzenesulfonamide (1.86 g, 5.0 mmol, 1.0 equiv.), K_2CO_3 (0.69 g, 5.0 mmol, 1.0 equiv.), and iodomethane (0.47 mL, 7.5 mmol, 1.5 equiv.) in DMF (15 mL) was stirred for 20 hours at room temperature. Afterwards, the reaction mixture was diluted with EtOAc (30 mL) and washed with water

(30 mL). The aqueous layer was back-extracted with EtOAc (3 × 30 mL). The combined organic layers were then dried over Na_2SO_4, filtered, and concentrated in vacuo.[1] Purification by petroleum ether/ethyl acetate (10:1) provided the compound **1a** as a white solid (1.18 g, 61% yield).

3.2 Preparation of **1b**

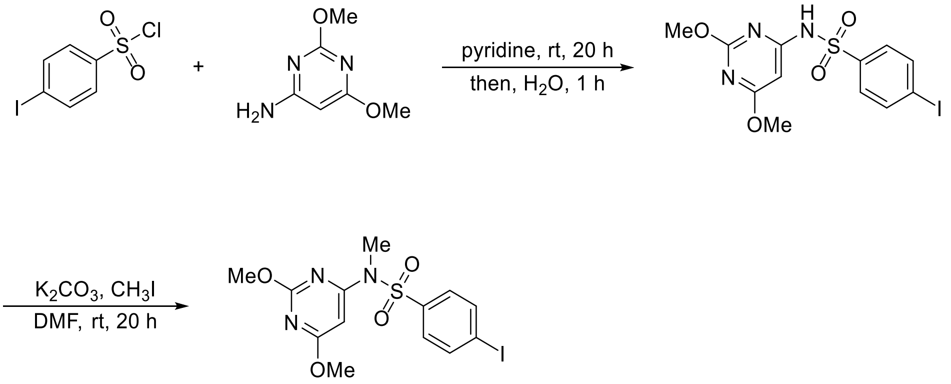

1b

A mixture of 4-Iodobenzenesulfonyl chloride (3.03 g, 10.0 mmol, 1.0 equiv.) and 2,6-dimethoxypyrimidin-4-amine (1.62 g, 10.5 mmol, 1.05 equiv.) in pyridine (25 mL) was stirred for 20 hours at room temperature. To the solution was then added H_2O (50 mL) to produce a slurry. After stirring for 1 hour, the slurry was filtered, and the filter cake was washed with water (25 mL), after which the solid was dried in vacuo to yield the compound as a white solid (3.07 g, 73% yield).

A mixture of 4-bromo-*N*-(2,6-dimethoxypyrimidin-4-yl)benzenesulfonamide (2.11 g, 5.0 mmol, 1.0 equiv.), K_2CO_3 (0.69 g, 5.0 mmol, 1.0 equiv.), and iodomethane (0.47 mL, 7.5 mmol, 1.5 equiv.) in DMF (15 mL) was stirred for 20 hours at room temperature. Afterwards, the reaction mixture was diluted with EtOAc (30 mL) and washed with water (30 mL). The aqueous layer was back-extracted with EtOAc (3 × 30 mL). The combined organic layers were then dried over Na_2SO_4, filtered, and concentrated in vacuo.[1] Purification by petroleum ether/ethyl acetate (10:1) provided the compound **1b** as a white solid (1.41 g, 65% yield).

3.3 Preparation of **1c**

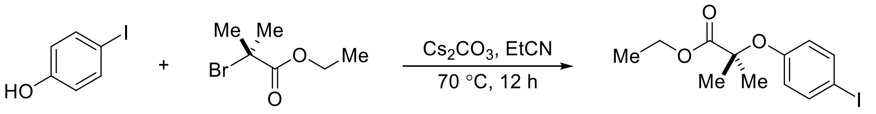

1c

A flame-dried 100-mL round-bottom flask, equipped with a stir bar, was charged with 4-iodophenol (440.0 mg, 2.0 mmol, 1.0 equiv.), ethyl 2-bromo-2-methylpropanoate (390.1 mg, 2.0 mmol, 1.0 equiv.), cesium carbonate (390.1 mg, 4.0 mmol, 2.0 equiv.), and MeCN (10 mL). The mixture was allowed to stir for 12 h at 70 ºC. The mixture was then allowed to cool to room temperature and diluted with water (15 mL). The aqueous solution was then washed with ethyl acetate (10 mL x 3). The combined organic layers were dried over Na_2SO_4 and filtered.[2] The solvent was removed by rotary evaporation and the residue was fully loaded onto a silica gel column and was eluted with petroleum ether/ethyl acetate (30:1 – 10:1) to give the compound **1c** as a colorless oil (621.3 mg, 93% yield).

3.4 Preparation of **1d**

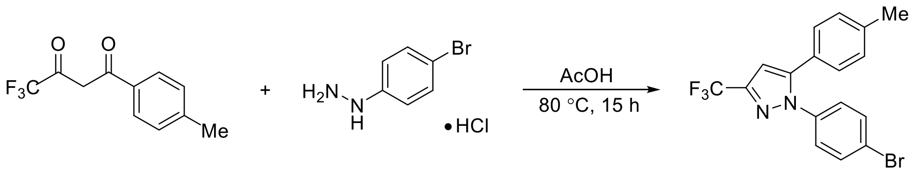

1d

A Schlenk tube containing (4-iodophenyl)hydrazine hydrochloride (443.9 mg, 2.0 mmol, 1.0 equiv.) and 4,4,4-trifluoro-1-(p-tolyl)butane-1,3-dione (460.4 mg, 2.0 mmol, 1.0 equiv.) was evacuated and back-filled with anhydrous dinitrogen three times. Degassed acetic acid (10 mL) was added and the reaction mixture was stirred at 80 ºC for 15 h. After cooling to room temperature, the reaction mixture was concentrated in vacuo.[3] The residue was fully loaded onto a silica gel column and was eluted with petroleum ether/ethyl acetate (20:1 – 10:1) to give the compound **1d** as a yellow solid (653.6 mg, 86% yield).

3.5 Preparation of **1e**

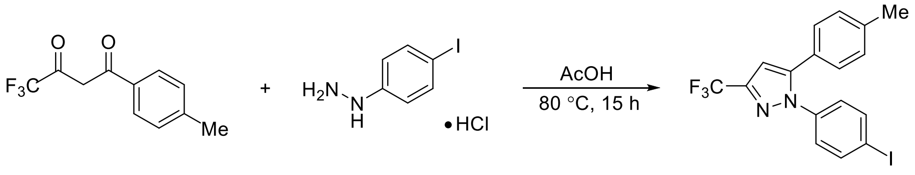

1e

A Schlenk tube containing (4-iodophenyl)hydrazine hydrochloride (541.0 mg, 2.0 mmol, 1.0 equiv.) and 4,4,4-trifluoro-1-(p-tolyl)butane-1,3-dione (460.4 mg, 2.0 mmol, 1.0 equiv.) was evacuated and back-filled with anhydrous dinitrogen three times. Degassed acetic acid (10 mL) was added and the reaction mixture was stirred at 80 ºC for 15 h. After cooling to room temperature, the reaction mixture was concentrated in vacuo.[3] The residue was fully loaded onto a silica gel column and was eluted with petroleum ether/ethyl acetate (20:1 – 10:1) to give the compound **1e** as a white solid (721.8 mg, 84% yield).

3.6 Preparation of **1f**

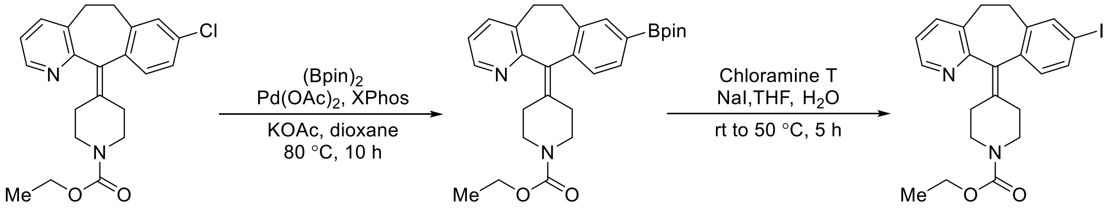

1f

An oven dried 40 mL flask was charged with Loratadine (1.15 g, 3.0 mmol, 1.0 equiv.), bis(pinacolato)diboron (0.91 g, 4.5 mmol, 1.2 equiv.), KOAc (0.59 g, 6.0 mmol, 2.0 equiv.), $Pd(OAc)_2$ (33.7 mg, 0.15 mmol, 5 mol%), XPhos (143.0 mg, 0.3 mmol, 10 mol%). They were transferred into a nitrogen-filled glovebox. Freshly distilled 1,4-dioxane (15 mL) was added to the flask in the glovebox.The flask was tightly sealed, transferred out of glovebox and stirred on a pie-block preheated to 80 ºC for 10 h. The reaction mixture was filtered through Celite and taken up in 100 mL 2N HCl/ ethyl acetate. The layers were separated and the organic phase was discarded. The aqueous phase was then washed with ethyl acetate (15 mL × 3). After careful adjustment of pH to basic by adding 10N KOH (in the ice bath), the resulting slurry was extracted with ethyl acetate (25 mL × 3).[4] The combined organic phase was washed with brine, dried over Na_2SO_4, and concentrated to give a white

solid (1.00 g, 70% yield).

The product (0.95 g 2.1 mmol) from the last step was dissolved in tetrahydrofuran (10 mL) and water (10 mL), and stirred at room temperature for 15 min. NaI (0.98 g, 4.3 mmol, 2.05 equiv.) and Chloramine T (0.61 g, 4.1 mmol, 1.95 equiv.) were then added at room temperature. After heating at 50 ºC for 5 h, yellow solid precipitated from the solution. The reaction mixture was then diluted with water (20 mL) and extracted with CH_2Cl_2 (20 mL × 3). The combined organic phase was washed with brine, dried over Na_2SO_4, and concentrated to give a yellowish solid.[5] The residue was fully loaded onto a silica gel column and was eluted with petroleum ether/ethyl acetate (20:1) to give the compound **2f** as a white solid (811.9 mg, 82% yield).

3.7 Preparation of **1g**

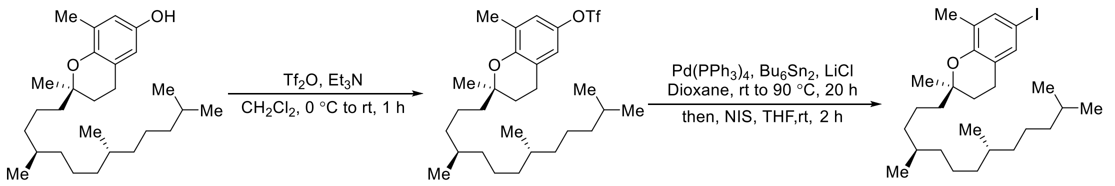

1g

To a stirred solution of *δ*-tocopherol (805.3 mg, 2.0 mmol, 1.0 equiv.), triethylamine (834.0 μL, 6.0 mmol, 3.0 equiv.) in CH_2Cl_2 (10 mL) was added trifluoromethanesulfonic anhydride (370.1 μL, 2.2 mmol, 1.1 equiv.) dropwise at 0 ºC. The resulting mixture was warmed up to room temperature and stirred for 1 h. The resulting mixture was quenched with saturated $NaHCO_3$ (20 mL), extracted with CH_2Cl_2 (20 mL × 3), dried over anhydrous Na_2SO_4, filtered, and concentrated under reduced pressure.[6] The residue was fully loaded onto a silica gel column and was eluted with petroleum ether/ethyl acetate (20:1) to give a yellow oil (1.03 g, 97% yield).

To a flask permanently connected to a reflux condenser was added and $Pd(PPh_3)_4$ (115.6 mg, 5mol%), LiCl (423.9 mg, 10.0 mmol, 5.0 equiv.), and bis(tributyltin) (2.32 g, 4.0 mmol, 2.0 equiv.) under argon atmosphere. Added to the mixture was the yellow oil (1.07 g, 2.0 mmol, 1.0 equiv.) from the last step and 20 mL dioxane. The mixture was heated to 90 ºC and stirred 20 h. After cooling to rt, 50 mL hexanes added, the mixture was filtered through celite and solvents were evaporated to give crude product. Without further

purification the product from the last step, it was dissolved in 75 mL THF followed by N-iodosuccinimide (1.35 g, 6.0 mmol, 3.0 equiv.). The mixture stirred for 2 h at rt. The reaction mixture was poured into saturated $Na_2S_2O_3$ and extracted with EtOAc (100 mL × 3). The organic layers were washed with brine and dried over Na_2SO_4. After rotary evaporation the mixture solvent was concentrated by rotary evaporation to obtain the residue.[7] The residue was fully loaded onto a silica gel column and was eluted with petroleum ether/ethyl acetate (100:1) to give **1g** as a clear oil (881.1 mg, 86% yield).

3.8 Preparation of **1h**

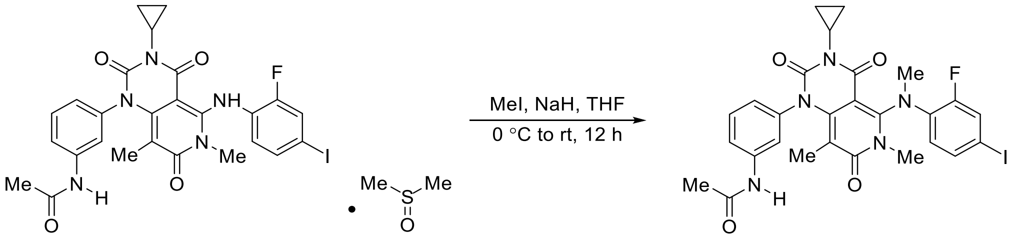

1h

A 50 mL single-necked flask with a magnetic stir bar was charged with Trametinib (1.39 g, 2.0 mmol, 1.0 equiv.) and dissolved in THF (10 mL). Add NaH (120.0 mg, 3.0 mmol, 1.5 equiv.) to the reaction mixture at 0 ºC and stir for 10 min, then add Iodomethane (186.8 μL, 3.0 mmol, 1.5 equiv.). After the addition was completed, the reaction mixture was stirred at 0 ºC for 10 min and then at room temperature for 12 h. After the completion of the reaction monitored by TLC (thin layer chromatography), the solvent was concentrated by rotary evaporation to obtain the residue.[8] The residue was fully loaded onto a silica gel column and was eluted with petroleum ether/ethyl acetate (1:1) to give **1h** as a white solid (943.6 mg, 75% yield).

3.9 Preparation of isocyanides **2a**

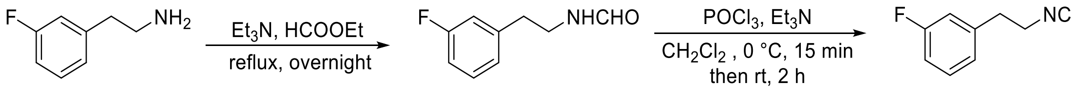

2a

A 50 mL single-necked flask equipped with a stir bar and a reflux condenser was charged with a mixture of 2-(3-fluorophenyl)ethan-1-amine (695.4 mg, 5.0 mmol, 1.0

equiv.), triethylamine (1.39 mL, 10.0 mmol, 2.0 equiv.) and ethyl formate (12.3 mL, 150.0 mmol, 30.0 equiv.). The reaction mixture was stirred and heated under reflux overnight. After the completion of the reaction monitored by TLC (thin layer chromatography), the solvent was concentrated by rotary evaporation to obtain the formamide as a residue, which was used directly in the next step without purification.

The residue prepared by above method was dissolved in CH_2Cl_2 (20 mL) and then triethylamine was added (2.08 mL, 15.0 mmol, 3.0 equiv.). The mixture was cooled at 0 ºC and then the solution of $POCl_3$ (513 μL, 5.5 mmol, 1.1 equiv.) in 10 mL of CH_2Cl_2 was added dropwise to the mixture over 30 min. After the addition was completed, the reaction mixture was stirred at 0 ºC for 15 min and then at room temperature for 2 h. After the completion of the reaction monitored by TLC (thin layer chromatography), an ice-cold saturated $NaHCO_3$ solution (20 mL) was added to the mixture and extracted with CH_2Cl_2 (3 × 20 mL). The combined organic layers were washed with saturated K_2CO_3 (3 × 60 mL), dried over anhydrous Na_2SO_4, filtered and concentrated by rotary evaporation.[9-11] The residue was fully loaded onto a silica gel column and was eluted with petroleum ether/ethyl acetate (20:1) to give 1-fluoro-3-(2-isocyanoethyl)benzene **2a** as a brown oil (693.1 mg, 93% yield).

4. Representative procedure for the synthesis of 3

*Synthesis of compound **3** from bromobenzene*

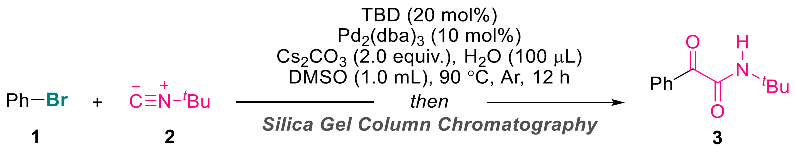

A 4-mL flame-dried vial with a magnetic stir bar was charged with bromobenzene (20.9 μL, 0.2 mmol), TBD (5.6 mg, 20 mol%), $Pd_2(dba)_3$ (18.3 mg, 10 mol%), Cs_2CO_3 (130.3 mg, 0.4 mmol), H_2O (100 μL) and extra dry DMSO (1.0 mL). The vial was evacuated and backfilled with argon for three times and then *tert*-butyl isocyanide (56.6 μL, 0.5 mmol) was added by syringe under argon. The vial was then sealed and was stirred at 90 ºC (oil bath) for 12 h. After the completion of the reaction monitored by TLC (thin layer chromatography), the reaction mixture was cooled to the room temperature and treated with water (15 mL), extracted with CH_2Cl_2 (3 × 15 mL), washed with brine (2 × 40 mL). Then the organic layer was dried over anhydrous Na_2SO_4, filtered and concentrated. The residue was purified by flash column chromatography on silica gel (10 g) and eluted with petroleum ether/ethyl acetate (30:1 – 10:1) to afford the product **3** (33.2 mg, 81% yield).

*Synthesis of compound **3** from iodobenzene*

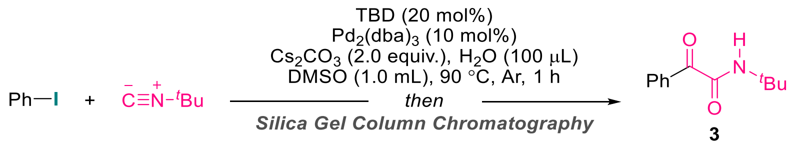

A 4-mL flame-dried vial with a magnetic stir bar was charged with iodobenzene (22.4 μL, 0.2 mmol), TBD (5.6 mg, 20 mol%), $Pd_2(dba)_3$ (18.3 mg, 10 mol%), Cs_2CO_3 (130.3 mg, 0.4 mmol), H_2O (100 μL) and extra dry DMSO (1.0 mL). The vial was evacuated and backfilled with argon for three times and then *tert*-butyl isocyanide (56.6 μL, 0.5 mmol) was added by syringe under argon. The vial was then sealed and was stirred at 90 ºC (oil bath) for 1 h. After the completion of the reaction monitored by TLC (thin layer chromatography), the reaction mixture was cooled to the room temperature and treated with water (15 mL), extracted with CH_2Cl_2 (3 × 15 mL), washed with brine (2 × 40 mL). Then the organic layer was dried over anhydrous Na_2SO_4, filtered and concentrated. The residue was purified by flash column chromatography on silica gel (10 g) and eluted with petroleum ether/ethyl

acetate (30:1 – 10:1) to afford the product **3** (38.2 mg, 93% yield).

*Synthesis of compound **3** from phenyl trifluoromethanesulfonate*

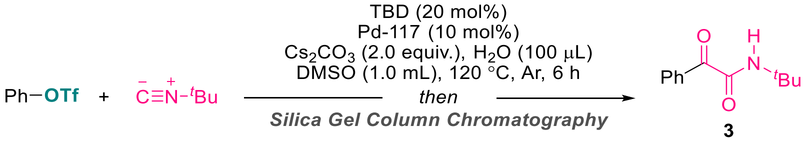

3

A 4-mL flame-dried vial with a magnetic stir bar was charged with phenyl trifluoromethanesulfonate (32.4 μL, 0.2 mmol), TBD (5.6 mg, 20 mol%), Pd-117 (14.3 mg, 10 mol%), Cs_2CO_3 (130.3 mg, 0.4 mmol), H_2O (100 μL) and extra dry DMSO (1.0 mL). The vial was evacuated and backfilled with argon for three times and then *tert*-butyl isocyanide (56.6 μL, 0.5 mmol) was added by syringe under argon. The vial was then sealed and was stirred at 120 ºC (oil bath) for 6 h. After the completion of the reaction monitored by TLC (thin layer chromatography), the reaction mixture was cooled to the room temperature and treated with water (15 mL), extracted with CH_2Cl_2 (3 × 15 mL), washed with brine (2 × 40 mL). Then the organic layer was dried over anhydrous Na_2SO_4, filtered and concentrated. The residue was purified by flash column chromatography on silica gel (10 g) and eluted with petroleum ether/ethyl acetate (30:1 – 10:1) to afford the product **3** (14.8 mg, 36% yield).

Products **5** – **64** were synthesized following these procedures.

5. Procedures for the synthesis of 65-67

5.1 Synthesis of **65** from **3**

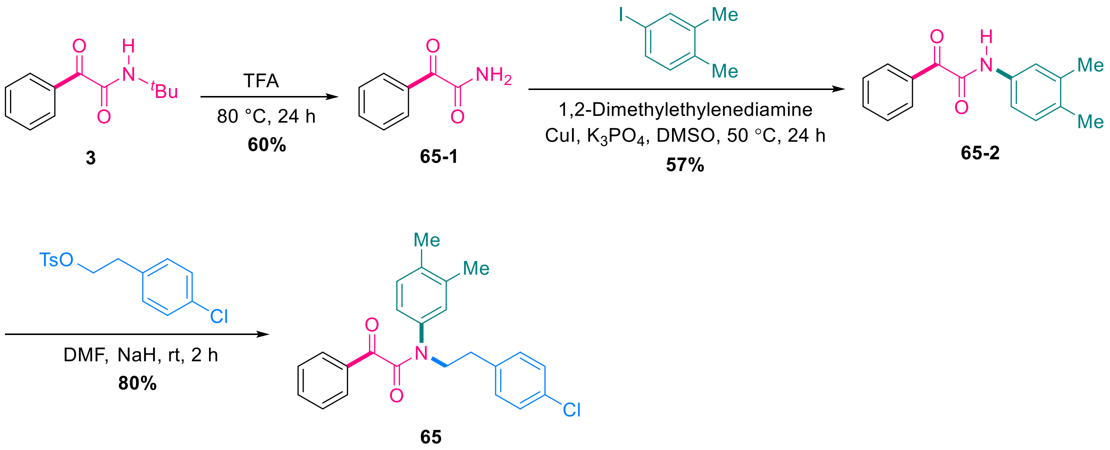

A 10-mL pressure tube with a magnetic stir bar was charged with **3** (41.0 mg, 0.2 mmol, 1.0 equiv.) and trifluoroacetic acid (2.0 mL). The mixture was stirred at 80 ˚C for 24 h. After the completion of the reaction monitored by TLC (thin layer chromatography), the reaction mixture was diluted with water (30 mL) and extracted with CH_2Cl_2 (3 × 30 mL). The combined organic layers were washed with water (60 mL) and saturated $NaHCO_3$ solution (2 × 60 mL), dried over Na_2SO_4 and filtered.[12] The solvent was concentrated by rotary evaporation and the residue was fully loaded onto a silica gel column and eluted with petroleum ether/ethyl acetate (10:1 – 1:1) to afford compound **65-1** as a white solid (17.9 mg, 60% yield).

A mixture of **65-1** (29.8 mg, 0.2 mmol, 1.0 equiv.), 4-iodo-1,2-dimethylbenzene (55.7 mg, 0.24 mmol, 1.2 equiv.), 1,2-Dimethylethylenediamine (1.8 mg, 10 mol%), CuI (3.8 mg, 10 mol%), K_3PO_4 (84.9 mg, 2.0 equiv.) and DMSO (1.0 mL). The vial was evacuated and backfilled with argon for three times and stirred at 50 ˚C for 24 h. After the completion of the reaction monitored by TLC (thin layer chromatography), the reaction mixture was cooled to the room temperature and treated with water (15 mL), extracted with CH_2Cl_2 (3 × 15 mL), washed with brine (2 × 40 mL). Then the organic layer was dried over anhydrous Na_2SO_4, filtered and concentrated.[13] The residue was purified by flash column chromatography on silica gel and eluted with petroleum ether/ethyl acetate (10:1 – 5:1) to afford the product **65-2** as a yellow solid (28.9 mg, 57% yield).

A 10 mL single-necked flask with a magnetic stir bar was charged with **65-2** (50.6 mg,

0.2 mmol, 1.0 equiv.) and dissolved in DMF (1.0 mL). Add NaH (5.8 mg, 0.24 mmol, 1.2 equiv.) to the reaction mixture at 0 ºC and stir for 10 min, then add 4-chlorophenethyl 4-methylbenzenesulfonate (74.4 mg, 0.24 mmol, 1.2 equiv.). After the addition was completed, the reaction mixture was stirred at 0 ºC for 10 min and then at room temperature for 2 h. After the completion of the reaction monitored by TLC (thin layer chromatography), the reaction mixture was treated with water (15 mL), extracted with CH_2Cl_2 (3 × 15 mL), washed with brine (2 × 40 mL). Then the organic layer was dried over anhydrous Na_2SO_4, filtered and concentrated.the solvent was concentrated by rotary evaporation to obtain a residue. The residue was fully loaded onto a silica gel column and was eluted with petroleum ether/ethyl acetate (5:1) to give **65** as a white solid (62.6 mg, 80% yield).

5.2 Synthesis of **66** from **14**

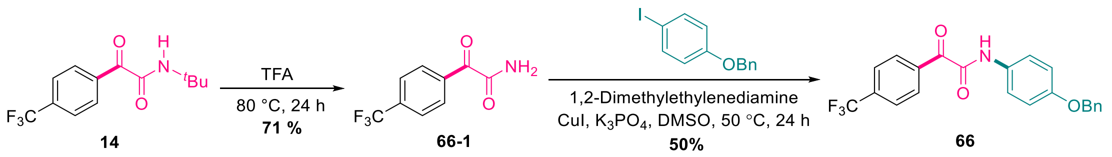

A 10-mL pressure tube with a magnetic stir bar was charged with **14** (54.6 mg, 0.2 mmol, 1.0 equiv.) and trifluoroacetic acid (2.0 mL). The mixture was stirred at 80 ˚C for 24 h. After the completion of the reaction monitored by TLC (thin layer chromatography), the reaction mixture was diluted with water (30 mL) and extracted with CH_2Cl_2 (3 × 30 mL). The combined organic layers were washed with water (60 mL) and saturated $NaHCO_3$ solution (2 × 60 mL), dried over Na_2SO_4 and filtered.[12] The solvent was concentrated by rotary evaporation and the residue was fully loaded onto a silica gel column and eluted with petroleum ether/ethyl acetate (10:1 – 1:1) to afford compound **66-1** as a white solid (30.8 mg, 71% yield).

A mixture of **66-1** (43.4 mg, 0.2 mmol, 1.0 equiv.), 1-(benzyloxy)-4-iodobenzene (74.4 mg, 0.24 mmol, 1.2 equiv.), 1,2-Dimethylethylenediamine (1.8 mg, 10 mol%), CuI (3.8 mg, 10 mol%), K_3PO_4 (84.9 mg, 2.0 equiv.) and DMSO (1.0 mL). The vial was evacuated and backfilled with argon for three times and stirred at 50 ˚C for 24 h. After the completion of the reaction monitored by TLC (thin layer chromatography), the reaction mixture was cooled to the room temperature and treated with water (15 mL), extracted with CH_2Cl_2 (3

× 15 mL), washed with brine (2 × 40 mL). Then the organic layer was dried over anhydrous Na_2SO_4, filtered and concentrated.[13] The residue was purified by flash column chromatography on silica gel and eluted with petroleum ether/ethyl acetate (10:1 – 5:1) to afford the product **66** as a yellow solid (39.9 mg, 50% yield).

5.3 Synthesis of **67** from **67-1**

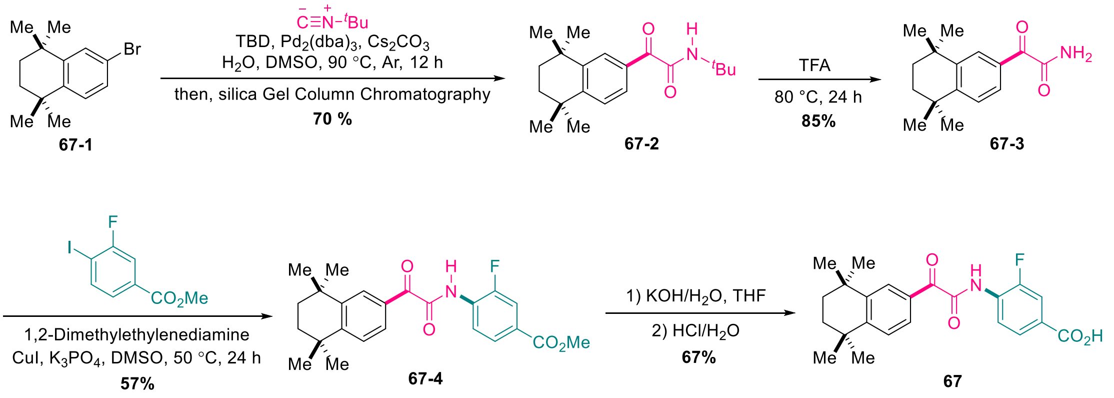

A 4-mL flame-dried vial with a magnetic stir bar was charged with **67-1** (53.2 mg, 0.2 mmol, 1.0 equiv.), TBD (5.6 mg, 20 mol%), $Pd_2(dba)_3$ (18.3 mg, 10 mol%), Cs_2CO_3 (130.3 mg, 0.4 mmol, 2.0 equiv.), H_2O (100 μL) and extra dry DMSO (1.0 mL). The vial was evacuated and backfilled with argon for three times and then *tert*-butyl isocyanide (56.6 μL, 0.5 mmol) was added by syringe under argon. The vial was then sealed and was stirred at 90 ºC (oil bath) for 12 h. After the completion of the reaction monitored by TLC (thin layer chromatography), the reaction mixture was cooled to the room temperature and treated with water (15 mL), extracted with CH_2Cl_2 (3 × 15 mL), washed with brine (2 × 40 mL). Then the organic layer was dried over anhydrous Na_2SO_4, filtered and concentrated. The residue was purified by flash column chromatography on silica gel and eluted with petroleum ether/ethyl acetate (30:1 – 10:1) to afford the product **67-2** (44.1 mg, 70% yield).

A 10-mL pressure tube with a magnetic stir bar was charged with **67-2** (63.0 mg, 0.2 mmol, 1.0 equiv.) and trifluoroacetic acid (2.0 mL). The mixture was stirred at 80 ˚C for 24 h. After the completion of the reaction monitored by TLC (thin layer chromatography), the reaction mixture was diluted with water (30 mL) and extracted with CH_2Cl_2 (3 × 30 mL). The combined organic layers were washed with water (60 mL) and saturated $NaHCO_3$ solution (2 × 60 mL), dried over Na_2SO_4 and filtered.[12] The solvent was concentrated by

rotary evaporation and the residue was fully loaded onto a silica gel column and eluted with petroleum ether/ethyl acetate (10:1 – 1:1) to afford compound **67-3** as a yellow solid (44.1 mg, 85% yield).

A mixture of **67-3** (51.8 mg, 0.2 mmol, 1.0 equiv.), methyl 3-fluoro-4-iodobenzoate (67.2 mg, 0.24 mmol, 1.2 equiv.), 1,2-Dimethylethylenediamine (1.8 mg, 10 mol%), CuI (3.8 mg, 10 mol%), K_3PO_4 (84.9 mg, 2.0 equiv.) and DMSO (1.0 mL). The vial was evacuated and backfilled with argon for three times and stirred at 50 ˚C for 24 h. After the completion of the reaction monitored by TLC (thin layer chromatography), the reaction mixture was cooled to the room temperature and treated with water (15 mL), extracted with CH_2Cl_2 (3 × 15 mL), washed with brine (2 × 40 mL). Then the organic layer was dried over anhydrous Na_2SO_4, filtered and concentrated.[13] The residue was purified by flash column chromatography on silica gel and eluted with petroleum ether/ethyl acetate (10:1 – 5:1) to afford the product **67-4** as a yellow solid (46.9 mg, 57% yield).

A 50 mL single-necked flask with a magnetic stir bar was charged with **67-4** (82.2 mg, 0.2 mmol, 1.0 equiv.), KOH (56.1 mg, 1.0 mmol, 5.0 equiv.), dissolved in H_2O (5.0 mL) and THF (5.0 mL). After the addition was completed, the reaction mixture was stirred at room temperature for 6 h. After the completion of the reaction monitored by TLC (thin layer chromatography), the reaction mixture was acidified with HCl/H_2O (1.0 mol/L), a white precipitate can be observed. Then the reaction mixture was treated with water (15 mL), extracted with CH_2Cl_2 (3 × 15 mL), and the organic layer was dried over anhydrous Na_2SO_4, filtered and concentrated.the solvent was concentrated by rotary evaporation to obtain a residue. The residue was fully loaded onto a silica gel column and was eluted with petroleum ether/ethyl acetate (5:1) to give **67** as a white solid (53.4 mg, 67% yield).

5.4 Synthesis of **68** and **69**

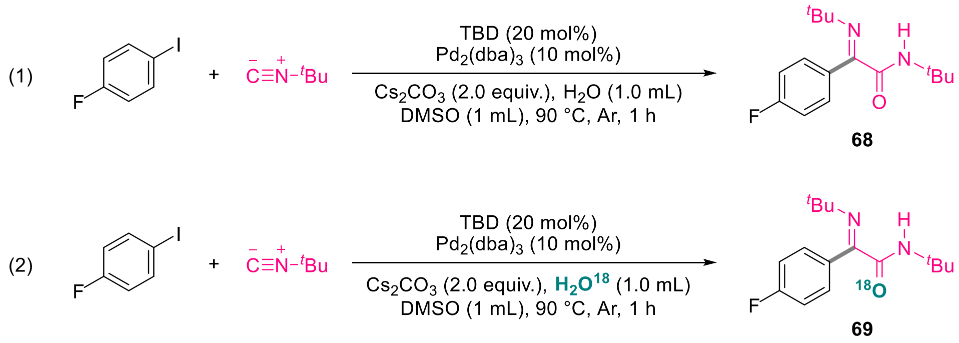

A 25-mL flame-dried flask with a magnetic stir bar was charged with 1-fluoro-4-iodobenzene (230.6 μL, 2.0 mmol, 1.0 equiv.), TBD (55.7 mg, 20 mol%), $Pd_2(dba)_3$ (183.0 mg, 10 mol%), Cs_2CO_3 (1303.3 mg, 4.0 mmol, 2.0 equiv.), H_2O (1.0 mL) and extra dry DMSO (5.0 mL). The flask was evacuated and backfilled with argon for three times and then *tert*-butyl isocyanide (565.5 μL, 5.0 mmol, 2.5 equiv.) was added by syringe under argon. The flask was then sealed and was stirred at 90 ºC (oil bath) for 1 h. After the completion of the reaction monitored by TLC (thin layer chromatography), the reaction mixture was cooled to the room temperature and treated with water (50 mL), extracted with CH_2Cl_2 (3 × 50 mL), washed with brine (2 × 150 mL). Then the organic layer was dried over anhydrous Na_2SO_4, filtered and concentrated. The residue was recrystallization with petroleum ether and dichloromethane. Finally, the product **68** was obtained as a yellow solid (323.6 mg, 58% yield by recrystallization). Product **69** was synthesized following these procedures.

5.5 Synthesis of **70**

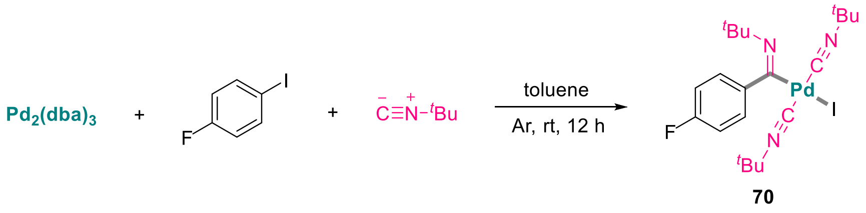

A 10-mL flame-dried flask with a magnetic stir bar was charged with $Pd_2(dba)_3$ (91.5 mg, 0.1 mmol, 1.0 equiv.), 1-fluoro-4-iodobenzene (46.1 μL, 0.4 mmol, 4.0 equiv.), dissolved in dry toluene (1.0 mL), and the flask was evacuated and backfilled with argon for three times and then *tert*-butyl isocyanide (90.5 μL, 0.8 mmol, 8.0 equiv.) was added by

syringe under argon. After the addition was completed, the flask was sealed and was stirred at room temperature for 12 h. After the completion of the reaction monitored by TLC (thin layer chromatography), the reaction concentrated by rotary evaporation to obtain a residue.[14] The residue was fully loaded onto a silica gel column and was eluted with petroleum ether/ethyl acetate (10:1 – 3:1) to give the compound **70** as a yellow solid (57.7 mg, 50% yield).

6. Characterization data of 3-70

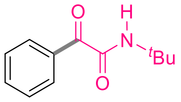

3

***N*-(*tert*-butyl)-2-oxo-2-phenylacetamide (3).** Yellow solid, 33.2 mg, 81% yield (from bromobenzene); 14.8 mg, 36% yield (from phenyl trifluoromethanesulfonate); 38.2 mg, 93% yield (from iodobenzene), m.p. = 72.8 – 74.3 ºC. **^{1}H-NMR** ($CDCl_3$, 400 MHz): *δ* 8.29 (d, *J* = 7.2 Hz, 2H), 7.60 (t, *J* = 7.4 Hz, 1H), 7.46 (t, *J* = 7.7 Hz, 2H), 6.94 (br s, 1H), 1.45 (s, 9H); **^{13}C-NMR** ($CDCl_3$, 101 MHz): *δ* 188.7, 161.2, 134.3, 133.5, 131.3, 128.5, 51.8, 28.5; **HRMS** (ESI-Orbitrap): calcd. for $[C_{12}H_{16}NO_2]^+$ ($M + H^+$): 206.1176, found: 206.1173.

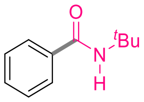

4

***N*-(*tert*-butyl)benzamide (4).** White solid, 26.2 mg, 74% yield (from bromobenzene), m.p. = 130.4 – 131.3 ºC. **^{1}H-NMR** ($CDCl_3$, 400 MHz): *δ* 7.65 (d, *J* = 7.3 Hz, 2H), 7.35 (t, *J* = 7.3 Hz, 1H), 7.28 (t, *J* = 7.4 Hz, 2H), 6.18 (br s, 1H), 1.39 (s, 9H); **^{13}C-NMR** ($CDCl_3$, 101 MHz): *δ* 166.9, 135.7, 130.9, 128.2, 126.7, 51.4, 28.7; **HRMS** (ESI-Orbitrap): calcd. for $[C_{11}H_{16}NO]^+$ ($M + H^+$): 178.1226, found: 178.1226.

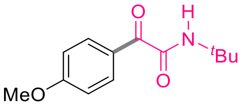

5

***N*-(*tert*-butyl)-2-(4-methoxyphenyl)-2-oxoacetamide (5).** White solid, 38.6 mg, 82% yield (from 1-bromo-4-methoxybenzene); 30.1 mg, 64% yield (from 1-iodo-4-methoxybenzene), m.p. = 60.3 – 62.9 ºC. **^{1}H-NMR** ($CDCl_3$, 400 MHz): *δ* 8.36 (d, *J* = 9.0 Hz, 2H), 6.98 (br s, 1H), 6.91 (d, *J* = 9.0 Hz, 2H), 3.86 (s, 3H), 1.43 (s, 9H); **^{13}C-NMR** ($CDCl_3$, 101 MHz): *δ* 186.6, 164.6, 161.8, 134.0, 126.5, 113.8, 55.6, 51.6, 28.5; **HRMS** (ESI-Orbitrap): calcd. for $[C_{13}H_{17}NNaO_3]^+$ ($M + Na^+$): 258.1101, found: 258.1101.

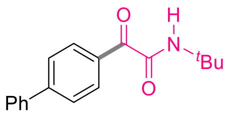

6

2-([1,1'-biphenyl]-4-yl)-*N*-(*tert*-butyl)-2-oxoacetamide (6). Yellow solid, 41.6 mg, 74% yield, (from 4-bromo-1,1'-biphenyl); 11.8 mg, 21% yield {from [1,1'-biphenyl]-4-yl trifluoromethanesulfonate}; 41.6 mg, 74% yield (from 4-iodo-1,1'-biphenyl), m.p. = 130.0 – 131.9 ºC. **^{1}H-NMR** ($CDCl_3$, 400 MHz): δ 8.41 (d, J = 8.5 Hz, 2H), 7.68 (d, J = 8.5 Hz, 2H), 7.63 (d, J = 7.2 Hz, 2H), 7.47 (t, J = 7.4 Hz, 2H), 7.40 (t, J = 7.3 Hz, 1H), 7.02 (br s, 1H), 1.48 (s, 9H); **^{13}C-NMR** ($CDCl_3$, 101 MHz): δ 188.0, 161.3, 146.8, 139.8, 132.2, 131.9, 129.0, 128.5, 127.4, 127.1, 51.7, 28.5; **HRMS** (ESI-Orbitrap): calcd. for $[C_{18}H_{19}NNaO_2]^+$ (M + Na^+): 304.1308, found: 304.1301.

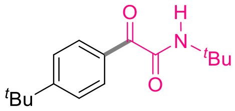

7

***N*-(*tert*-butyl)-2-(4-(*tert*-butyl)phenyl)-2-oxoacetamide (7).** Yellow solid, 43.4 mg, 83% yield, [from 1-bromo-4-(*tert*-butyl)benzene]; 10.4 mg, 20% yield [from 4-(*tert*-butyl)phenyl trifluoromethanesulfonate]; 44.9 mg, 86% yield [from 1-(*tert*-butyl)-4-iodobenzene], m.p. = 73.3 – 74.2 ºC. **^{1}H-NMR** ($CDCl_3$, 400 MHz): δ 8.23 (d, J = 8.7 Hz, 2H), 7.46 (d, J = 8.7 Hz, 2H), 6.94 (br s, 1H), 1.44 (s, 9H), 1.32 (s, 9H); **^{13}C-NMR** ($CDCl_3$, 101 MHz): δ 188.3, 161.5, 158.1, 131.3, 130.9, 125.5, 51.7, 35.3, 31.1, 28.5; **HRMS** (ESI-Orbitrap): calcd. for $[C_{16}H_{24}NO_2]^+$ (M + H^+): 262.1802, found: 262.1801.

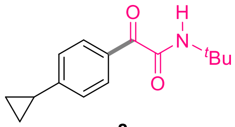

8

***N*-(*tert*-butyl)-2-(4-cyclopropylphenyl)-2-oxoacetamide (8).** Yellow solid, 25.5 mg, 52% yield (from 1-bromo-4-cyclopropylbenzene), m.p. = 77.6 – 79.1 ºC. **^{1}H-NMR** ($CDCl_3$, 400 MHz): δ 8.21 (d, J = 8.3 Hz, 2H), 7.09 (d, J = 8.3 Hz, 2H), 6.95 (br s, 1H), 2.01 – 1.85 (m, 1H), 1.43 (s, 9H), 1.13 – 0.99 (m, 2H), 0.85 – 0.72 (m, 2H); **^{13}C-NMR** ($CDCl_3$, 101 MHz): δ

187.9, 161.6, 152.0, 131.5, 130.7, 125.3, 51.7, 28.5, 16.1, 10.9; **HRMS** (ESI-Orbitrap): calcd. for $[C_{15}H_{20}NO_2]^+$ (M + H$^+$): 246.1489, found: 246.1489.

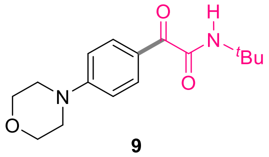

9

***N*-(*tert*-butyl)-2-(4-morpholinophenyl)-2-oxoacetamide (9).** Yellow solid, 45.8 mg, 79% yield [from 4-(4-bromophenyl)morpholine], m.p. = 90.9 – 92.3 ºC. **^{1}H-NMR** ($CDCl_3$, 400 MHz): δ 8.33 (d, J = 9.1 Hz, 2H), 7.01 (br s, 1H), 6.82 (d, J = 9.1 Hz, 2H), 3.82 (t, 4H), 3.33 (t, 4H), 1.42 (s, 9H); **^{13}C-NMR** ($CDCl_3$, 101 MHz): δ 185.7, 162.3, 154.9, 133.8, 123.8, 112.8, 66.5, 51.5, 47.1, 28.5; **HRMS** (ESI-Orbitrap): calcd. for $[C_{16}H_{23}N_2O_3]^+$ (M + H$^+$): 291.1703, found: 291.1706.

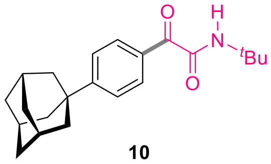

10

2-(4-((1*s*,3*R*,5*S*,7*s*)-adamantan-1-yl)phenyl)-*N*-(*tert*-butyl)-2-oxoacetamide (10). Yellow solid, 34.6 mg, 51% yield [from (1*S*,3*R*,5*S*,7*S*)-1-(4-bromophenyl)adamantine], m.p. = 134.9 – 136.7 ºC. **^{1}H-NMR** ($CDCl_3$, 400 MHz): δ 8.25 (d, J = 8.5 Hz, 2H), 7.44 (d, J = 8.6 Hz, 2H), 6.96 (br s, 1H), 2.10 (s, 3H), 1.90 (s, 6H), 1.76 (q, J = 12.4 Hz, 6H), 1.44 (s, 9H); **^{13}C-NMR** ($CDCl_3$, 101 MHz): δ 188.2, 161.5, 158.2, 131.3, 130.8, 125.1, 51.6, 42.8, 36.9, 36.7, 28.8, 28.4; **HRMS** (ESI-Orbitrap): calcd. for $[C_{22}H_{30}NO_2]^+$ (M + H$^+$): 340.2271, found: 340.2273.

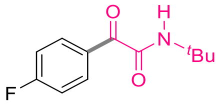

11

***N*-(*tert*-butyl)-2-(4-fluorophenyl)-2-oxoacetamide (11).** White solid, 33.5 mg, 75% yield (from 1-bromo-4-fluorobenzene); 9.4 mg, 21% yield (from 4-fluorophenyl trifluoromethanesulfonate); 43.7 mg, 98% yield (From 1-fluoro-4-iodobenzene), m.p. = 48.4

– 49.3 ºC. **^{1}H-NMR** ($CDCl_3$, 400 MHz): *δ* 8.43 – 8.31 (m, 2H), 7.10 (t, *J* = 8.7 Hz, 2H), 6.99 (br s, 1H), 1.42 (s, 9H); **^{13}C-NMR** ($CDCl_3$, 101 MHz): *δ* 186.7, 166.5 (d, J = 257.2 Hz), 161.0, 134.3 (d, *J* = 9.6 Hz), 129.8 (d, *J* = 3.0 Hz), 115.7 (d, *J* = 21.8 Hz), 51.8, 28.4; **^{19}F NMR** ($CDCl_3$, 376 MHz) *δ* -99.04 – -106.36 (m); **HRMS** (ESI-Orbitrap): calcd. for $[C_{12}H_{15}FNO_2]^+$ ($M + H^+$): 224.1081, found: 224.1090.

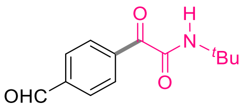

12

***N*-(*tert*-butyl)-2-(4-formylphenyl)-2-oxoacetamide (12).** Yellow solid, 39.2 mg, 84% yield (from 4-bromobenzaldehyde); 40.1 mg, 86% yield (from 4-iodobenzaldehyde), m.p. = 28.5 – 30.4 ºC. **^{1}H-NMR** ($CDCl_3$, 400 MHz): *δ* 10.06 (s, 1H), 8.38 (d, *J* = 8.2 Hz, 2H), 7.91 (d, *J* = 8.5 Hz, 2H), 6.98 (br s, 1H), 1.42 (s, 9H); **^{13}C-NMR** ($CDCl_3$, 101 MHz): *δ* 191.8, 188.0, 160.4, 139.4, 137.8, 131.7, 129.3, 51.9, 28.4; **HRMS** (ESI-Orbitrap): calcd. for $[C_{13}H_{16}NO_3]^+$ ($M + H^+$): 234.1125, found: 234.1123.

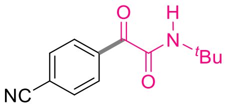

13

***N*-(*tert*-butyl)-2-(4-cyanophenyl)-2-oxoacetamide (13).** Yellow solid, 40.0 mg, 87% yield (from 4-bromobenzonitrile); 41.0 mg, 89% yield (from 4-iodobenzonitrile), m.p. = 108.8 – 110.3 ºC. **^{1}H-NMR** ($CDCl_3$, 400 MHz): *δ* 8.37 (d, *J* = 8.5 Hz, 2H), 7.73 (d, *J* = 8.5 Hz, 2H), 6.97 (br s, 1H), 1.42 (s, 9H); **^{13}C-NMR** ($CDCl_3$, 101 MHz): *δ* 187.2, 160.0, 136.6, 132.1, 131.6, 117.9, 117.1, 52.0, 28.3; **HRMS** (ESI-Orbitrap): calcd. for $[C_{13}H_{15}N_2O_2]^+$ ($M + H^+$): 231.1128, found: 231.1124.

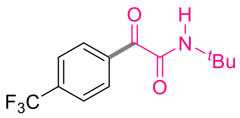

14

***N*-(*tert*-butyl)-2-oxo-2-(4-(trifluoromethyl)phenyl)acetamide (14).** White solid, 41.5 mg, 76% yield [from 1-bromo-4-(trifluoromethyl)benzene]; 14.2 mg, 26% yield [from 4-(trifluoromethyl)phenyl trifluoromethanesulfonate]; 48.1 mg, 88% yield [from 1-iodo-4-(trifluoromethyl)benzene], m.p. = 83.9 – 85.9ºC. **^{1}H-NMR** ($CDCl_3$, 400 MHz): *δ* 8.39 (d, *J* = 8.1 Hz, 2H), 7.70 (d, *J* = 8.3 Hz, 2H), 6.99 (br s, 1H), 1.44 (s, 9H); **^{13}C-NMR** ($CDCl_3$, 101 MHz): *δ* 187.6, 160.4, 136.2 (d, *J* = 1.1 Hz), 135.1 (q, *J* = 32.7 Hz), 131.6, 125.4 (q, *J* = 3.7 Hz), 123.6 (q, *J* = 272.9 Hz), 52.0, 28.4; **HRMS** (ESI-Orbitrap): calcd. for $[C_{13}H_{15}F_3NO_2]^+$ (M + H^+): 274.1049, found: 274.1051.

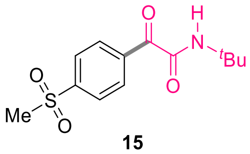

15

***N*-(*tert*-butyl)-2-(4-(methylsulfonyl)phenyl)-2-oxoacetamide (15).** Yellow solid, 34.5 mg, 61% yield [from 1-bromo-4-(methylsulfonyl)benzene], m.p. = 122.4 – 124.5 ºC. **^{1}H-NMR** ($CDCl_3$, 400 MHz): *δ* 8.45 (d, *J* = 8.7 Hz, 2H), 8.03 (d, *J* = 8.7 Hz, 2H), 6.99 (br s, 1H), 3.07 (s, 3H), 1.45 (s, 9H); **^{13}C-NMR** ($CDCl_3$, 101 MHz): *δ* 187.5, 160.0, 144.7, 137.6, 132.1, 127.4, 52.1, 44.4, 28.4; **HRMS** (ESI-Orbitrap): calcd. for $[C_{13}H_{18}NO_4S]^+$ (M + H^+): 284.0951, found: 284.0956.

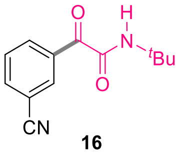

16

***N*-(*tert*-butyl)-2-(3-cyanophenyl)-2-oxoacetamide (16).** Yellow solid, 30.4 mg, 66% yield (from 3-bromobenzonitrile); 40.5 mg, 88% yield (from 3-iodobenzonitrile), m.p. = 70.3 – 71.2 ºC. **^{1}H-NMR** ($CDCl_3$, 400 MHz): *δ* 8.63 (s, 1H), 8.49 (d, *J* = 8.0 Hz, 1H), 7.83 (d, *J* = 7.8 Hz, 1H), 7.57 (t, *J* = 7.9 Hz, 1H), 7.00 (br s, 1H), 1.42 (s, 9H); **^{13}C-NMR** ($CDCl_3$, 101

MHz): δ 186.3, 160.0, 136.8, 135.1, 135.0, 134.2, 129.4, 117.8, 112.9, 52.0, 28.3; **HRMS** (ESI-Orbitrap): calcd. for $[C_{13}H_{15}N_2O_2]^+$ (M + H$^+$): 231.1128, found: 231.1122.

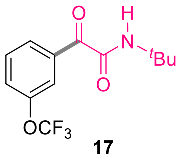

17

***N*-(*tert*-butyl)-2-oxo-2-(3-(trifluoromethoxy)phenyl)acetamide (17).** Yellow oil, 42.8 mg, 74% yield [from 1-bromo-3-(trifluoromethoxy)benzene]; 47.4 mg, 82% yield [from 1-iodo-3-(trifluoromethoxy)benzene]. **^{1}H-NMR** ($CDCl_3$, 400 MHz): δ 8.27 (d, J = 7.6 Hz, 1H), 8.17 (s, 1H), 7.49 (dd, J = 11.9, 3.9 Hz, 1H), 7.43 (d, J = 8.3 Hz, 1H), 6.98 (br s, 1H), 1.43 (s, 9H); **^{13}C-NMR** ($CDCl_3$, 101 MHz): δ 187.0, 160.5, 149.2 (q, J = 1.9 Hz), 135.2, 130.0, 129.8, 126.6, 123.6 (d, J = 0.6 Hz), 120.5 (q, J = 258.0 Hz), 51.9, 28.4; **HRMS** (ESI-Orbitrap): calcd. for $[C_{13}H_{15}F_3NO_3]^+$ (M + H$^+$): 290.0999, found: 290.0999.

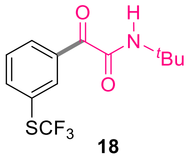

18

***N*-(*tert*-butyl)-2-oxo-2-(3-((trifluoromethyl)thio)phenyl)acetamide (18).** Yellow oil, 50.0 mg, 82% yield [from (3-bromophenyl)(trifluoromethyl)sulfane]. **^{1}H-NMR** ($CDCl_3$, 400 MHz): δ 8.59 (s, 1H), 8.46 (d, J = 7.9 Hz, 1H), 7.88 (d, J = 7.7 Hz, 1H), 7.54 (t, J = 7.8 Hz, 1H), 6.99 (br s, 1H), 1.45 (s, 9H); **^{13}C-NMR** ($CDCl_3$, 101 MHz): δ 187.2, 160.4, 141.5, 138.9, 134.8, 133.7, 129.7, 129.5 (q, J = 308.2 Hz), 125.3 (q, J = 2.2 Hz), 52.0, 28.5; **HRMS** (ESI-Orbitrap): calcd. for $[C_{13}H_{15}F_3NO_2S]^+$ (M + H$^+$): 306.0770, found: 306.0773.

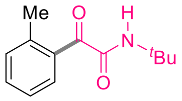

19

***N*-(*tert*-butyl)-2-oxo-2-(o-tolyl)acetamide (19).** Yellow solid, 21.9 mg, 50% yield (from 1-bromo-2-methylbenzene), 28.0 mg, 64% yield (from 1-iodo-2-methylbenzene), m.p. = 56.0 – 57.2 ºC. **^{1}H-NMR** ($CDCl_3$, 400 MHz): δ 7.85 (d, J = 7.9 Hz, 1H), 7.40 (t, J = 7.5 Hz, 1H),

7.25 (t, *J* = 7.8 Hz, 2H), 6.92 (br s, 1H), 2.46 (s, 3H), 1.44 (s, 9H); **^{13}C-NMR** ($CDCl_3$, 101 MHz): *δ* 192.4, 161.3, 139.8, 133.0, 132.5, 131.8, 131.6, 125.3, 51.7, 28.4, 20.8; **HRMS** (ESI-Orbitrap): calcd. for $[C_{13}H_{17}NNaO_2]^+$ (M + Na^+): 242.1151, found: 242.1152.

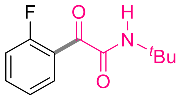

20

***N*-(*tert*-butyl)-2-(2-fluorophenyl)-2-oxoacetamide (20).** Yellow solid, 39.3 mg, 88% yield (from 1-bromo-2-fluorobenzene), 30.8 mg, 69% yield (from 1-fluoro-2-iodobenzene), m.p. = 63.6 – 64.8 ºC. **^{1}H-NMR** ($CDCl_3$, 400 MHz): *δ* 7.84 (t, *J* = 6.7 Hz, 1H), 7.53 (dd, *J* = 13.1, 6.2 Hz, 1H), 7.21 (t, *J* = 7.6 Hz, 1H), 7.11 (t, 1H), 6.74 (br s, 1H), 1.43 (s, 9H); **^{13}C-NMR** ($CDCl_3$, 101 MHz): *δ* 188.9, 161.8 (d, *J* = 257.8 Hz), 160.8, 135.1 (d, *J* = 9.0 Hz), 132.0 (d, *J* = 1.6 Hz), 124.1 (d, *J* = 3.7 Hz), 123.0 (d, *J* = 11.6 Hz), 116.5 (d, *J* = 21.6 Hz), 51.9, 28.4; **HRMS** (ESI-Orbitrap): calcd. for $[C_{12}H_{15}FNO_2]^+$ (M + H^+): 224.1081, found: 224.1085.

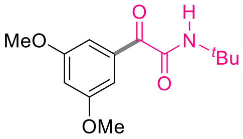

21

***N*-(*tert*-butyl)-2-(3,5-dimethoxyphenyl)-2-oxoacetamide (21).** Yellow solid, 45.1 mg, 85% yield (from 1-bromo-3,5-dimethoxybenzene); 42.4 mg, 80% yield (from 1-iodo-3,5-dimethoxybenzene), m.p. = 61.4 – 62.4 ºC. **^{1}H-NMR** ($CDCl_3$, 400 MHz): *δ* 7.42 (dd, J = 2.3, 1.5 Hz, 2H), 6.90 (br s, 1H), 6.65 (td, J = 2.3, 1.1 Hz, 1H), 3.79 (d, J = 0.9 Hz, 6H), 1.42 (s, 9H); **^{13}C-NMR** ($CDCl_3$, 101 MHz): *δ* 188.1, 161.3, 160.5, 134.9, 108.6, 107.3, 55.6, 51.7, 28.4; **HRMS** (ESI-Orbitrap): calcd. for $[C_{14}H_{20}NO_4]^+$ (M + H^+): 266.1387, found: 266.1385.

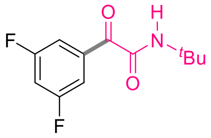

22

***N*-(*tert*-butyl)-2-(3,5-difluorophenyl)-2-oxoacetamide (22).** Yellow oil, 33.8 mg, 70% yield (from 1-bromo-3,5-difluorobenzene); 39.1 mg,81% yield (from 1,3-difluoro-5-iodobenzene). **^{1}H-NMR** ($CDCl_3$, 400 MHz): δ 7.93 – 7.83 (m, 2H), 7.04 (tt, J = 8.4, 2.4 Hz, 1H), 6.96 (br s, 1H), 1.44 (s, 9H); **^{13}C-NMR** ($CDCl_3$, 101 MHz): δ 185.9 (t, J = 2.6 Hz), 162.7 (d, J = 250.3 Hz), 62.6 (d, J = 250.3 Hz), 160.1, 135.9 (t, J = 8.6 Hz), 114.4 (d, J = 7.5 Hz), 114.2 (d, J = 7.4 Hz), 109.6 (t, J = 25.3 Hz), 52.0, 28.4; **HRMS** (ESI-Orbitrap): calcd. for $[C_{12}H_{14}FNO_2]^+$ (M + H^+): 242.0987, found: 242.0988.

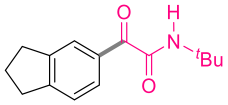

23

***N*-(*tert*-butyl)-2-(2,3-dihydro-1*H*-inden-5-yl)-2-oxoacetamide (23).** Yellow solid, 29.9 mg, 61% yield (from 5-bromo-2,3-dihydro-1*H*-indene), m.p. = 93.8 – 96.6 ºC. **^{1}H-NMR** ($CDCl_3$, 400 MHz): δ 8.17 (s, 1H), 8.09 (d, J = 8.0 Hz, 1H), 7.29 (d, J = 7.8 Hz, 1H), 6.94 (br s, 1H), 2.94 (t, J = 7.5 Hz, 4H), 2.10 (p, J = 7.5 Hz, 2H), 1.45 (s, 9H); **^{13}C-NMR** ($CDCl_3$, 101 MHz): δ 188.6, 161.7, 151.9, 144.7, 131.8, 129.9, 127.2, 124.4, 51.7, 33.3, 32.6, 28.5, 25.4; **HRMS** (ESI-Orbitrap): calcd. for $[C_{15}H_{20}NO_2]^+$ (M + H^+): 246.1489, found: 246.1489.

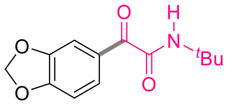

24

2-(benzo[*d*][1,3]dioxol-5-yl)-*N*-(*tert*-butyl)-2-oxoacetamide (24). Yellow solid, 38.9 mg, 78% yield {from 5-bromobenzo[d][1,3]dioxole}; 19.9 mg, 40% yield {from benzo[d][1,3]dioxol-5-yl trifluoromethanesulfonate}; 41.4 mg, 83% yield {from 5-iodobenzo[d][1,3]dioxole}, m.p. = 27.1 – 29.2 ºC. **^{1}H-NMR** ($CDCl_3$, 400 MHz): δ 8.10 (d, J = 8.3 Hz, 1H), 7.71 (s, 1H), 6.95 (br s, 1H), 6.82 (d, J = 8.3 Hz, 1H), 6.01 (s, 2H), 1.41 (s, 9H); **^{13}C-NMR** ($CDCl_3$, 101 MHz): δ 186.2, 161.6, 152.9, 147.9, 129.0, 127.9, 110.4, 108.0,

102.0, 51.6, 28.4; **HRMS** (ESI-Orbitrap): calcd. for $[C_{13}H_{16}NO_4]^+$ (M + H^+): 250.1074, found: 250.1071.

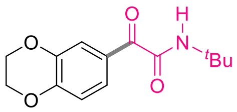

25

***N*-(*tert*-butyl)-2-(2,3-dihydrobenzo[*b*][1,4]dioxin-6-yl)-2-oxoacetamide (25).** Yellow oil, 36.8 mg, 70% yield {from 6-bromo-2,3-dihydrobenzo[b][1,4]dioxine}. **^{1}H-NMR** ($CDCl_3$, 400 MHz): δ 7.93 (dd, J = 8.5, 2.0 Hz, 1H), 7.89 (d, J = 1.9 Hz, 1H), 6.92 (br s, 1H), 6.88 (d, J = 8.5 Hz, 1H), 4.36 – 4.21 (m, 4H), 1.42 (s, 9H); **^{13}C-NMR** ($CDCl_3$, 101 MHz): δ 186.7, 161.6, 149.4, 143.3, 127.1, 126.1, 120.9, 117.3, 65.0, 64.1, 51.7, 28.5; **HRMS** (ESI-Orbitrap): calcd. for $[C_{14}H_{18}NO_4]^+$ (M + H^+): 264.1230, found: 264.1240.

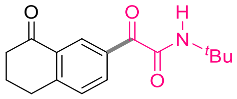

26

N-**(*tert*-butyl)-2-oxo-2-(8-oxo-5,6,7,8-tetrahydronaphthalen-2-yl)acetamide (26).** Yellow solid, 29.5 mg, 54% yield [from 7-bromo-3,4-dihydronaphthalen-1(2*H*)-one], m.p. = 39.8 – 41.7 °C. **^{1}H-NMR** ($CDCl_3$, 400 MHz): δ 8.88 (s, 1H), 8.42 (dd, J = 8.0, 1.7 Hz, 1H), 7.35 (d, J = 8.1 Hz, 1H), 6.95 (br s, 1H), 3.02 (t, J = 6.0 Hz, 2H), 2.69 (t, 2H), 2.21 – 2.11 (m, 2H), 1.45 (s, 9H); **^{13}C-NMR** ($CDCl_3$, 101 MHz): δ 197.3, 187.7, 160.7, 150.5, 135.4, 132.7, 132.3, 130.6, 129.2, 51.9, 39.1, 30.1, 28.5, 22.8; **HRMS** (ESI-Orbitrap): calcd. for $[C_{16}H_{20}NO_3]^+$ (M + H^+): 274.1438, found: 274.1438.

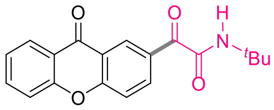

27

***N*-(*tert*-butyl)-2-oxo-2-(9-oxo-9*H*-xanthen-2-yl)acetamide (27).** Yellow solid, 50.4 mg, 78% yield (from 2-bromo-9*H*-xanthen-9-one), m.p. = 130.0 – 132.3 °C. **^{1}H-NMR** ($CDCl_3$, 400 MHz): δ 9.27 (d, J = 2.0 Hz, 1H), 8.67 (dd, J = 8.9, 2.2 Hz, 1H), 8.31 (dd, J = 8.0, 1.4

Hz, 1H), 7.73 (t, *J* = 7.0 Hz, 1H), 7.53 – 7.46 (m, 2H), 7.40 (t, *J* = 7.1 Hz, 1H), 7.04 (br s, 1H), 1.47 (s, 9H); **^{13}C-NMR** ($CDCl_3$, 101 MHz): δ 186.6, 176.4, 160.8, 159.4, 155.9, 136.9, 135.4, 131.9, 129.3, 127.0, 124.8, 121.9, 121.5, 118.4, 118.2, 51.9, 28.5; **HRMS** (ESI-Orbitrap): calcd. for $[C_{19}H_{18}NO_4]^+$ (M + H^+): 324.1230, found: 324.1230.

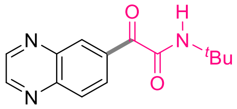

28

***N*-(*tert*-butyl)-2-oxo-2-(quinoxalin-6-yl)acetamide (28).** Yellow solid, 26.7 mg, 52% yield (from 6-bromoquinoxaline), m.p. = 87.4 – 89.2 ºC. **^{1}H-NMR** ($CDCl_3$, 400 MHz): δ 9.20 (d, *J* = 1.5 Hz, 1H), 8.92 (d, *J* = 4.6 Hz, 2H), 8.47 (dd, *J* = 8.8, 1.8 Hz, 1H), 8.14 (d, *J* = 8.8 Hz, 1H), 7.00 (br s, 1H), 1.48 (s, 9H); **^{13}C-NMR** ($CDCl_3$, 101 MHz): δ 188.0, 160.6, 147.1, 146.2, 145.5, 142.3, 135.2, 134.2, 130.1, 129.9, 52.0, 28.5; **HRMS** (ESI-Orbitrap): calcd. for $[C_{14}H_{16}N_3O_2]^+$ (M + H^+): 258.1237, found: 258.1237.

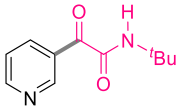

29

***N*-(*tert*-butyl)-2-oxo-2-(pyridin-3-yl)acetamide (29).** Yellow oil, 30.1 mg, 73% yield (from 3-bromopyridine); 39.2 mg, 95% yield (from 3-iodopyridine). **^{1}H-NMR** ($CDCl_3$, 400 MHz): δ 9.38 (s, 1H), 8.72 (d, *J* = 4.7 Hz, 1H), 8.54 (d, *J* = 8.0 Hz, 1H), 7.34 (dd, *J* = 8.0, 4.9 Hz, 1H), 7.03 (br s, 1H), 1.39 (s, 9H); **^{13}C-NMR** ($CDCl_3$, 101 MHz): δ 187.5, 160.2, 154.0, 152.1, 138.5, 129.2, 123.2, 51.8, 28.3; **HRMS** (ESI-Orbitrap): calcd. for $[C_{11}H_{15}N_2O_2]^+$ (M + H^+): 207.1128, found: 207.1127.

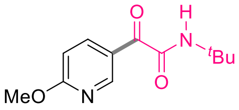

30

***N*-(*tert*-butyl)-2-(6-methoxypyridin-3-yl)-2-oxoacetamide (30).** Yellow oil, 34.0 mg, 72% yield (from 5-bromo-2-methoxypyridine); 46.3 mg, 98% yield (from 5-iodo-2-

methoxypyridine). ^{1}**H-NMR** ($CDCl_3$, 400 MHz): δ 9.26 (s, 1H), 8.47 (d, J = 8.8 Hz, 1H), 7.00 (br s, 1H), 6.75 (d, J = 8.8 Hz, 1H), 3.99 (s, 3H), 1.42 (s, 9H); ^{13}**C-NMR** ($CDCl_3$, 101 MHz): δ 186.2, 167.2, 160.9, 153.0, 140.9, 123.6, 110.9, 54.3 51.7, 28.4; **HRMS** (ESI-Orbitrap): calcd. for $[C_{12}H_{17}N_2O_3]^+$ (M + H$^+$): 237.1234, found: 237.1234.

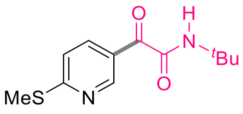

31

***N*-(*tert*-butyl)-2-(6-(methylthio)pyridin-3-yl)-2-oxoacetamide (31).** Yellow oil, 23.2 mg, 46% yield [from 5-bromo-2-(methylthio)pyridine]. ^{1}**H-NMR** ($CDCl_3$, 400 MHz): δ 9.37 (d, J = 1.4 Hz, 1H), 8.40 (dd, J = 8.6, 2.1 Hz, 1H), 7.23 (d, J = 8.6 Hz, 1H), 7.00 (br s, 1H), 2.60 (s, 3H), 1.44 (s, 9H); ^{13}**C-NMR** ($CDCl_3$, 101 MHz): δ 186.7, 167.3, 160.6, 152.7, 137.5, 125.0, 120.8, 51.8, 28.4, 13.4; **HRMS** (ESI-Orbitrap): calcd. for $[C_{12}H_{17}N_2O_2S]^+$ (M + H$^+$): 253.1005, found: 253.1004.

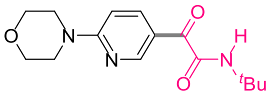

32

***N*-(*tert*-butyl)-2-(6-morpholinopyridin-3-yl)-2-oxoacetamide (32).** Yellow solid, 46.0 mg, 79% yield [from 4-(5-bromopyridin-2-yl)morpholine]; 47.2 mg, 81% yield [from 4-(5-iodopyridin-2-yl)morpholine], m.p. = 90.9 – 92.2 ºC. ^{1}**H-NMR** ($CDCl_3$, 400 MHz): δ 9.22 (d, J = 2.3 Hz, 1H), 8.41 (dd, J = 9.2, 2.3 Hz, 1H), 7.03 (br s, 1H), 6.53 (d, J = 9.2 Hz, 1H), 3.78 – 3.73 (m, 4H), 3.70 – 3.67 (m, 4H), 1.40 (s, 9H); ^{13}**C-NMR** ($CDCl_3$, 101 MHz): δ 185.0, 161.7, 160.4 154.2, 140.1, 119.4, 104.9, 66.6, 51.4, 44.8, 28.4; **HRMS** (ESI-Orbitrap): calcd. for $[C_{15}H_{22}N_3O_3]^+$ (M + H$^+$): 292.1656, found: 292.1655.

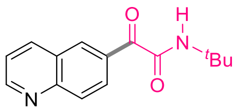

33

***N*-(*tert*-butyl)-2-oxo-2-(quinolin-6-yl)acetamide (33).** Yellow solid, 46.1 mg, 90% yield (from 6-bromoquinoline); 16.9 mg, 33% yield (from quinolin-6-yl trifluoromethanesulfonate); 48.2 mg, 94% yield (from 6-iodoquinoline), m.p. = 106.6 – 108.5 ºC. **^{1}H-NMR** ($CDCl_3$, 400 MHz): δ 9.07 (d, J = 1.8 Hz, 1H), 8.92 (dd, J = 4.2, 1.7 Hz, 1H), 8.30 (dd, J = 8.9, 1.9 Hz, 1H), 8.20 (dd, J = 8.3, 1.3 Hz, 1H), 8.04 (d, J = 8.9 Hz, 1H), 7.38 (dd, J = 8.3, 4.3 Hz, 1H), 7.09 (br s, 1H), 1.42 (s, 9H); **^{13}C-NMR** ($CDCl_3$, 101 MHz): δ 187.4, 161.0, 153.1, 150.3, 138.1, 134.6, 131.0, 129.8, 129.2, 127.2, 121.9, 51.8, 28.4; **HRMS** (ESI-Orbitrap): calcd. for $[C_{15}H_{17}N_2O_2]^+$ (M + H$^+$): 257.1285, found: 257.1287.

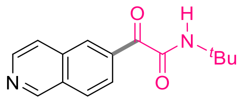

34

***N*-(*tert*-butyl)-2-(isoquinolin-6-yl)-2-oxoacetamide (34).** Yellow solid, 33.8 mg, 66% yield (from 6-bromoisoquinoline); 16.4 mg, 32% yield (from isoquinolin-6-yl trifluoromethanesulfonate), m.p. = 67.1 – 69.1 ºC. **^{1}H-NMR** ($CDCl_3$, 400 MHz): δ 9.31 (s, 1H), 9.08 (s, 1H), 8.61 (d, J = 5.6 Hz, 1H), 8.25 (d, J = 8.6 Hz, 1H), 8.01 (d, J = 8.6 Hz, 1H), 7.79 (d, J = 5.6 Hz, 1H), 7.07 (br s, 1H), 1.48 (s, 9H); **^{13}C-NMR** ($CDCl_3$, 101 MHz): δ 187.9, 160.6, 152.5, 144.0, 135.0, 134.4, 132.8, 130.2 127.9, 127.1, 122.1, 52.0, 28.5; **HRMS** (ESI-Orbitrap): calcd. for $[C_{15}H_{17}N_2O_2]^+$ (M + H$^+$): 257.1285, found: 257.1284.

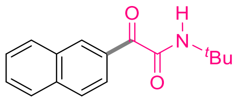

35

***N*-(*tert*-butyl)-2-(naphthalen-2-yl)-2-oxoacetamide (35).** Yellow solid, 38.8 mg, 76% yield (from 2-bromonaphthalene); 13.3 mg, 26% yield (from naphthalen-2-yl trifluoromethanesulfonate); 43.9 mg, 86% yield (from 2-iodonaphthalene), m.p. = 75.8 – 77.5 ºC. **^{1}H-NMR** ($CDCl_3$, 400 MHz): δ 9.16 (s, 1H), 8.16 (dd, J = 8.7, 1.6 Hz, 1H), 7.98 (d,

J = 8.1 Hz, 1H), 7.84 (t, J = 8.5 Hz, 2H), 7.62 – 7.56 (m, 1H), 7.55 – 7.49 (m, 1H), 7.09 (br s, 1H), 1.49 (s, 9H); **^{13}C-NMR** ($CDCl_3$, 101 MHz): δ 188.1, 161.4, 136.0, 134.9, 132.4, 130.6, 130.3, 129.2, 128.3, 127.8, 126.8, 125.5, 51.8, 28.5; **HRMS** (ESI-Orbitrap): calcd. for $[C_{16}H_{18}NO_2]^+$ (M + H$^+$): 256.1332, found: 256.1333.

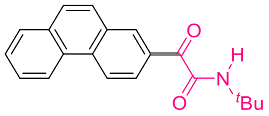

36

***N*-(*tert*-butyl)-2-oxo-2-(phenanthren-2-yl)acetamide (36).** Yellow solid, 45.2 mg, 74% yield (from 2-bromophenanthrene), m.p. = 138.3 – 139.7 ºC. **^{1}H-NMR** ($CDCl_3$, 400 MHz): δ 9.08 (d, J = 1.6 Hz, 1H), 8.69 – 8.60 (m, 2H), 8.40 (dd, J = 8.8, 1.7 Hz, 1H), 7.90 – 7.85 (m, 1H), 7.78 (dd, J = 25.2, 8.9 Hz, 2H), 7.69 – 7.61 (m, 2H), 7.13 (br s, 1H), 1.52 (s, 9H); **^{13}C-NMR** ($CDCl_3$, 101 MHz): δ 187.9, 161.4, 134.1, 133.9, 133.3, 131.2, 131.1, 129.5, 128.7, 128.1, 127.8, 127.7, 127.2, 127.0, 123.5, 123.0, 51.8, 28.5; **HRMS** (ESI-Orbitrap): calcd. for $[C_{20}H_{20}NO_2]^+$ (M + H$^+$): 306.1489, found: 306.1487.

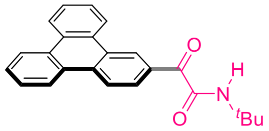

37

***N*-(*tert*-butyl)-2-oxo-2-(triphenylen-2-yl)acetamide (37).** Yellow solid, 52.6 mg, 74% yield (from 2-bromotriphenylene), m.p. = 159.7 – 160.5 ºC. **^{1}H-NMR** ($CDCl_3$, 400 MHz): δ 9.72 (s, 1H), 8.66 – 8.58 (m, 1H), 8.52 (t, J = 8.3 Hz, 4H), 8.44 – 8.37 (m, 1H), 7.69 – 7.54 (m, 4H), 7.18 (br s, 1H), 1.56 (s, 9H); **^{13}C-NMR** ($CDCl_3$, 101 MHz): δ 187.7, 161.5, 134.0, 131.5, 131.0, 129.7, 129.5, 129.3, 128.7, 128.6, 128.4, 127.9, 127.8, 127.6, 127.4, 124.2, 123.7, 123.4, 123.3, 123.2, 51.8, 28.6; **HRMS** (ESI-Orbitrap): calcd. for $[C_{24}H_{22}NO_2]^+$ (M + H$^+$): 356.1645, found: 356.1645.

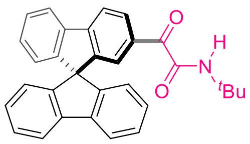

38

2-(9,9'-spirobi[fluoren]-2-yl)-*N*-(*tert*-butyl)-2-oxoacetamide (38). Yellow solid, 70.9 mg, 80% yield {from 2-bromo-9,9'-spirobi[fluorene]}, m.p. = 207.4 – 210.1 °C. **^{1}H-NMR** ($CDCl_3$, 400 MHz): δ 8.59 (dd, J = 8.1, 1.5 Hz, 1H), 7.95 (dd, J = 7.8, 2.5 Hz, 2H), 7.88 (d, J = 7.6 Hz, 2H), 7.63 (d, J = 0.9 Hz, 1H), 7.40 (q, J = 7.6 Hz, 3H), 7.19 (t, J = 7.5 Hz, 1H), 7.12 (t, J = 7.5 Hz, 2H), 6.88 (br s, 1H), 6.78 (d, J = 7.6 Hz, 1H), 6.73 (d, J = 7.6 Hz, 2H), 1.41 (s, 9H); **^{13}C-NMR** ($CDCl_3$, 101 MHz): δ 187.8, 161.4, 150.5, 149.0, 147.8, 147.7, 141.9, 140.2, 132.8, 132.6, 129.6, 128.1, 128.1, 128.0, 126.4, 124.3, 124.0, 121.3, 120.3, 119.7, 65.9, 51.6, 28.4; **HRMS** (ESI-Orbitrap): calcd. for $[C_{31}H_{26}NO_2]^+$ (M + H$^+$): 444.1958, found: 444.1959.

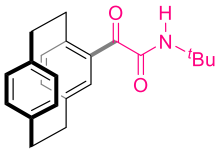

39

2-(1,4(1,4)-dibenzenacyclohexaphane-1^2-yl)-*N*-(*tert*-butyl)-2-oxoacetamide (39). Yellow solid, 55.6 mg, 83% yield [from 12-bromo-1,4(1,4)-dibenzenacyclohexaphane], m.p. = 70.4 – 72.1 °C. **^{1}H-NMR** ($CDCl_3$, 400 MHz): δ 6.84 (d, J = 7.9 Hz, 1H), 6.65 (s, 1H), 6.58 – 6.51 (m, 3H), 6.49 – 6.41 (m, 2H), 5.40 (br s, 1H), 3.76 – 3.47 (m, 1H), 3.23 – 2.82 (m, 7H), 1.47 (s, 9H); **^{13}C-NMR** ($CDCl_3$, 101 MHz): δ 168.7, 140.2, 139.6, 139.3, 138.2, 136.6, 135.8, 134.6, 132.6 (d, J = 1.8 Hz), 132.5, 132.1, 131.9, 51.5, 35.5, 35.4, 35.2, 34.9, 29.0; **HRMS** (ESI-Orbitrap): calcd. for $[C_{22}H_{26}NO_2]^+$ (M + H$^+$): 336.1958, found: 336.1960.

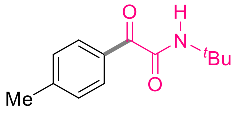

40

***N*-(*tert*-butyl)-2-oxo-2-(*p*-tolyl)acetamide (40).** White solid, 18.8 mg, 43% yield (from 1-iodo-4-methylbenzene); 36.4 mg, 83% yield (from p-tolyl trifluoromethanesulfonate), m.p.

= 48.4 – 49.5 ºC. **^{1}H-NMR** ($CDCl_3$, 400 MHz): *δ* 8.21 (d, *J* = 8.2 Hz, 2H), 7.24 (d, *J* = 8.1 Hz, 2H), 6.95 (br s, 1H), 2.40 (s, 3H), 1.44 (s, 9H); **^{13}C-NMR** ($CDCl_3$, 101 MHz): *δ* 188.1, 161.5, 145.4, 131.6, 130.9, 129.2, 51.7, 28.5, 22.0; **HRMS** (ESI-Orbitrap): calcd. for $[C_{13}H_{18}NO_2]^+$ (M + H^+): 220.1332, found: 220.1330.

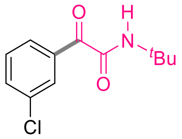

41

***N*-(*tert*-butyl)-2-(3-chlorophenyl)-2-oxoacetamide (41).** Yellow oil, 12.0 mg, 25% yield (from 3-chlorophenyl trifluoromethanesulfonate). **^{1}H-NMR** ($CDCl_3$, 400 MHz): *δ* 8.31 (s, 1H), 8.22 (d, *J* = 7.8 Hz, 1H), 7.60 – 7.54 (m, 1H), 7.41 (t, *J* = 7.9 Hz, 1H), 6.95 (br s, 1H), 1.45 (s, 9H); **^{13}C-NMR** ($CDCl_3$, 101 MHz): *δ* 187.2, 160.5, 134.9, 134.7, 134.2, 131.2, 129.8, 129.5, 51.9, 28.4; **HRMS** (ESI-Orbitrap): calcd. for $[C_{12}H_{15}ClNO_2]^+$ (M + H^+): 240.0786, found: 240.0788.

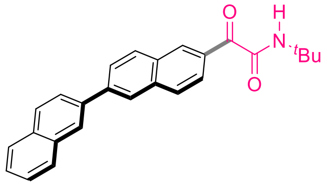

42

2-([2,2'-binaphthalen]-6-yl)-*N*-(*tert*-butyl)-2-oxoacetamide (42). Yellow solid, 19.1 mg, 25% yield {from [2,2'-binaphthalen]-6-yl trifluoromethanesulfonate}, m.p. = 143.2 – 145.7 ºC. **^{1}H-NMR** ($CDCl_3$, 400 MHz): *δ* 9.24 (s, 1H), 8.27 – 8.15 (m, 3H), 8.11 (d, *J* = 8.5 Hz, 1H), 8.01 – 7.81 (m, 6H), 7.60 – 7.47 (m, 2H), 7.10 (br s, 1H), 1.51 (s, 9H); **^{13}C-NMR** ($CDCl_3$, 101 MHz): *δ* 187.9, 161.4, 141.8, 137.8, 136.5, 134.8, 133.7, 133.0, 131.6, 131.1, 130.7, 128.8, 128.6, 128.5, 127.8, 126.7, 126.6, 126.5, 126.1, 125.9, 125.6, 51.8, 28.6; **HRMS** (ESI-Orbitrap): calcd. for $[C_{26}H_{24}NO_2]^+$ (M + H^+): 382.1802, found: 382.1804.

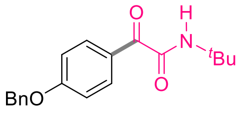

43

2-(4-(benzyloxy)phenyl)-*N*-(*tert*-butyl)-2-oxoacetamide (43). Yellow solid, 54.8 mg, 88% yield [from 1-(benzyloxy)-4-iodobenzene], m.p. = 93.6 – 95.3 ºC. **^{1}H-NMR** ($CDCl_3$, 400 MHz): δ 8.38 (d, J = 8.9 Hz, 2H), 7.46 – 7.33 (m, 5H), 7.05 – 6.94 (m, 3H), 5.12 (s, 2H), 1.45 (s, 9H); **^{13}C-NMR** ($CDCl_3$, 101 MHz): δ 186.6, 163.7, 161.7, 136.0, 134.0, 128.8, 128.4, 127.6, 126.6, 114.6, 70.2, 51.6, 28.5; **HRMS** (ESI-Orbitrap): calcd. for $[C_{19}H_{22}NO_3]^+$ (M + H^+): 312.1594, found: 312.1598.

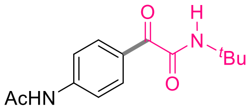

44

2-(4-acetamidophenyl)-*N*-(*tert*-butyl)-2-oxoacetamide (44). Yellow solid, 30.4 mg, 58% yield [from *N*-(4-iodophenyl)acetamide], m.p. = 161.2 – 163.1 ºC. **^{1}H-NMR** ($CDCl_3$, 400 MHz): δ 8.53 (s, 1H), 8.14 (d, J = 8.7 Hz, 2H), 7.52 (d, J = 8.7 Hz, 2H), 7.00 (br s, 1H), 2.13 (s, 3H), 1.44 (s, 9H); **^{13}C-NMR** ($CDCl_3$, 101 MHz): δ 187.3, 169.5, 162.4, 143.8, 132.5, 128.5, 119.0, 51.9, 28.5, 24.7; **HRMS** (ESI-Orbitrap): calcd. for $[C_{14}H_{19}N_2O_3]^+$ (M + H^+): 263.1390, found: 263.1391.

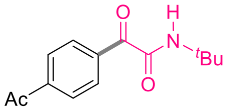

45

2-(4-acetylphenyl)-*N*-(*tert*-butyl)-2-oxoacetamide (45). Yellow solid, 35.1 mg, 71% yield [from 1-(4-iodophenyl)ethan-1-one], m.p. = 67.2 – 69.3 ºC. **^{1}H-NMR** ($CDCl_3$, 400 MHz): δ 8.33 (d, J = 8.4 Hz, 2H), 7.97 (d, J = 8.4 Hz, 2H), 6.97 (br s, 1H), 2.61 (s, 3H), 1.43 (s, 9H); **^{13}C-NMR** ($CDCl_3$, 101 MHz): δ 197.6, 188.0, 160.6, 140.6, 136.7, 131.4, 128.1, 51.9, 28.4, 27.0; **HRMS** (ESI-Orbitrap): calcd. for $[C_{14}H_{18}NO_3]^+$ (M + H^+): 248.1281, found: 248.1282.

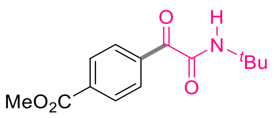

46

Methyl 4-(2-(*tert*-butylamino)-2-oxoacetyl)benzoate (46). White solid, 35.3 mg, 67% yield (from methyl 4-iodobenzoate), m.p. = 115.9 – 117.1 ºC. **^{1}H-NMR** ($CDCl_3$, 400 MHz): δ 8.32 (d, J = 8.5 Hz, 2H), 8.07 (d, J = 8.5 Hz, 2H), 6.97 (br s, 1H), 3.92 (s, 3H), 1.43 (s, 9H); **^{13}C-NMR** ($CDCl_3$, 101 MHz): δ 188.1, 166.2, 160.6, 136.8, 134.6, 131.2, 129.5, 52.6, 51.9, 28.4; **HRMS** (ESI-Orbitrap): calcd. for $[C_{14}H_{18}NO_4]^+$ (M + H^+): 264.1230, found: 264.1233.

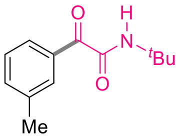

47

***N*-(*tert*-butyl)-2-oxo-2-(*m*-tolyl)acetamide (47).** White solid, 33.7 mg, 77% yield (from 1-iodo-3-methylbenzene), m.p. = 58.5 – 60.2 ºC. **^{1}H-NMR** ($CDCl_3$, 400 MHz): δ 8.07 (d, J = 8.0 Hz, 2H), 7.39 (d, J = 7.3 Hz, 1H), 7.33 (t, J = 7.7 Hz, 1H), 6.94 (br s, 1H), 2.39 (s, 3H), 1.44 (s, 9H); **^{13}C-NMR** ($CDCl_3$, 101 MHz): δ 188.8, 161.3, 138.2, 135.1, 133.4, 131.6, 128.5, 128.3, 51.7, 28.4, 21.4; **HRMS** (ESI-Orbitrap): calcd. for $[C_{13}H_{17}NNaO_2]^+$ (M + Na^+): 242.1151, found: 242.1155.

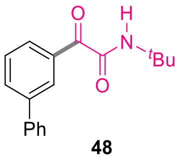

48

2-([1,1'-biphenyl]-3-yl)-*N*-(*tert*-butyl)-2-oxoacetamide (48). Yellow oil, 36.5 mg, 65% yield (from 3-iodo-1,1'-biphenyl). **^{1}H-NMR** ($CDCl_3$, 400 MHz): δ 8.54 (s, 1H), 8.30 (d, J = 7.8 Hz, 1H), 7.83 (d, J = 7.9 Hz, 1H), 7.63 (d, J = 7.3 Hz, 2H), 7.53 (t, J = 7.8 Hz, 1H), 7.46 (t, J = 7.5 Hz, 2H), 7.38 (t, J = 7.3 Hz, 1H), 7.02 (br s, 1H), 1.48 (s, 9H); **^{13}C-NMR** ($CDCl_3$, 101 MHz): δ 188.6, 161.2, 141.5, 140.0, 133.9, 132.8, 130.1, 129.8, 129.0, 128.9, 127.8, 127.3, 51.8, 28.4; **HRMS** (ESI-Orbitrap): calcd. for $[C_{18}H_{19}NNaO_2]^+$ (M + Na^+): 304.1308, found: 304.1304.

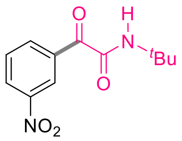

49

***N*-(*tert*-butyl)-2-(3-nitrophenyl)-2-oxoacetamide (49).** Yellow solid, 37.5 mg, 75% yield (from 1-iodo-3-nitrobenzene), m.p. = 27.3 – 29.1 ºC. **^{1}H-NMR** ($CDCl_3$, 400 MHz): δ 9.12 (s, 1H), 8.66 (d, J = 7.8 Hz, 1H), 8.42 (ddd, J = 8.2, 2.1, 0.9 Hz, 1H), 7.66 (t, J = 8.0 Hz, 1H), 7.02 (br s, 1H), 1.45 (s, 9H); **^{13}C-NMR** ($CDCl_3$, 101 MHz): δ 186.2, 159.9, 148.2, 137.0, 134.7, 129.7, 128.2, 126.2, 52.1, 28.4; **HRMS** (ESI-Orbitrap): calcd. for $[C_{12}H_{14}N_2NaO_4]^+$ (M + Na^+): 273.0846, found: 273.0851.

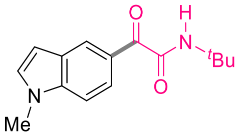

50

***N*-(*tert*-butyl)-2-(1-methyl-1*H*-indol-5-yl)-2-oxoacetamide (50).** Yellow solid, 28.4 mg, 55% yield (from 5-iodo-1-methyl-1*H*-indole), m.p. = 64.0 – 66.5 ºC. **^{1}H-NMR** ($CDCl_3$, 400 MHz): δ 8.89 (d, J = 1.5 Hz, 1H), 8.13 (dd, J = 8.8, 1.6 Hz, 1H), 7.31 (d, J = 8.8 Hz, 1H), 7.08 (d, J = 3.2 Hz, 1H), 7.01 (br s, 1H), 6.61 (d, J = 3.1 Hz, 1H), 3.78 (s, 3H), 1.47 (s, 9H); **^{13}C-NMR** ($CDCl_3$, 101 MHz): δ 188.2, 162.6, 139.8, 130.6, 128.0, 127.5, 125.2, 124.3, 109.3, 103.9, 51.6, 33.1, 28.5; **HRMS** (ESI-Orbitrap): calcd. for $[C_{15}H_{19}N_2O_2]^+$ (M + H^+): 259.1441, found: 259.1446.

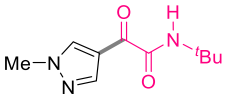

51

***N*-(*tert*-butyl)-2-(1-methyl-1*H*-pyrazol-4-yl)-2-oxoacetamide (51).** Yellow solid, 33.9 mg, 81% yield (from 4-iodo-1-methyl-1*H*-pyrazole), m.p. = 66.2 – 67.9 ºC. **^{1}H-NMR** ($CDCl_3$, 400 MHz): δ 8.60 (s, 1H), 8.16 (s, 1H), 7.14 (br s, 1H), 3.90 (s, 3H), 1.39 (s, 9H); **^{13}C-NMR** ($CDCl_3$, 101 MHz): δ 181.1, 160.5, 143.1, 137.1, 118.6, 51.3, 39.3, 28.4; **HRMS** (ESI-Orbitrap): calcd. for $[C_{10}H_{16}N_3O_2]^+$ (M + H^+): 210.1237, found: 210.1237.

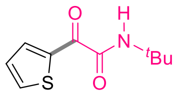

52

***N*-(*tert*-butyl)-2-oxo-2-(thiophen-2-yl)acetamide (52).** Yellow solid, 25.8 mg, 61% yield (from 2-iodothiophene), m.p. = 31.3 – 33.7 ºC. **^{1}H-NMR** ($CDCl_3$, 400 MHz): δ 8.34 (dd, J = 3.8, 0.7 Hz, 1H), 7.81 (dd, J = 4.9, 1.0 Hz, 1H), 7.19 (br s, 1H), 7.16 (t, J = 4.4 Hz, 1H), 1.44 (s, 9H); **^{13}C-NMR** ($CDCl_3$, 101 MHz): δ 179.3, 160.1, 138.8, 138.0, 136.3, 128.1, 51.7, 28.5; **HRMS** (ESI-Orbitrap): calcd. for $[C_{10}H_{14}NO_2S]^+$ (M + H^+): 212.0740, found: 212.0741.

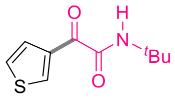

53

***N*-(*tert*-butyl)-2-oxo-2-(thiophen-3-yl)acetamide (53).** Yellow solid, 35.9 mg, 85% yield (from 3-iodothiophene), m.p. = 36.8 – 37.6 ºC. **^{1}H-NMR** ($CDCl_3$, 400 MHz): δ 9.08 (dd, J = 2.9, 1.1 Hz, 1H), 7.73 (dd, J = 5.1, 1.1 Hz, 1H), 7.26 (dd, J = 5.2, 2.9 Hz, 1H), 7.13 (br s, 1H), 1.41 (s, 9H); **^{13}C-NMR** ($CDCl_3$, 101 MHz): δ 181.0, 160.6, 139.4, 137.0, 128.9, 125.6, 51.5, 28.4; **HRMS** (ESI-Orbitrap): calcd. for $[C_{10}H_{14}NO_2S]^+$ (M + H^+): 212.0740, found: 212.0742.

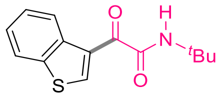

54

2-(benzo[*b*]thiophen-3-yl)-*N*-(*tert*-butyl)-2-oxoacetamide (54). Yellow solid, 50.6 mg, 97% yield {from 3-iodobenzo[b]thiophene}, m.p. = 35.3 – 36.2 ºC. **^{1}H-NMR** ($CDCl_3$, 400 MHz): δ 9.72 (s, 1H), 8.70 (d, J = 8.2 Hz, 1H), 7.87 (d, J = 8.0 Hz, 1H), 7.50 (td, J = 7.64 Hz, 1.1 Hz, 1H), 7.41 (td, J = 8.1, 1.2 Hz, 1H), 7.30 (br s, 1H), 1.47 (s, 9H); **^{13}C-NMR** ($CDCl_3$, 101 MHz): δ 181.2, 161.0, 145.7, 138.9, 137.6, 129.9, 126.1, 125.6, 125.2, 122.4, 51.5, 28.4; **HRMS** (ESI-Orbitrap): calcd. for $[C_{14}H_{16}NO_2S]^+$ (M + H^+): 262.0896, found: 262.0897.

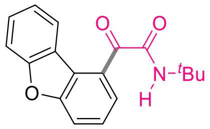

55

***N*-(*tert*-butyl)-2-(dibenzo[*b,d*]furan-1-yl)-2-oxoacetamide (55).** White solid, 42.5 mg, 72% yield {from 1-iododibenzo[b,d]furan}, m.p. = 117.4 – 119.3 ºC. **^{1}H-NMR** ($CDCl_3$, 400 MHz): δ 8.46 (d, J = 8.0 Hz, 1H), 8.24 (d, J = 7.7 Hz, 1H), 7.77 (d, J = 8.2 Hz, 1H), 7.57 (d, J = 8.2 Hz, 1H), 7.53 – 7.48 (m, 2H), 7.33 (t, J = 8.1 Hz, 1H), 7.12 (br s, 1H), 1.51 (s, 9H); **^{13}C-NMR** ($CDCl_3$, 101 MHz): δ 189.8, 161.5, 157.0, 156.6, 128.6, 128.5, 126.0, 125.5, 124.2, 122.8, 117.2, 111.5, 51.9, 28.5; **HRMS** (ESI-Orbitrap): calcd. for $[C_{18}H_{18}NO_3]^+$ (M + H$^+$): 296.1281, found: 296.1281.

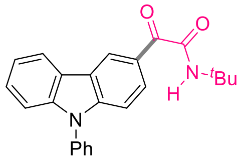

56

***N*-(*tert*-butyl)-2-oxo-2-(9-phenyl-9*H*-carbazol-3-yl)acetamide (56).** Yellow solid, 62.9 mg, 85% yield (from 3-iodo-9-phenyl-9*H*-carbazole), m.p. = 194.9 – 196.7 ºC. **^{1}H-NMR** ($CDCl_3$, 400 MHz): δ 9.38 (s, 1H), 8.41 (d, J = 8.8 Hz, 1H), 8.24 (d, J = 7.7 Hz, 1H), 7.62 (d, J = 7.7 Hz, 2H), 7.57 – 7.51 (m, 3H), 7.48 – 7.32 (m, 5H), 7.13 (br s, 1H), 1.51 (s, 9H); **^{13}C-NMR** ($CDCl_3$, 101 MHz): δ 187.2, 162.2, 144.3, 141.8, 136.8, 130.2, 129.6, 128.3, 127.2, 126.9, 125.9, 125.7, 123.7, 123.2, 121.3, 121.0, 110.4, 109.7, 51.7, 28.6; **HRMS** (ESI-Orbitrap): calcd. for $[C_{24}H_{23}N_2O_2]^+$ (M + H$^+$): 371.1754, found: 371.1755.

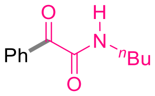

57

***N*-butyl-2-oxo-2-phenylacetamide (57).** Yellow oil, 32.8 mg, 80% yield (from 1-isocyanobutane). **^{1}H-NMR** ($CDCl_3$, 400 MHz): δ 8.38 – 8.27 (m, 2H), 7.61 (t, J = 7.4 Hz, 1H), 7.47 (t, J = 7.8 Hz, 2H), 7.11 (br s, 1H), 3.39 (dd, J = 13.6, 6.8 Hz, 2H), 1.67 – 1.51 (m, 2H), 1.47 – 1.33 (m, 2H), 0.95 (t, J = 7.3 Hz, 3H); **^{13}C-NMR** ($CDCl_3$, 101 MHz): δ 188.0,

161.9, 134.5, 133.5, 131.3, 128.6, 39.3, 31.4, 20.2, 13.8; **HRMS** (ESI-Orbitrap): calcd. for $[C_{12}H_{16}NO_2]^+$ (M + H$^+$): 206.1176, found: 206.1180.

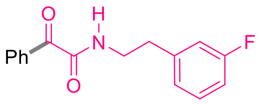

58

***N*-(2-fluorophenethyl)-2-oxo-2-phenylacetamide (58).** Yellow oil, 40.7 mg, 75% yield [from 1-fluoro-3-(2-isocyanoethyl)benzene]. **^{1}H-NMR** ($CDCl_3$, 400 MHz): *δ* 8.29 (d, *J* = 7.8 Hz, 2H), 7.62 (t, *J* = 7.4 Hz, 1H), 7.47 (t, *J* = 7.7 Hz, 2H), 7.31 – 7.24 (m, 1H), 7.21 (br s, 1H), 7.00 (d, *J* = 7.6 Hz, 1H), 6.97 – 6.90 (m, 2H), 3.65 (q, *J* = 6.9 Hz, 2H), 2.90 (t, *J* = 7.1 Hz, 2H); **^{13}C-NMR** ($CDCl_3$, 101 MHz): *δ* 187.7, 163.0 (d, *J* = 246.2 Hz), 161.9, 140.9 (d, *J* = 7.4 Hz), 134.6, 133.3, 131.2, 130.3 (d, *J* = 8.4 Hz), 128.6, 124.5 (d, *J* = 2.8 Hz), 115.7 (d, *J* = 21.1 Hz), 113.7 (d, *J* = 21.0 Hz), 40.4, 35.3; **HRMS** (ESI-Orbitrap): calcd. for $[C_{16}H_{15}FNO_2]^+$ (M + H$^+$): 272.1081, found: 272.1090.

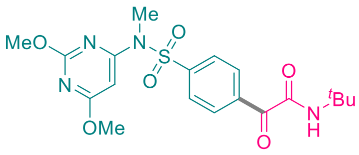

59

***N*-(*tert*-butyl)-2-(4-(*N*-(2,6-dimethoxypyrimidin-4-yl)-*N*-methylsulfamoyl)phenyl)-2-oxoacetamide (59).** Yellow solid, 56.7 mg, 65% yield (from aryl bromide derivative of Sulfadimethoxine); 75.9 mg, 87% yield (from aryl iodide derivative of Sulfadimethoxine), m.p. = 39.4 – 41.4 ºC. **^{1}H-NMR** ($CDCl_3$, 400 MHz): *δ* 8.36 (d, *J* = 8.3 Hz, 2H), 7.84 (d, *J* = 8.3 Hz, 2H), 6.95 (br s, 1H), 6.55 (s, 1H), 3.89 (s, 3H), 3.80 (s, 3H), 3.43 (s, 3H), 1.41 (s, 9H); **^{13}C-NMR** ($CDCl_3$, 101 MHz): *δ* 187.1, 172.6, 164.3, 161.0, 160.0, 142.9, 137.0, 131.9, 127.0, 90.0, 54.9, 54.2, 52.0, 34.7, 28.3; **HRMS** (ESI-Orbitrap): calcd. for $[C_{19}H_{25}N_4O_6S]^+$ (M + H$^+$): 437.1489, found: 437.1490.

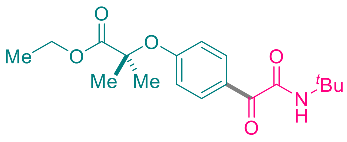

60

Ethyl 2-(4-(2-(*tert*-butylamino)-2-oxoacetyl)phenoxy)-2-methylpropanoate (60). Yellow solid, 50.9 mg, 76% yield (from aryl iodide derivative of Clofibrate), m.p. = 96.9 – 98.6 °C. **^{1}H-NMR** ($CDCl_3$, 400 MHz): δ 8.28 (d, J = 9.1 Hz, 2H), 6.95 (br s, 1H), 6.79 (d, J = 9.1 Hz, 2H), 4.19 (q, J = 7.1 Hz, 2H), 1.63 (s, 6H), 1.41 (s, 9H), 1.18 (t, J = 7.1 Hz, 3H); **^{13}C-NMR** ($CDCl_3$, 101 MHz): δ 186.7, 173.6, 161.6, 160.9, 133.5, 126.8, 117.0, 79.4, 61.8, 51.6, 28.4, 25.4, 14.1; **HRMS** (ESI-Orbitrap): calcd. for $[C_{18}H_{26}NO_5]^+$ (M + H$^+$): 336.1805, found: 336.1806.

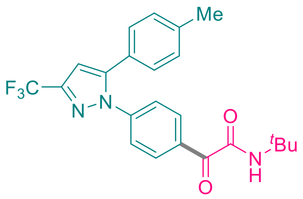

61

***N*-(*tert*-butyl)-2-oxo-2-(4-(5-(p-tolyl)-3-(trifluoromethyl)-1*H*-pyrazol-1-yl)phenyl) acetamide (61).** Yellow solid, 73.0 mg, 85% yield (from aryl bromide derivative of Celecoxib); 78.1 mg, 91% yield (from aryl iodide derivative of Celecoxib), m.p. = 89.5 – 91.3 °C. **^{1}H-NMR** ($CDCl_3$, 400 MHz): δ 8.34 (d, J = 8.7 Hz, 2H), 7.42 (d, J = 8.7 Hz, 2H), 7.13 (dd, J = 18.6, 8.1 Hz, 4H), 6.99 (br s, 1H), 6.73 (s, 1H), 2.37 (s, 3H), 1.45 (s, 9H); **^{13}C-NMR** ($CDCl_3$, 101 MHz): δ 187.1, 160.7 145.3, 144.0 (q, J = 78.1, 39.6 Hz), 143.6, 139.7, 134.8, 132.6, 132.4, 130.6, 129.7, 129.0, 128.8, 128.5, 126.0, 125.5, 124.7, 121.2 (q, J = 269.1 Hz), 106.4 (d, J = 1.8 Hz), 51.8, 28.4, 21.4; **HRMS** (ESI-Orbitrap): calcd. for $[C_{23}H_{23}F_3N_3O_2]^+$ (M + H$^+$): 430.1737, found: 430.1739.

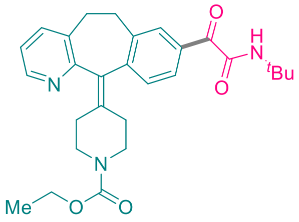

62

Ethyl 4-(8-(2-(*tert*-butylamino)-2-oxoacetyl)-5,6-dihydro-11*H*-benzo[5,6]cyclohepta [1,2-*b*]pyridin-11-ylidene)piperidine-1-carboxylate (62). Yellow solid, 82.7 mg, 87% yield (from aryl iodide derivative of Loratadine), m.p. = 175.9 – 177.8 ºC. **^{1}H-NMR** ($CDCl_3$, 400 MHz): δ 8.37 (d, J = 3.6 Hz, 1H), 8.14 (s, 1H), 8.06 (d, J = 8.0 Hz, 1H), 7.41 (d, J = 7.5 Hz, 1H), 7.28 (s, 1H), 7.07 (dd, J = 7.7, 4.8 Hz, 1H), 6.95 (br s, 1H), 4.10 (q, J = 7.1 Hz, 2H), 3.79 (s, 2H), 3.50 – 3.39 (m, 1H), 3.38 – 3.28 (m, 1H), 3.18 – 3.02 (m, 2H), 2.94 – 2.78 (m, 2H), 2.54 – 2.42 (m, 1H), 2.38 – 2.21 (m, 3H), 1.40 (s, 9H), 1.21 (t, J = 7.1 Hz, 3H); **^{13}C-NMR** ($CDCl_3$, 101 MHz): δ 188.0, 161.2, 156.1, 155.4, 146.7, 145.7, 138.1, 138.1 137.8, 134.6, 133.5, 132.5, 131.8, 129.3, 129.1, 122.4, 61.3, 51.6, 44.8, 31.6 (d, J = 4.4 Hz), 30.9, 30.5, 28.3, 28.3; **HRMS** (ESI-Orbitrap): calcd. for $[C_{28}H_{34}N_3O_4]^+$ (M + H^+): 476.2544, found: 476.2544.

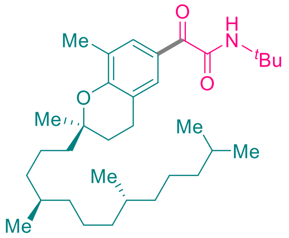

63

***N*-(*tert*-butyl)-2-((*R*)-2,8-dimethyl-2-((4*R*,8*R*)-4,8,12-trimethyltridecyl)chroman-6-yl)-2-oxoacetamide (63).** Yellow oil, 41.1 mg, 40% yield (from aryl iodide derivative of δ-Vitamin E). **^{1}H-NMR** ($CDCl_3$, 400 MHz): δ 8.10 (s, 1H), 7.96 (s, 1H), 6.98 (br s, 1H), 2.79 (t, J = 6.6 Hz, 2H), 2.18 (s, 3H), 1.92 – 1.69 (m, 2H), 1.63 – 0.97 (m, 33H), 0.94 – 0.74 (m, 12H); **^{13}C-NMR** ($CDCl_3$, 101 MHz): δ 186.9, 162.2, 158.4, 132.1, 131.7, 126.7, 124.7, 120.3, 78.0, 51.5, 40.3, 39.5, 37.6, 37.5, 37.5, 37.4, 32.9, 32.8, 31.0, 28.6, 28.1, 24.9, 24.6, 24.5, 22.9, 22.8, 22.3, 21.1, 19.9, 19.8, 16.2; **HRMS** (ESI-Orbitrap): calcd. for $[C_{33}H_{56}NO_3]^+$ (M + H^+): 514.4255, found: 514.4263.

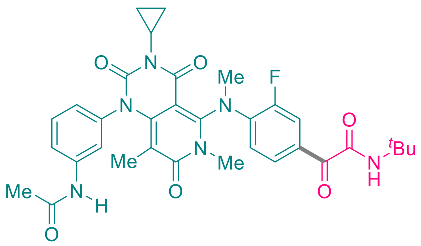

64

2-(4-((1-(3-acetamidophenyl)-3-cyclopropyl-6,8-dimethyl-2,4,7-trioxo-1,2,3,4,6,7-hexahydropyrido[4,3-*d*]pyrimidin-5-yl)(methyl)amino)-3-fluorophenyl)-*N*-(*tert*-butyl)-2-oxoacetamide (64). Yellow solid, 121.0 mg, 96% yield (from aryl iodide derivative of Trametinib), m.p. = 167.6 – 169.9 ºC. **^{1}H-NMR** ($CDCl_3$, 400 MHz): δ 11.34 – 11.14 (m, 1H), 7.54 (d, *J* = 11.1 Hz, 1H), 7.47 (d, *J* = 8.3 Hz, 1H), 7.41 (t, *J* = 8.1 Hz, 1H), 7.23 – 7.10 (m, 3H), 6.86 (t, *J* = 8.0 Hz, 1H), 6.31 (s, 1H), 3.18 (s, 3H), 3.10 (s, 3H), 2.63 (dt, *J* = 10.7, 3.6 Hz, 1H), 1.83 (s, 3H), 1.37 (s, 9H), 1.32 (s, 3H), 1.03 (d, *J* = 6.9 Hz, 2H), 0.70 (s, 2H); **^{13}C-NMR** ($CDCl_3$, 101 MHz): δ 170.4, 164.7, 164.6, 164.6, 163.8, 154.8 (d, *J* = 249.7 Hz), 151.9, 151.5, 145.1, 144.8, 141.1, 134.3, 134.2, 130.4 (d, *J* = 12.1 Hz), 130.1, 128.1, 126.8, 123.3, 123.2, 123.0, 115.5 (d, *J* = 20.7 Hz), 110.1, 103.6, 90.1, 52.1, 37.3, 34.8, 28.9, 25.3, 22.7, 13.8, 8.5; **HRMS** (ESI-Orbitrap): calcd. for $[C_{33}H_{36}FN_6O_6]^+$ (M + H$^+$): 653.2494, found: 653.2503.

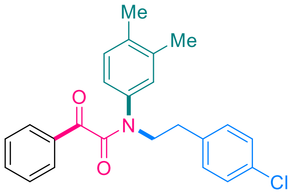

65

***N*-(4-chlorophenethyl)-*N*-(3,4-dimethylphenyl)-2-oxo-2-phenylacetamide (65).** White solid, 20.0 mg, 25% yield, m.p. = 113.2 – 114.6 ºC. **^{1}H-NMR** ($CDCl_3$, 400 MHz): δ 7.62 (d, *J* = 7.2 Hz, 2H), 7.55 (t, *J* = 7.4 Hz, 1H), 7.40 (t, *J* = 7.7 Hz, 2H), 7.31 (d, *J* = 8.3 Hz, 2H), 7.21 (d, *J* = 8.3 Hz, 2H), 6.93 (d, *J* = 7.9 Hz, 1H), 6.77 (s, 1H), 6.68 (d, *J* = 7.9 Hz, 1H), 4.18 (t, *J* = 7.4 Hz, 2H), 2.91 (t, *J* = 7.4 Hz, 2H), 2.16 (s, 3H), 2.09 (s, 3H); **^{13}C-NMR** ($CDCl_3$, 101 MHz): δ 190.7, 167.0, 138.3, 137.2, 136.9, 136.7, 134.3, 133.7, 132.5, 130.7, 130.5, 129.5, 128.8, 128.7, 125.1, 48.9, 33.1, 19.8, 19.5; **HRMS** (ESI-Orbitrap): calcd. for $[C_{24}H_{23}ClNO_2]^+$ (M + H$^+$): 392.1412, found: 392.1411.

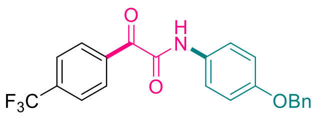

66

***N*-(4-(benzyloxy)phenyl)-2-oxo-2-(4-(trifluoromethyl)phenyl)acetamide (66).** Yellow solid, 24.7 mg, 31% yield, m.p. = 127.1 – 128.1 ºC. **^{1}H-NMR** ($CDCl_3$, 400 MHz): *δ* 8.89 (br s, 1H), 8.52 (d, *J* = 8.2 Hz, 2H), 7.77 (d, *J* = 8.3 Hz, 2H), 7.62 (d, *J* = 8.8 Hz, 2H), 7.48 – 7.30 (m, 5H), 7.01 (d, *J* = 8.8 Hz, 2H), 5.08 (s, 2H); **^{13}C-NMR** ($CDCl_3$, 101 MHz): *δ* 186.8, 158.0, 156.5, 136.8, 136.0, 135.5 (q, *J* = 32.6 Hz), 131.9, 129.8, 128.8, 128.2, 127.6, 125.6 (q, *J* = 3.7 Hz), 123.6 (q, *J* = 272.9 Hz), 121.7, 115.5, 70.4; **HRMS** (ESI-Orbitrap): calcd. for $[C_{22}H_{17}F_3NO_3]^+$ (M + H$^+$): 400.1155, found: 400.1157.

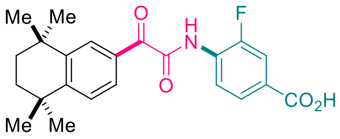

67

3-fluoro-4-(2-oxo-2-(5,5,8,8-tetramethyl-5,6,7,8-tetrahydronaphthalen-2-yl) acetamido) benzoic acid (67). White solid, 18.3 mg, 23% yield, m.p. = 33.2 – 34.9 ºC. **^{1}H-NMR** (DMSO-*d*$_6$, 400 MHz): *δ* 13.18 (br s, 1H), 11.07 (s, 1H), 8.14 (t, *J* = 7.8 Hz, 1H), 7.99 (s, 1H), 7.85 (d, *J* = 8.1 Hz, 1H), 7.79 (d, *J* = 10.9 Hz, 1H), 7.74 (d, *J* = 8.1 Hz, 1H), 7.57 (d, *J* = 8.3 Hz, 1H), 1.66 (s, 4H), 1.26 (s, 12H); **^{13}C-NMR** (DMSO-*d*$_6$, 101 MHz): *δ* 188.7, 166.0, 164.7, 153.4 (d, *J* = 247.8 Hz), 152.5, 145.5, 130.1, 129.0, 128.8, 127.9 (d, *J* = 5.5 Hz), 127.6, 127.2, 126.1, 124.0, 116.5 (d, *J* = 21.4 Hz), 34.7, 34.1, 31.5, 31.2; **HRMS** (ESI-Orbitrap): calcd. for $[C_{23}H_{25}FNO_4]^+$ (M + H$^+$): 398.1762, found: 398.1789.

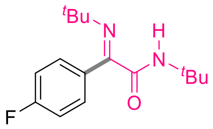

68

***N*-(*tert*-butyl)-2-(*tert*-butylimino)-2-(4-fluorophenyl)acetamide (68).** Yellow solid, 58% yield by recrystallization, m.p. = 122.4 – 124.0 ºC. **^{1}H-NMR** (DMSO-*d6*, 400 MHz): *δ* 8.32 (s, 1H), 7.85 – 7.66 (m, 2H), 7.24 (t, *J* = 8.9 Hz, 2H), 1.36 (s, 9H), 1.34 (s, 9H); **^{13}C-NMR** (DMSO-*d*$_6$, 101 MHz): *δ* 167.2, 163.2 (d, *J* = 247.2 Hz), 156.5, 134.1 (d, *J* = 2.9 Hz), 129.1

(d, J = 8.7 Hz), 115.1 (d, J = 21.7 Hz), 56.9, 51.1, 30.3, 28.2; **^{19}F NMR** (DMSO-d_6, 376 MHz) δ -111.33 (s, 1F). **HRMS** (ESI-Orbitrap): calcd. for $[C_{16}H_{24}FN_2O]^+$ (M + H$^+$): 279.1867, found: 279.1878.

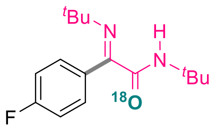

69

***N*-(*tert*-butyl)-2-(tert-butylimino)-2-(4-fluorophenyl)acetamide-^{18}O (69).** Yellow solid, 64% yield by ^{19}F NMR, m.p. = 128.9 – 130.4 °C. **HRMS** (ESI-Orbitrap): calcd. for $[C_{16}H_{24}FN_2{}^{18}O]^+$ (M + H$^+$): 281.1910, found: 279.1921.

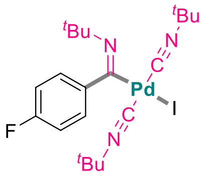

70

Bis((*tert*-butyl-λ^4-azanylidyne)methyl)((*tert*-butylimino)(4-fluorophenyl)methyl) palladium (IV) iodide (70). Yellow solid, 57.7 mg, 50 % yield, m.p. = 152.1 – 154.0 ºC. **^{1}H-NMR** ($CDCl_3$, 400 MHz): δ 7.83 (dd, J = 8.7, 5.7 Hz, 2H), 6.99 (t, J = 8.7 Hz, 2H), 1.55 (s, 9H), 1.38 (s, 18H); **^{13}C-NMR** ($CDCl_3$, 101 MHz): δ 165.3 (d, J = 246.9 Hz), 161.6, 141.4 (d, J = 3.0 Hz), 130.6 (d, J = 8.2 Hz), 114.7 (d, J = 21.5 Hz), 58.0, 57.3, 31.3, 29.8; **^{19}F NMR** $CDCl_3$, 376 MHz) δ -113.33 (s, 1F); **HRMS** (ESI-Orbitrap): calcd. for $[C_{21}H_{32}FIN_3Pd]^+$ (M + H$^+$): 578.0654, found: 578.0678.

7. Gram-scale experiments

*Synthesis of compound **3** from bromobenzene in 10 mmol scale*

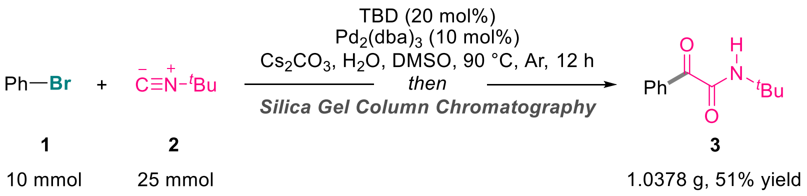

A 100 mL oven-dried single-necked flask equipped with a stir bar was charged with bromobenzene (**1**, 1.5701 g, 1055.9 μL, 10.0 mmol, 1.0 equiv.), TBD (278.4 mg, 20 mol%), $Pd_2(dba)_3$ (915.7 mg, 10 mol%), Cs_2CO_3 (6516.4 mg, 20.0 mmol, 2.0 equiv.), H_2O (5.0 mL) and extra dry DMSO (50 mL). The vial was evacuated and backfilled with argon for three times and then *tert*-butyl isocyanide (**2**, 2827.6 μL, 25 mmol) was added by syringe under argon. The vial was then sealed and was stirred at 90 ºC (oil bath) for 12 h. After the completion of the reaction monitored by TLC (thin layer chromatography), the reaction mixture was cooled to the room temperature and treated with water (80 mL), extracted with CH_2Cl_2 (3 × 80 mL), washed with brine (2 × 200 mL). Then the organic layer was dried over anhydrous Na_2SO_4, filtered and concentrated. The residue was purified by flash column chromatography on silica gel (60 g) and eluted with petroleum ether/ethyl acetate (30:1 – 10:1) to afford the product **3** (1.0378 g, 51% yield).

*Synthesis of compound **3** from bromobenzene in 20 mmol scale*

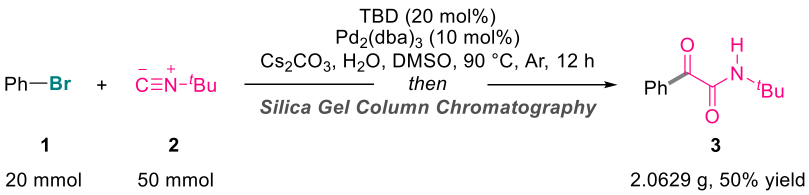

A 350 mL oven-dried single-necked flask equipped with a stir bar was charged with bromobenzene (**1**, 3.1402 g, 2111.8 μL, 20.0 mmol, 1.0 equiv.), TBD (556.8 mg, 20 mol%), $Pd_2(dba)_3$ (1831.4 mg, 10 mol%), Cs_2CO_3 (13.0328 g, 20.0 mmol, 2.0 equiv.), H_2O (10.0 mL) and extra dry DMSO (100 mL). The vial was evacuated and backfilled with argon for three times and then *tert*-butyl isocyanide (**2**, 5655.1 μL, 50 mmol) was added by syringe under argon. The vial was then sealed and was stirred at 90 ºC (oil bath) for 12 h. After the completion of the reaction monitored by TLC (thin layer chromatography), the reaction

mixture was cooled to the room temperature and treated with water (150 mL), extracted with CH_2Cl_2 (3 × 150 mL), washed with brine (2 × 250 mL). Then the organic layer was dried over anhydrous Na_2SO_4, filtered and concentrated. The residue was purified by flash column chromatography on silica gel (90 g) and eluted with petroleum ether/ethyl acetate (30:1 – 10:1) to afford the product **3** (2.0629 g, 50% yield).

Synthesis of compound ***3*** *from iodobenzene in 10 mmol scale*

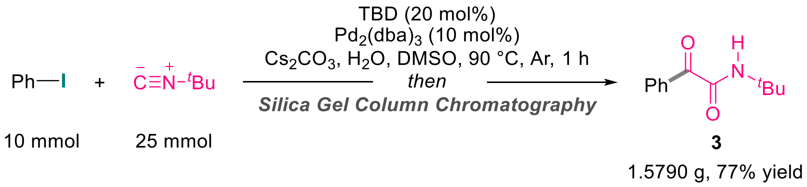

A 100 mL oven-dried single-necked flask equipped with a stir bar was charged with iodobenzene (2.0401 g, 1120.9 μL, 10.0 mmol, 1.0 equiv.), TBD (278.4 mg, 20 mol%), $Pd_2(dba)_3$ (915.7 mg, 10 mol%), Cs_2CO_3 (6516.4 mg, 20.0 mmol, 2.0 equiv.), H_2O (5.0 mL) and extra dry DMSO (50 mL). The flask was evacuated and backfilled with argon for three times and then *tert*-butyl isocyanide (2827.6 μL, 25 mmol) was added by syringe under argon. The flask was then sealed and was stirred at 90 ºC (oil bath) for 1 h. After the completion of the reaction monitored by TLC (thin layer chromatography), the reaction mixture was cooled to the room temperature and treated with water (80 mL), extracted with CH_2Cl_2 (3 × 80 mL), washed with brine (2 × 200 mL). Then the organic layer was dried over anhydrous Na_2SO_4, filtered and concentrated. The residue was purified by flash column chromatography on silica gel (60 g) and eluted with petroleum ether/ethyl acetate (30:1 – 10:1) to afford the product **3** (1.5790 g, 77% yield).

Synthesis of compound ***3*** *from iodobenzene with 5 mol%* $Pd_2(dba)_3$ *in 20 mmol scale*

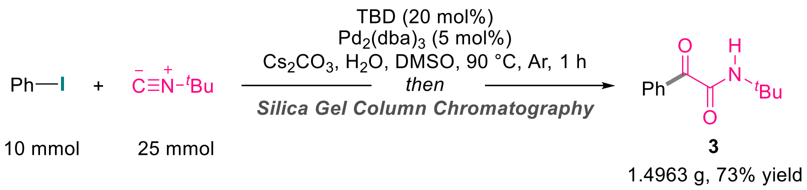

A 100 mL oven-dried single-necked flask equipped with a stir bar was charged with iodobenzene (2.0401 g, 1120.9 μL, 10.0 mmol, 1.0 equiv.), TBD (278.4 mg, 20 mol%), $Pd_2(dba)_3$ (457.9 mg, 5 mol%), Cs_2CO_3 (6516.4 mg, 20.0 mmol, 2.0 equiv.), H_2O (5.0 mL) and extra dry DMSO (50 mL). The flask was evacuated and backfilled with argon for three times and then *tert*-butyl isocyanide (2827.6 μL, 25 mmol) was added by syringe under argon. The flask was then sealed and was stirred at 90 ºC (oil bath) for 1 h. After the completion of the reaction monitored by TLC (thin layer chromatography), the reaction mixture was cooled to the room temperature and treated with water (80 mL), extracted with CH_2Cl_2 (3 × 80 mL), washed with brine (2 × 200 mL). Then the organic layer was dried over anhydrous Na_2SO_4, filtered and concentrated. The residue was purified by flash column chromatography on silica gel (60 g) and eluted with petroleum ether/ethyl acetate (30:1 – 10:1) to afford the product **3** (1.4963 g, 73% yield).

8. Mechanistic studies

8.1 Control experiment

8.1.1 Crossover reaction

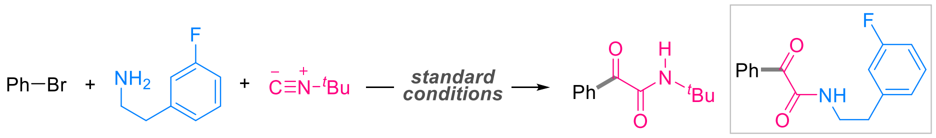

A 4-mL flame-dried vial with a magnetic stir bar was charged with bromobenzene (20.9 μL, 0.2 mmol), 2-(3-fluorophenyl)ethan-1-amine (65.3μL, 0.5 mmol, 2.5 equiv.), TBD (5.6 mg, 20 mol%), $Pd_2(dba)_3$ (18.3 mg, 10 mol%), Cs_2CO_3 (130.3 mg, 0.4 mmol), H_2O (100 μL) and extra dry DMSO (1.0 mL). The vial was evacuated and backfilled with argon for three times and then *tert*-butyl isocyanide (56.6 μL, 0.5 mmol) was added by syringe under argon. The vial was then sealed and was stirred at 90 ºC (oil bath) for 12 h. After the completion of the reaction monitored by TLC (thin layer chromatography), the reaction mixture was cooled to the room temperature and treated with water (15 mL), extracted with CH_2Cl_2 (3 × 15 mL), washed with brine (2 × 40 mL). Then the organic layer was dried over anhydrous Na_2SO_4, filtered and concentrated. The residue was purified by flash column chromatography on silica gel (10 g) and eluted with petroleum ether/ethyl acetate (30:1 – 10:1) to afford the product **3** (19.7 mg, 48% yield), the compound **58** was not detected.

8.1.2 The model reaction in the absence of TBD

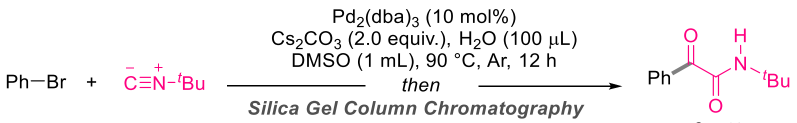

A 4-mL flame-dried vial with a magnetic stir bar was charged with bromobenzene (20.9 μL, 0.2 mmol), $Pd_2(dba)_3$ (18.3 mg, 10 mol%), Cs_2CO_3 (130.3 mg, 0.4 mmol), H_2O (100 μL) and extra dry DMSO (1.0 mL). The vial was evacuated and backfilled with argon for three times and then *tert*-butyl isocyanide (56.6 μL, 0.5 mmol) was added by syringe under argon. The vial was then sealed and was stirred at 90 ºC (oil bath) for 12 h. After the completion of the reaction monitored by TLC (thin layer chromatography), the reaction mixture was cooled to the room temperature and treated with water (15 mL), extracted with CH_2Cl_2 (3

× 15 mL), washed with brine (2 × 40 mL). Then the organic layer was dried over anhydrous Na_2SO_4, filtered and concentrated. The residue was purified by flash column chromatography on silica gel (10 g) and eluted with petroleum ether/ethyl acetate (30:1 – 10:1). However, product **3** was not obtained (0% yield).

8.1.3 The model reaction in the absence of Cs_2CO_3

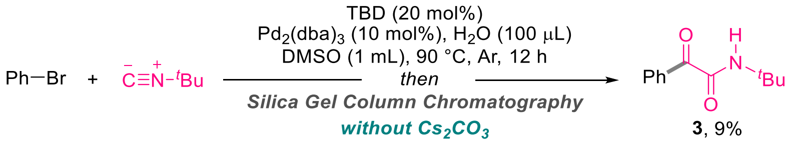

A 4-mL flame-dried vial with a magnetic stir bar was charged with bromobenzene (20.9 μL, 0.2 mmol), TBD (5.6 mg, 20 mol%), $Pd_2(dba)_3$ (18.3 mg, 10 mol%), H_2O (100 μL) and extra dry DMSO (1.0 mL). The vial was evacuated and backfilled with argon for three times and then *tert*-butyl isocyanide (56.6 μL, 0.5 mmol) was added by syringe under argon. The vial was then sealed and was stirred at 90 ºC (oil bath) for 12 h. After the completion of the reaction monitored by TLC (thin layer chromatography), the reaction mixture was cooled to the room temperature and treated with water (15 mL), extracted with CH_2Cl_2 (3 × 15 mL), washed with brine (2 × 40 mL). Then the organic layer was dried over anhydrous Na_2SO_4, filtered and concentrated. The residue was purified by flash column chromatography on silica gel (10 g) and eluted with petroleum ether/ethyl acetate (30:1 – 10:1) to afford the product **3** (3.7 mg, 9% yield).

8.1.4 The model reaction by replacing TBD with MTBD

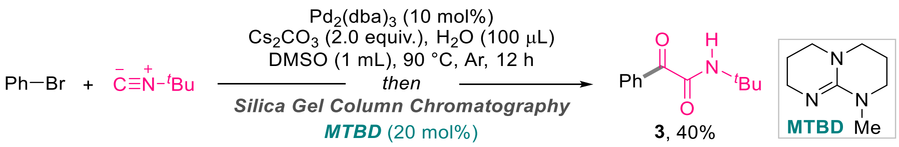

A 4-mL flame-dried vial with a magnetic stir bar was charged with bromobenzene (20.9 μL, 0.2 mmol), MTBD (6.1 mg, 20 mol%), $Pd_2(dba)_3$ (18.3 mg, 10 mol%), Cs_2CO_3 (130.3 mg, 0.4 mmol), H_2O (100 μL) and extra dry DMSO (1.0 mL). The vial was evacuated and backfilled with argon for three times and then *tert*-butyl isocyanide (56.6 μL, 0.5 mmol) was added by syringe under argon. The vial was then sealed and was stirred at 90 ºC (oil bath)

for 12 h. After the completion of the reaction monitored by TLC (thin layer chromatography), the reaction mixture was cooled to the room temperature and treated with water (15 mL), extracted with CH_2Cl_2 (3 × 15 mL), washed with brine (2 × 40 mL). Then the organic layer was dried over anhydrous Na_2SO_4, filtered and concentrated. The residue was purified by flash column chromatography on silica gel (10 g) and eluted with petroleum ether/ethyl acetate (30:1 – 10:1) to afford the product **3** (16.4 mg, 40% yield).

8.2 Isotope labeling experiment

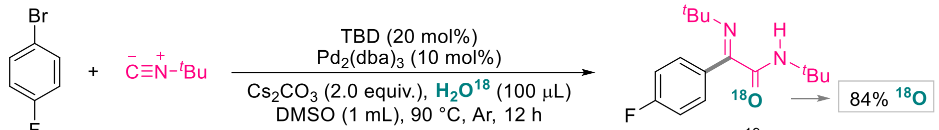

69, 64% by ^{19}F NMR

A 4-mL flame-dried vial with a magnetic stir bar was charged with 1-bromo-4-fluorobenzene (22.0 µL, 0.2 mmol), TBD (5.6 mg, 20 mol%), $Pd_2(dba)_3$ (18.3 mg, 10 mol%), Cs_2CO_3 (130.3 mg, 0.4 mmol), $H_2{}^{18}O$ (100 µL) and extra dry DMSO (1.0 mL). The vial was evacuated and backfilled with argon for three times and then *tert*-butyl isocyanide (56.6 µL, 0.5 mmol) was added by syringe under argon. The vial was then sealed and was stirred at 90 °C (oil bath) for 12 h. After the completion of the reaction monitored by TLC (thin layer chromatography), the reaction mixture was cooled to the room temperature and added with benzotrifluoride (internal standard, 24.6 µL, 0.2 mmol), stirred for additional 1 min at room temperature, filtered through a filter membrane. Then the yield of compound **69** was given in 64% yield determined by ^{19}F-NMR.

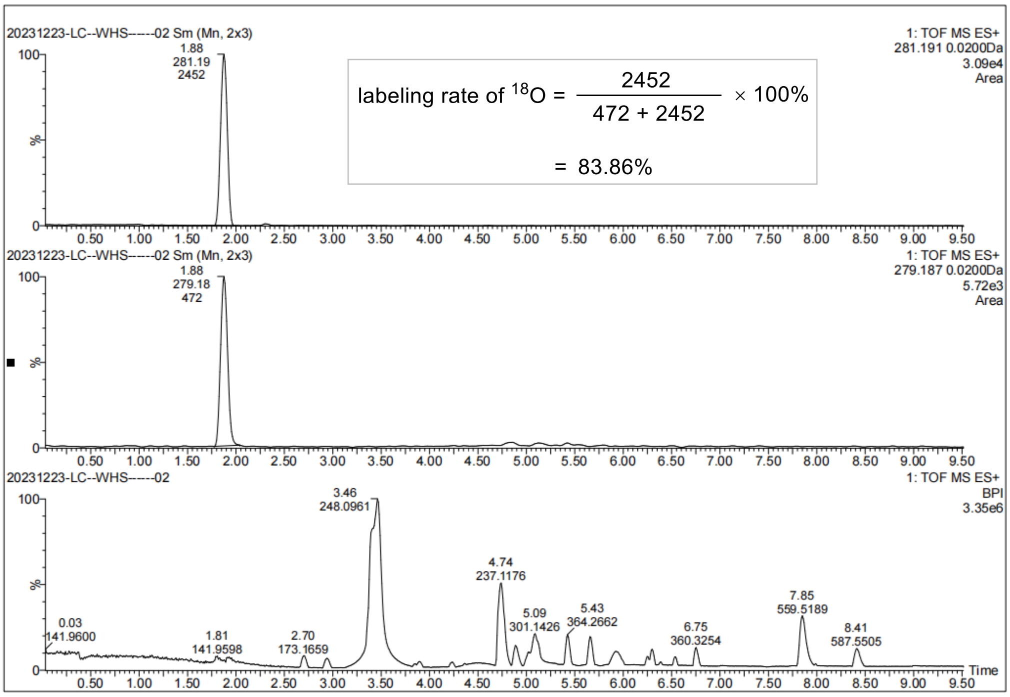

Figure S2. Content determination of ^{18}O labelled midbody

8.3 Control experiment with imidoylpalladium complex

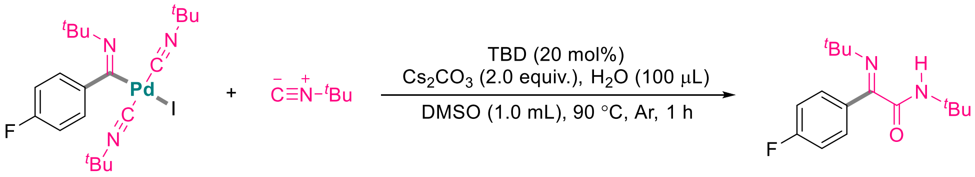

A 4-mL flame-dried vial with a magnetic stir bar was charged with **70** (115.4 mg, 0.2 mmol), TBD (5.6 mg, 20 mol%), Cs_2CO_3 (130.3 mg, 0.4 mmol), H_2O (100 μL) and extra dry DMSO (1.0 mL). The vial was evacuated and backfilled with argon for three times and then *tert*-butyl isocyanide (22.6 μL, 0.2 mmol) was added by syringe under argon. The vial was then sealed and was stirred at 90 ºC (oil bath) for 1 h. After the completion of the reaction monitored by TLC (thin layer chromatography), the reaction mixture was cooled to the room temperature and added with benzotrifluoride (internal standard, 24.6 μL, 0.2 mmol), stirred for additional 1 min at room temperature, filtered through a filter membrane. Then the yield of compound **68** was given in 45% determined by ^{19}F-NMR.

8.4 Control experiment with α-ketoimine amide

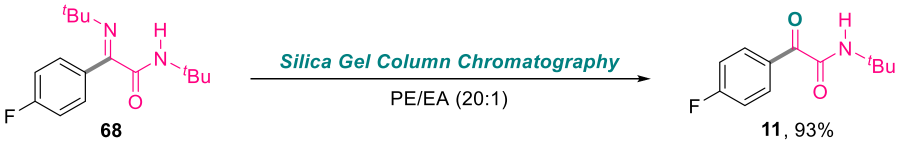

68 → **11**, 93%

The sample **68** (55.6 mg, 0.2 mmol) was eluted by flash column chromatography on silica gel (10 g) and eluted with petroleum ether/ethyl acetate (20:1) to give the product **11** (41.3 mg, 93% yield).

8.5 Hammett's correlation

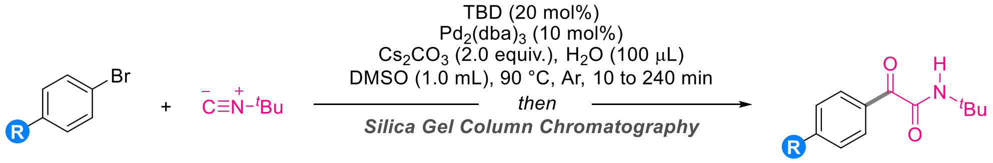

A **2** **B**

A 4-mL flame-dried vial with a magnetic stir bar was charged with substrate (**A1** – **A7**), TBD (5.6 mg, 20 mol%), $Pd_2(dba)_3$ (18.3 mg, 10 mol%), Cs_2CO_3 (130.3 mg, 0.4 mmol), H_2O (100 μL) and extra dry DMSO (1.0 mL). The vial was evacuated and backfilled with argon for three times and then *tert*-butyl isocyanide (56.6 μL, 0.5 mmol) was added by syringe under argon. The vial was then sealed and was stirred at 90 ºC (oil bath) for 10 min – 240 min. After the completion of the reaction monitored by TLC (thin layer chromatography), the reaction mixture was cooled to the room temperature and treated with water (15 mL), extracted with CH_2Cl_2 (3 × 15 mL), washed with brine (2 × 40 mL). Then the organic layer was dried over anhydrous Na_2SO_4, filtered and concentrated.[15,16] The residue was purified by flash column chromatography on silica gel (10 g) and eluted with petroleum ether/ethyl acetate (30:1 – 5:1) to afford the corresponding product.

Table S6. Raw data of Hammett correlation experiment

Substrate (R)	Weight of **A** (mg)	Time (min)	Weight of **B** (mg)	Yield of **B** (%)
A1 (H)	31.4	60	4.5	11.0
A1 (H)	31.4	120	5.5	13.4
A1 (H)	31.4	180	9.2	22.4

A1 (H)	31.4	240	11.1	27.1
A2 (F)	35.0	60	3.9	8.7
A2 (F)	35.0	120	10.4	23.3
A2 (F)	35.0	180	14.1	31.6
A2 (F)	35.0	240	19.8	44.4
A3 (Cl)	38.3	60	6.8	14.2
A3 (Cl)	38.3	120	11.5	24.1
A3 (Cl)	38.3	180	18.7	39.1
A3 (Cl)	38.3	240	27.6	57.7
A4 (CF_3)	45.0	30	14.6	26.7
A4 (CF_3)	45.0	60	22.9	41.9
A4 (CF_3)	45.0	90	30.4	55.7
A4 (CF_3)	45.0	120	41.5	76.0
A5 (CN)	36.4	10	3.6	7.8
A5 (CN)	36.4	20	7.2	15.6
A5 (CN)	36.4	30	12	26.1
A5 (CN)	36.4	40	17.5	38.0
A6 (Me)	34.2	60	1.8	4.1
A6 (Me)	34.2	120	3.7	8.4
A6 (Me)	34.2	180	5.8	13.2
A6 (Me)	34.2	240	7.6	17.3
A7 (OMe)	37.4	60	3.8	8.1
A7 (OMe)	37.4	120	4.6	9.8
A7 (OMe)	37.4	180	7.1	15.1
A7 (OMe)	37.4	240	9.7	20.6

Table S7. Plot of Hammett correlation experiment

Substrate (R)	Rate constant	k_R/k_H	Log (k_R/k_H)	Substitution constant (σ_P)
A1 (H)	0.0955	1	0	0

A2 (F)	0.1923	2.01	0.3	0.06
A3 (Cl)	0.2425	2.54	0.4	0.28
A4 (CF_3)	0.539	5.64	0.75	0.54
A5 (CN)	1.011	10.59	1.02	0.66
A6 (Me)	0.074	0.775	-0.11	-0.17
A7 (OMe)	0.0713	0.747	-0.13	-0.27

The individual rate constants were then plotted against the substituent constants (σm) and a hammett correlation with a negative slope was obtained (ρ = 1.235, R^2= 0.961).

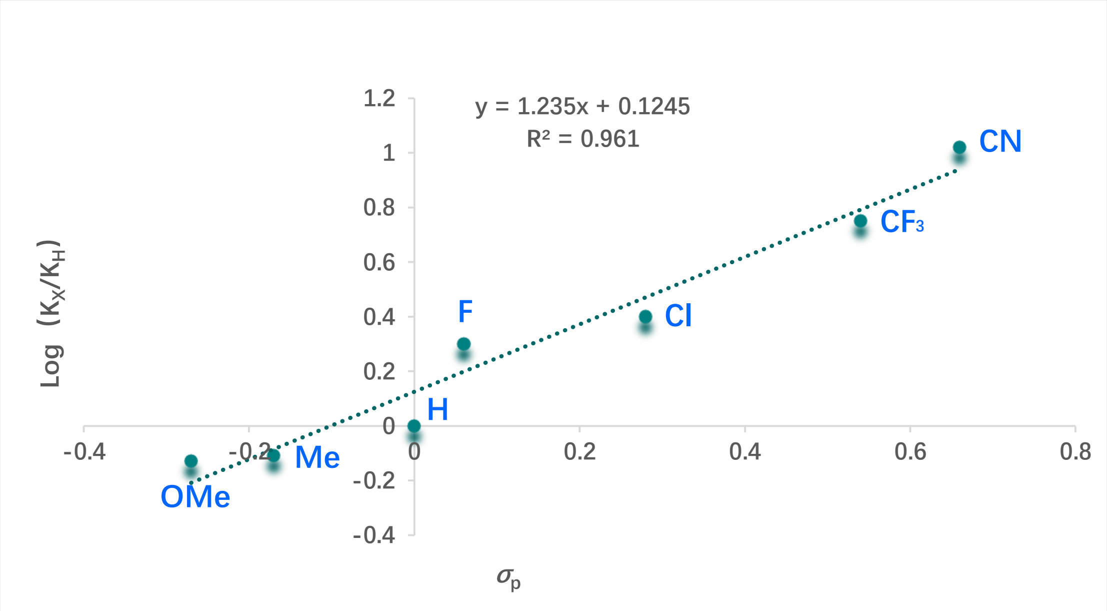

Figure S3. Hammett correlation plot

8.6 Kinetic Studies under standard reaction conditions

8.6.1 Dependence of the reaction rate on concentration of *p*-bromofluorobenzene (**1i**)

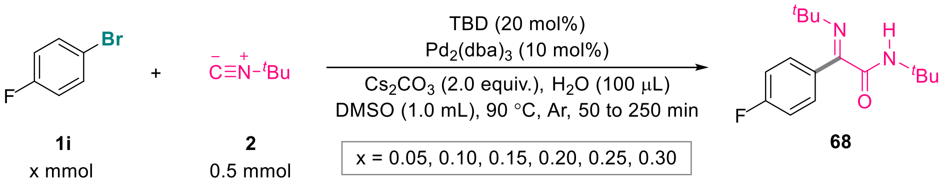

General procedure: **1i (x mmol)**, TBD (5.6 mg, 20 mol%), $Pd_2(dba)_3$ (18.3 mg, 10 mol%), Cs_2CO_3 (130.3 mg, 0.4 mmol), H_2O (100 μL) and extra dry DMSO (1.0 mL) were added to a group of 4 mL glass vials (5 in total) at room temperature. The vials were evacuated and backfilled with argon for three times and then *tert*-butyl isocyanide **2** (56.6

μL, 0.5 mmol) was individually added by syringe under argon. The vials were then sealed and the reaction mixture was stirred at 90 ºC (oil bath). One of the reaction vials was cooled to room temperature every 50 minutes. The reaction mixture was added with benzotrifluoride (internal standard, 24.6 μL, 0.2 mmol), stirred for additional 1 min at room temperature, and filtered through a filter membrane. The yield of **68** was determined by ^{19}F-NMR using benzotrifluoride as an internal standard.[17,18]

Kinetic profiles of different initial concentrations of **1i** were collected (Figure S4). The rate was plotted against the concentration of **1i** (Figure S5).

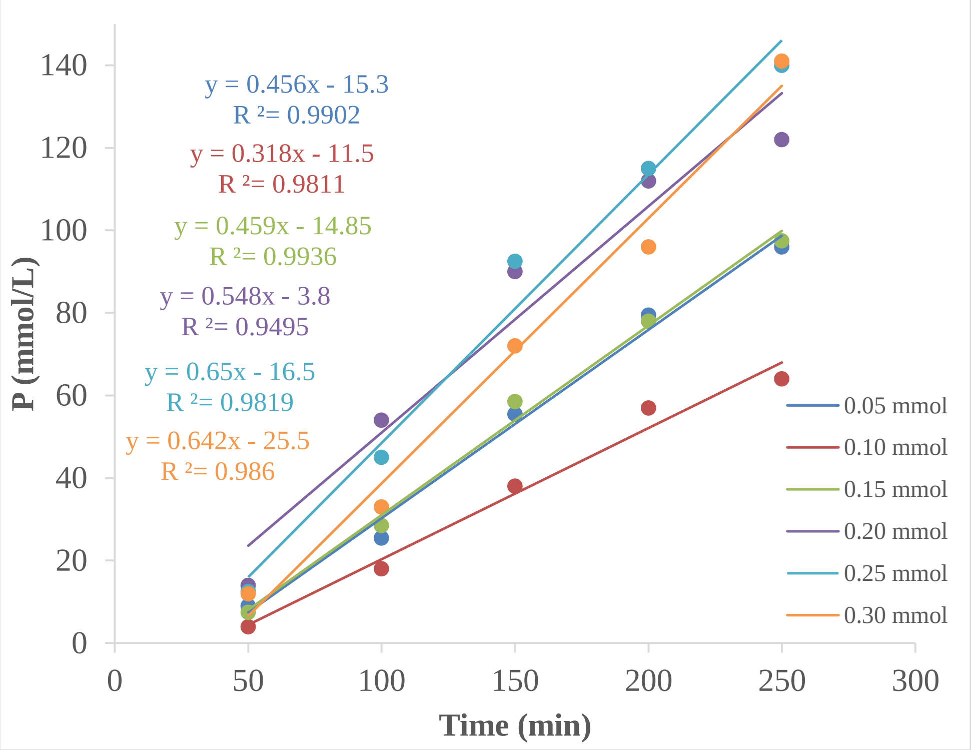

Figure S4. Reaction profiles for **1i**

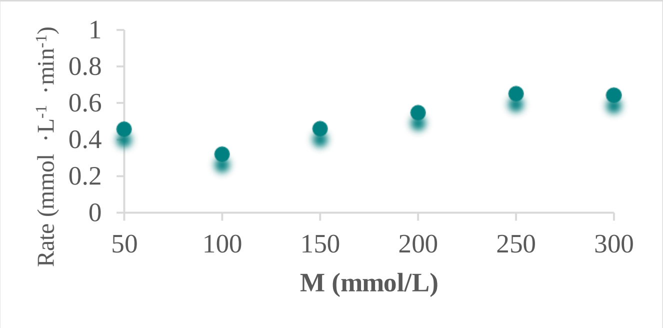

Figure S5. Initial reaction rate dependence on concentration of **1i**

8.6.2 Dependence of the reaction rate on concentration of *tert*-butyl isocyanide (**2**)

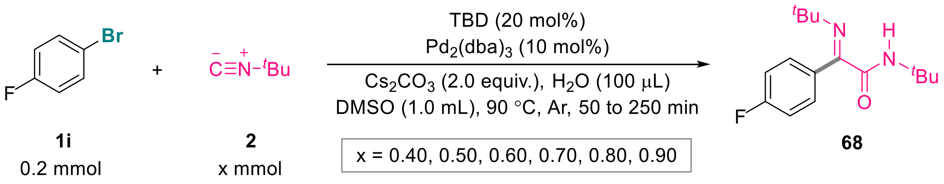

General procedure: **1i** (22.0 µL, 0.2 mmol), TBD (5.6 mg, 20 mol%), $Pd_2(dba)_3$ (18.3 mg, 10 mol%), Cs_2CO_3 (130.3 mg, 0.4 mmol), H_2O (100 µL) and extra dry DMSO (1.0 mL) were added to a group of 4 mL glass vials (5 in total) at room temperature. The vials were evacuated and backfilled with argon for three times and then *tert*-butyl isocyanide **2 (x mmol)** was individually added by syringe under argon. The vials were then sealed and the reaction mixture was stirred at 90 ºC (oil bath). One of the reaction vials was cooled to room temperature every 50 minutes. The reaction mixture was added with benzotrifluoride (internal standard, 24.6 µL, 0.2 mmol), stirred for additional 1 min at room temperature, and filtered through a filter membrane. The yield of **68** was determined by ^{19}F-NMR using benzotrifluoride as an internal standard.[17,18]

Kinetic profiles of different initial concentrations of **2** were collected (Figure S6). The rate was plotted against the concentration of **2** (Figure S7).

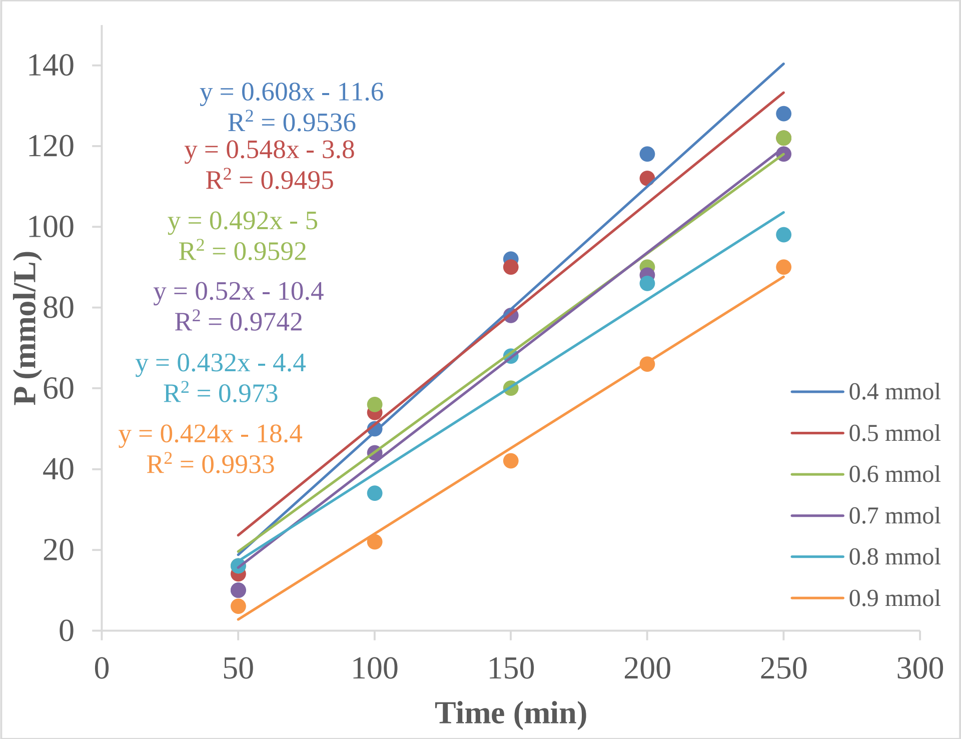

Figure S6. Reaction profiles for **2**

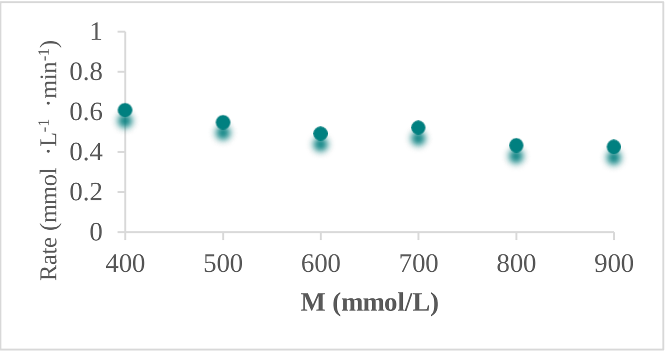

Figure S7. Initial reaction rate dependence on concentration of **2**

8.6.3 Dependence of the reaction rate on concentration of **H_2O**

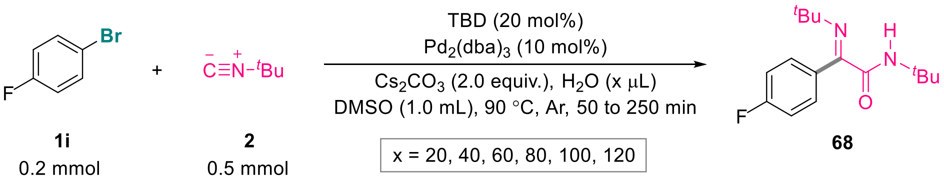

General procedure: **1i** (22.0 μL, 0.2 mmol), TBD (5.6 mg, 20 mol%), $Pd_2(dba)_3$ (18.3 mg, 10 mol%), Cs_2CO_3 (130.3 mg, 0.4 mmol), **H_2O (x μL)** and extra dry DMSO (1.0 mL) were added to a group of 4 mL glass vials (5 in total) at room temperature. The vials were evacuated and backfilled with argon for three times and then *tert*-butyl isocyanide **2** (56.6 μL, 0.5 mmol) was individually added by syringe under argon. The vials were then sealed and the reaction mixture was stirred at 90 ºC (oil bath). One of the reaction vials was cooled to room temperature every 50 minutes. The reaction mixture was added with benzotrifluoride (internal standard, 24.6 μL, 0.2 mmol), stirred for additional 1 min at room temperature, and filtered through a filter membrane. The yield of **68** was determined by ^{19}F-NMR using benzotrifluoride as an internal standard.[17,18]

Kinetic profiles of different initial concentrations of **H_2O** were collected (Figure S8). The rate was plotted against the concentration of **H_2O** (Figure S9).

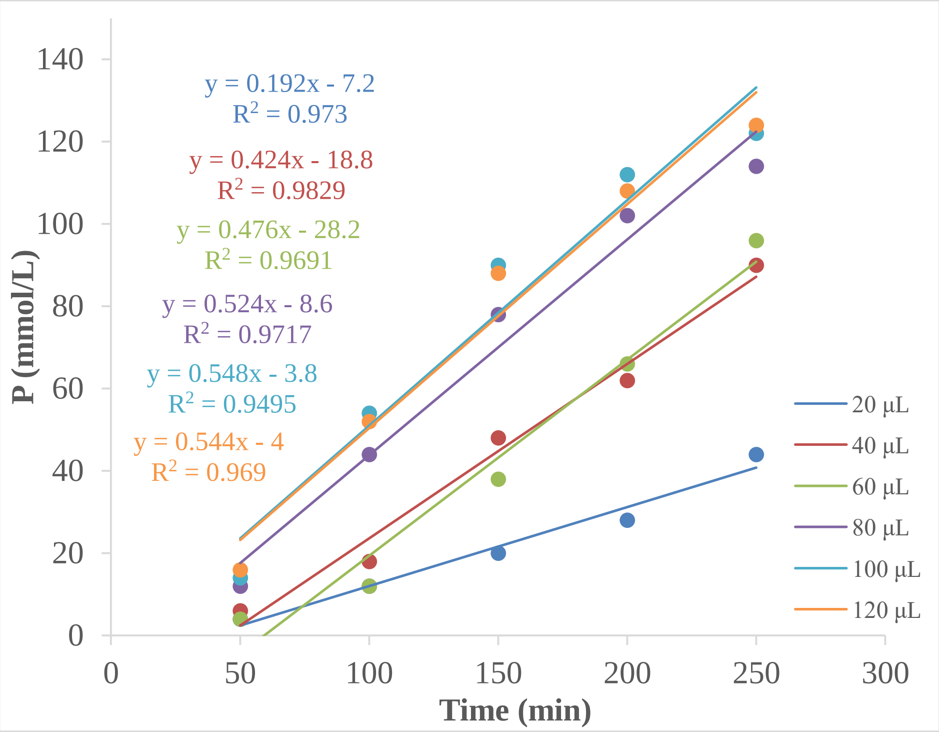

Figure S8. Reaction profiles for **H_2O**

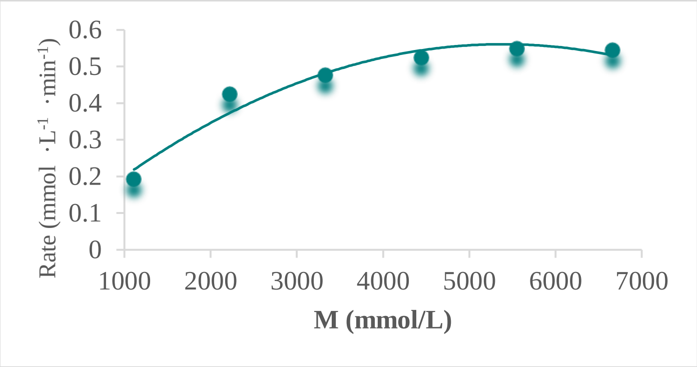

Figure S9. Initial reaction rate dependence on concentration of **H_2O**

8.6.4 Dependence of the reaction rate on concentration of **$Pd_2(dba)_3$**

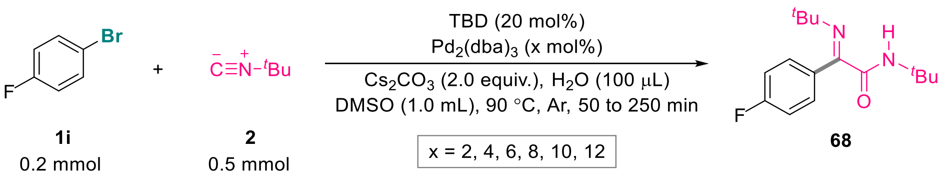

1i 0.2 mmol; **2** 0.5 mmol; **68**

General procedure: **1i** (22.0 μL, 0.2 mmol), TBD (5.6 mg, 20 mol%), **$Pd_2(dba)_3$ (x mol%)**, Cs_2CO_3 (130.3 mg, 0.4 mmol), H_2O (100 μL) and extra dry DMSO (1.0 mL) were added to a group of 4 mL glass vials (5 in total) at room temperature. The vials were

evacuated and backfilled with argon for three times and then *tert*-butyl isocyanide **2** (56.6 μL, 0.5 mmol) was individually added by syringe under argon. The vials were then sealed and the reaction mixture was stirred at 90 °C (oil bath). One of the reaction vials was cooled to room temperature every 50 minutes. The reaction mixture was added with benzotrifluoride (internal standard, 24.6 μL, 0.2 mmol), stirred for additional 1 min at room temperature, and filtered through a filter membrane. The yield of **68** was determined by ^{19}F-NMR using benzotrifluoride as an internal standard.[17,18]

Kinetic profiles of different initial concentrations of **$Pd_2(dba)_3$** were collected (Figure S10). The rate was plotted against the concentration of **$Pd_2(dba)_3$** (Figure S11).

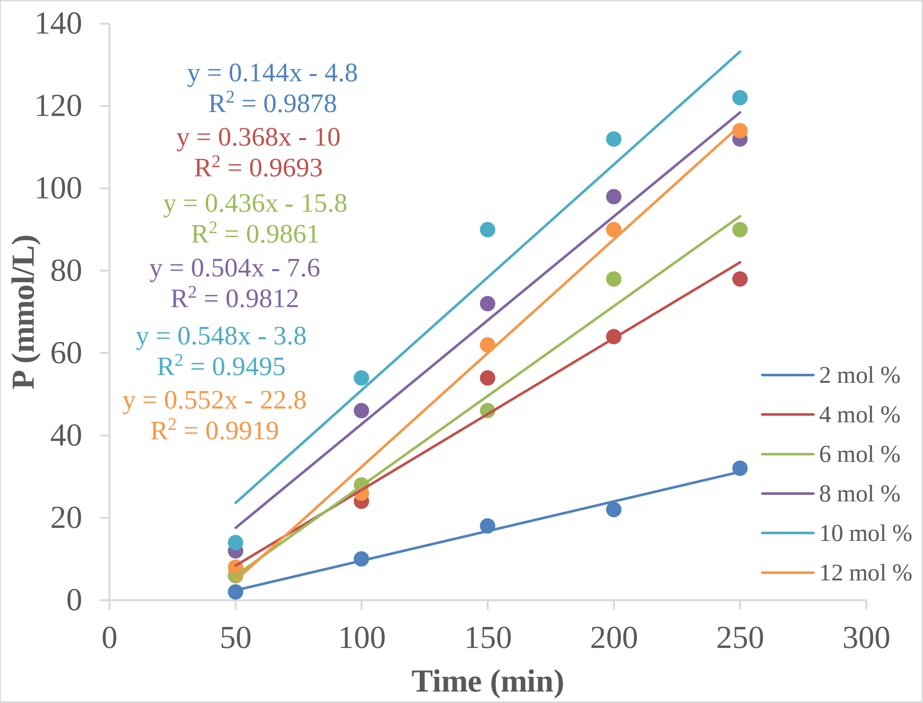

Figure S10. Reaction profiles for **$Pd_2(dba)_3$**

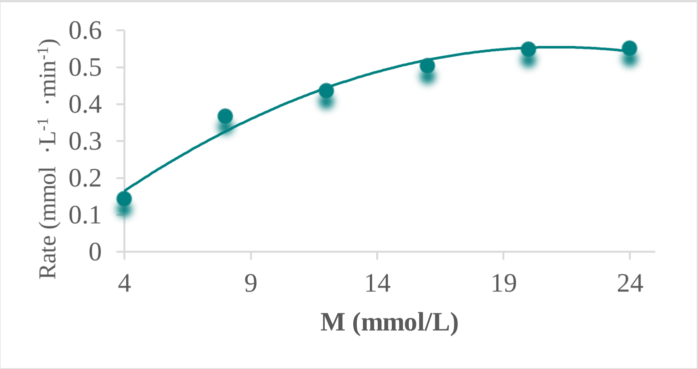

Figure S11. Initial reaction rate dependence on concentration of **$Pd_2(dba)_3$**

8.6.5 Dependence of the reaction rate on concentration of **TBD**

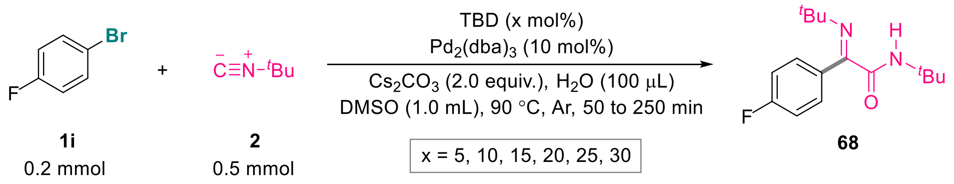

General procedure: **1i** (22.0 μL, 0.2 mmol), **TBD (x mol%)**, $Pd_2(dba)_3$ (18.3 mg, 10 mol%), Cs_2CO_3 (130.3 mg, 0.4 mmol), H_2O (100 μL) and extra dry DMSO (1.0 mL) were added to a group of 4 mL glass vials (5 in total) at room temperature. The vials were evacuated and backfilled with argon for three times and then *tert*-butyl isocyanide **2** (56.6 μL, 0.5 mmol) was individually added by syringe under argon. The vials were then sealed and the reaction mixture was stirred at 90 ºC (oil bath). One of the reaction vials was cooled to room temperature every 50 minutes. The reaction mixture was added with benzotrifluoride (internal standard, 24.6 μL, 0.2 mmol), stirred for additional 1 min at room temperature, and filtered through a filter membrane. The yield of **68** was determined by ^{19}F-NMR using benzotrifluoride as an internal standard.[17,18]

Kinetic profiles of different initial concentrations of **TBD** were collected (Figure S12). The rate was plotted against the concentration of **TBD** (Figure S13).

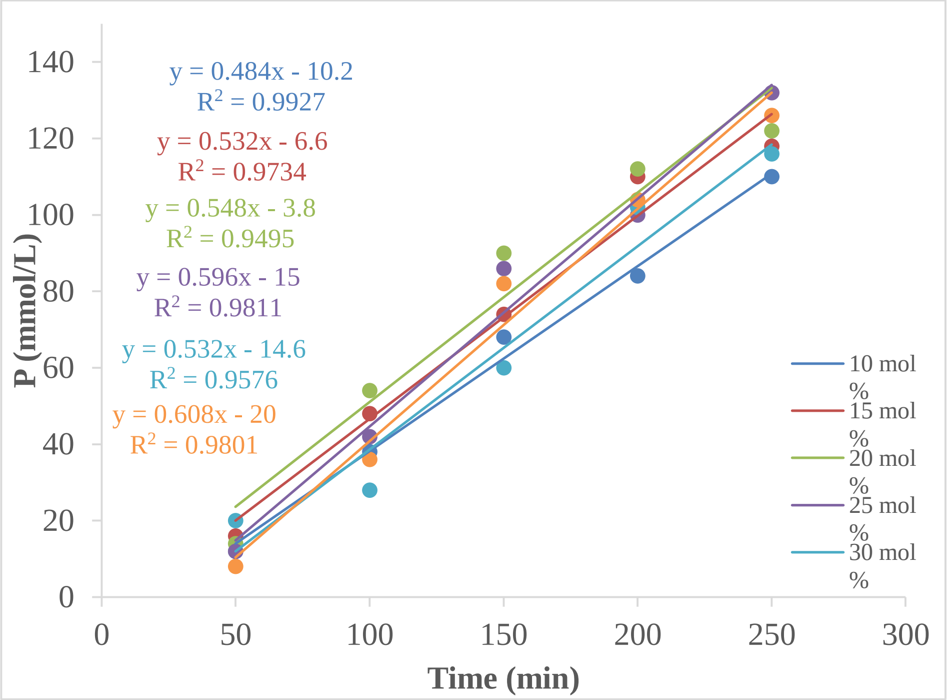

Figure S12. Reaction profiles for **TBD**

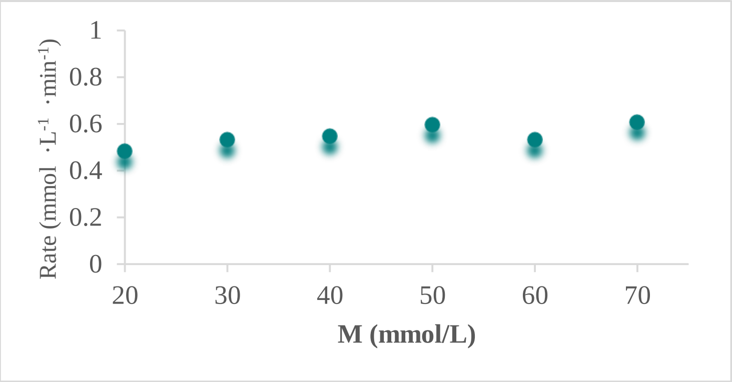

Figure S13. Initial reaction rate dependence on concentration of **TBD**

9. Single crystal X-ray diffraction

9.1 Cultivation of single crystals

Compounds **6**, **68** and **70** were dissolved in a mixture of ethyl acetate and petroleum ether solution, respectively, then put it in the ambient temperature. Suitable crystals of compounds **6**, **68** and **70** were obtained by slowly evaporating a mixture of ethyl acetate and petroleum ether solution at ambient temperature.

9.2 Crystal measurement

Compounds **6**, **68** and **70**, were collected at 100 K on a Rigaku Oxford Diffraction Supernova Dual Source, Cu at Zero equipped with an AtlasS2 CCD using Cu Kα radiation. The data were collected and processed using CrysAlisPro. The structures were solved by direct methods using Olex2 software, and the non-hydrogen atoms were located from the trial structure and then refined anisotropically with SHELXL-2018 using a full-matrix least squares procedure based on F^2. The weighted *R* factor, *wR* and goodness-of-fit S values were obtained based on F^2. The hydrogen atom positions were fixed geometrically at the calcd. distances and allowed to ride on their parent atoms.

CCDC-2338684 (**6**), CCDC-2338685 (**68**), CCDC-2338686 (**70**) contain the supplementary crystallographic data for this paper. These data can be obtained free of charge from The Cambridge Crystallographic Data Centre via www.ccdc.cam.ac.uk/data_request/cif.

9.3 X-ray crystallographic data of **6**, **68** and **70**

The ellipsoids are shown at 30% probability levels.

Table S8. X-ray crystallographic data of 6

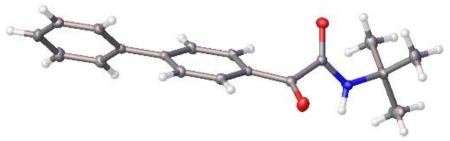

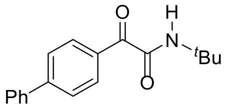

6 (CCDC:2338684)

**Identification code**	**6**
Empirical formula	$C_{18}H_{19}NO_2$
Formula weight	281.34
Temperature/K	170.00(10)
Crystal system	monoclinic
Space group	$P2_1/n$
a/Å	5.9331(9)
b/Å	26.240(3)
c/Å	9.8813(11)
α/°	90
β/°	104.418(13)
γ/°	90
Volume/Å^3	1489.9(3)
Z	4
ρ_{calc}g/cm^3	1.254
μ/mm^{-1}	0.081
F(000)	600.0
Crystal size/mm^3	0.15 × 0.13 × 0.12
Radiation	Mo Kα (λ = 0.71073)
2θ range for data collection/°	4.53 to 49.996
Index ranges	-5 ≤ h ≤ 7, -29 ≤ k ≤ 31, -11 ≤ l ≤ 11
Reflections collected	6245
Independent reflections	2612 [R_{int} = 0.0318, R_{sigma} = 0.0455]
Data/restraints/parameters	2612/0/193
Goodness-of-fit on F^2	1.079
Final R indexes [I>=2σ (I)]	R_1 = 0.0473, wR_2 = 0.0996
Final R indexes [all data]	R_1 = 0.0631, wR_2 = 0.1089
Largest diff. peak/hole / e Å^{-3}	0.17/-0.20

Table S9. X-ray crystallographic data of 68

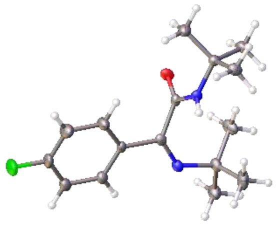

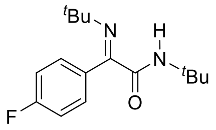

68 (CCDC:2338685)

**Identification code**	**68**
Empirical formula	$C_{16}H_{23}FN_2O$
Formula weight	278.36
Temperature/K	149.94(12)
Crystal system	triclinic
Space group	P-1
a/Å	9.3415(10)
b/Å	10.0623(13)
c/Å	19.6690(17)
α/°	81.197(9)
β/°	78.568(8)
γ/°	63.180(12)
Volume/Å^3	1612.9(3)
Z	4
ρ_{calc}g/cm^3	1.146
μ/mm^{-1}	0.080
F(000)	600.0
Crystal size/mm^3	0.15 × 0.12 × 0.1
Radiation	Mo Kα (λ = 0.71073)
2θ range for data collection/°	4.236 to 49.998
Index ranges	-11 ≤ h ≤ 11, -11 ≤ k ≤ 11, -20 ≤ l ≤ 23
Reflections collected	10972
Independent reflections	5670 [R_{int} = 0.0384, R_{sigma} = 0.0617]
Data/restraints/parameters	5670/0/377
Goodness-of-fit on F^2	1.056
Final R indexes [I>=2σ (I)]	R_1 = 0.0871, wR_2 = 0.2139
Final R indexes [all data]	R_1 = 0.1141, wR_2 = 0.2326
Largest diff. peak/hole / e Å^{-3}	0.41/-0.34

Table S10. X-ray crystallographic data of 70

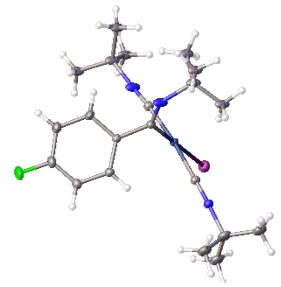

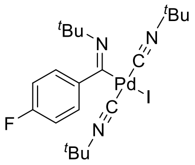

70 (CCDC:2338686)

**Identification code**	**70**
Empirical formula	$C_{21}H_{31}FIN_3Pd$
Formula weight	577.79
Temperature/K	293.05(13)
Crystal system	orthorhombic
Space group	$P2_12_12_1$
a/Å	9.84600(10)
b/Å	15.3637(2)
c/Å	17.0733(3)
α/°	90
β/°	90
γ/°	90
Volume/Å^3	2582.70(6)
Z	4
ρ_{calc}g/cm^3	1.486
μ/mm^{-1}	15.306
F(000)	1144.0
Crystal size/mm^3	0.14 × 0.1 × 0.08
Radiation	Cu Kα (λ = 1.54184)
2θ range for data collection/°	7.742 to 148.468
Index ranges	-11 ≤ h ≤ 8, -18 ≤ k ≤ 19, -20 ≤ l ≤ 21
Reflections collected	12944
Independent reflections	5108 [R_{int} = 0.0288, R_{sigma} = 0.0328]
Data/restraints/parameters	5108/241/181
Goodness-of-fit on F^2	1.035
Final R indexes [I>=2σ (I)]	R_1 = 0.0553, wR_2 = 0.1520
Final R indexes [all data]	R_1 = 0.0592, wR_2 = 0.1557
Largest diff. peak/hole / e Å^{-3}	1.29/-1.25
Flack parameter 0.012(6)	Flack parameter 0.012(6)

10. References

[1] V. Bacauanu, S. Cardinal, M. Yamauchi, M. Kondo, D. F. Fernández, R. Remy, D. W. C. MacMillan, *Angew. Chem., Int. Ed.* **2018**, *57*, 12543–12548; *Angew. Chem.* **2018**, *130*, 12723–12728.

[2] X. Shao, S. J. Malcolmson, *Org. Lett.* **2019**, *21*, 7380–7385.

[3] V. Magné, L. T. Ball, *Chem. - Eur. J.* **2019**, *25*, 8903–8910.

[4] J. Wang, R. Li, Z. Dong, P. Liu, G. Dong, *Nat. Chem.* **2018**, *10*, 866–872.

[5] L. Gong, H.-B. Sun, L.-F. Deng, X. Zhang, J. Liu, S. Yang, D. Niu, *J. Am. Chem. Soc.* **2019**, *141*, 7680–7686.

[6] G. S. Lee, D. Kim, S. H. Hong, *Nat. Commun.* **2021**, *12,* 991.

[7] D. K. Miller, *Tetrahedron Lett.* **2013**, *54*, 811–813.

[8] X. Ji, J. Guo, Y. Liu, A. Lu, Z. Wang, Y. Li, S. Yang, Q. Wang, *J. Agric. Food Chem.* **2018**, *66*, 4062–4072.

[9] M. Konstantinidou, F. Magari, F. Sutanto, J. Haupenthal, V. R. Jumde, M. Yagiz Ünver, A. Heine, C. J. Camacho, A. K. H. Hirsch, G. Klebe, A. Dömling, *ChemMedChem* **2020**, *15*, 680–684.

[10] Y. Li, A. Chao, F. F. Fleming, *Chem. Commun.* **2016**, *52*, 2111–2113.

[11] Q. Tong, R.-F. Xiu, J.-H. Chen, Y. Zhang, B.-D. Cui, N.-W. Wan, Y.-Z. Chen, W.-Y. Han, *ACS Catal.* **2023**, *13*, 12692−12699.

[12] X. Wang, J.-P. Fu, J.-X. Xie, Q.-H. Teng, H.-T. Tang, Y.-M. Pan, *Org. Biomol. Chem.* **2020**, *18*, 4936–4940.

[13] M. Rovira, M. Soler, I. Güell, M.-Z. Wang, L. Gómez, X. Ribas, *J. Org. Chem.* **2016**, *81*, 7315–7325.

[14] L. A. Perego, P. Fleurat-Lessard, L. El Kaïm, I. Ciofini, L. Grimaud, *Chem. - Eur. J.* **2016**, *22*, 15491–15500.

[15] D. Chowdhury, S. Ghosh, K.S.S.V. P. Reddy, S. S.R.K.C. Yamijala, M. Baidya, *ACS Catal.* **2023**, *13*, 12543–12552.

[16] J. Shen, Z. Xu, S. Yang, S. Li, J. Jiang, Y.-Q. Zhang, *J. Am. Chem. Soc.* **2023**, *145*, 21122–21131.

[17] F. Li, H.-M. Li, R.-F. Xiu, J.-K. Zhang, B.-D. Cui, N.-W. Wan, Y.-Z. Chen, W.-Y. Han,

Org. Lett. **2022**, *24*, 9392–9397.

[18] H.-R. Tong, S. Zheng, X. Li, Z. Deng, H. Wang, G. He, Q. Peng, G. Chen, *ACS Catal.* **2018**, *8*, 11502–11512.

11. Copies of ^{1}H-NMR, ^{13}C-NMR and ^{19}F-NMR spectra

CJH-1-37-1-H

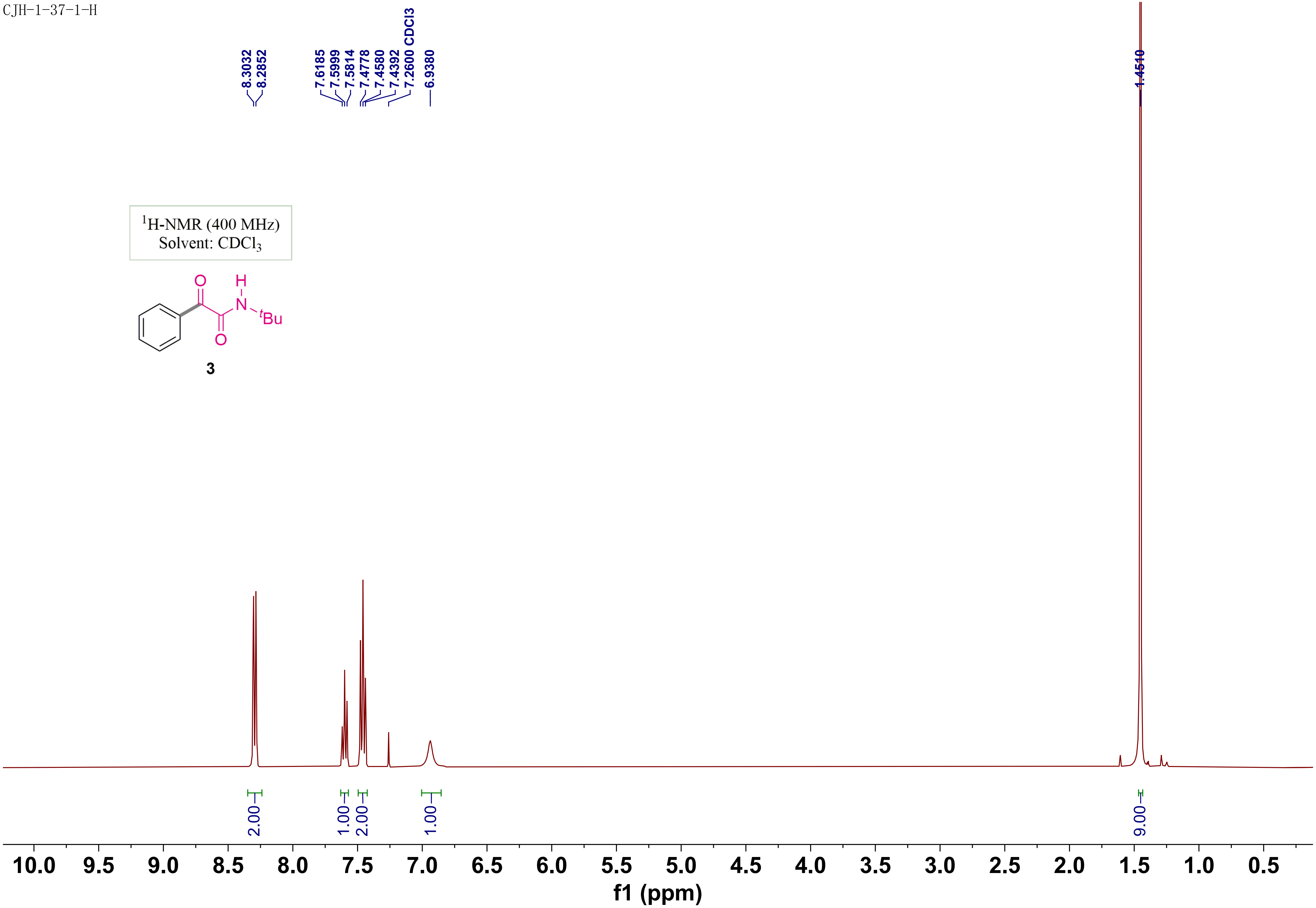

CJH-1-37-1-C

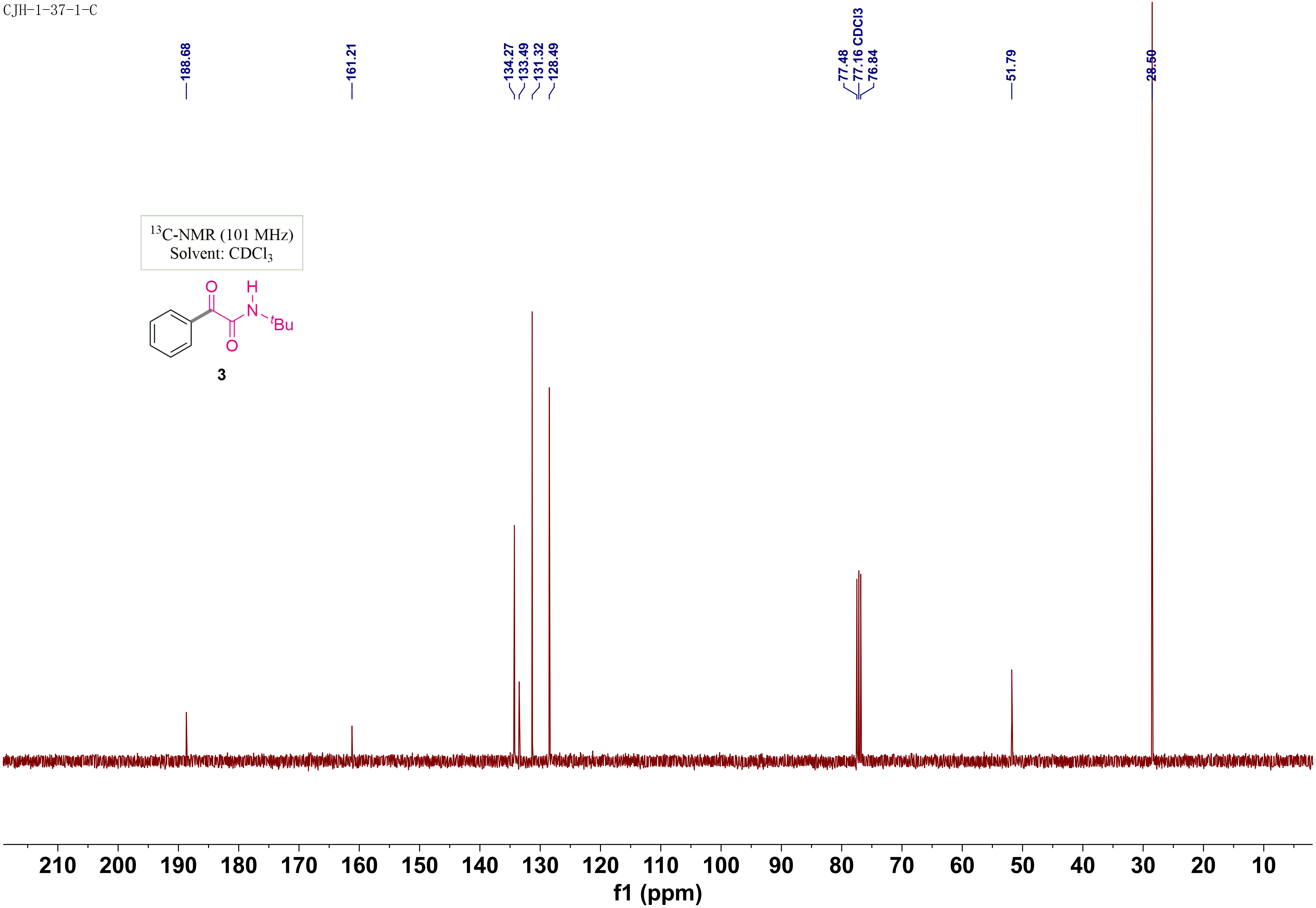
188.68
161.21
134.27
133.49
131.32
128.49
77.48
77.16 CDCl3
76.84
51.79
28.50
^{13}C-NMR (101 MHz)
Solvent: $CDCl_3$
O
H
N
tBu
O
3
210
200
190
180
170
160
150
140
130
120
110
100
90
80
70
60
50
40
30
20
10
f1 (ppm)

CJH-1-38-1-H

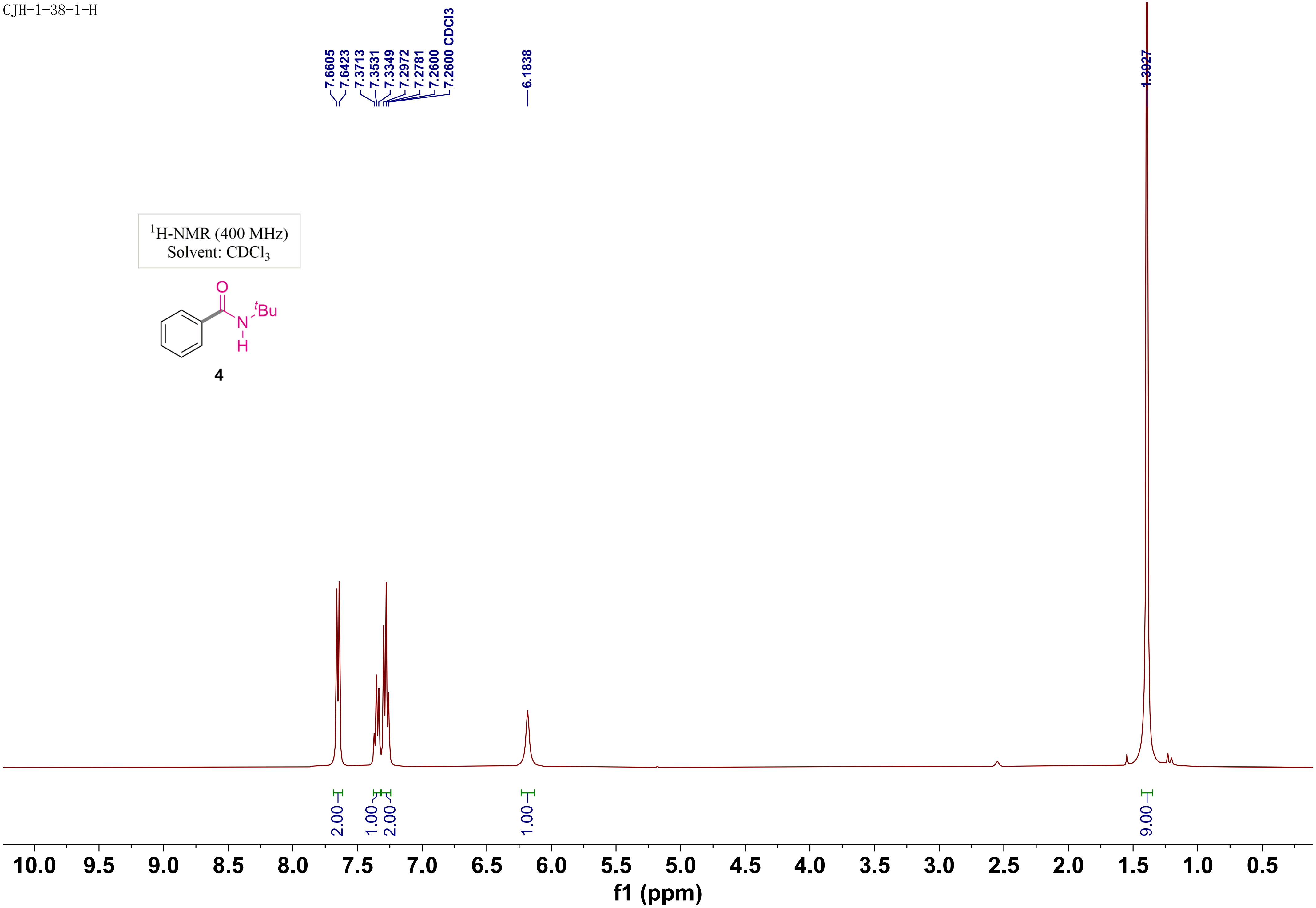

^{1}H-NMR (400 MHz)
Solvent: $CDCl_3$

4

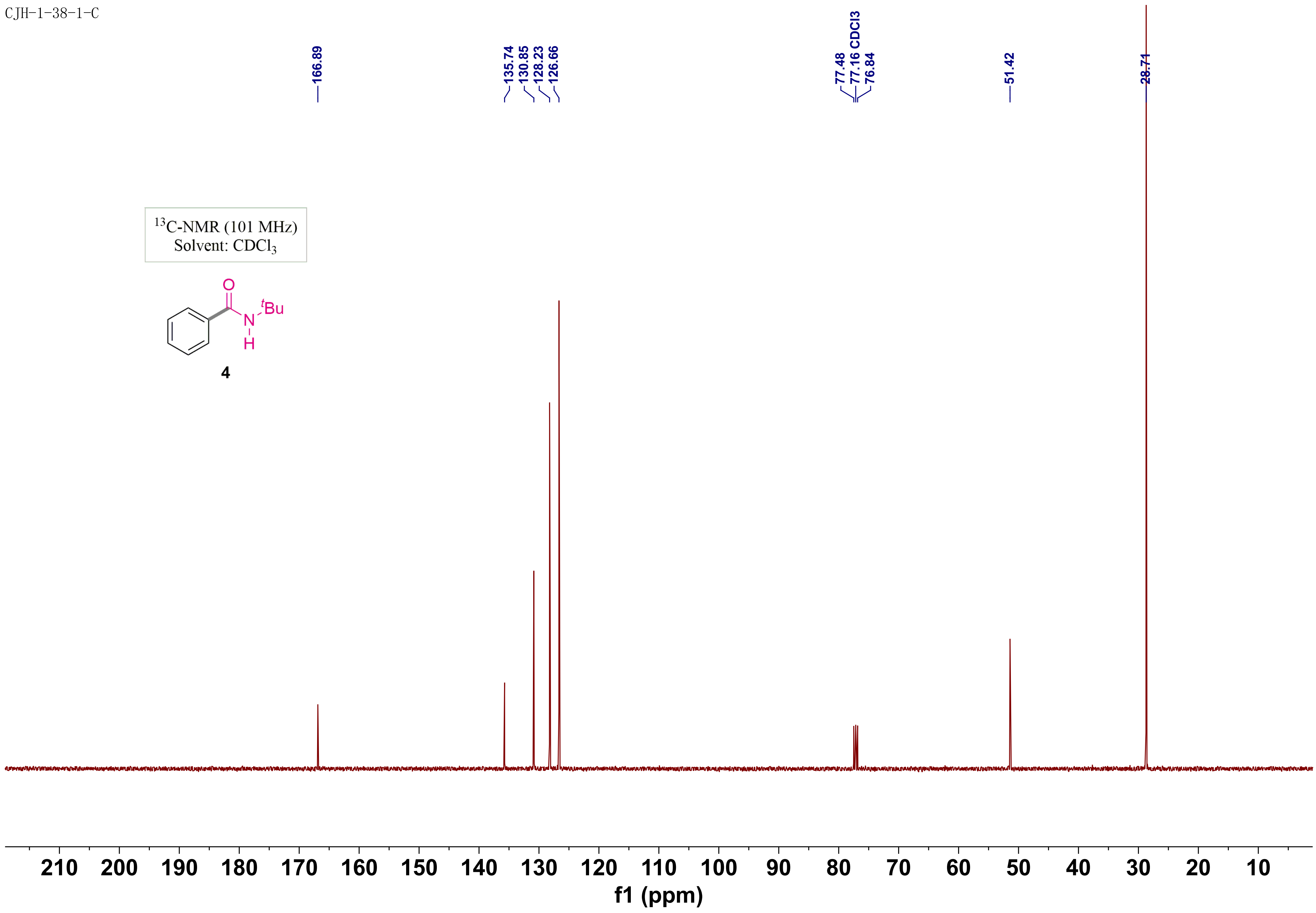
CJH-1-38-1-C
166.89
135.74
130.85
128.23
126.66
77.48
77.16 CDCl3
76.84
51.42
28.71
^{13}C-NMR (101 MHz)
Solvent: $CDCl_3$
O
^{t}Bu
N
H
4
210 200 190 180 170 160 150 140 130 120 110 100 90 80 70 60 50 40 30 20 10
f1 (ppm)

CJH-1-102-3-H

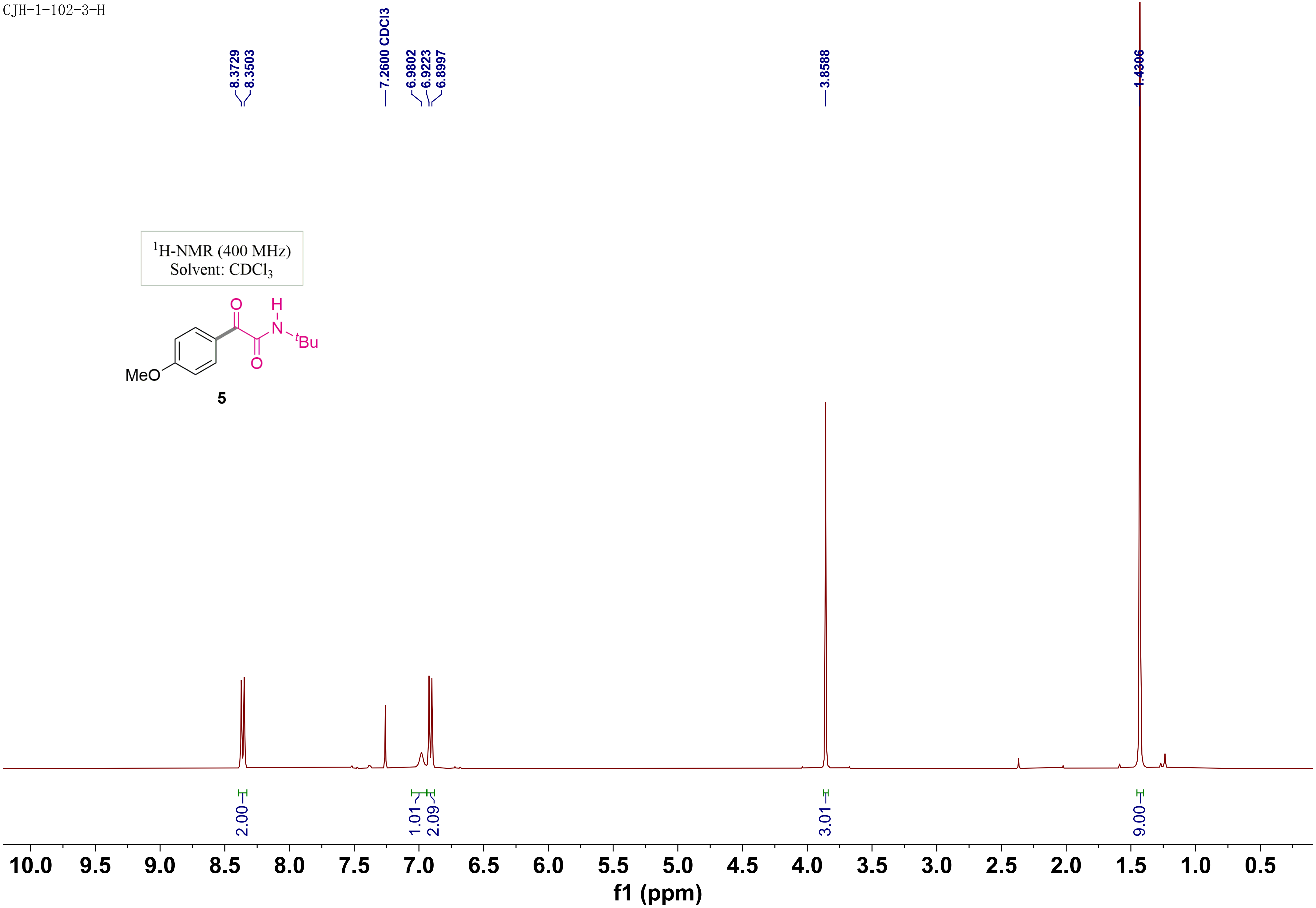

8.3729
8.3503
7.2600 CDCl3
6.9802
6.9223
6.8997
3.8588
1.4306
^{1}H-NMR (400 MHz)
Solvent: $CDCl_3$
O
H
N
tBu
O
MeO
5
2.00
1.01
2.09
3.01
9.00
10.0
9.5
9.0
8.5
8.0
7.5
7.0
6.5
6.0
5.5
5.0
4.5
4.0
3.5
3.0
2.5
2.0
1.5
1.0
0.5
f1 (ppm)

CJH-1-102-3-C

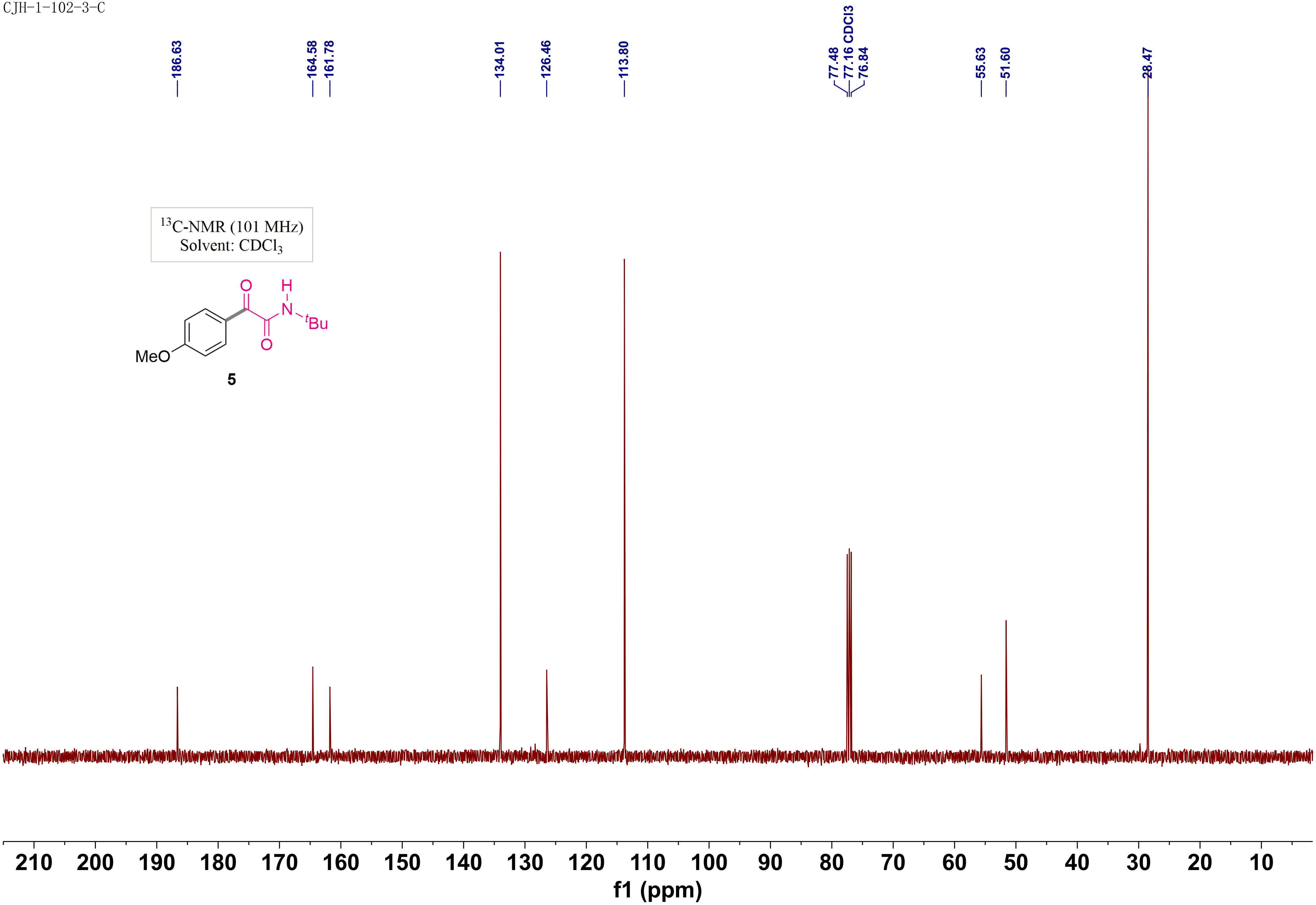
186.63
164.58
161.78
134.01
126.46
113.80
77.48
77.16 CDCl3
76.84
55.63
51.60
28.47
^{13}C-NMR (101 MHz)
Solvent: $CDCl_3$
MeO
O
O
H
N
^{t}Bu
5
210
200
190
180
170
160
150
140
130
120
110
100
90
80
70
60
50
40
30
20
10
f1 (ppm)

CJH-1-103-1-H

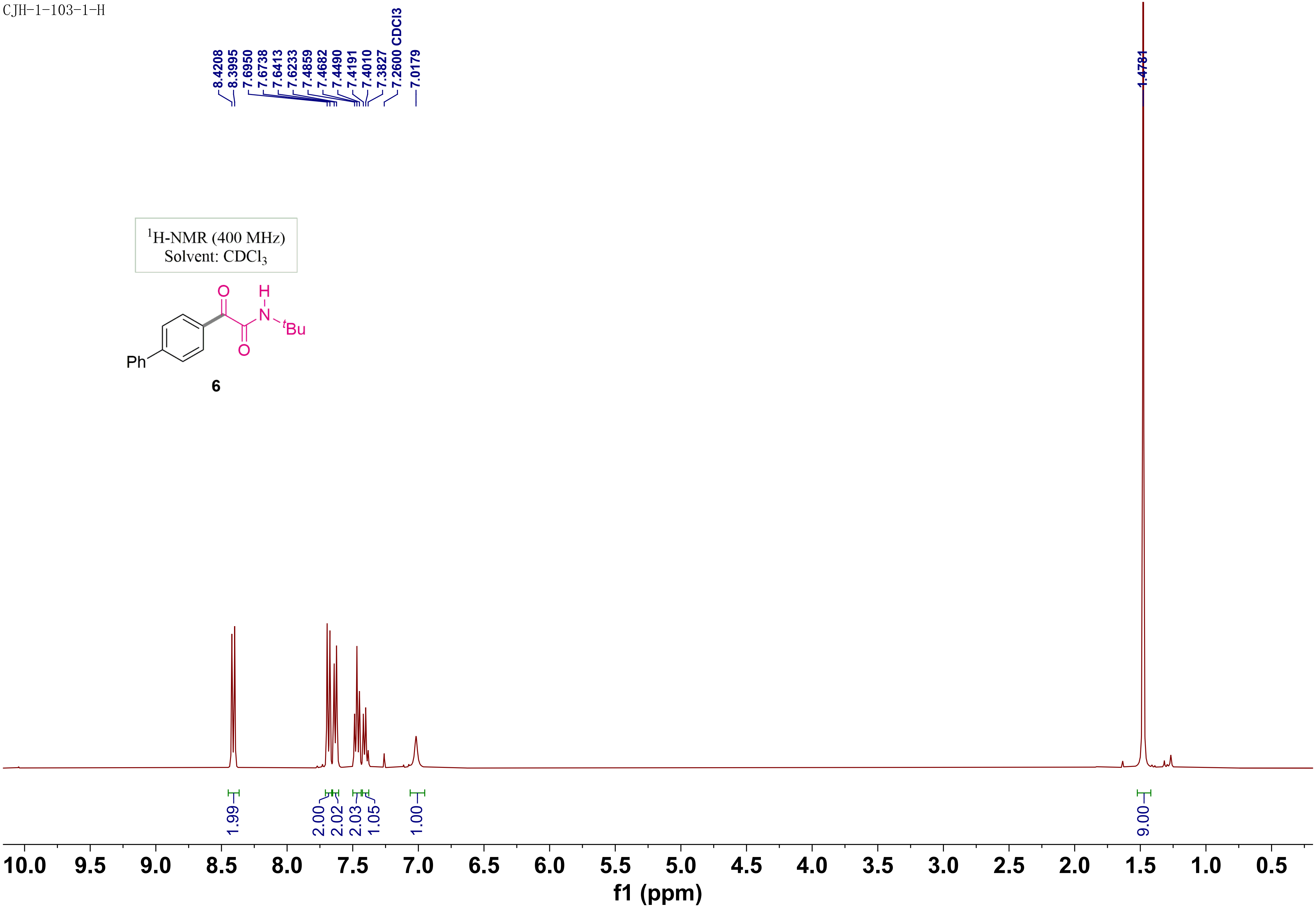

8.4208
8.3995
7.6950
7.6738
7.6413
7.6233
7.4859
7.4682
7.4490
7.4191
7.4010
7.3827
7.2600 CDCl3
7.0179
1.4781
^{1}H-NMR (400 MHz)
Solvent: $CDCl_3$
O
H
N
tBu
O
Ph
6
1.99
2.00
2.02
2.03
1.05
1.00
9.00
10.0 9.5 9.0 8.5 8.0 7.5 7.0 6.5 6.0 5.5 5.0 4.5 4.0 3.5 3.0 2.5 2.0 1.5 1.0 0.5
f1 (ppm)

CJH-1-103-1-C

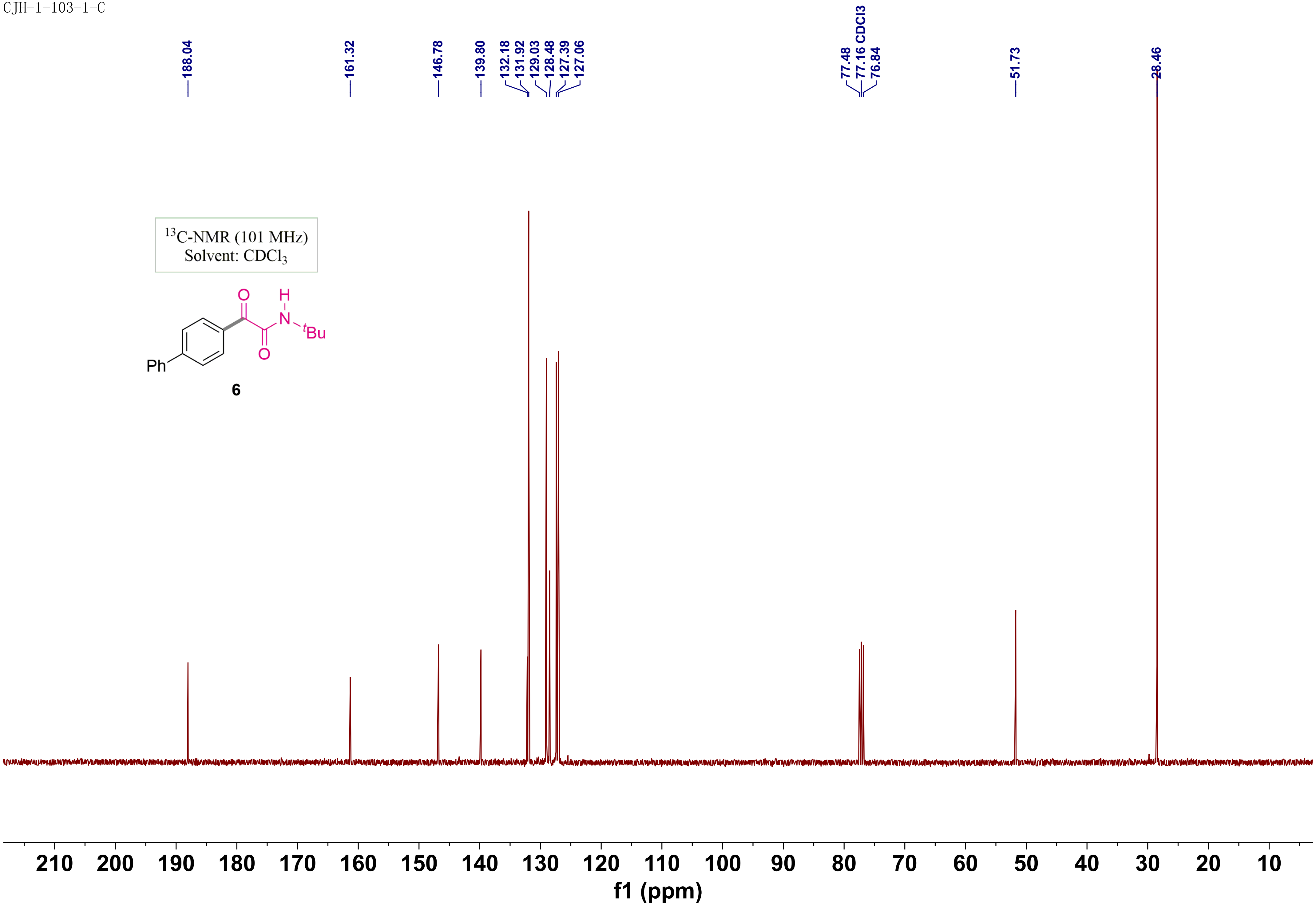
188.04
161.32
146.78
139.80
132.18
131.92
129.03
128.48
127.39
127.06
77.48
77.16 CDCl3
76.84
51.73
28.46
^{13}C-NMR (101 MHz)
Solvent: $CDCl_3$
O
H
N
^{t}Bu
O
Ph
6
210
200
190
180
170
160
150
140
130
120
110
100
90
80
70
60
50
40
30
20
10
f1 (ppm)

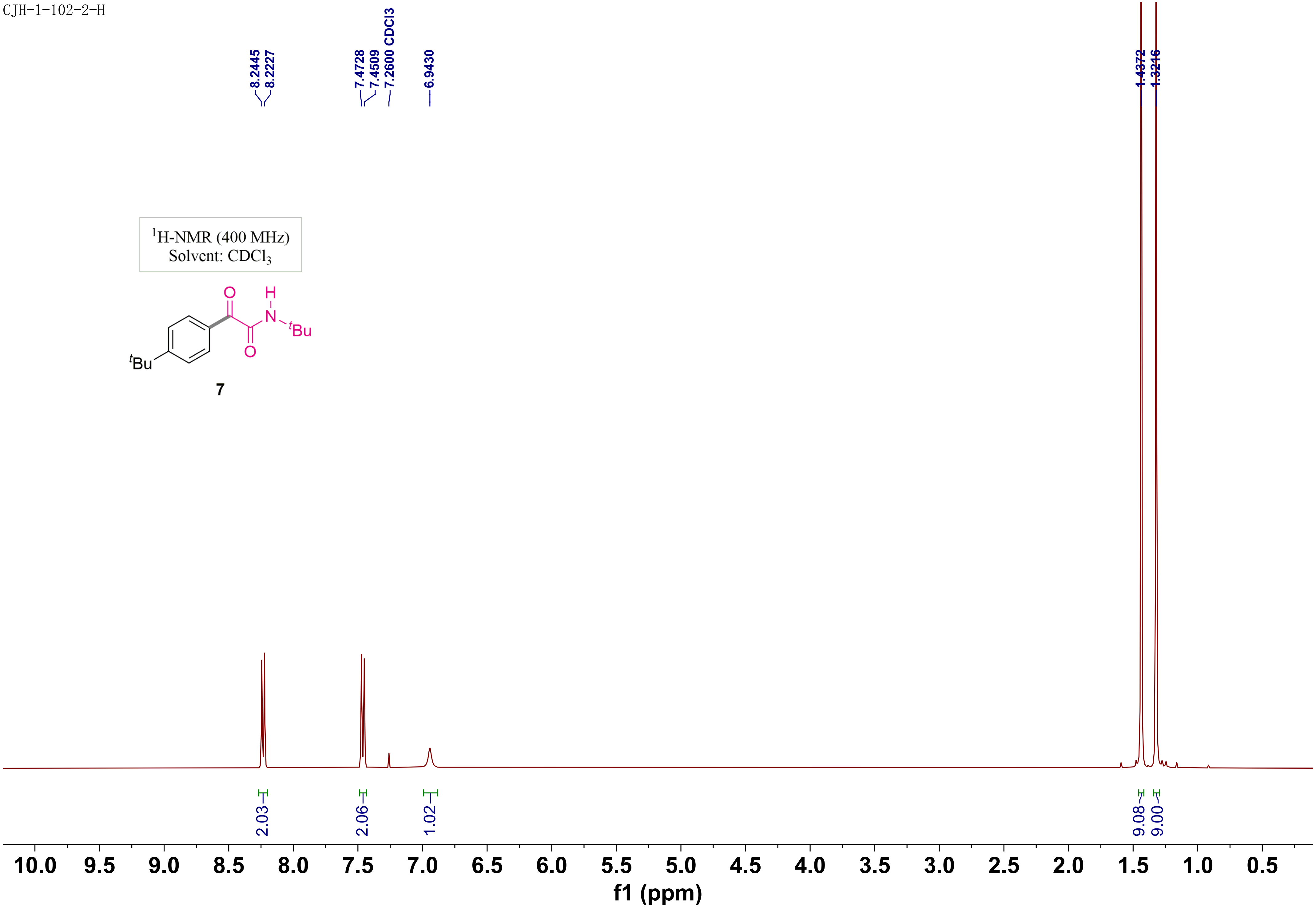
CJH-1-102-2-H
8.2445
8.2227
7.4728
7.4509
7.2600 CDCl3
6.9430
1.4372
1.3216
¹H-NMR (400 MHz)
Solvent: CDCl3
O
H
N
tBu
O
tBu
7
2.03
2.06
1.02
9.08
9.00
10.0
9.5
9.0
8.5
8.0
7.5
7.0
6.5
6.0
5.5
5.0
4.5
4.0
3.5
3.0
2.5
2.0
1.5
1.0
0.5
f1 (ppm)

CJH-1-102-2-C

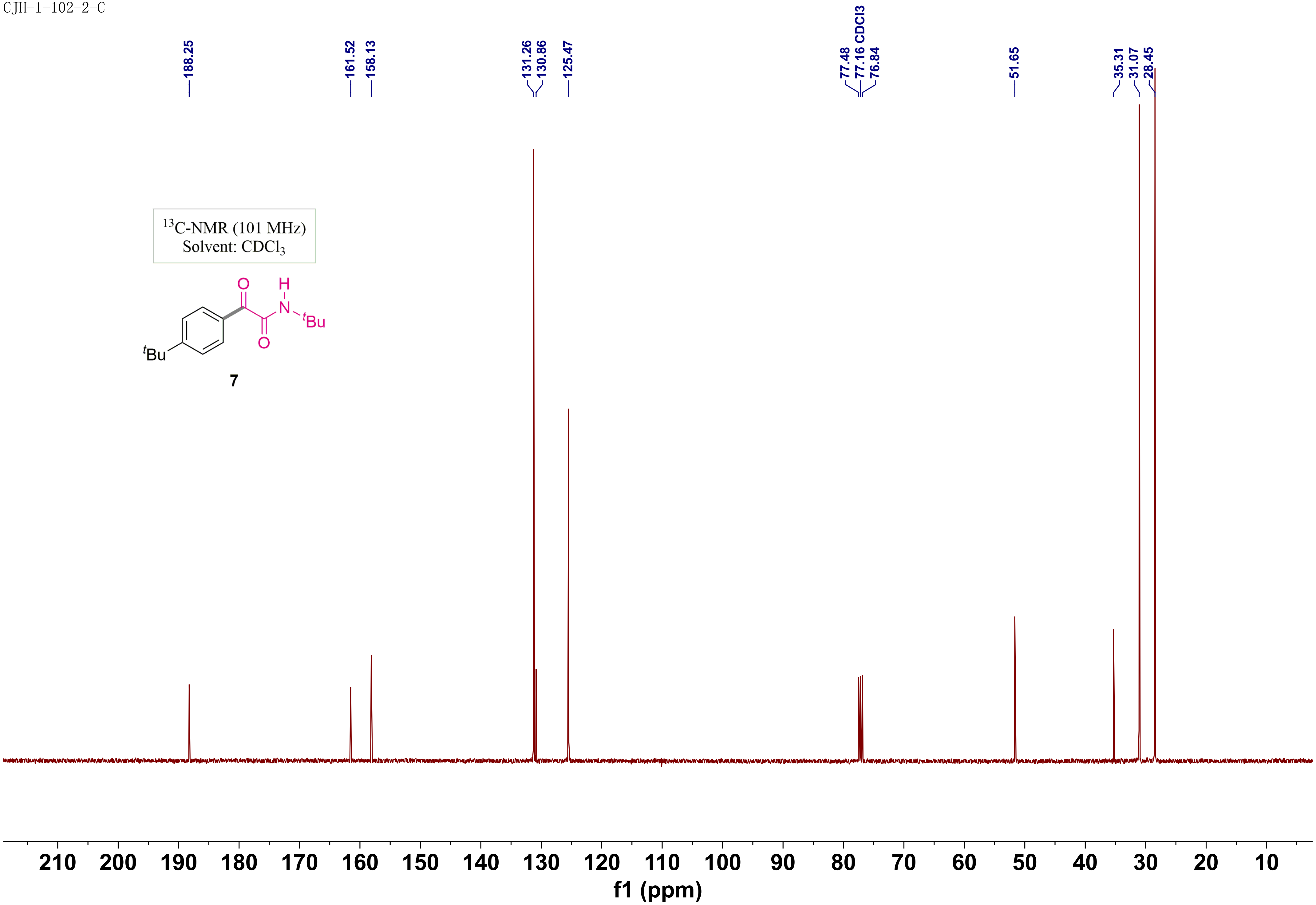

188.25
161.52
158.13
131.26
130.86
125.47
77.48
77.16 CDCl3
76.84
51.65
35.31
31.07
28.45
^{13}C-NMR (101 MHz)
Solvent: $CDCl_3$
O
H
N
tBu
O
tBu
7
210 200 190 180 170 160 150 140 130 120 110 100 90 80 70 60 50 40 30 20 10
f1 (ppm)

CJH-2-61-3-H

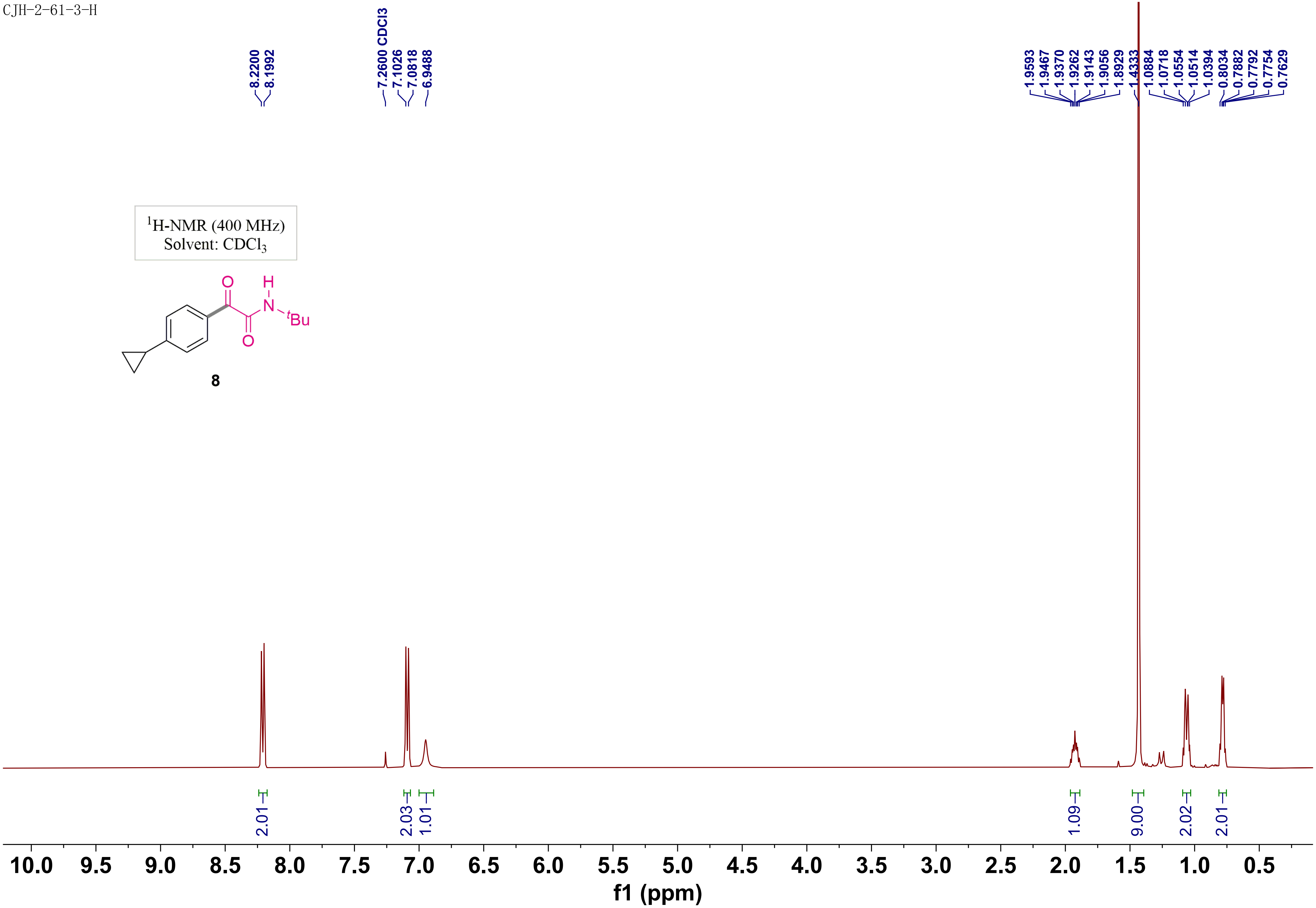
8.2200
8.1992
7.2600 CDCl3
7.1026
7.0818
6.9488
1.9593
1.9467
1.9370
1.9262
1.9143
1.9056
1.8929
1.4333
1.0884
1.0718
1.0554
1.0514
1.0394
0.8034
0.7882
0.7792
0.7754
0.7629
1H-NMR (400 MHz)
Solvent: CDCl3
O
H
N
tBu
O
8
2.01
2.03
1.01
1.09
9.00
2.02
2.01
10.0 9.5 9.0 8.5 8.0 7.5 7.0 6.5 6.0 5.5 5.0 4.5 4.0 3.5 3.0 2.5 2.0 1.5 1.0 0.5
f1 (ppm)

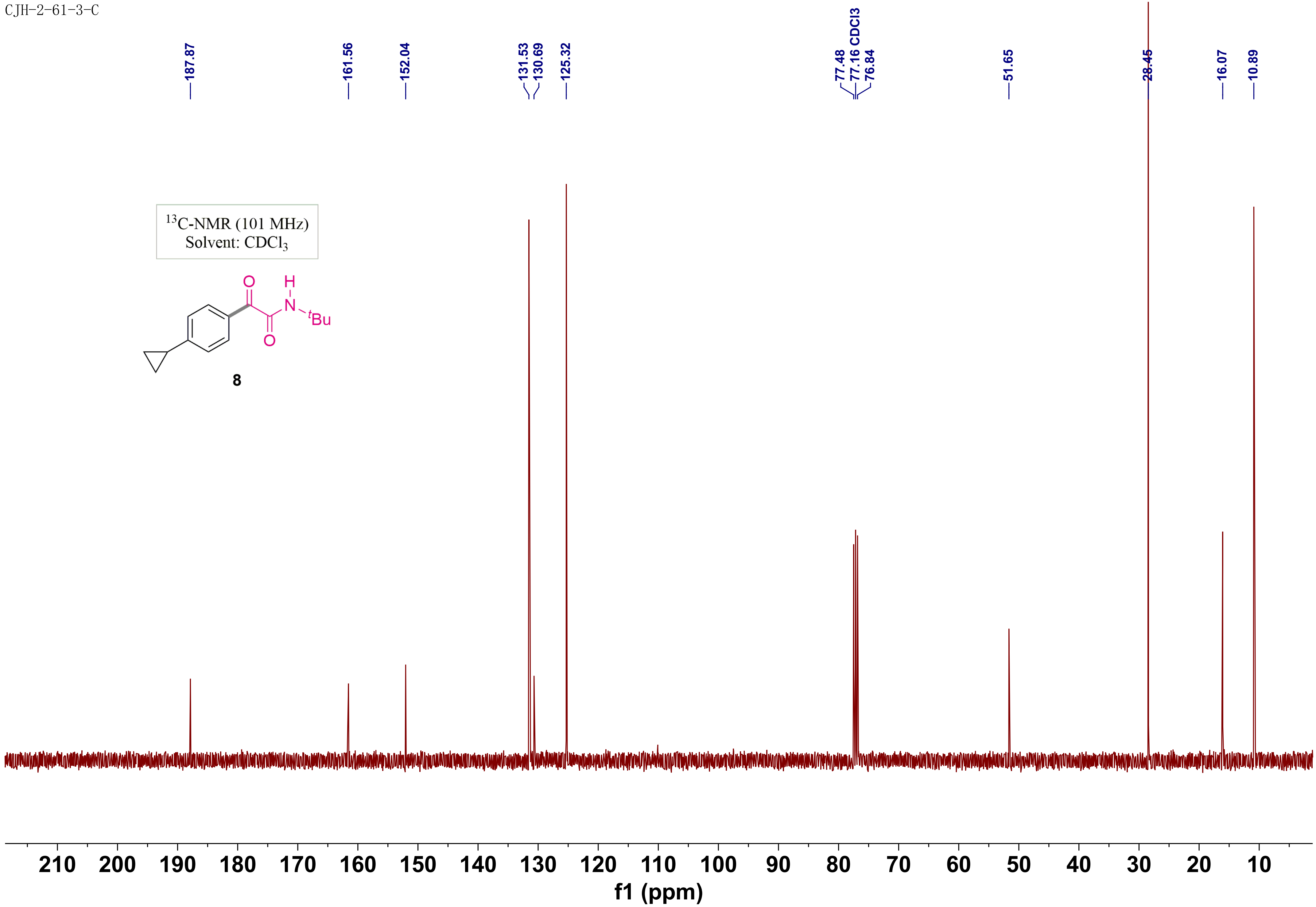
CJH-2-61-3-C
187.87
161.56
152.04
131.53
130.69
125.32
77.48
77.16 CDCl3
76.84
51.65
28.45
16.07
10.89
^{13}C-NMR (101 MHz)
Solvent: $CDCl_3$
O
H
N
^{t}Bu
O
8
210 200 190 180 170 160 150 140 130 120 110 100 90 80 70 60 50 40 30 20 10
f1 (ppm)

CJH-1-109-1-H

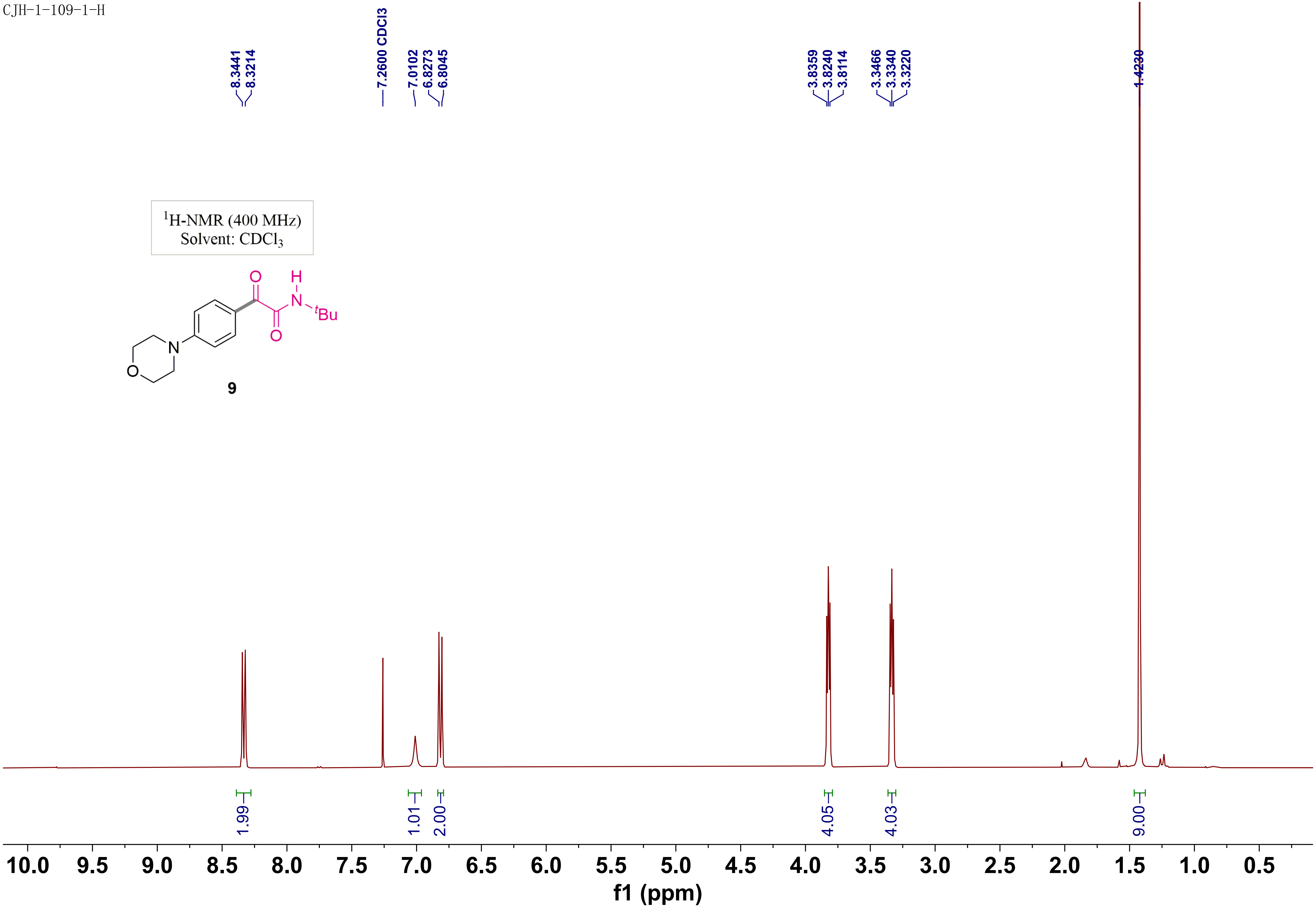

8.3441
8.3214
7.2600 CDCl3
7.0102
6.8273
6.8045
3.8359
3.8240
3.8114
3.3466
3.3340
3.3220
1.4230
^{1}H-NMR (400 MHz)
Solvent: $CDCl_3$
9
1.99
1.01
2.00
4.05
4.03
9.00
10.0 9.5 9.0 8.5 8.0 7.5 7.0 6.5 6.0 5.5 5.0 4.5 4.0 3.5 3.0 2.5 2.0 1.5 1.0 0.5
f1 (ppm)

CJH-1-109-1-C

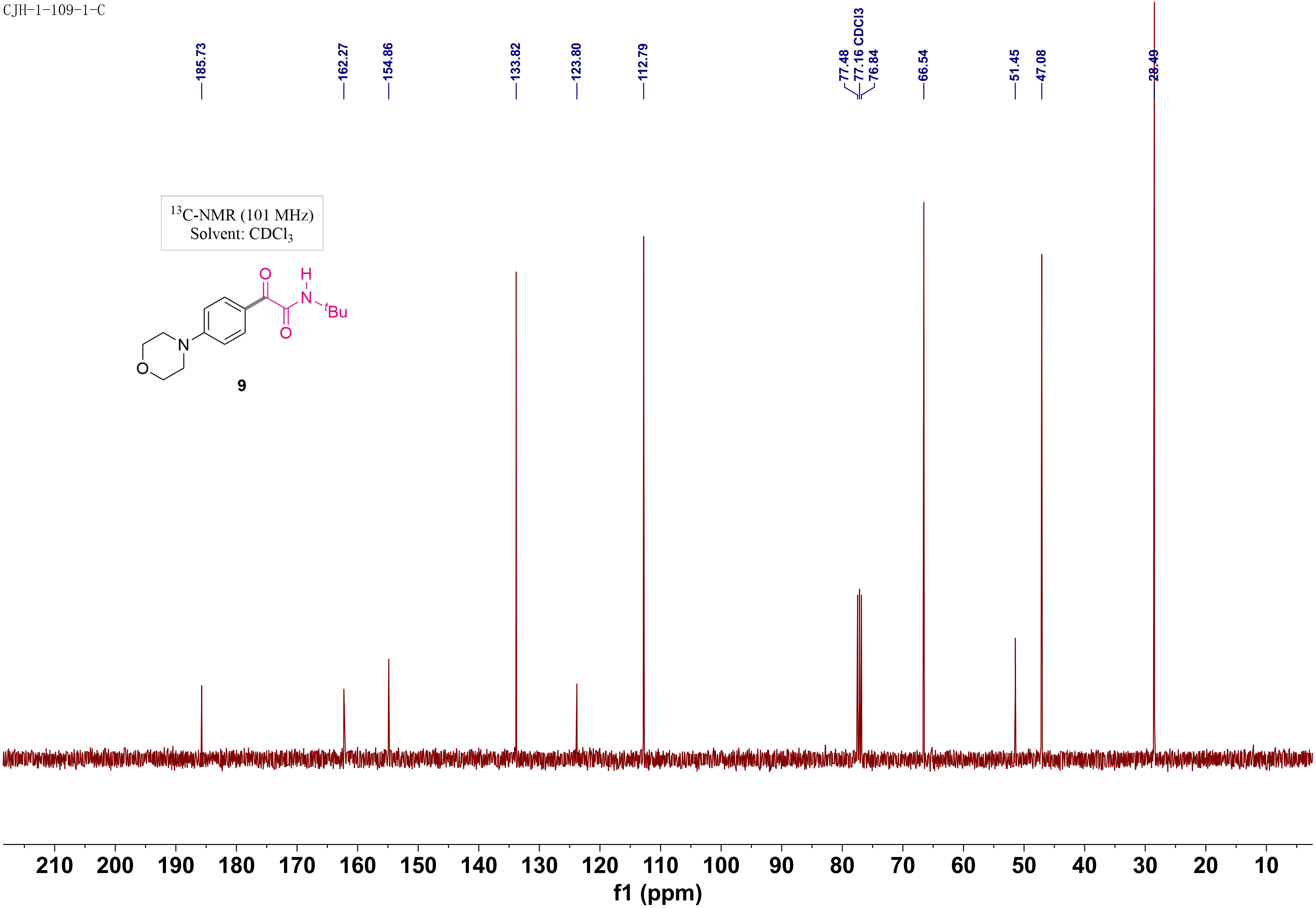

185.73
162.27
154.86
133.82
123.80
112.79
77.48
77.16 CDCl3
76.84
66.54
51.45
47.08
28.49
^{13}C-NMR (101 MHz)
Solvent: $CDCl_3$
O
H
N
tBu
O
N
O
9
210
200
190
180
170
160
150
140
130
120
110
100
90
80
70
60
50
40
30
20
10
f1 (ppm)

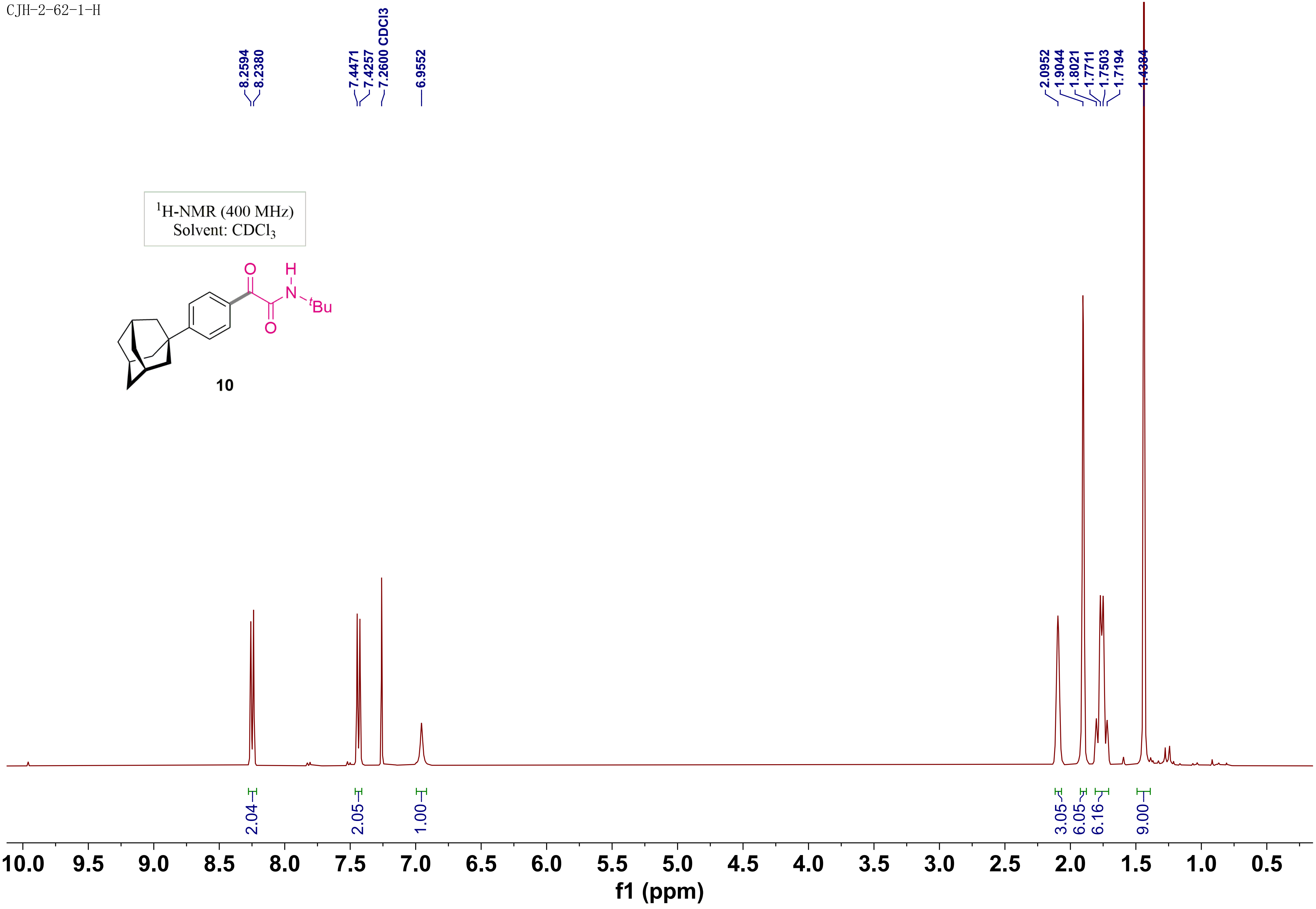
CJH-2-62-1-H
8.2594
8.2380
7.4471
7.4257
7.2600 CDCl3
6.9552
2.0952
1.9044
1.8021
1.7711
1.7503
1.7194
1.4384
^{1}H-NMR (400 MHz)
Solvent: $CDCl_3$
O
H
N
^{t}Bu
O
10
2.04
2.05
1.00
3.05
6.05
6.16
9.00
10.0 9.5 9.0 8.5 8.0 7.5 7.0 6.5 6.0 5.5 5.0 4.5 4.0 3.5 3.0 2.5 2.0 1.5 1.0 0.5
f1 (ppm)

CJH-2-62-1-C

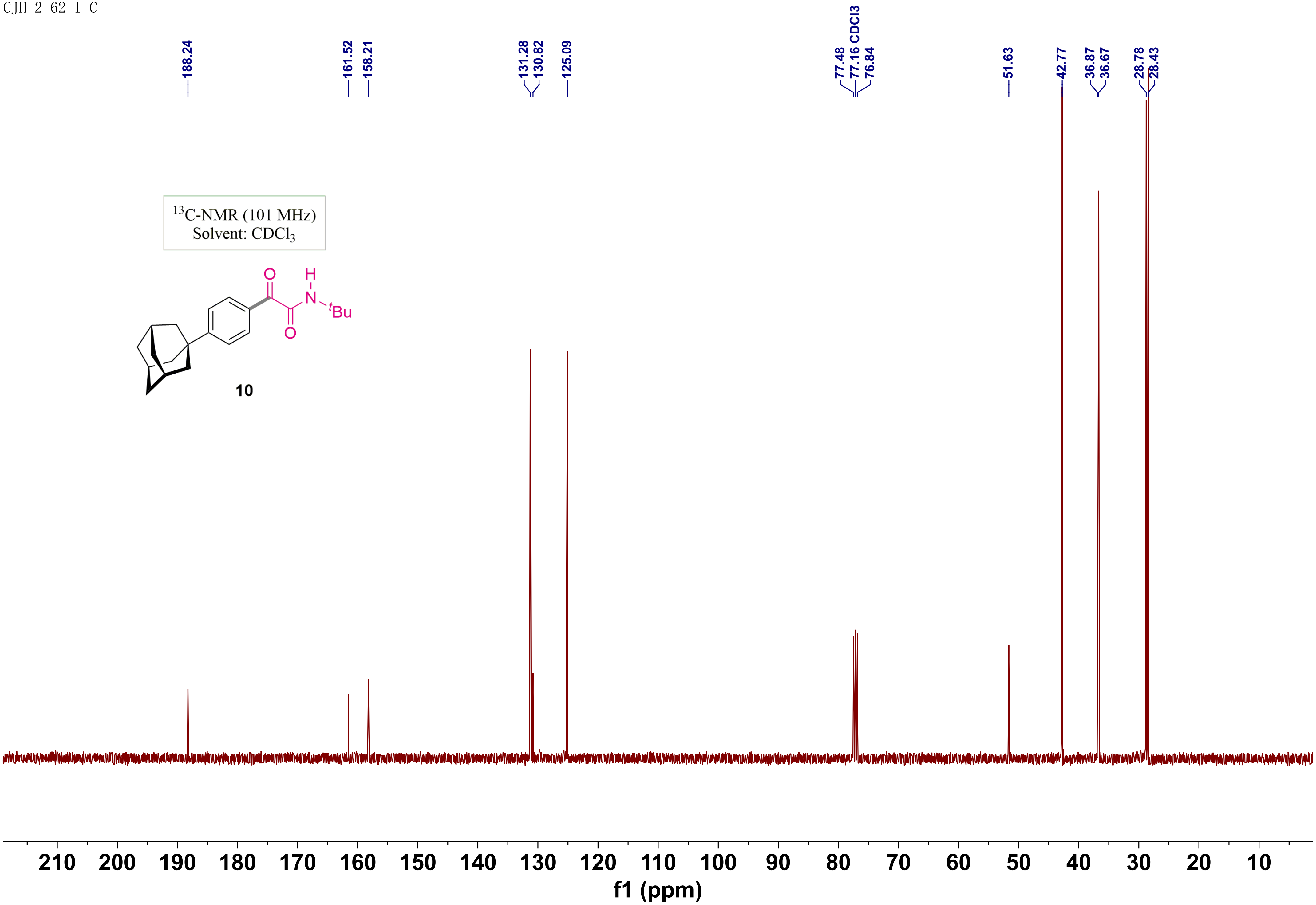
188.24
161.52
158.21
131.28
130.82
125.09
77.48
77.16 CDCl3
76.84
51.63
42.77
36.87
36.67
28.78
28.43
13C-NMR (101 MHz)
Solvent: CDCl3
O
H
N
tBu
O
10
210
200
190
180
170
160
150
140
130
120
110
100
90
80
70
60
50
40
30
20
10
f1 (ppm)

CJH-3-55-1-H

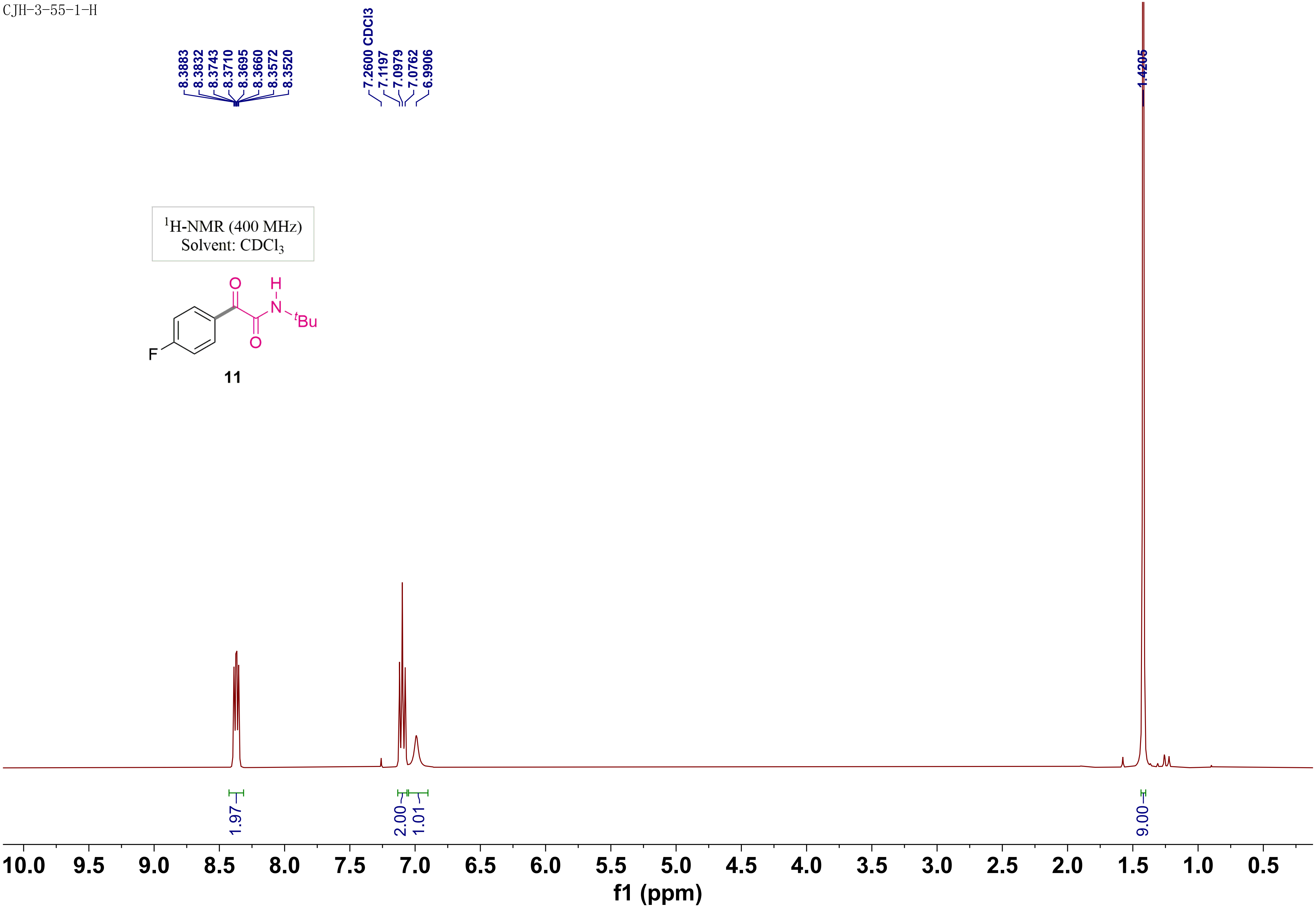

8.3883
8.3832
8.3743
8.3710
8.3695
8.3660
8.3572
8.3520
7.2600 CDCl3
7.1197
7.0979
7.0762
6.9906
1.4205
^{1}H-NMR (400 MHz)
Solvent: $CDCl_3$
O
H
N
tBu
O
F
11
1.97
2.00
1.01
9.00
10.0 9.5 9.0 8.5 8.0 7.5 7.0 6.5 6.0 5.5 5.0 4.5 4.0 3.5 3.0 2.5 2.0 1.5 1.0 0.5
f1 (ppm)

CJH-3-55-1-C

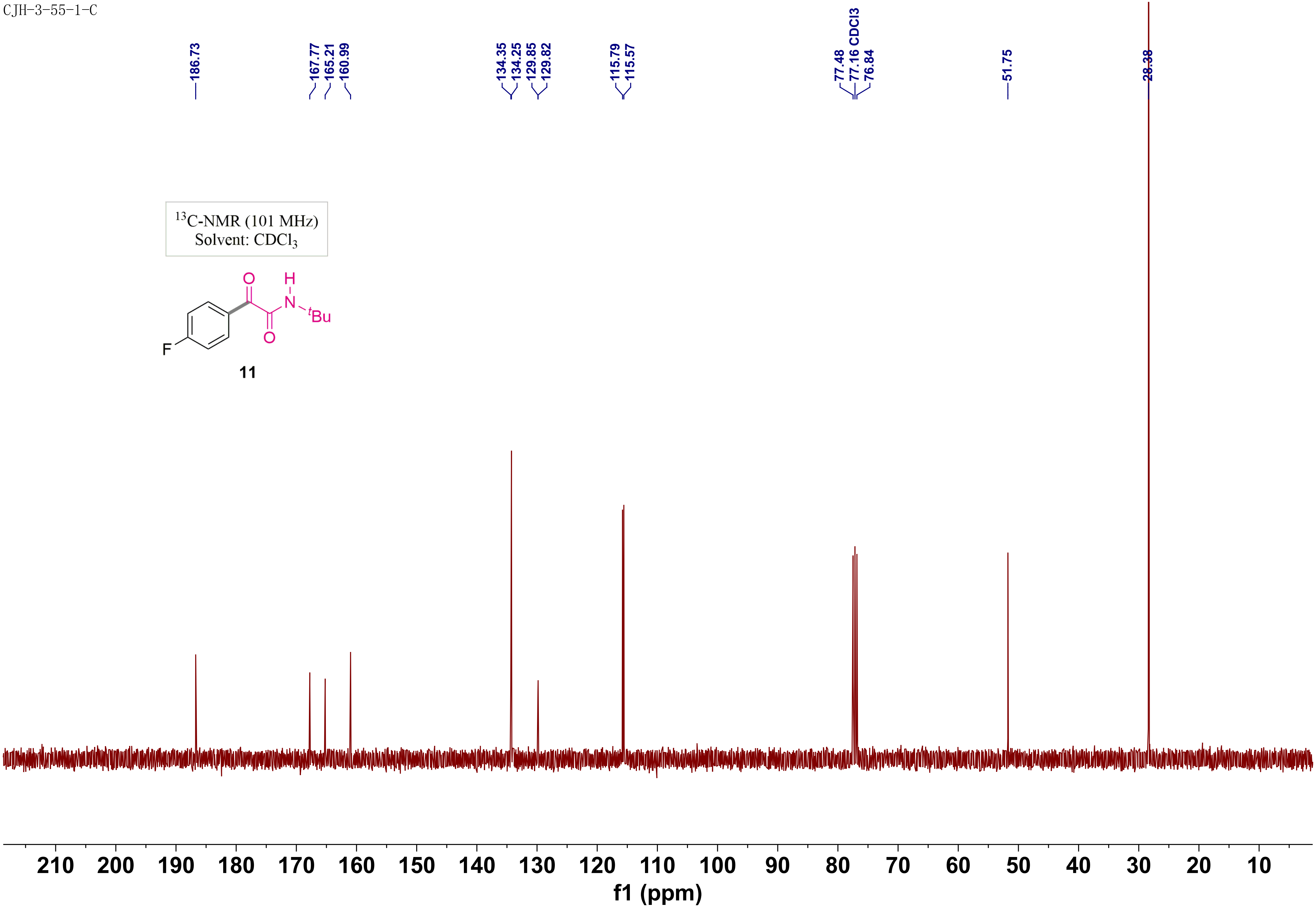

186.73
167.77
165.21
160.99
134.35
134.25
129.85
129.82
115.79
115.57
77.48
77.16 CDCl3
76.84
51.75
28.38
^{13}C-NMR (101 MHz)
Solvent: $CDCl_3$
O
H
N
tBu
O
F
11
210
200
190
180
170
160
150
140
130
120
110
100
90
80
70
60
50
40
30
20
10
f1 (ppm)

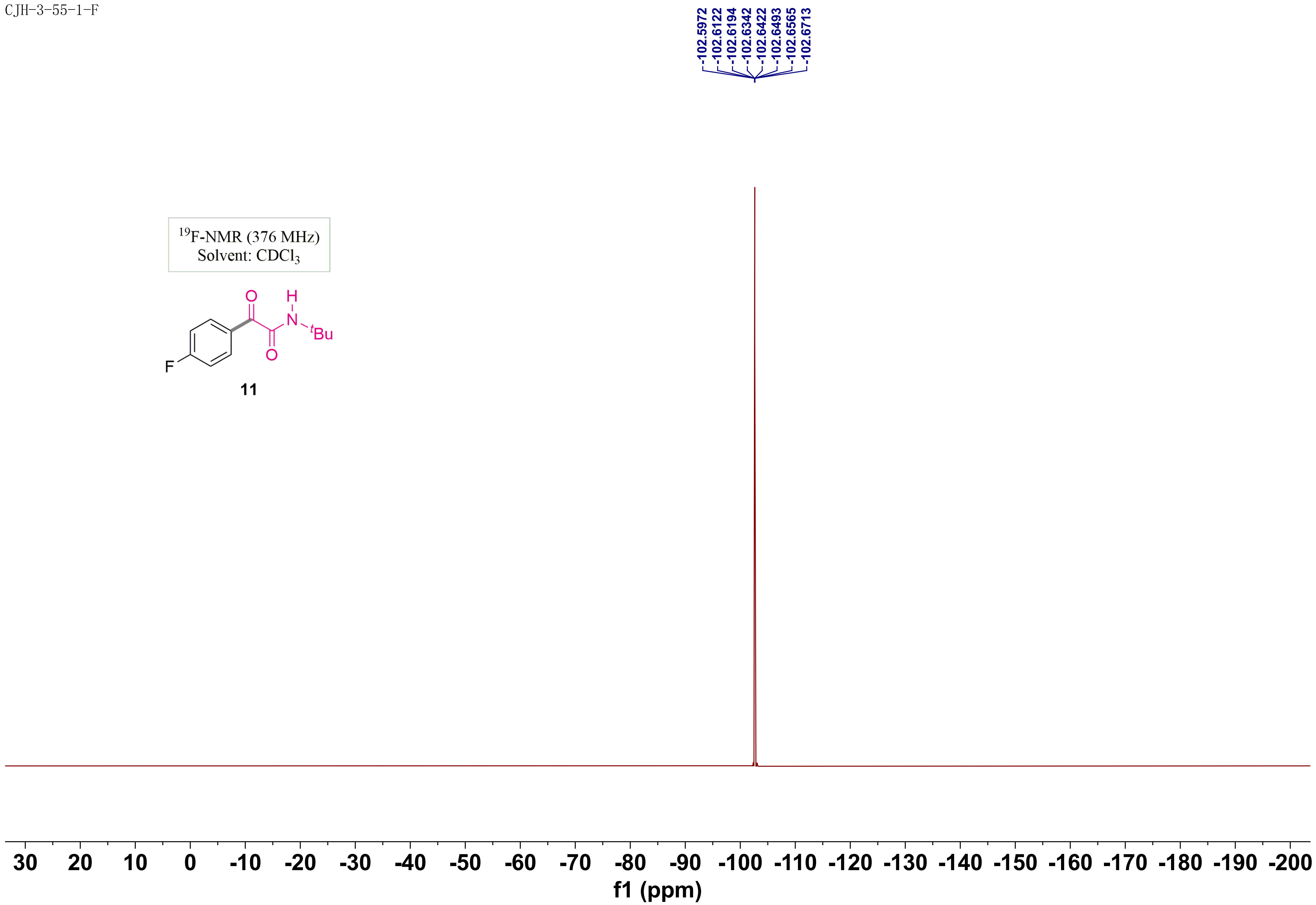
CJH-3-55-1-F
-102.5972
-102.6122
-102.6194
-102.6342
-102.6422
-102.6493
-102.6565
-102.6713
19F-NMR (376 MHz)
Solvent: CDCl3
O
H
N
tBu
O
F
11
30 20 10 0 -10 -20 -30 -40 -50 -60 -70 -80 -90 -100 -110 -120 -130 -140 -150 -160 -170 -180 -190 -200
f1 (ppm)

CJH-1-103-2-H

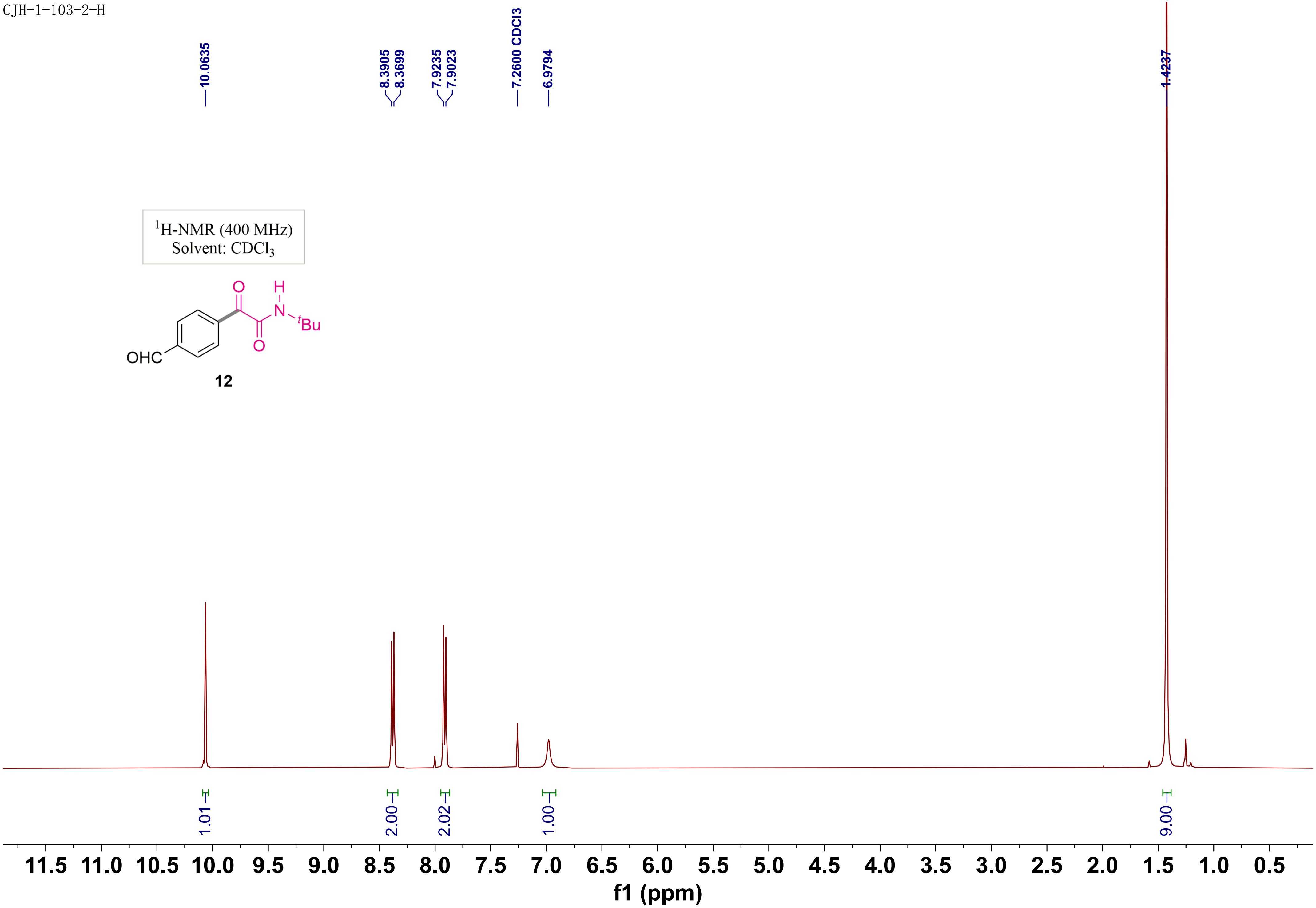

12

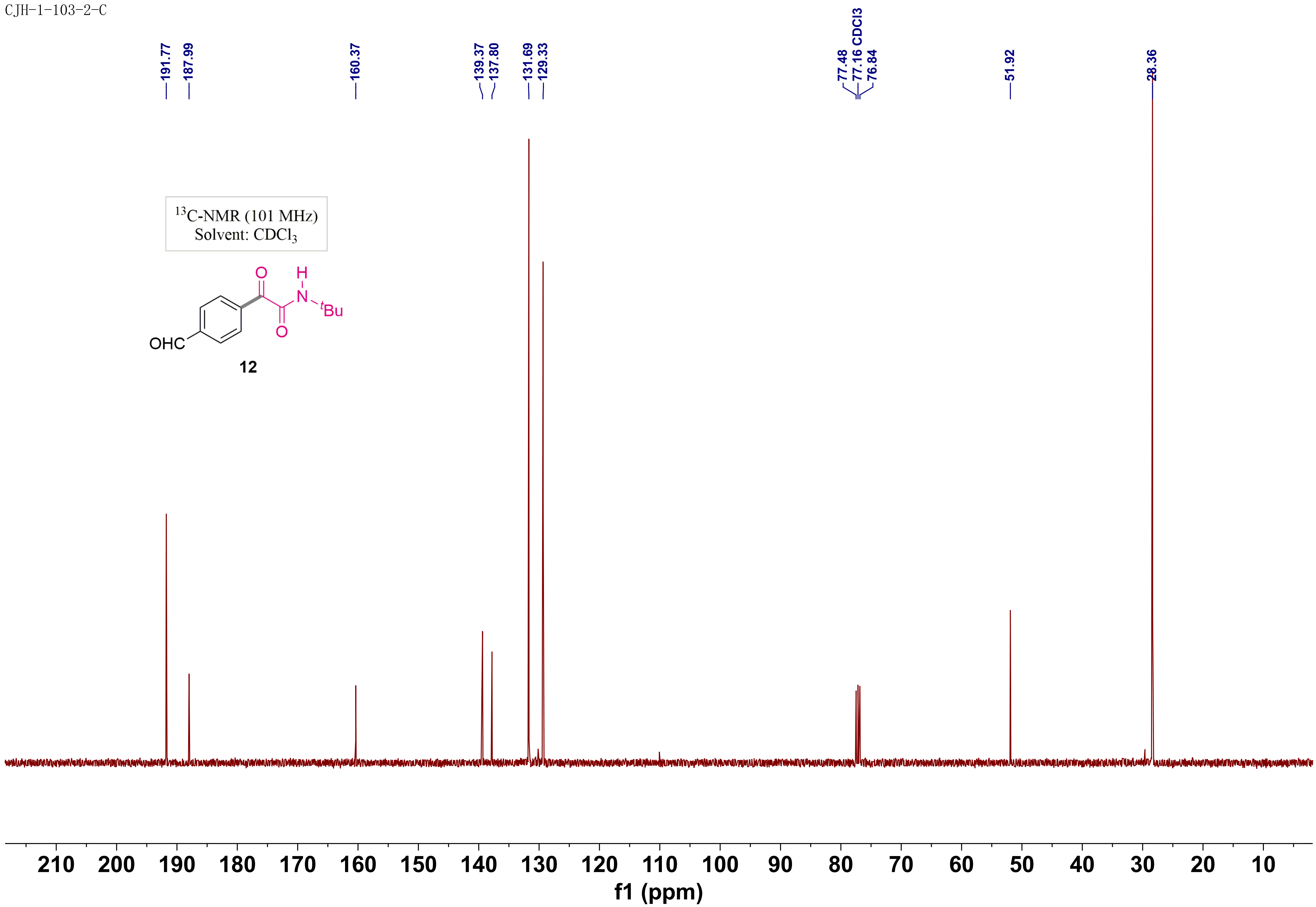
CJH-1-103-2-C
191.77
187.99
160.37
139.37
137.80
131.69
129.33
77.48
77.16 CDCl3
76.84
51.92
28.36
13C-NMR (101 MHz)
Solvent: CDCl3
O
H
N
tBu
O
OHC
12
210
200
190
180
170
160
150
140
130
120
110
100
90
80
70
60
50
40
30
20
10
f1 (ppm)

CJH-1-103-3-H

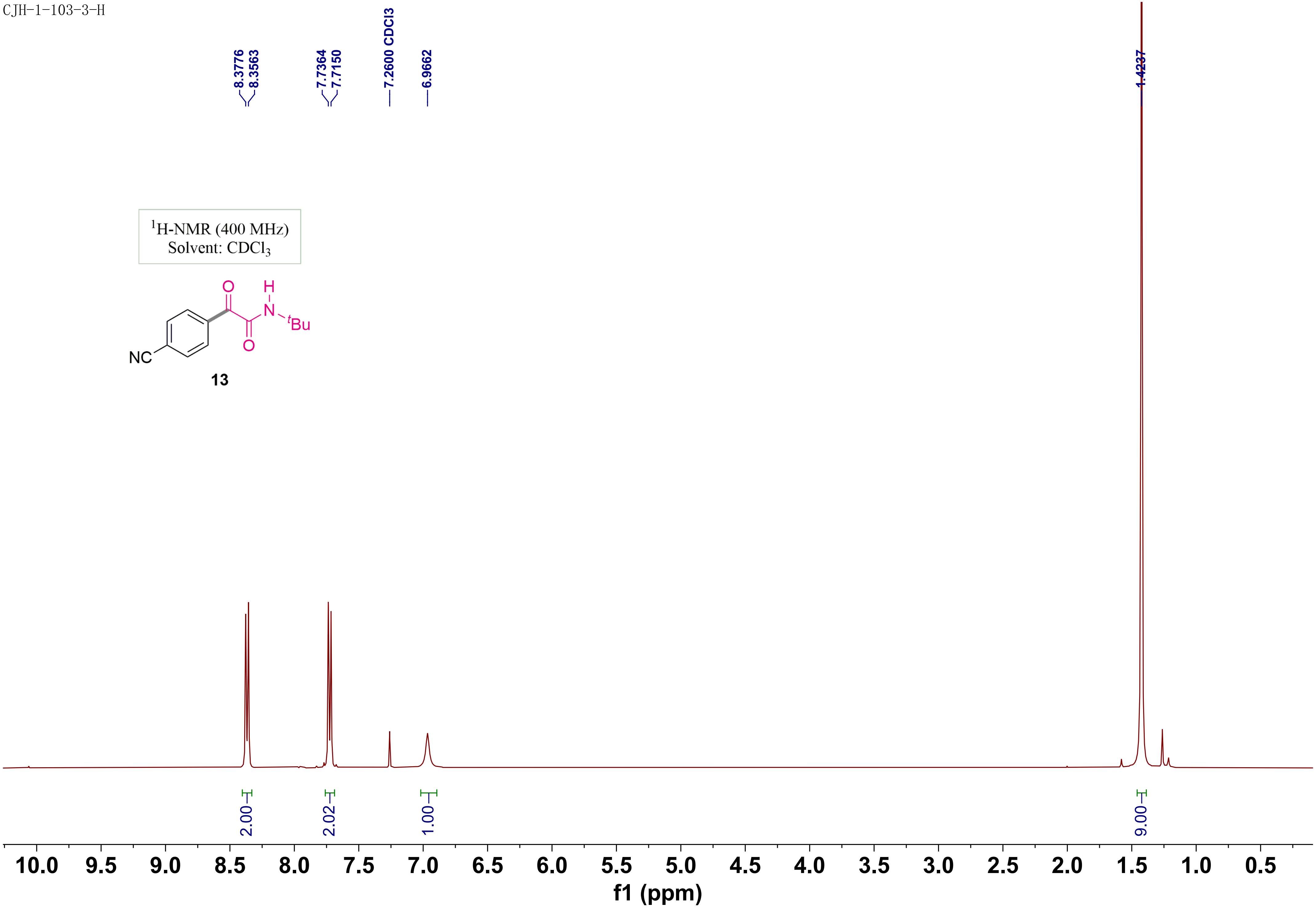
8.3776
8.3563
7.7364
7.7150
7.2600 CDCl3
6.9662
1.4237
^{1}H-NMR (400 MHz)
Solvent: CDCl3
NC
O
H
N
tBu
O
13
2.00
2.02
1.00
9.00
10.0 9.5 9.0 8.5 8.0 7.5 7.0 6.5 6.0 5.5 5.0 4.5 4.0 3.5 3.0 2.5 2.0 1.5 1.0 0.5
f1 (ppm)

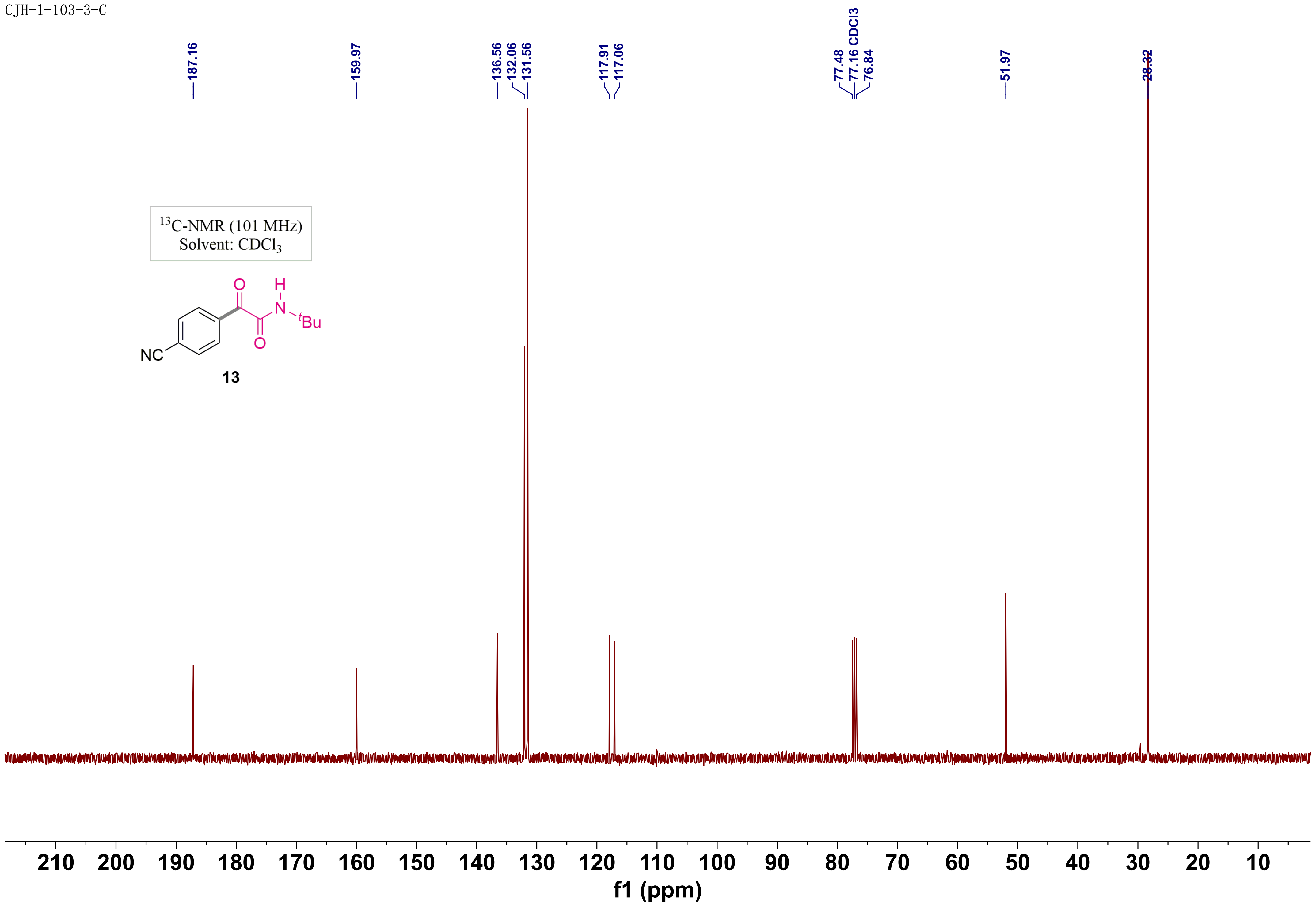

CJH-1-103-3-C
187.16
159.97
136.56
132.06
131.56
117.91
117.06
77.48
77.16 CDCl3
76.84
51.97
28.32
13C-NMR (101 MHz)
Solvent: CDCl3
NC
O
H
N
tBu
O
13
210
200
190
180
170
160
150
140
130
120
110
100
90
80
70
60
50
40
30
20
10
f1 (ppm)

CJH-1-107-1-H

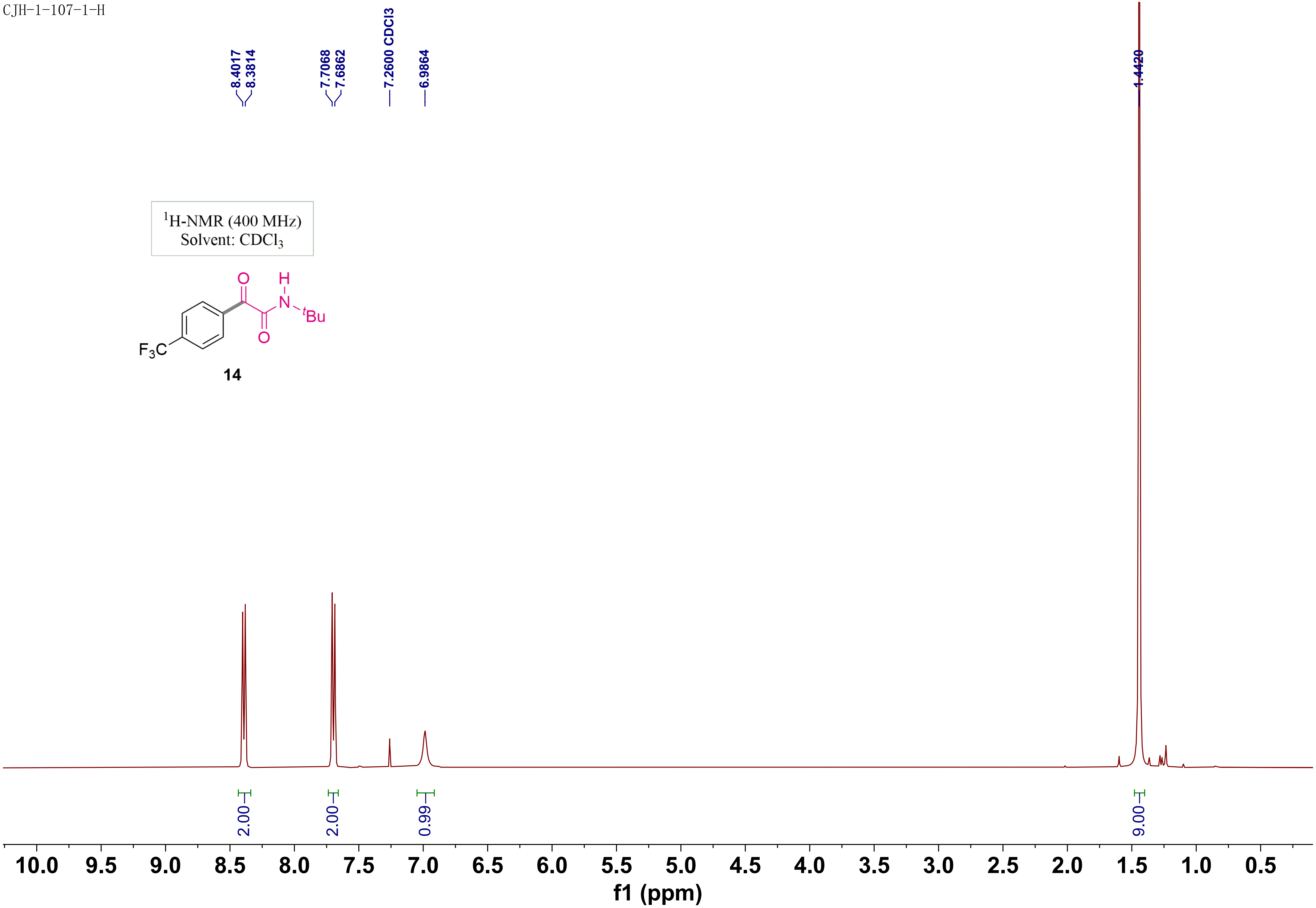

^{1}H-NMR (400 MHz)
Solvent: $CDCl_3$

14

CJH-1-107-1-C

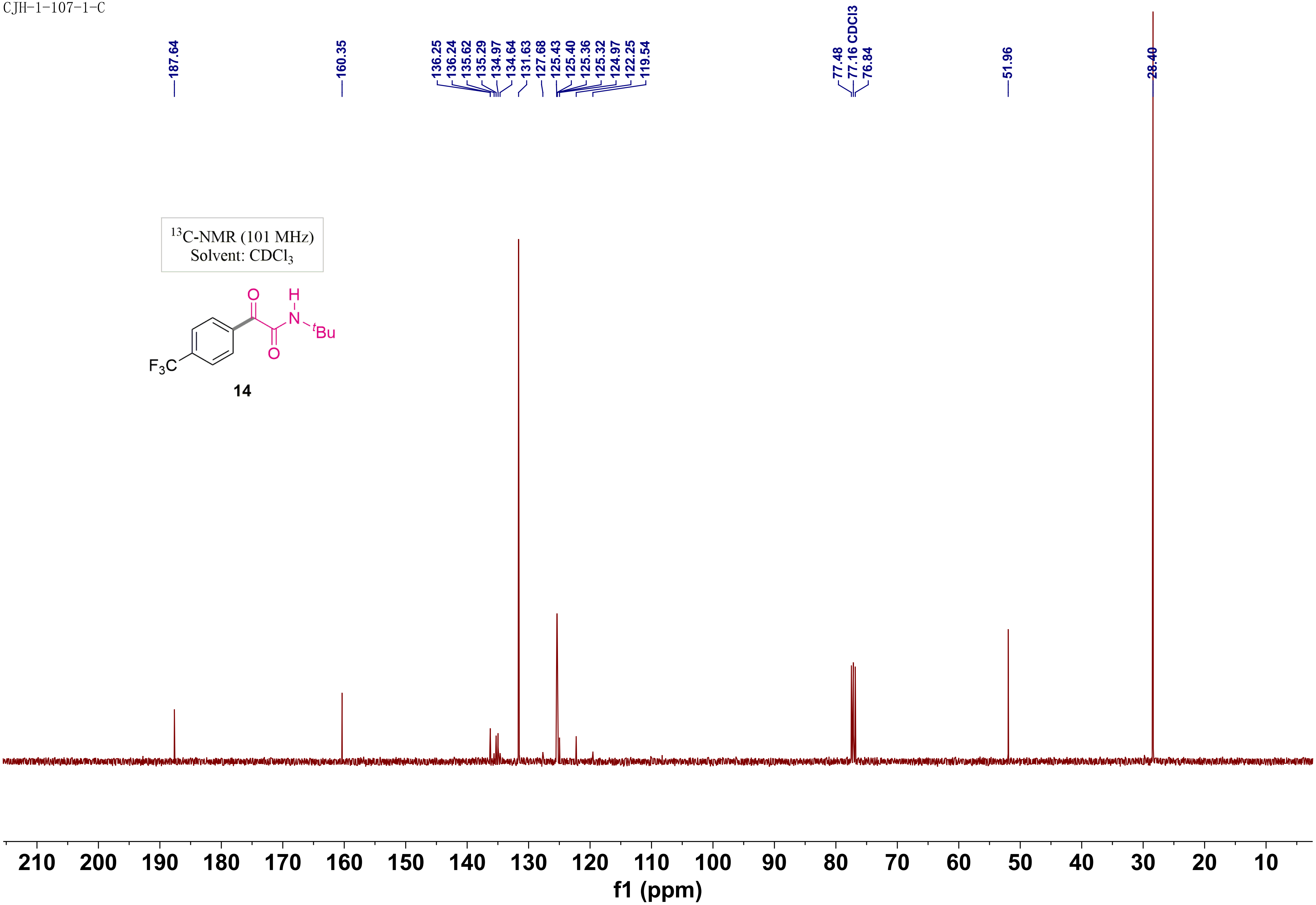
187.64
160.35
136.25
136.24
135.62
135.29
134.97
134.64
131.63
127.68
125.43
125.40
125.36
125.32
124.97
122.25
119.54
77.48
77.16 CDCl3
76.84
51.96
28.40
^{13}C-NMR (101 MHz)
Solvent: $CDCl_3$
O
H
N
^{t}Bu
O
F_3C
14
210
200
190
180
170
160
150
140
130
120
110
100
90
80
70
60
50
40
30
20
10
f1 (ppm)

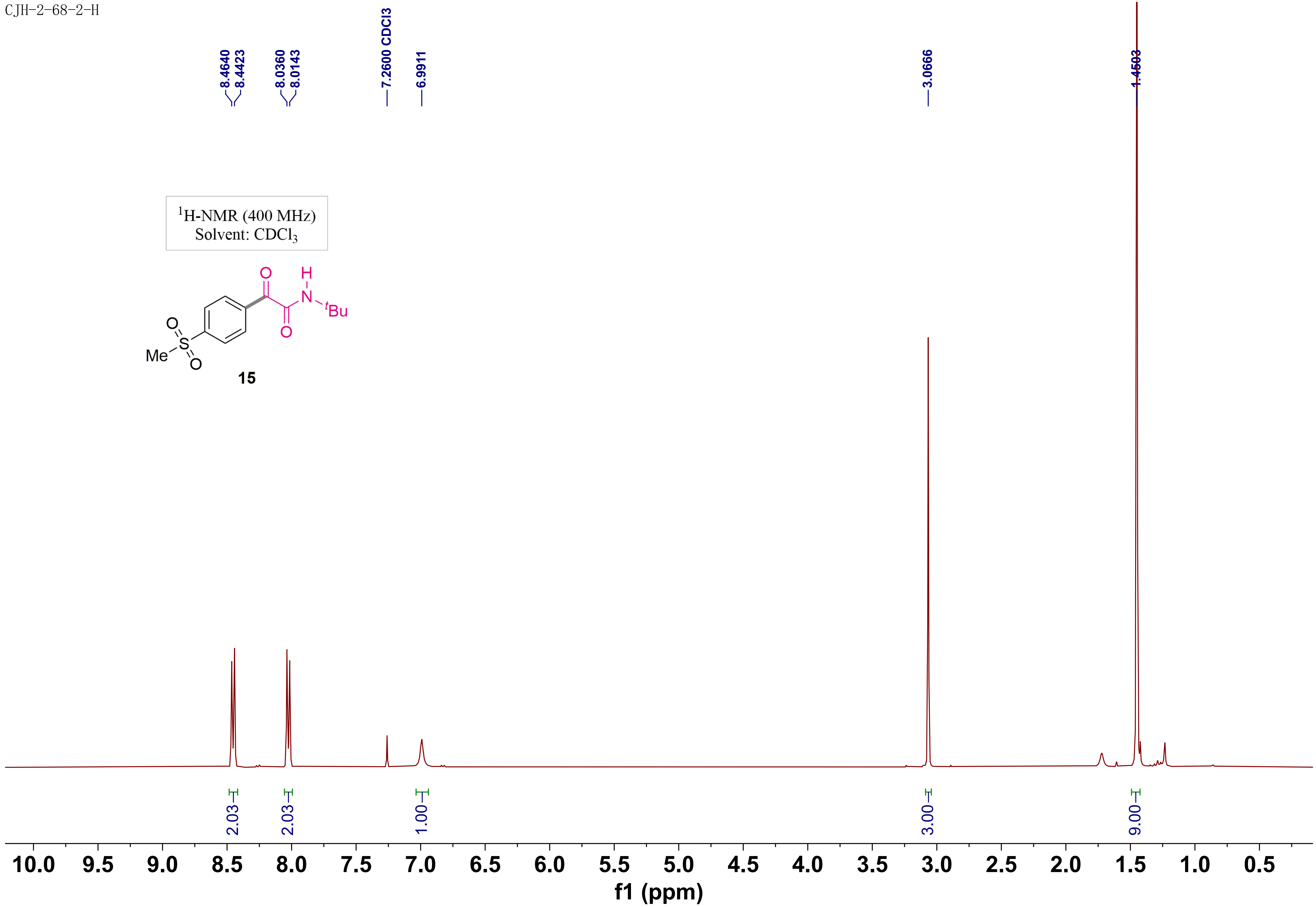
CJH-2-68-2-H
8.4640
8.4423
8.0360
8.0143
7.2600 CDCl3
6.9911
3.0666
1.4503
1H-NMR (400 MHz)
Solvent: CDCl3
O
H
N
tBu
O
O
S
O
Me
15
2.03
2.03
1.00
3.00
9.00
10.0
9.5
9.0
8.5
8.0
7.5
7.0
6.5
6.0
5.5
5.0
4.5
4.0
3.5
3.0
2.5
2.0
1.5
1.0
0.5
f1 (ppm)

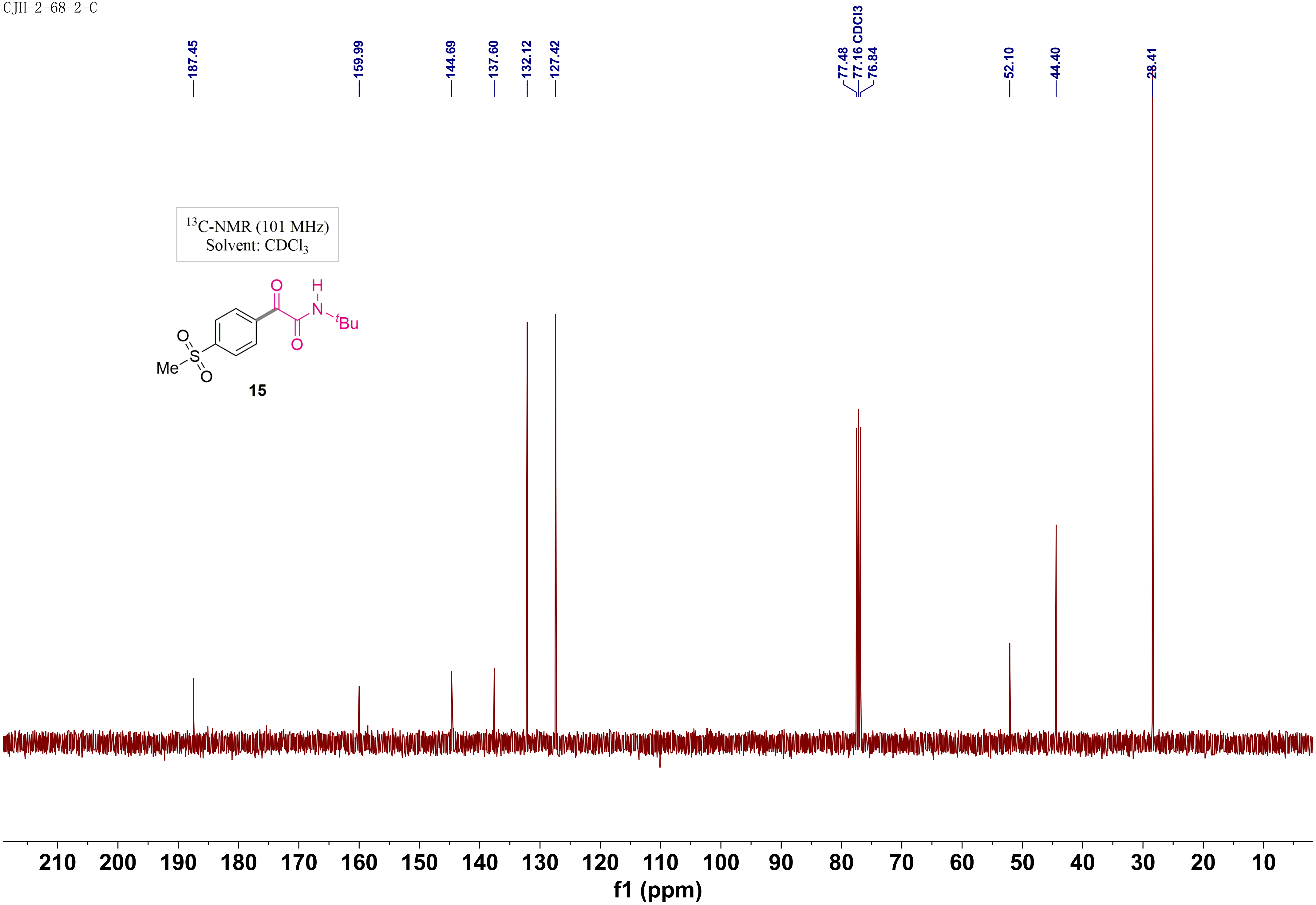
CJH-2-68-2-C
187.45
159.99
144.69
137.60
132.12
127.42
77.48
77.16 CDCl3
76.84
52.10
44.40
28.41
13C-NMR (101 MHz)
Solvent: CDCl3
O
H
N
tBu
O
O
S
Me
O
15
210
200
190
180
170
160
150
140
130
120
110
100
90
80
70
60
50
40
30
20
10
f1 (ppm)

CJH-1-108-1-H

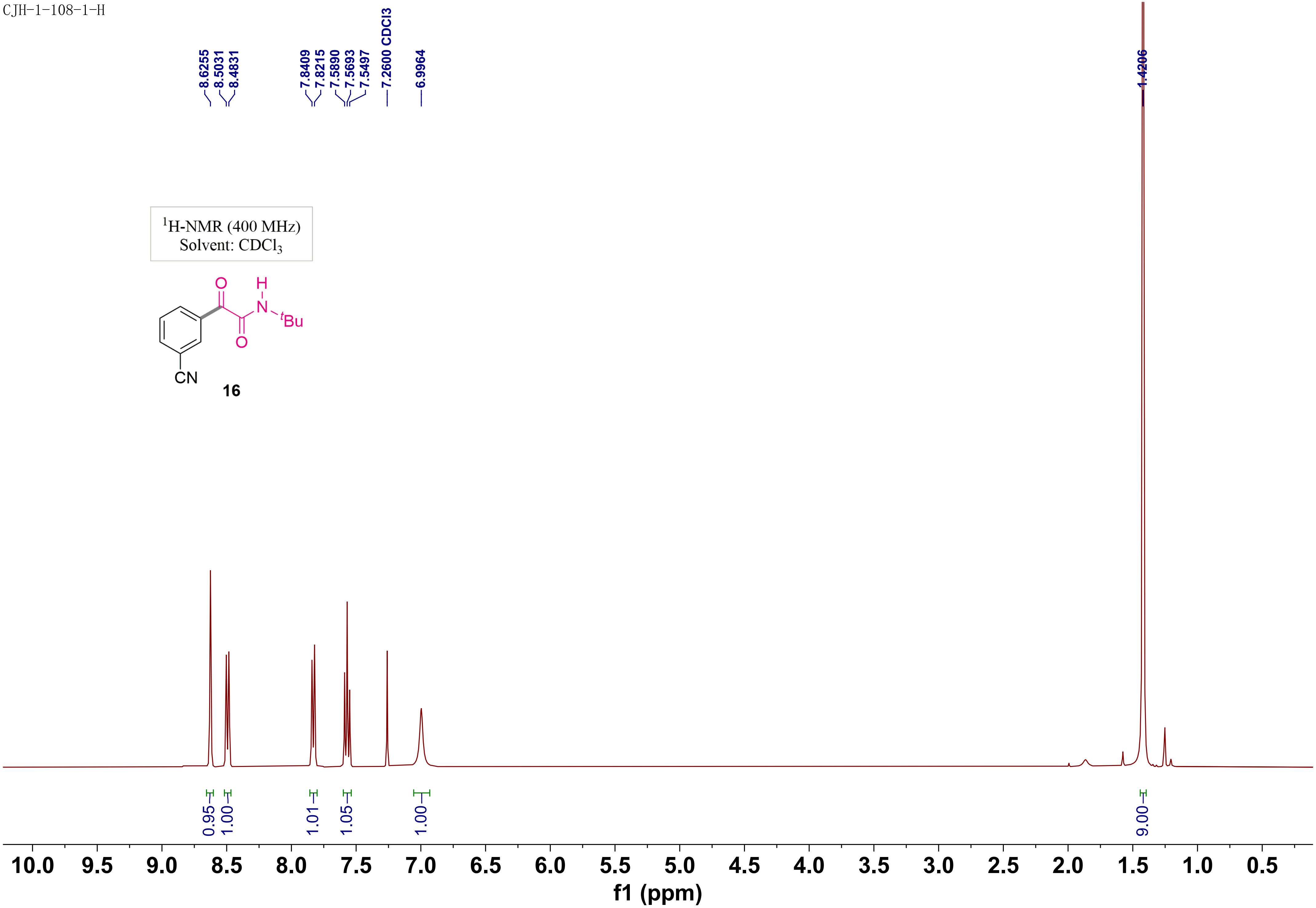

8.6255
8.5031
8.4831
7.8409
7.8215
7.5890
7.5693
7.5497
7.2600 CDCl3
6.9964
1.4206
¹H-NMR (400 MHz)
Solvent: CDCl3
O
H
N
tBu
O
CN
16
0.95
1.00
1.01
1.05
1.00
9.00
10.0
9.5
9.0
8.5
8.0
7.5
7.0
6.5
6.0
5.5
5.0
4.5
4.0
3.5
3.0
2.5
2.0
1.5
1.0
0.5
f1 (ppm)

CJH-1-108-1-C

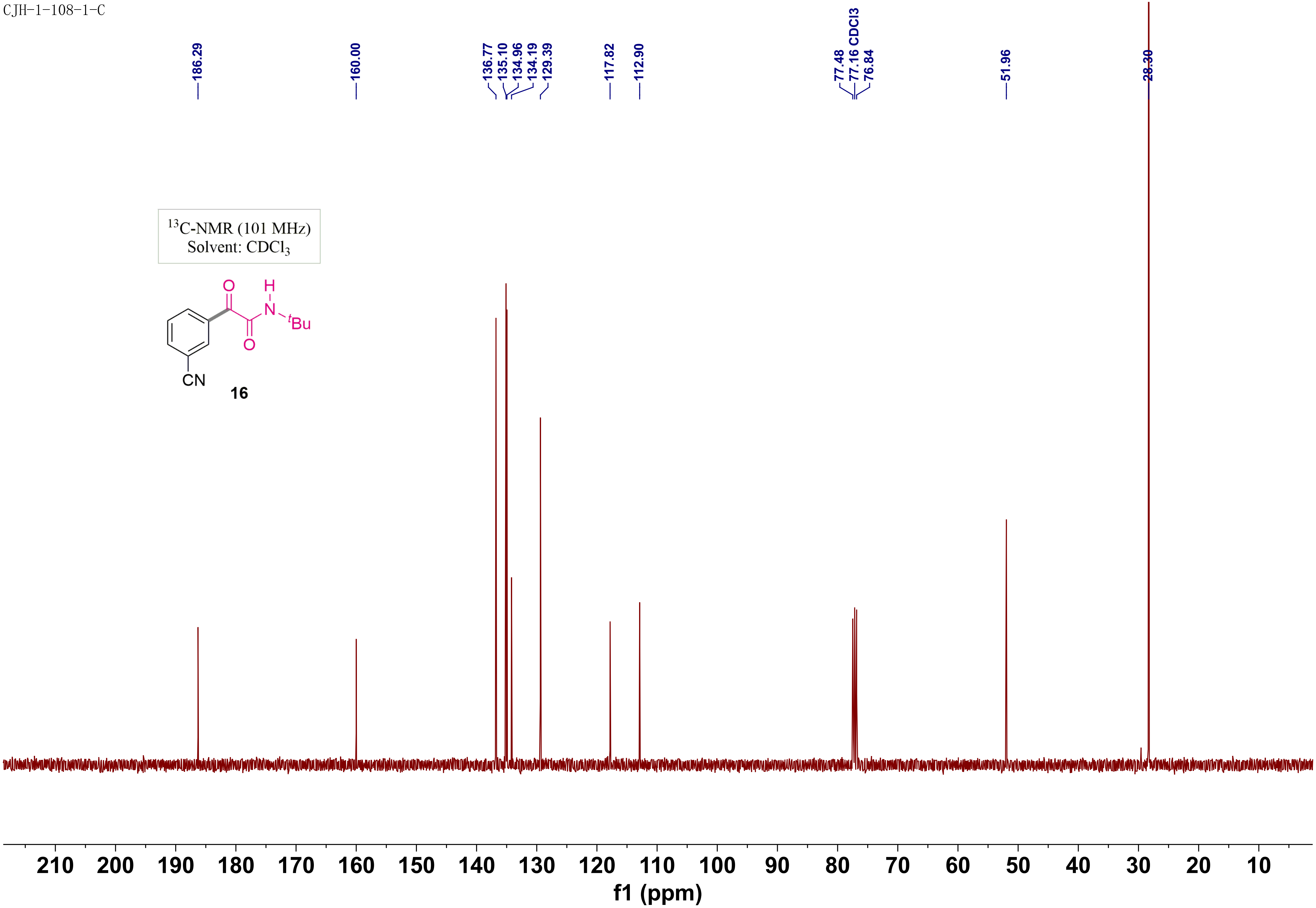

186.29
160.00
136.77
135.10
134.96
134.19
129.39
117.82
112.90
77.48
77.16 CDCl3
76.84
51.96
28.30
13C-NMR (101 MHz)
Solvent: CDCl3
O
H
N
tBu
O
CN
16
210 200 190 180 170 160 150 140 130 120 110 100 90 80 70 60 50 40 30 20 10
f1 (ppm)

CJH-1-108-2-H

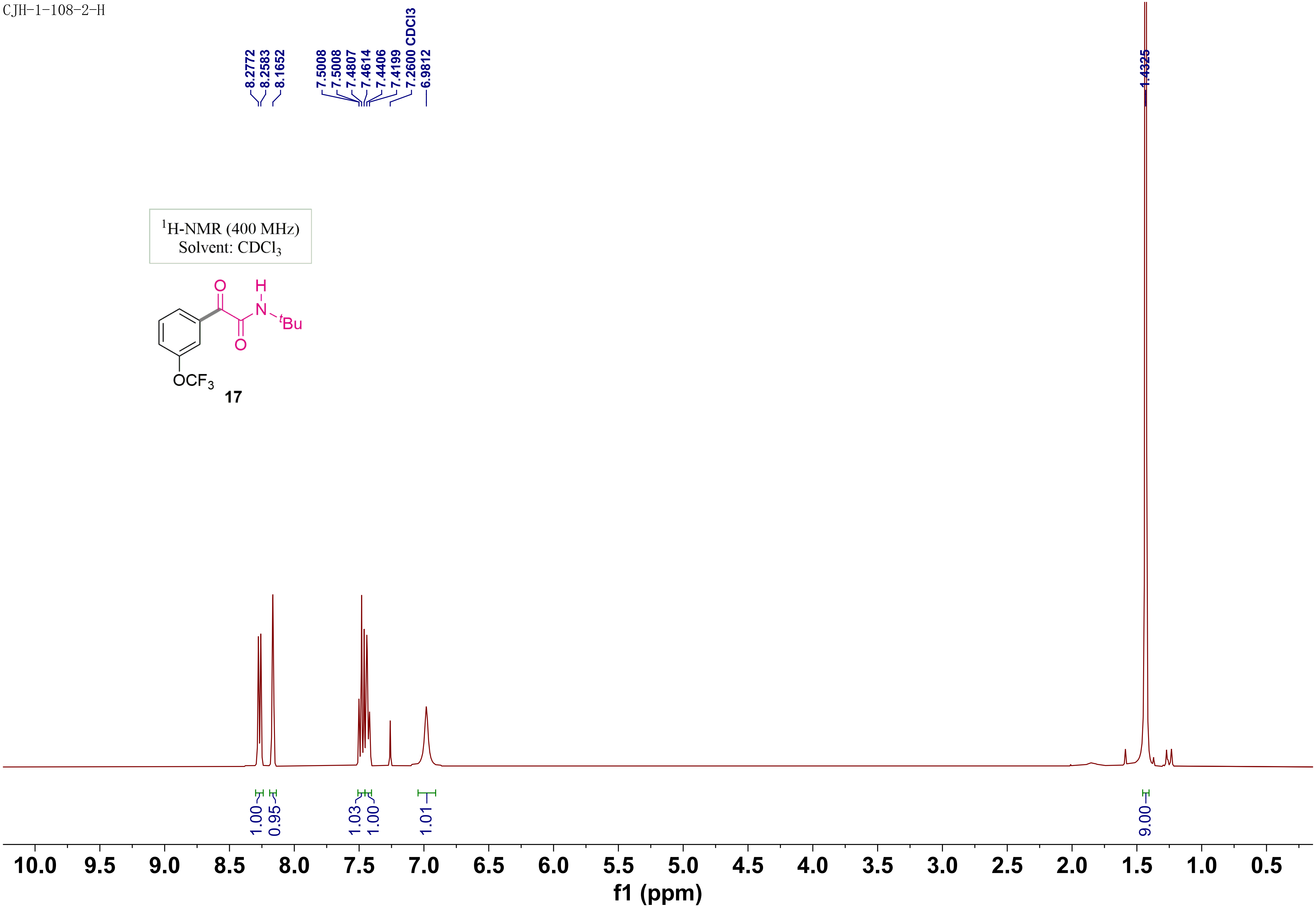

17

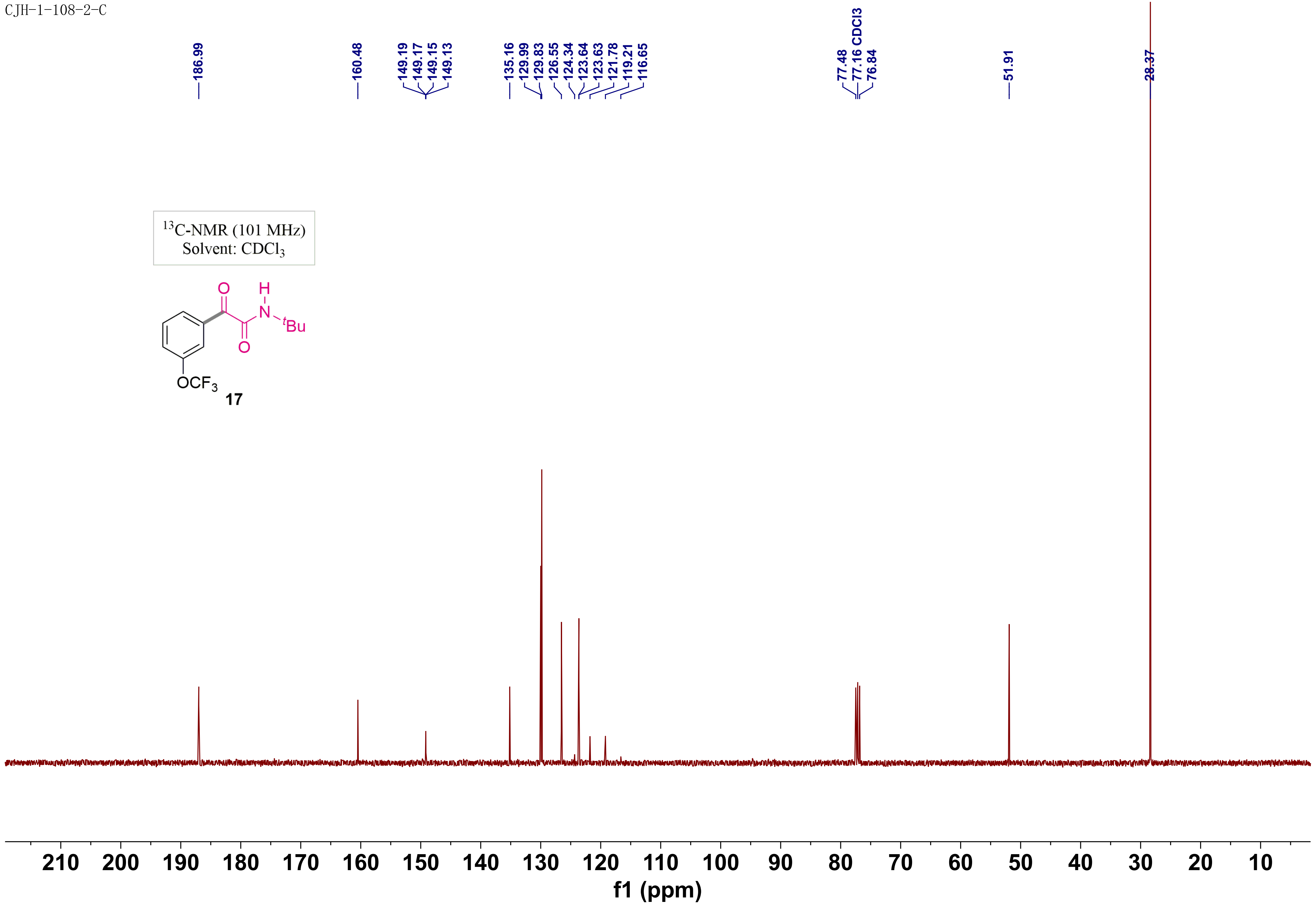
CJH-1-108-2-C
186.99
160.48
149.19
149.17
149.15
149.13
135.16
129.99
129.83
126.55
124.34
123.64
123.63
121.78
119.21
116.65
77.48
77.16 CDCl3
76.84
51.91
28.37
13C-NMR (101 MHz)
Solvent: CDCl3
OCF3
tBu
17
210 200 190 180 170 160 150 140 130 120 110 100 90 80 70 60 50 40 30 20 10
f1 (ppm)

CJH-2-68-1-H

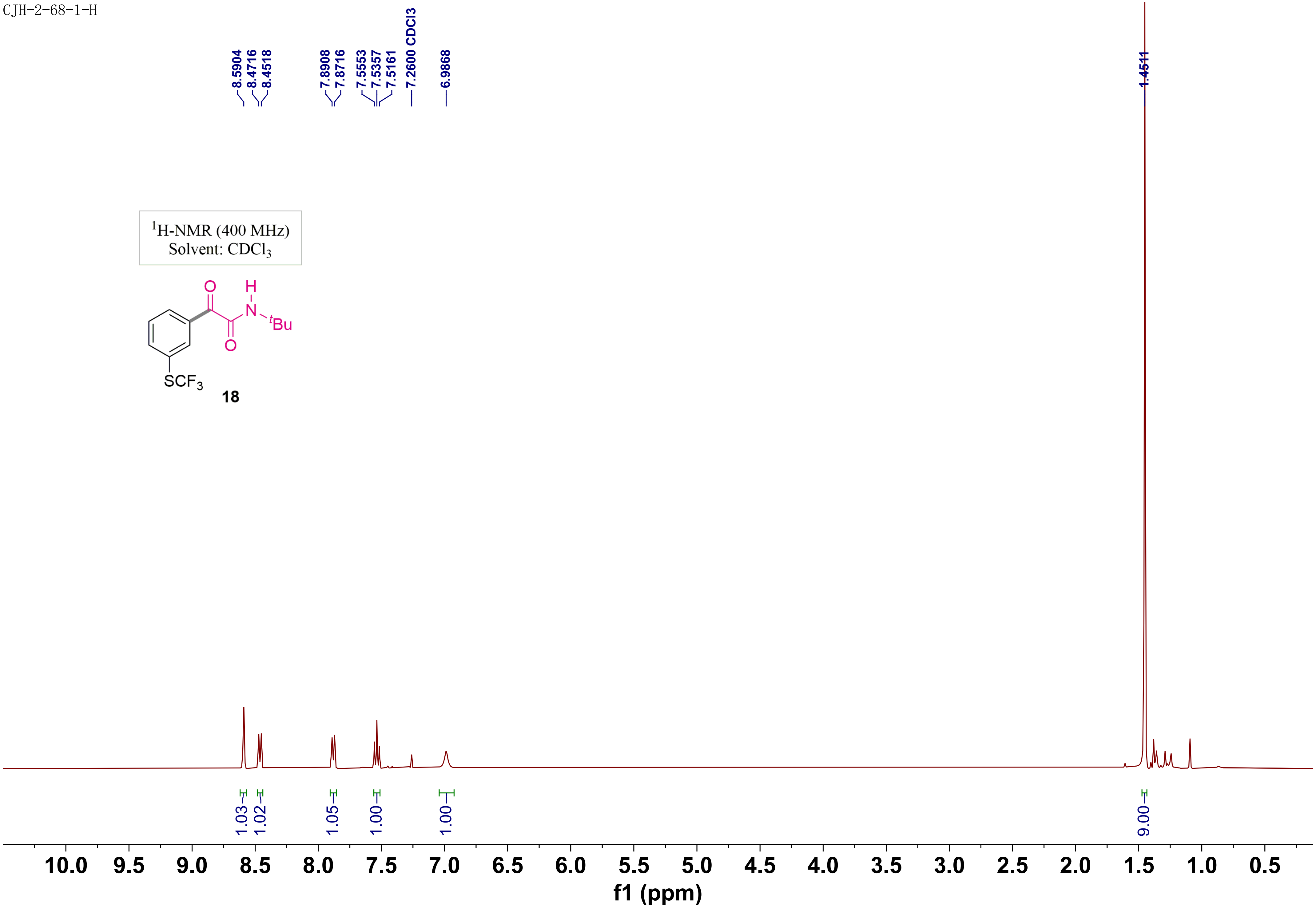
8.5904
8.4716
8.4518
7.8908
7.8716
7.5553
7.5357
7.5161
7.2600 CDCl3
6.9868
1.4511
1H-NMR (400 MHz)
Solvent: CDCl3
O
H
N
tBu
O
SCF3
18
1.03
1.02
1.05
1.00
1.00
9.00
10.0
9.5
9.0
8.5
8.0
7.5
7.0
6.5
6.0
5.5
5.0
4.5
4.0
3.5
3.0
2.5
2.0
1.5
1.0
0.5
f1 (ppm)

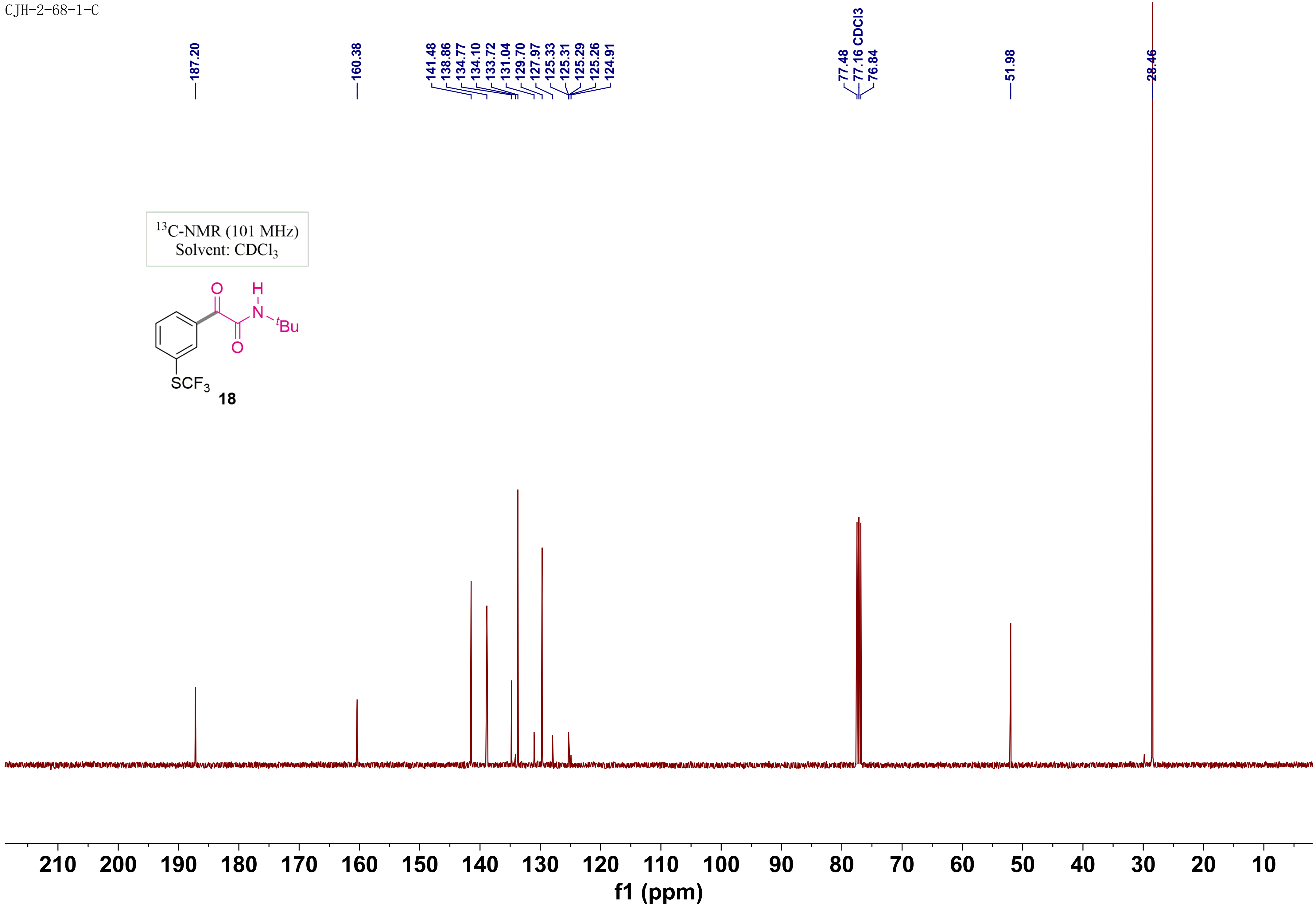
CJH-2-68-1-C
187.20
160.38
141.48
138.86
134.77
134.10
133.72
131.04
129.70
127.97
125.33
125.31
125.29
125.26
124.91
77.48
77.16 CDCl3
76.84
51.98
28.46
^{13}C-NMR (101 MHz)
Solvent: $CDCl_3$
O
H
N
^{t}Bu
O
SCF_3
18
210
200
190
180
170
160
150
140
130
120
110
100
90
80
70
60
50
40
30
20
10
f1 (ppm)

CJH-1-104-2-H

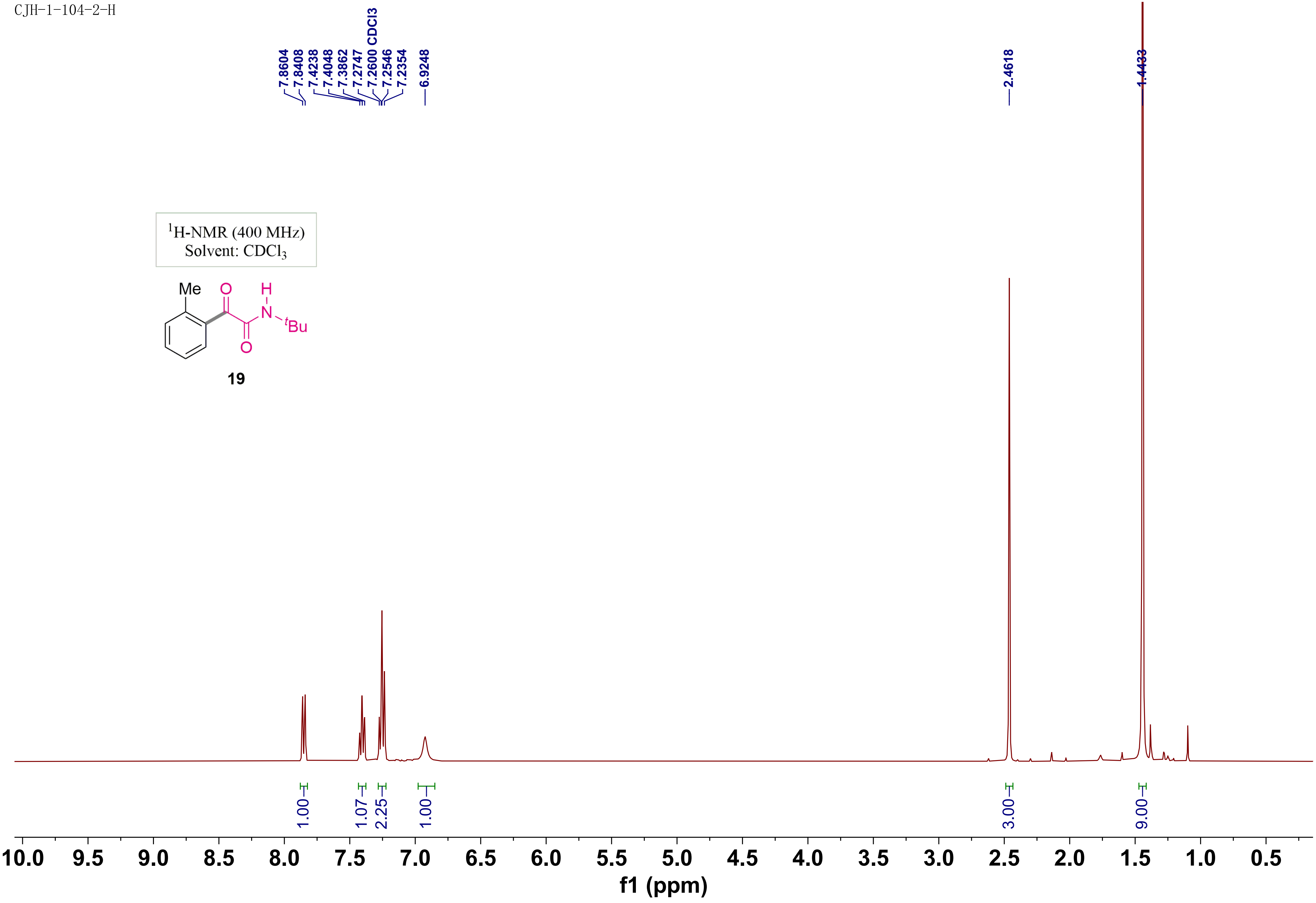

7.8604
7.8408
7.4238
7.4048
7.3862
7.2747
7.2600 CDCl3
7.2546
7.2354
6.9248
2.4618
1.4433
^{1}H-NMR (400 MHz)
Solvent: $CDCl_3$
Me
O
H
N
^{t}Bu
O
19
1.00
1.07
2.25
1.00
3.00
9.00
10.0
9.5
9.0
8.5
8.0
7.5
7.0
6.5
6.0
5.5
5.0
4.5
4.0
3.5
3.0
2.5
2.0
1.5
1.0
0.5
f1 (ppm)

CJH-1-104-2-C

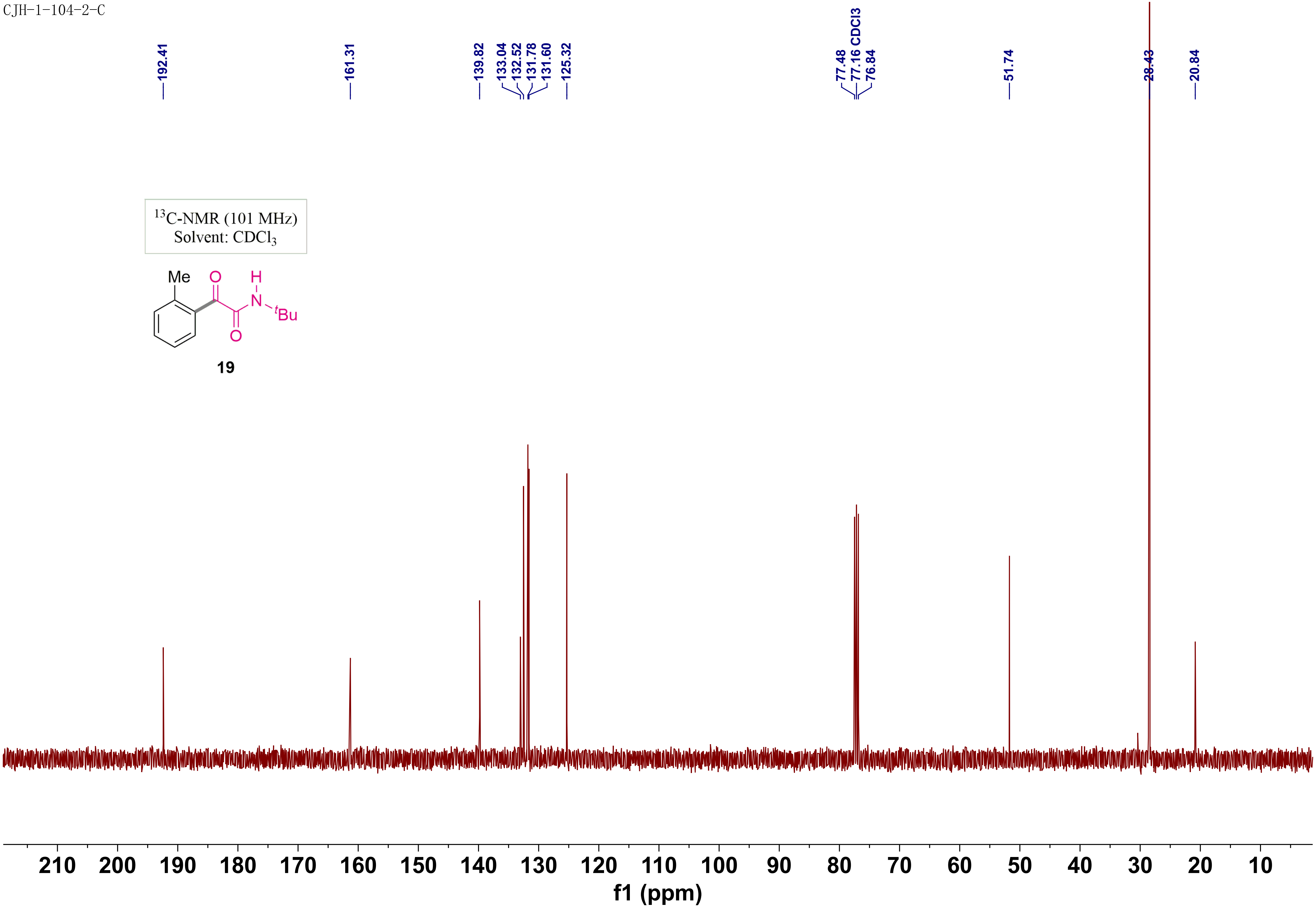
192.41
161.31
139.82
133.04
132.52
131.78
131.60
125.32
77.48
77.16 CDCl3
76.84
51.74
28.43
20.84
^{13}C-NMR (101 MHz)
Solvent: $CDCl_3$
Me
O
H
N
^{t}Bu
O
19
210 200 190 180 170 160 150 140 130 120 110 100 90 80 70 60 50 40 30 20 10
f1 (ppm)

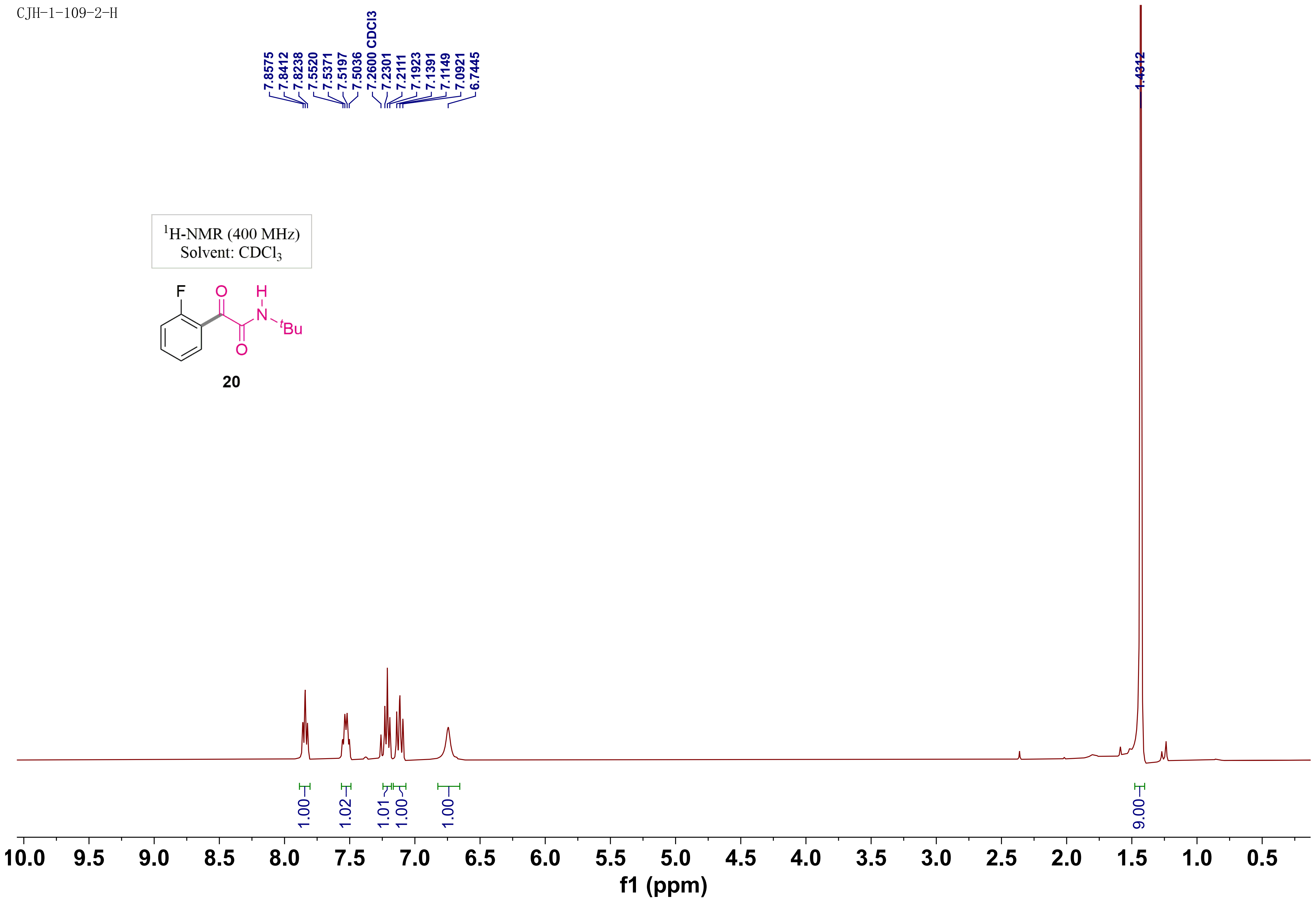
CJH-1-109-2-H
7.8575
7.8412
7.8238
7.5520
7.5371
7.5197
7.5036
7.2600 CDCl3
7.2301
7.2111
7.1923
7.1391
7.1149
7.0921
6.7445
1.4312
^{1}H-NMR (400 MHz)
Solvent: $CDCl_3$
F
O
H
N
^{t}Bu
O
20
1.00
1.02
1.01
1.00
1.00
9.00
10.0 9.5 9.0 8.5 8.0 7.5 7.0 6.5 6.0 5.5 5.0 4.5 4.0 3.5 3.0 2.5 2.0 1.5 1.0 0.5
f1 (ppm)

CJH-1-109-2-C

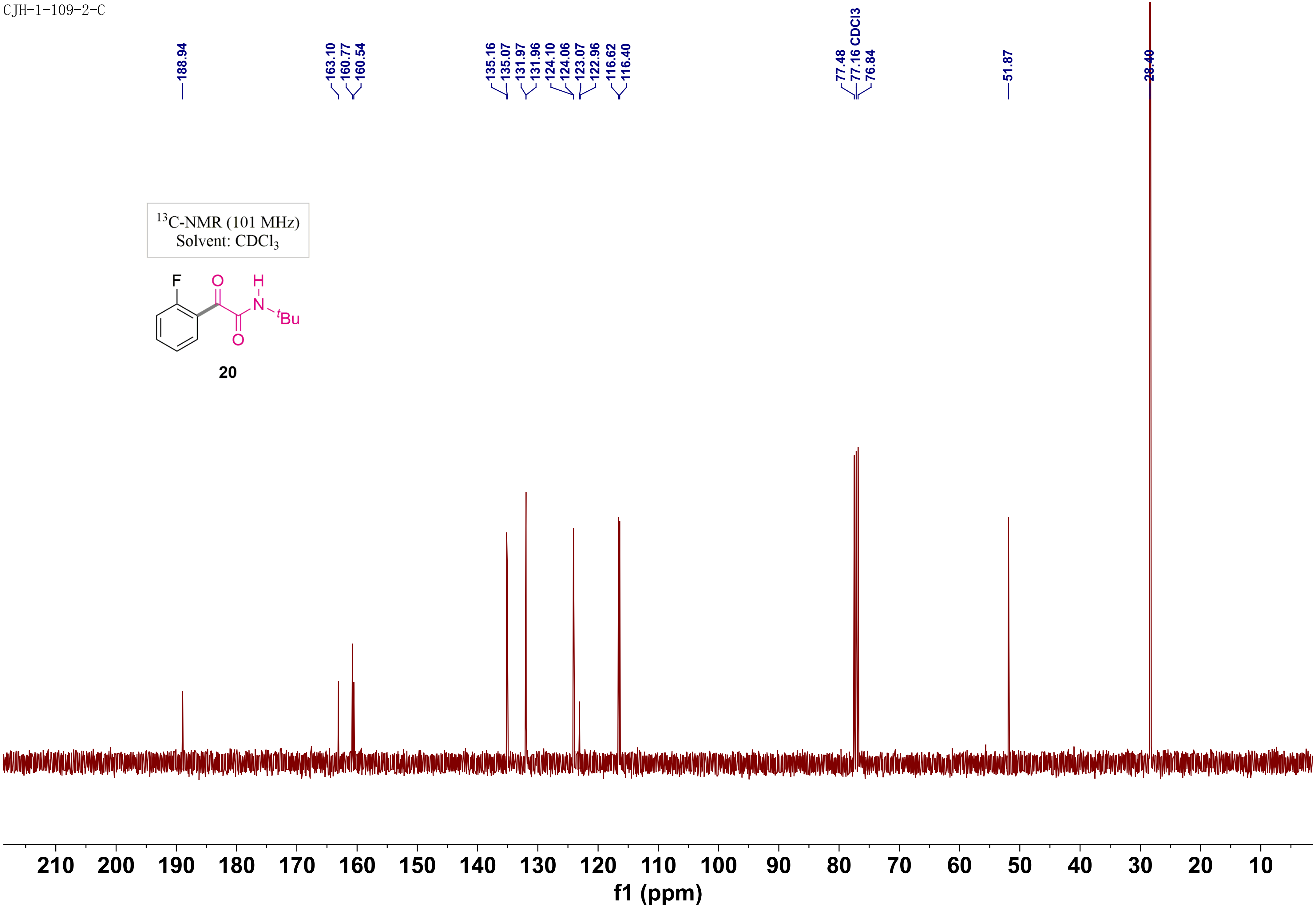

^{13}C-NMR (101 MHz)
Solvent: $CDCl_3$

20

CJH-1-110-2-H

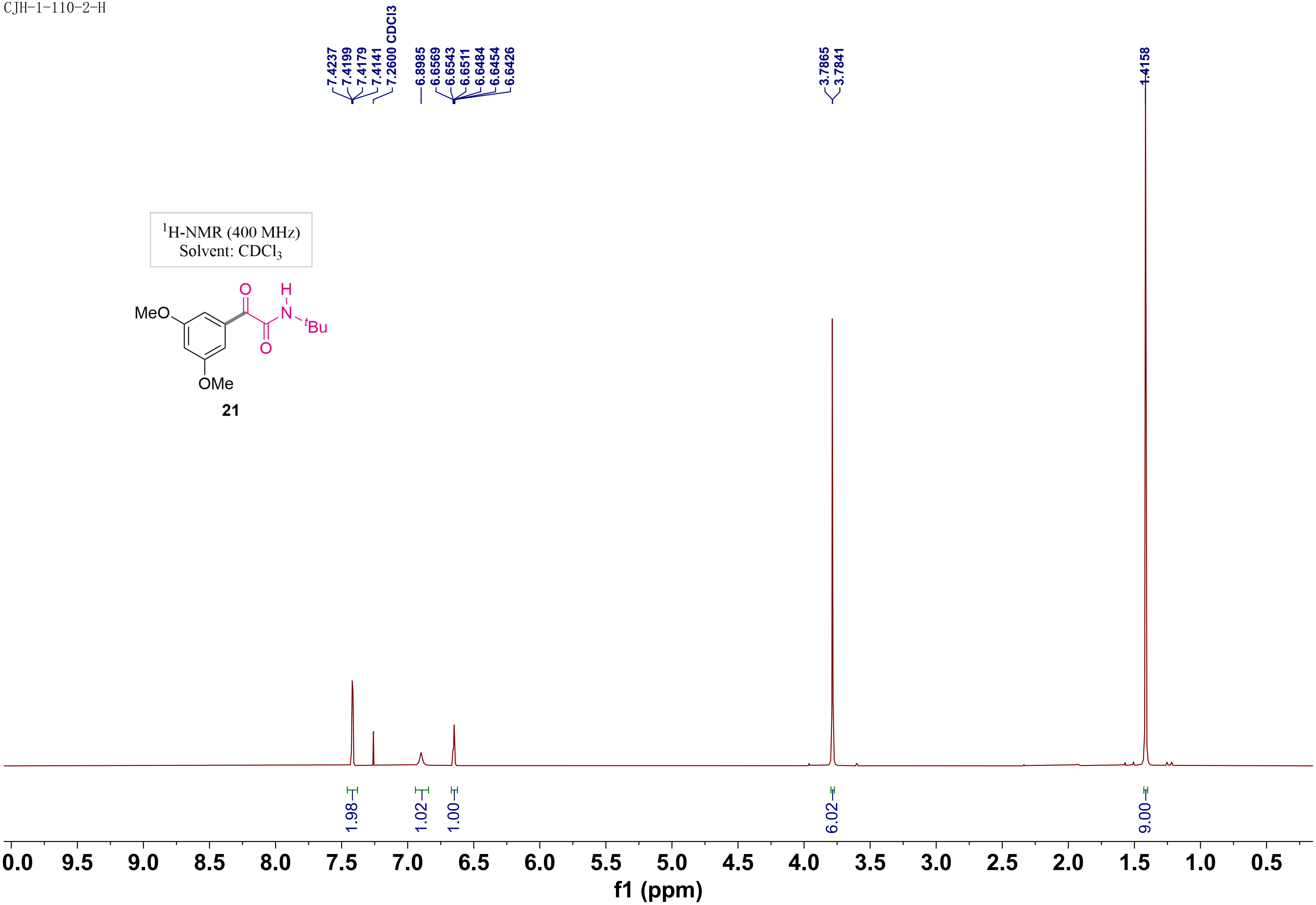
7.4237
7.4199
7.4179
7.4141
7.2600 CDCl3
6.8985
6.6569
6.6543
6.6511
6.6484
6.6454
6.6426
3.7865
3.7841
1.4158
^{1}H-NMR (400 MHz)
Solvent: $CDCl_3$
MeO
OMe
O
O
H
N
^{t}Bu
21
1.98
1.02
1.00
6.02
9.00
10.0
9.5
9.0
8.5
8.0
7.5
7.0
6.5
6.0
5.5
5.0
4.5
4.0
3.5
3.0
2.5
2.0
1.5
1.0
0.5
f1 (ppm)

CJH-1-110-2-C

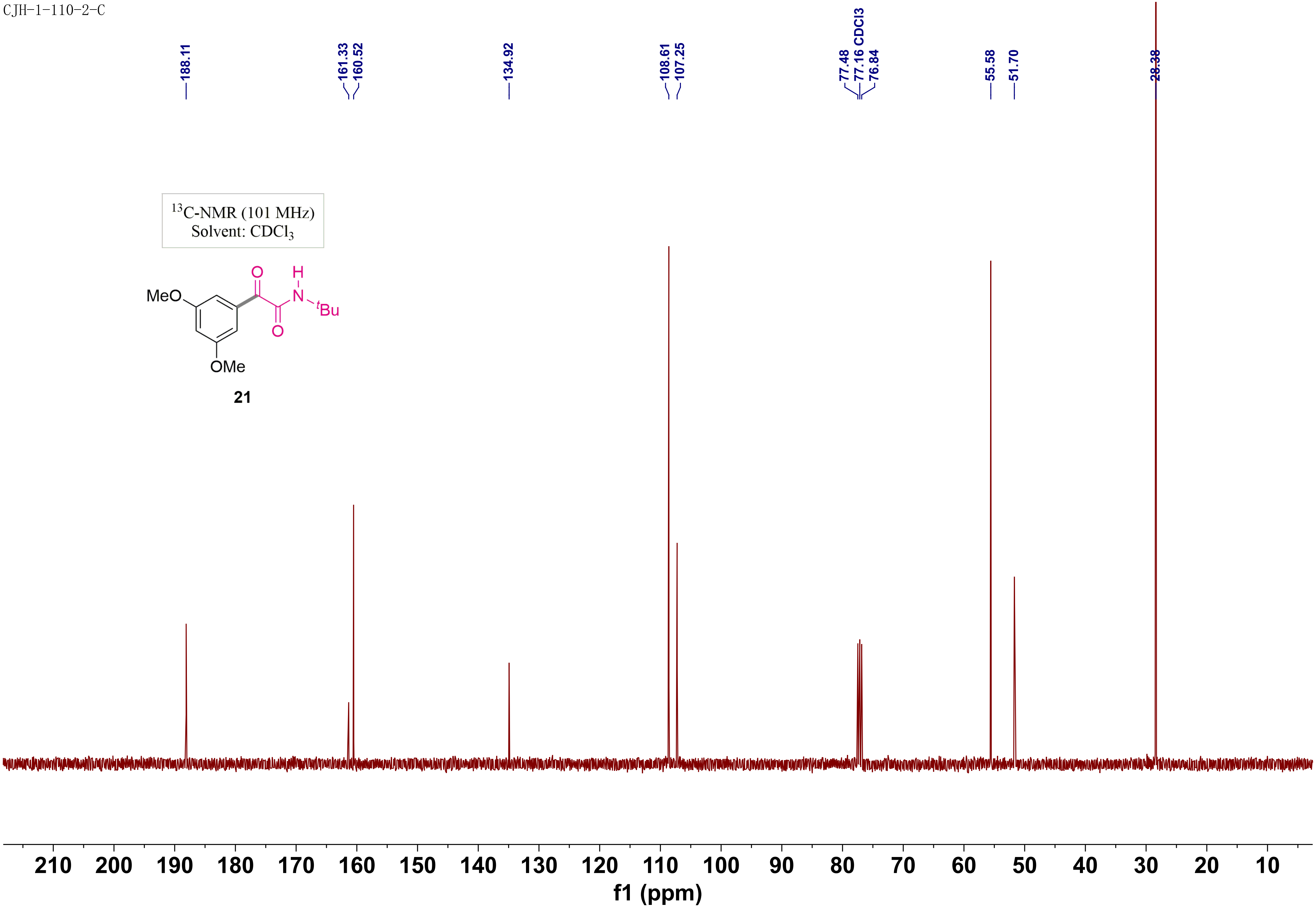
188.11
161.33
160.52
134.92
108.61
107.25
77.48
77.16 CDCl3
76.84
55.58
51.70
28.38
13C-NMR (101 MHz)
Solvent: CDCl3
MeO
O
H
N
tBu
O
OMe
21
210
200
190
180
170
160
150
140
130
120
110
100
90
80
70
60
50
40
30
20
10
f1 (ppm)

CJH-1-110-1-H

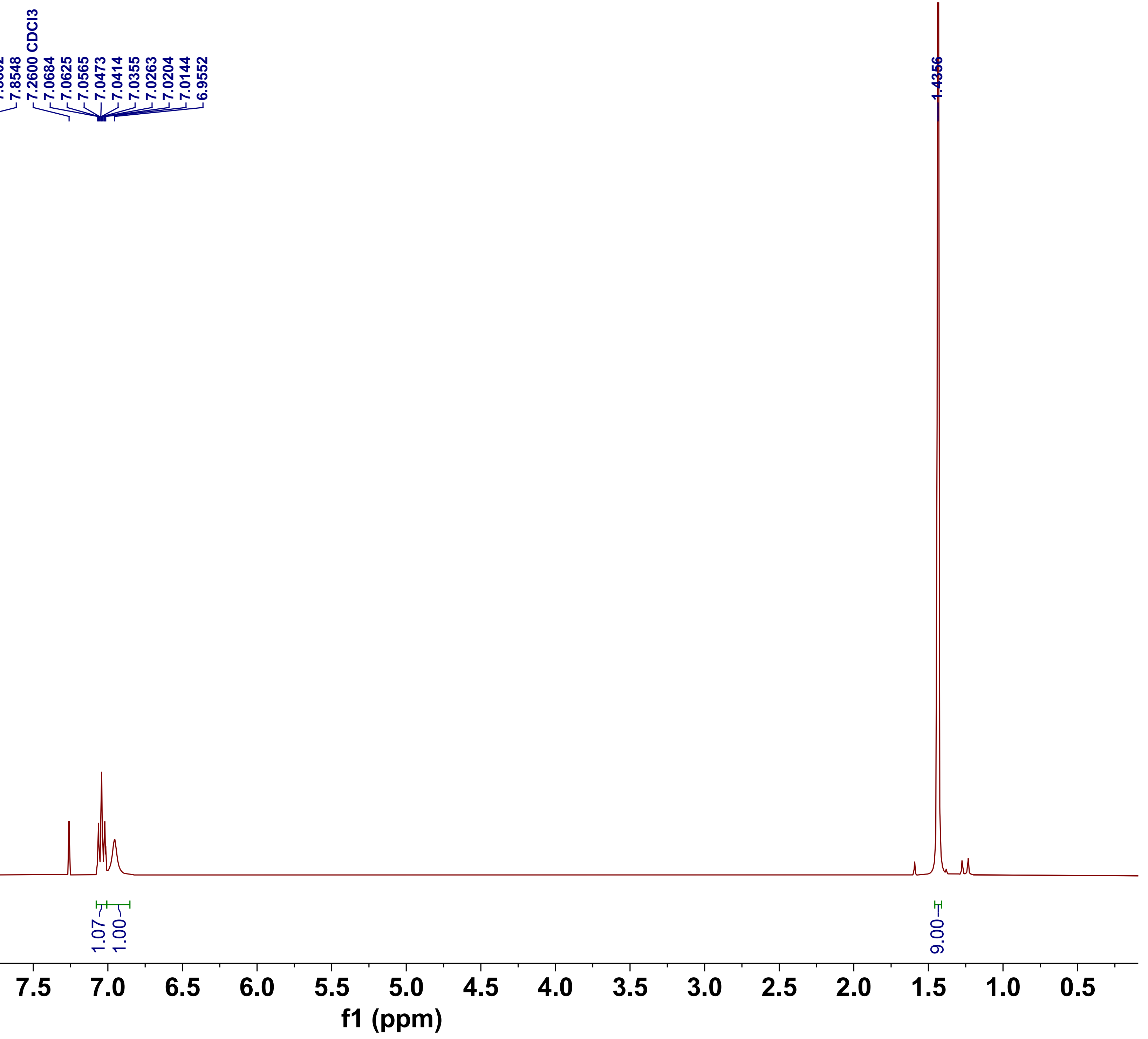

^{1}H-NMR (400 MHz)
Solvent: $CDCl_3$

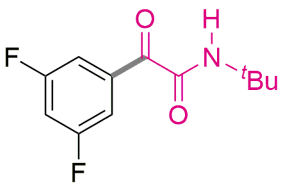

22

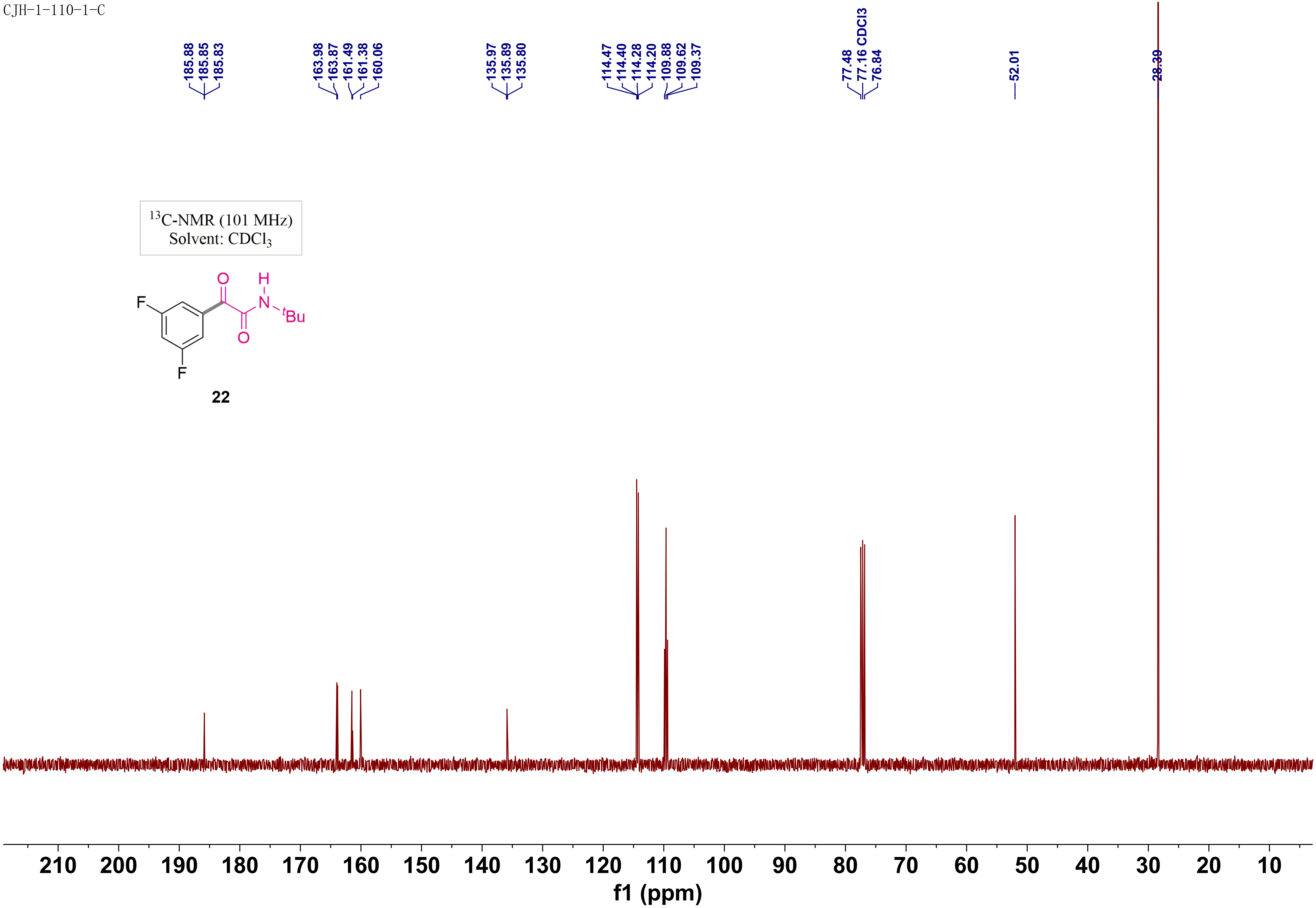
CJH-1-110-1-C
185.88
185.85
185.83
163.98
163.87
161.49
161.38
160.06
135.97
135.89
135.80
114.47
114.40
114.28
114.20
109.88
109.62
109.37
77.48
77.16 CDCl3
76.84
52.01
28.39
^{13}C-NMR (101 MHz)
Solvent: $CDCl_3$
F
F
O
O
H
N
^{t}Bu
22
210 200 190 180 170 160 150 140 130 120 110 100 90 80 70 60 50 40 30 20 10
f1 (ppm)

CJH-2-67-1-H

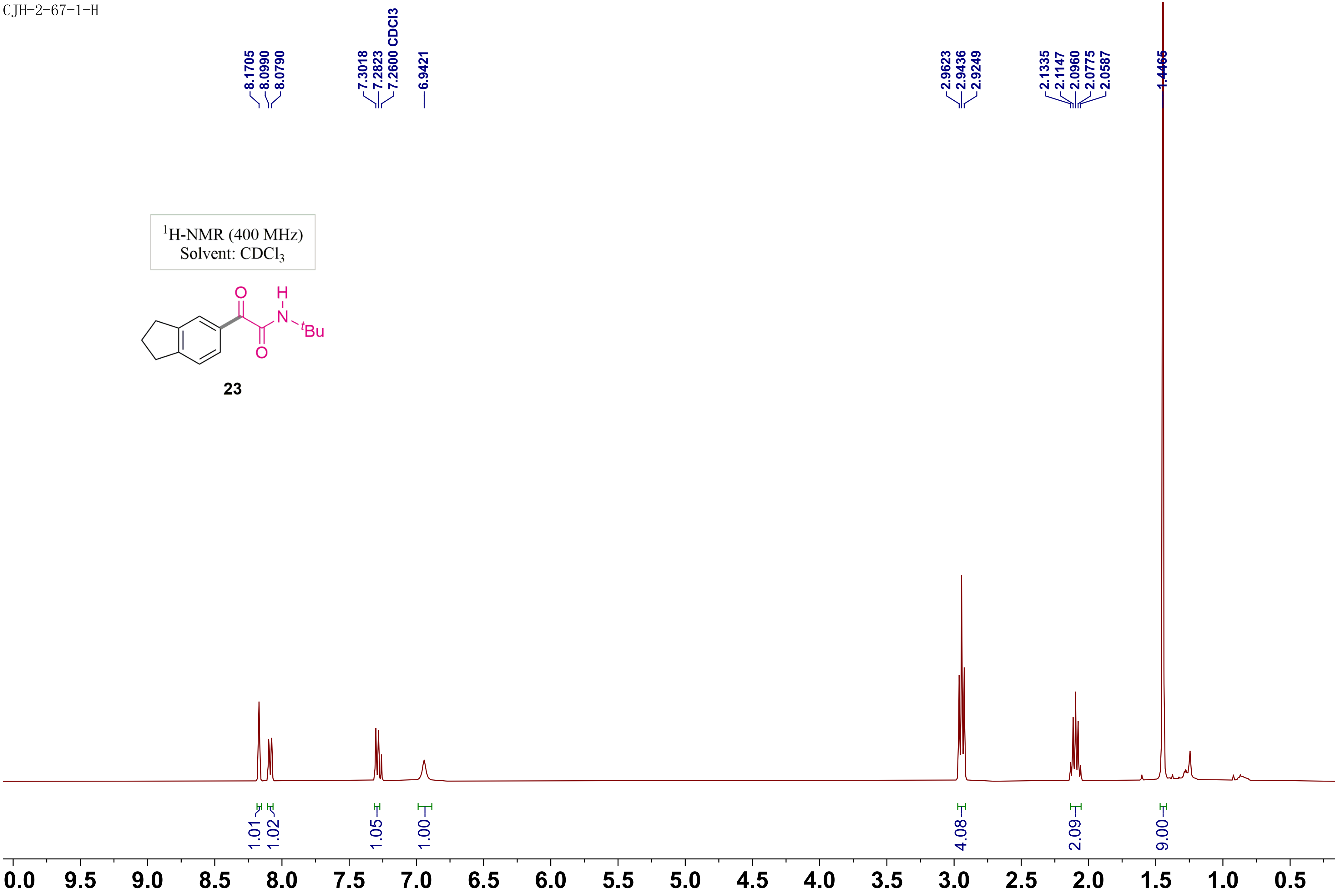

8.1705
8.0990
8.0790
7.3018
7.2823
7.2600 CDCl3
6.9421
2.9623
2.9436
2.9249
2.1335
2.1147
2.0960
2.0775
2.0587
1.4465
^{1}H-NMR (400 MHz)
Solvent: $CDCl_3$
O
H
N
^{t}Bu
O
23
1.01
1.02
1.05
1.00
4.08
2.09
9.00
10.0
9.5
9.0
8.5
8.0
7.5
7.0
6.5
6.0
5.5
5.0
4.5
4.0
3.5
3.0
2.5
2.0
1.5
1.0
0.5
f1 (ppm)

CJH-2-67-1-C

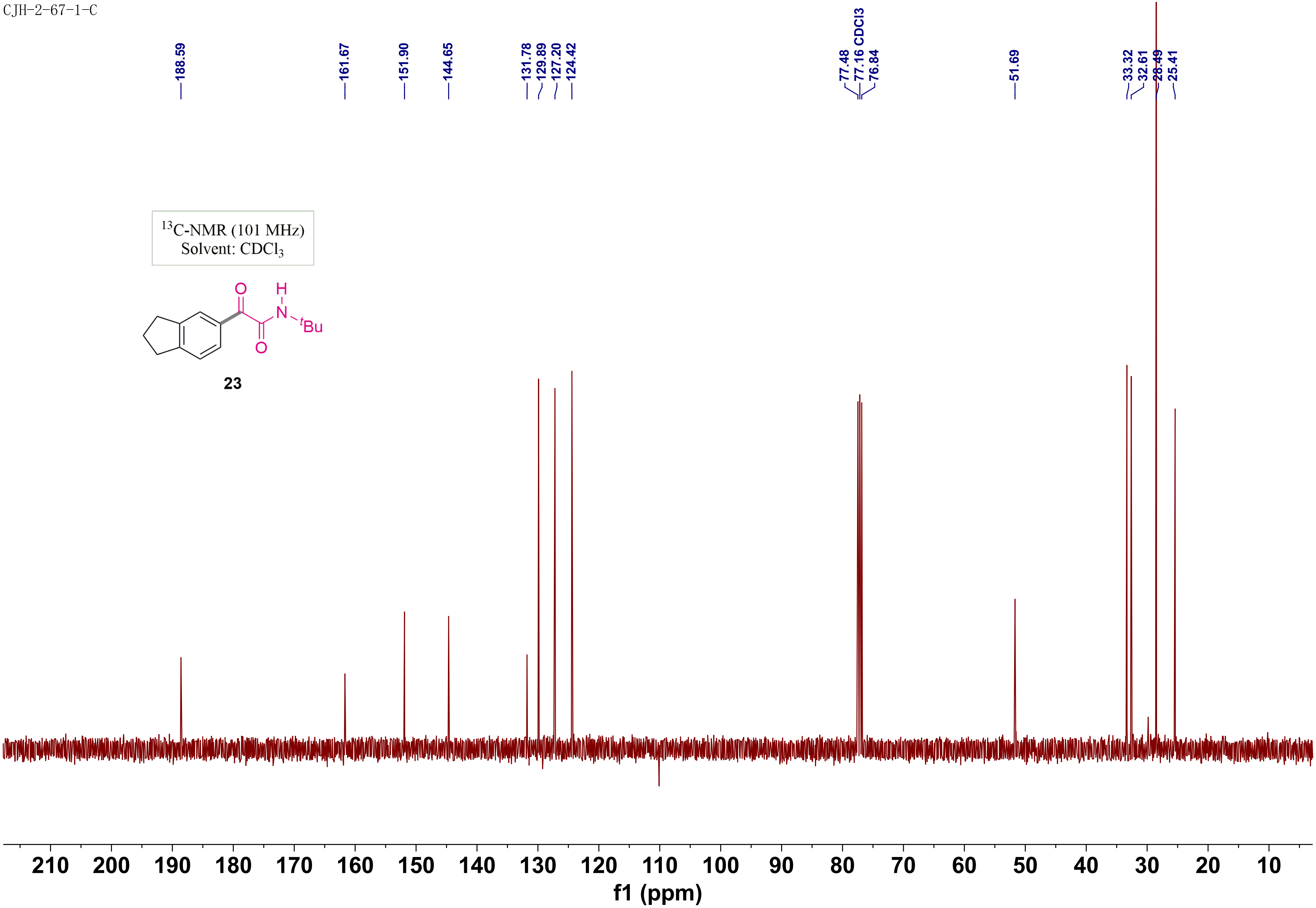

188.59
161.67
151.90
144.65
131.78
129.89
127.20
124.42
77.48
77.16 CDCl3
76.84
51.69
33.32
32.61
28.49
25.41
^{13}C-NMR (101 MHz)
Solvent: $CDCl_3$
210 200 190 180 170 160 150 140 130 120 110 100 90 80 70 60 50 40 30 20 10
f1 (ppm)
O
H
N
tBu
O
23

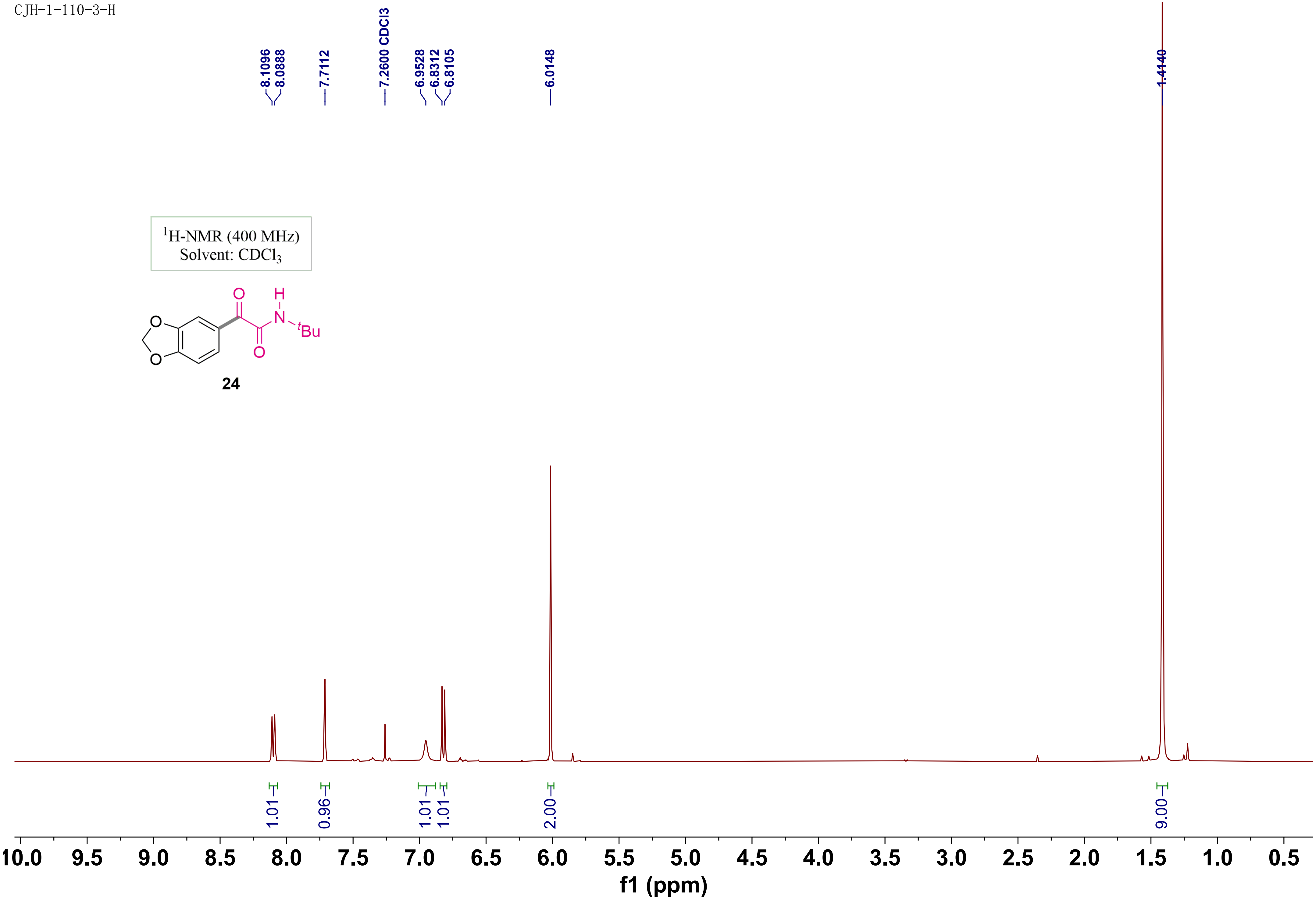
CJH-1-110-3-H
8.1096
8.0888
7.7112
7.2600 CDCl3
6.9528
6.8312
6.8105
6.0148
1.4140
^{1}H-NMR (400 MHz)
Solvent: $CDCl_3$
O
H
N
O
O
^{t}Bu
O
24
1.01
0.96
1.01
1.01
2.00
9.00
10.0 9.5 9.0 8.5 8.0 7.5 7.0 6.5 6.0 5.5 5.0 4.5 4.0 3.5 3.0 2.5 2.0 1.5 1.0 0.5
f1 (ppm)

CJH-1-110-3-C

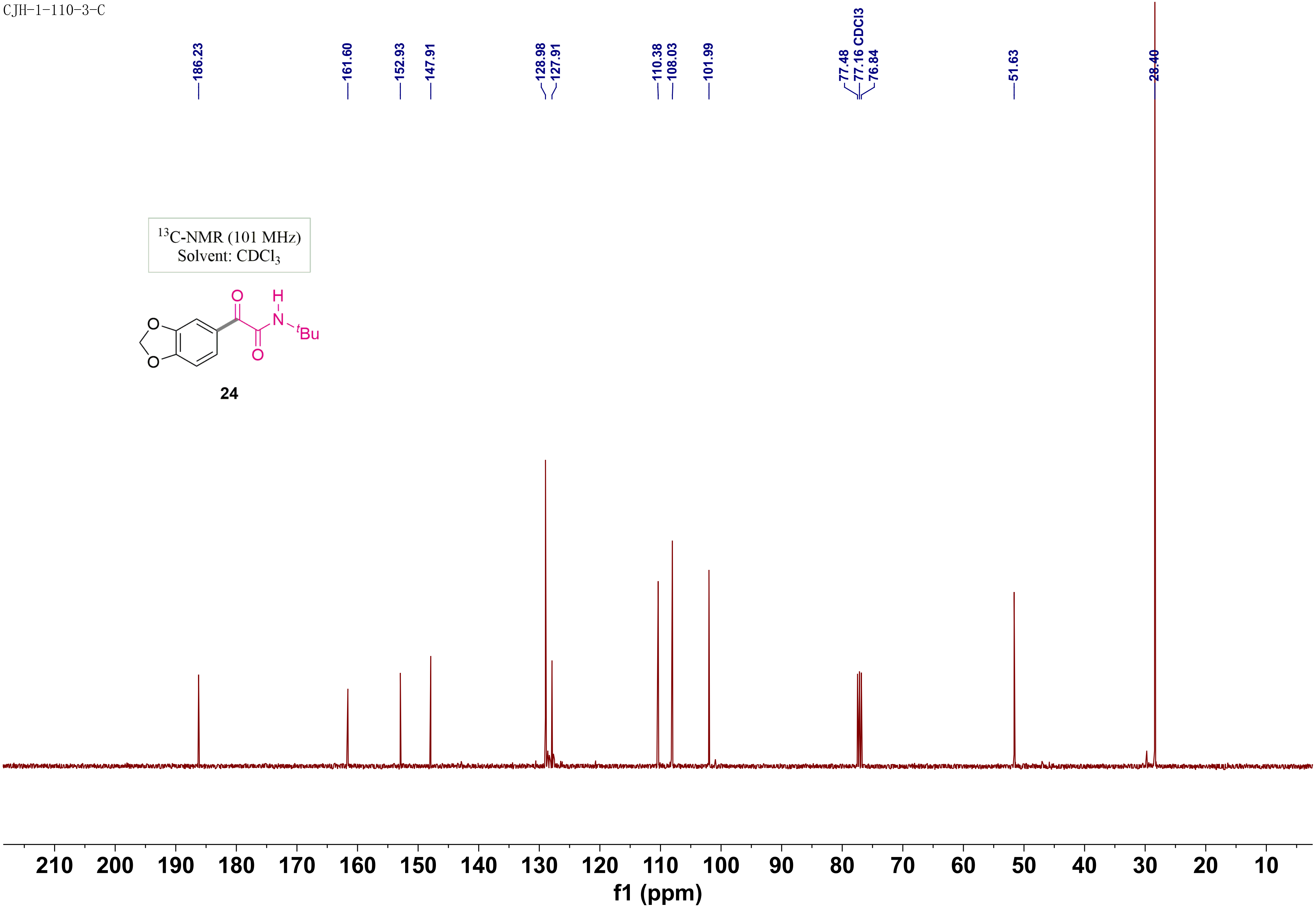
186.23
161.60
152.93
147.91
128.98
127.91
110.38
108.03
101.99
77.48
77.16 CDCl3
76.84
51.63
28.40
^{13}C-NMR (101 MHz)
Solvent: $CDCl_3$
O
H
N
tBu
O
O
O
24
210
200
190
180
170
160
150
140
130
120
110
100
90
80
70
60
50
40
30
20
10
f1 (ppm)

CJH-2-64-1-H

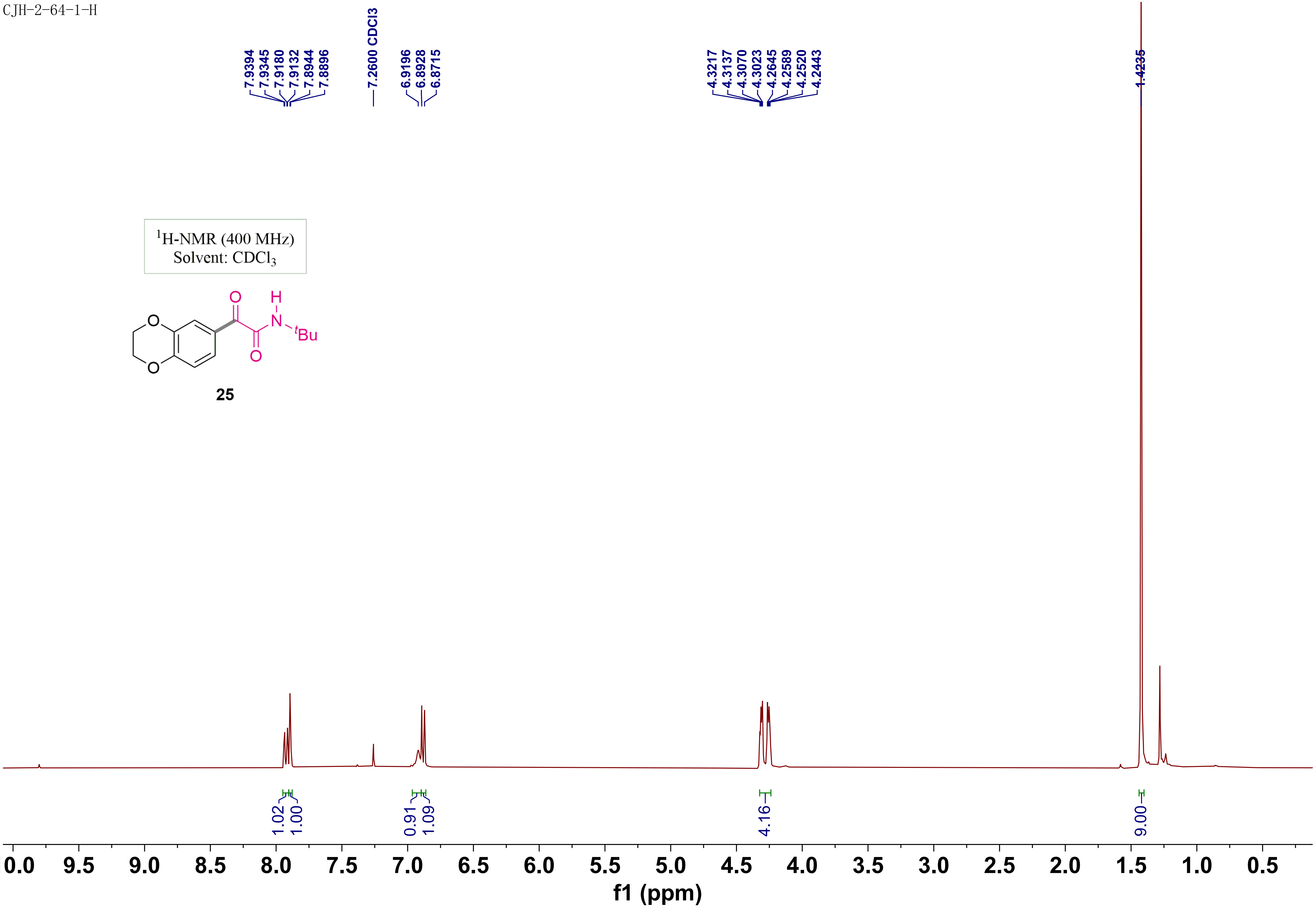

7.9394
7.9345
7.9180
7.9132
7.8944
7.8896
7.2600 CDCl3
6.9196
6.8928
6.8715
4.3217
4.3137
4.3070
4.3023
4.2645
4.2589
4.2520
4.2443
1.4235
^{1}H-NMR (400 MHz)
Solvent: $CDCl_3$
O
H
N
tBu
O
O
O
25
1.02
1.00
0.91
1.09
4.16
9.00
10.0 9.5 9.0 8.5 8.0 7.5 7.0 6.5 6.0 5.5 5.0 4.5 4.0 3.5 3.0 2.5 2.0 1.5 1.0 0.5
f1 (ppm)

CJH-2-64-1-C

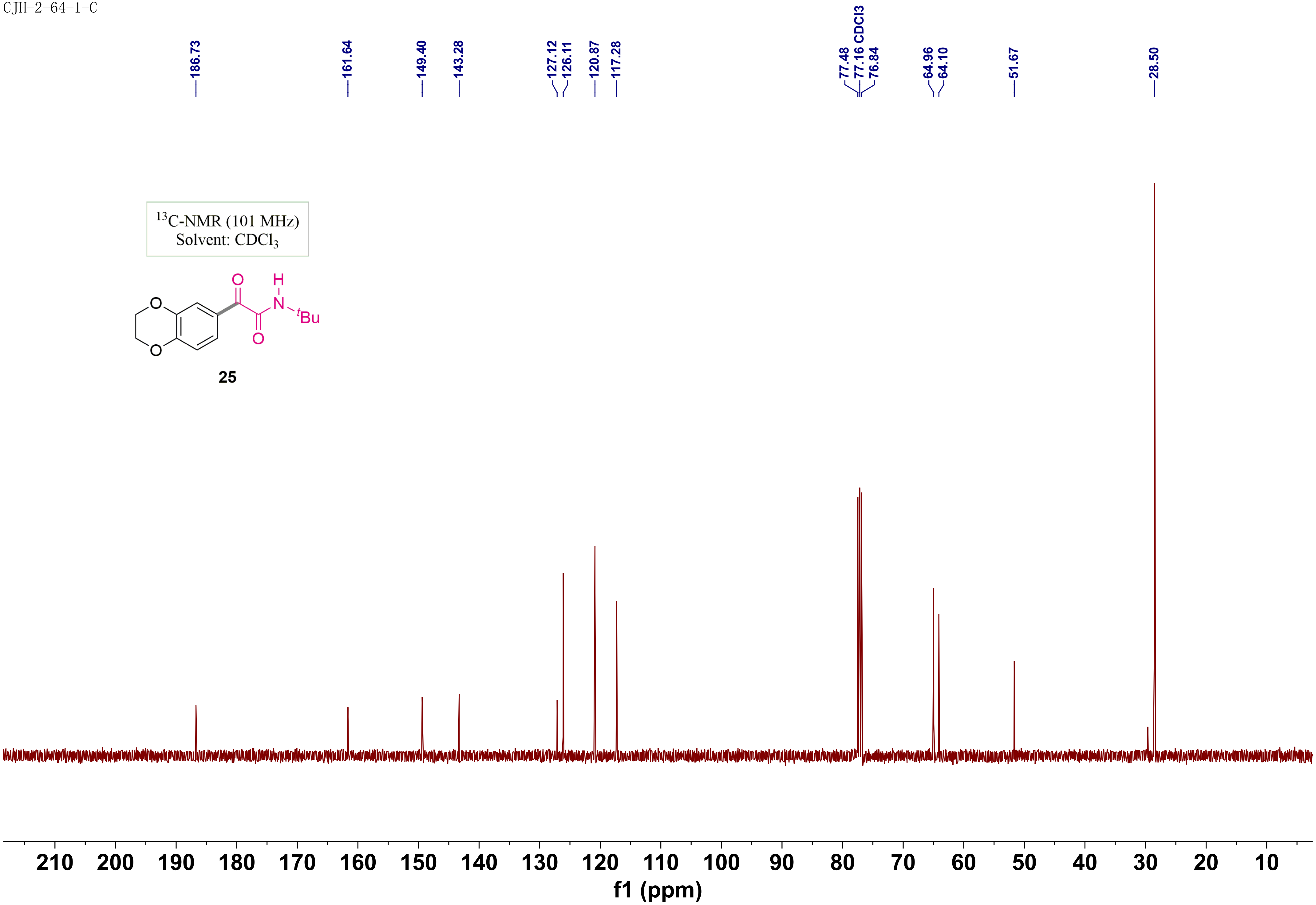

^{13}C-NMR (101 MHz)
Solvent: $CDCl_3$

25

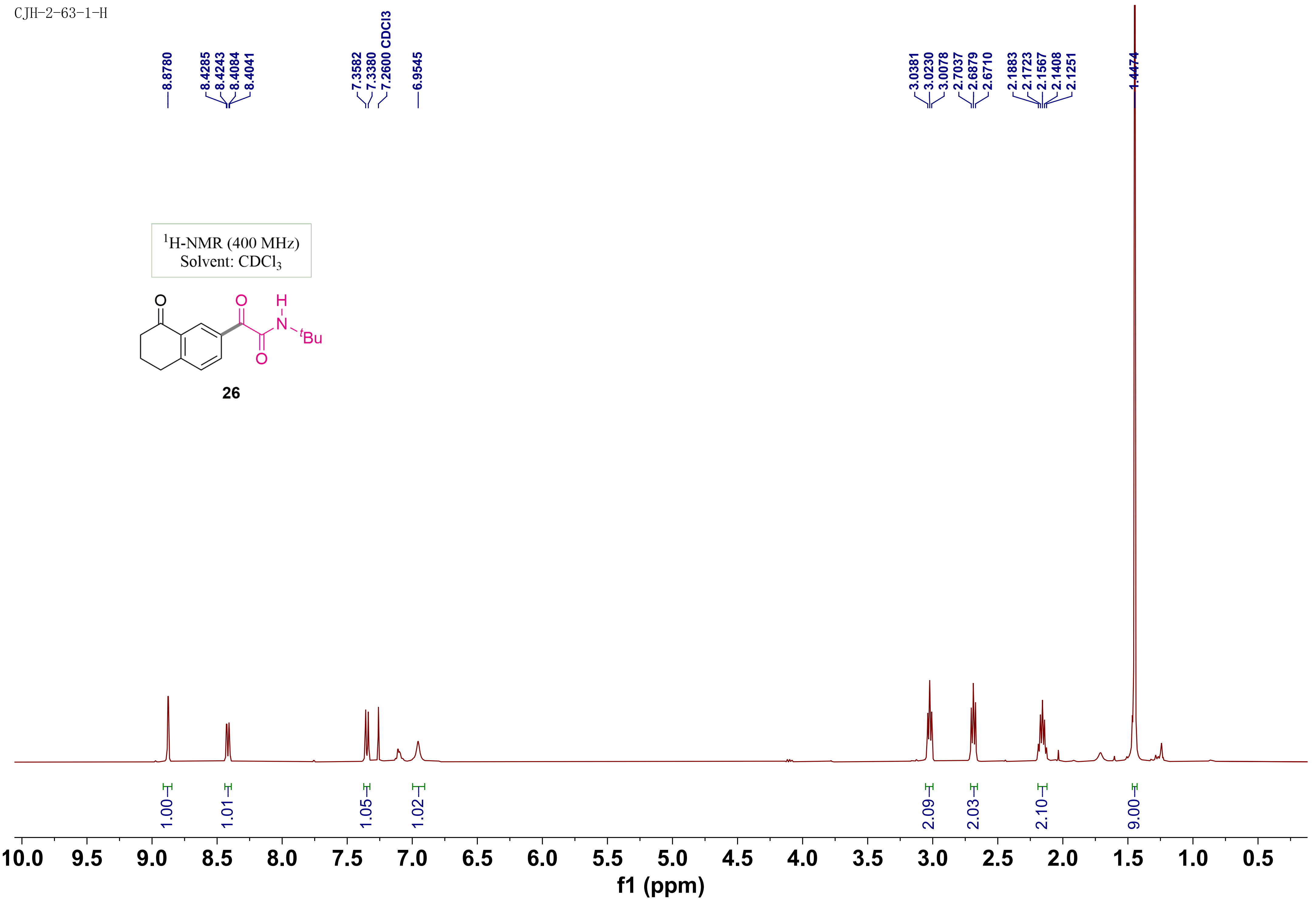
CJH-2-63-1-H
8.8780
8.4285
8.4243
8.4084
8.4041
7.3582
7.3380
7.2600 CDCl3
6.9545
3.0381
3.0230
3.0078
2.7037
2.6879
2.6710
2.1883
2.1723
2.1567
2.1408
2.1251
1.4474
^{1}H-NMR (400 MHz)
Solvent: $CDCl_3$
26
1.00
1.01
1.05
1.02
2.09
2.03
2.10
9.00
10.0 9.5 9.0 8.5 8.0 7.5 7.0 6.5 6.0 5.5 5.0 4.5 4.0 3.5 3.0 2.5 2.0 1.5 1.0 0.5
f1 (ppm)

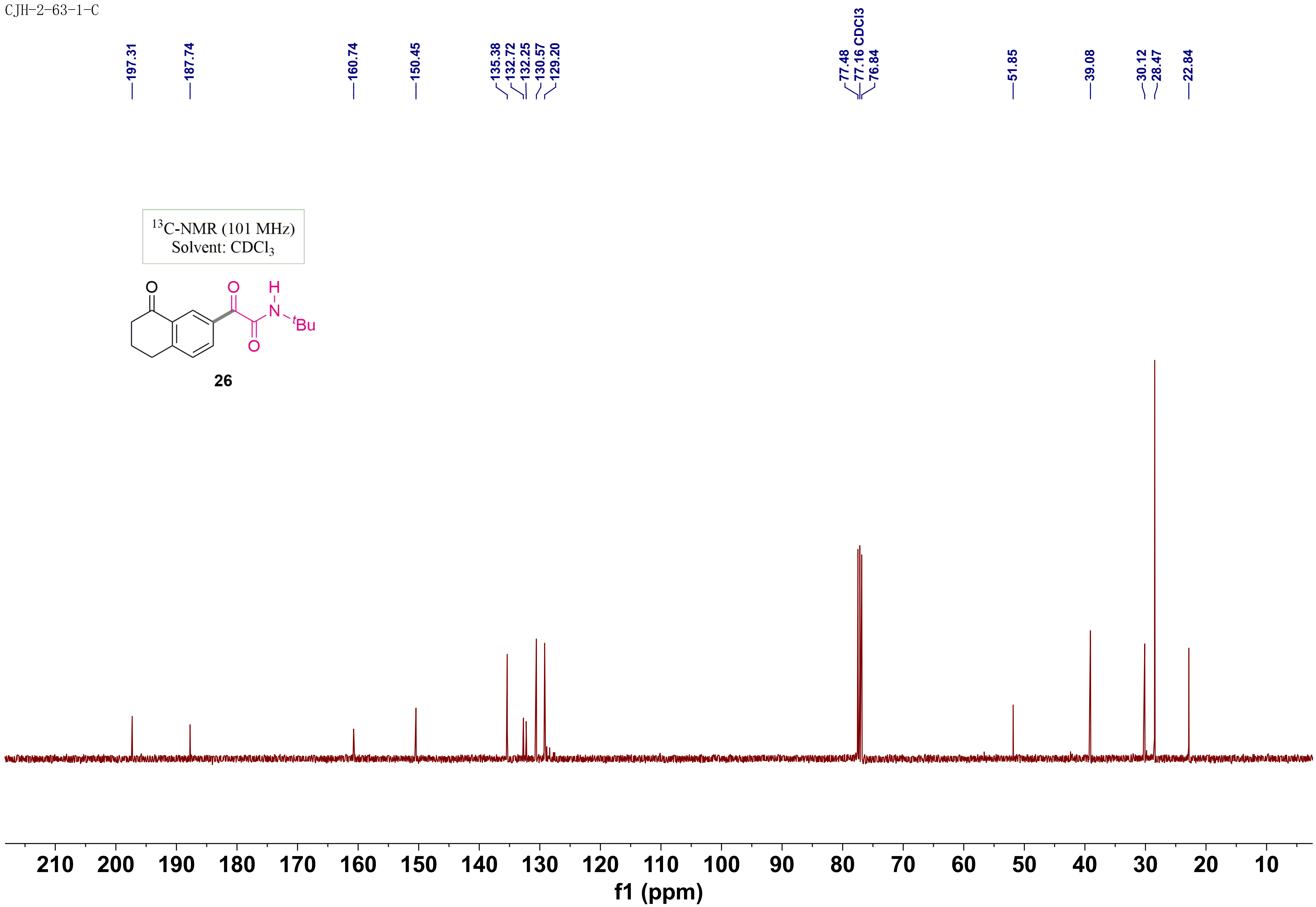
CJH-2-63-1-C
197.31
187.74
160.74
150.45
135.38
132.72
132.25
130.57
129.20
77.48
77.16 CDCl3
76.84
51.85
39.08
30.12
28.47
22.84
^{13}C-NMR (101 MHz)
Solvent: $CDCl_3$
O
O
H
N
^{t}Bu
O
26
210 200 190 180 170 160 150 140 130 120 110 100 90 80 70 60 50 40 30 20 10
f1 (ppm)

CJH-2-64-2-H

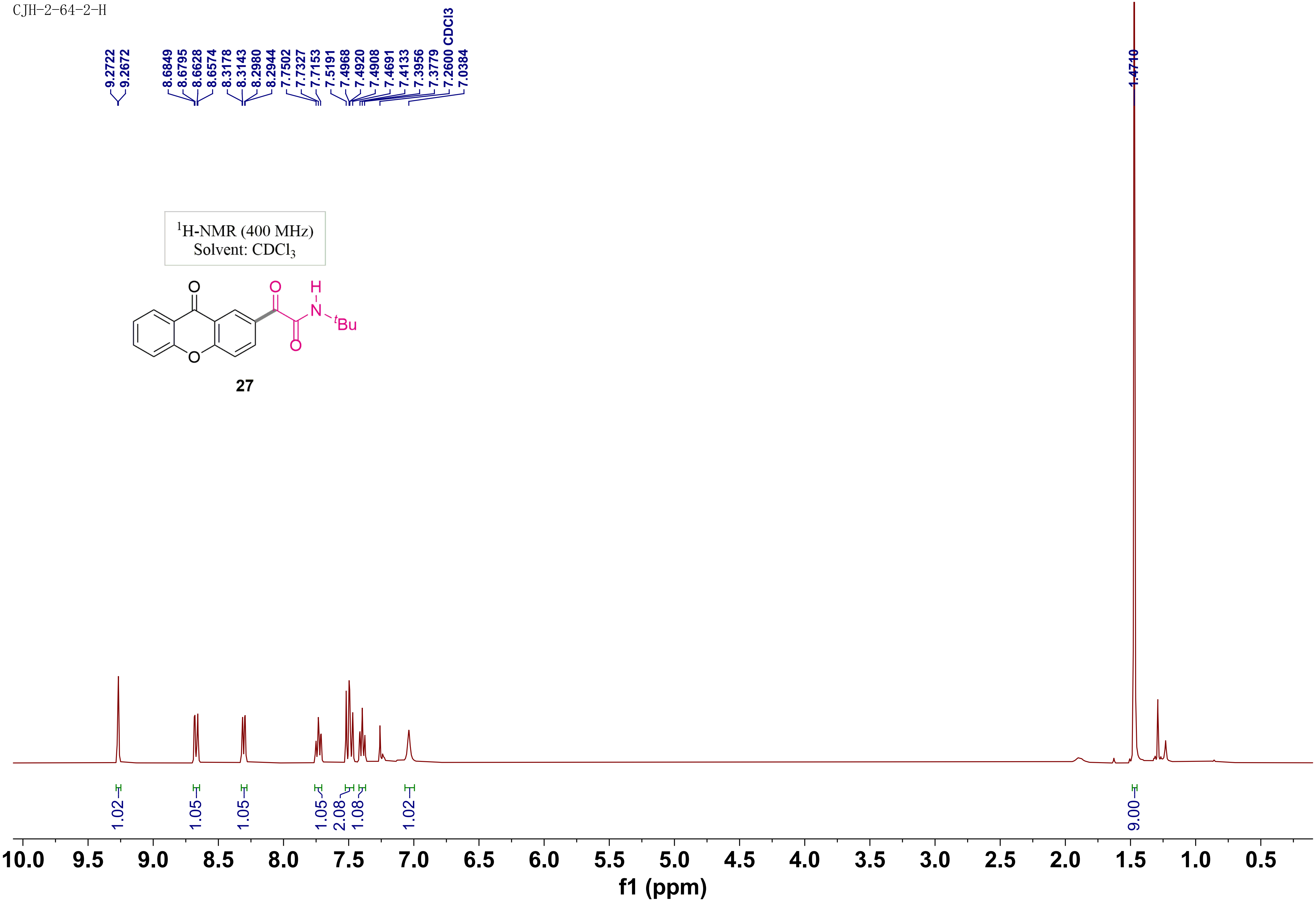

9.2722
9.2672
8.6849
8.6795
8.6628
8.6574
8.3178
8.3143
8.2980
8.2944
7.7502
7.7327
7.7153
7.5191
7.4968
7.4920
7.4908
7.4691
7.4133
7.3956
7.3779
7.2600 CDCl3
7.0384
1.4740
^{1}H-NMR (400 MHz)
Solvent: $CDCl_3$
O
O
H
N
^{t}Bu
O
O
1.02
1.05
1.05
1.05
2.08
1.08
1.02
9.00
10.0 9.5 9.0 8.5 8.0 7.5 7.0 6.5 6.0 5.5 5.0 4.5 4.0 3.5 3.0 2.5 2.0 1.5 1.0 0.5
f1 (ppm)

27

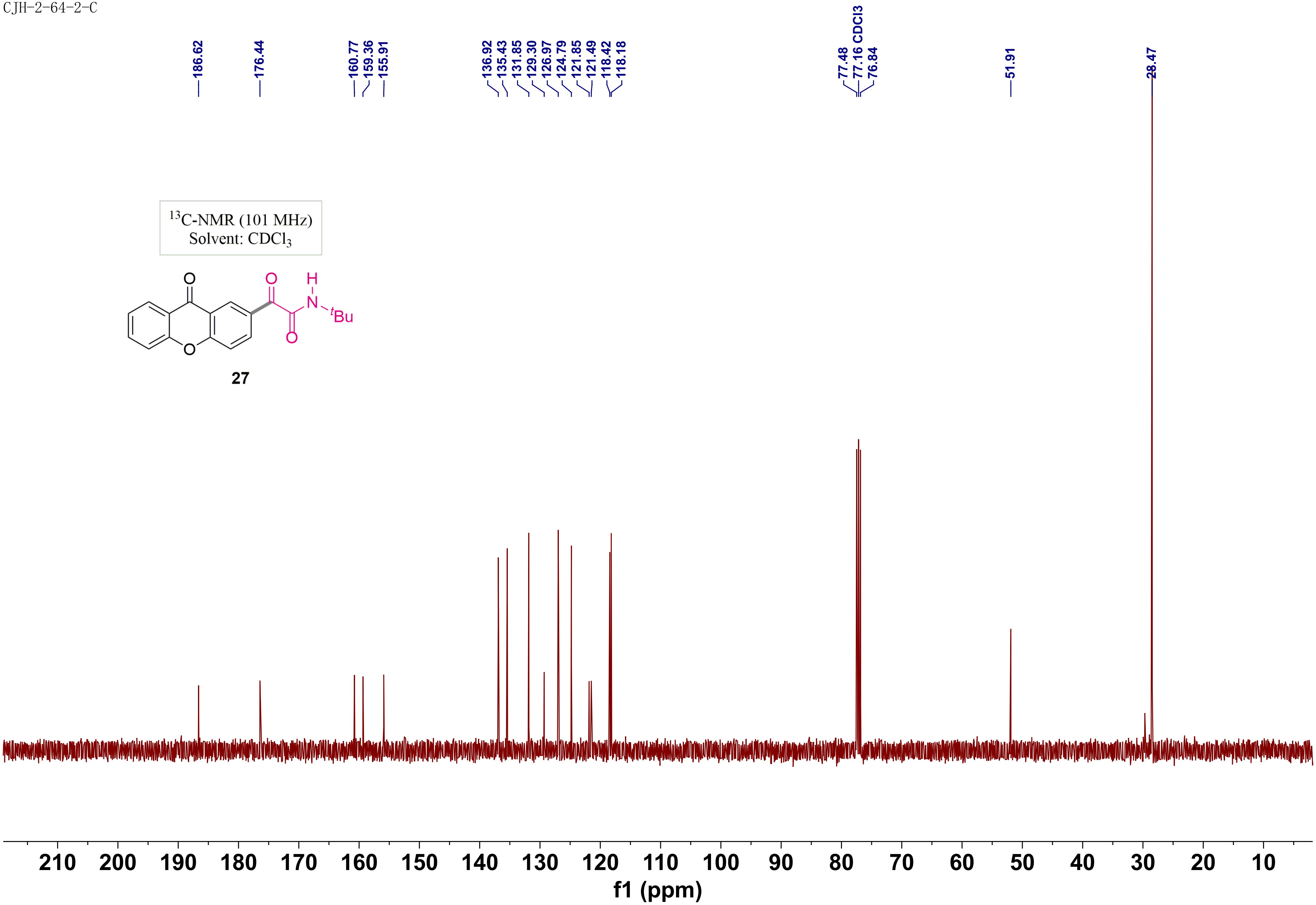
CJH-2-64-2-C
186.62
176.44
160.77
159.36
155.91
136.92
135.43
131.85
129.30
126.97
124.79
121.85
121.49
118.42
118.18
77.48
77.16 CDCl3
76.84
51.91
28.47
^{13}C-NMR (101 MHz)
Solvent: $CDCl_3$
O
O
H
N
^{t}Bu
O
O
27
210 200 190 180 170 160 150 140 130 120 110 100 90 80 70 60 50 40 30 20 10
f1 (ppm)

CJH-2-62-3-H

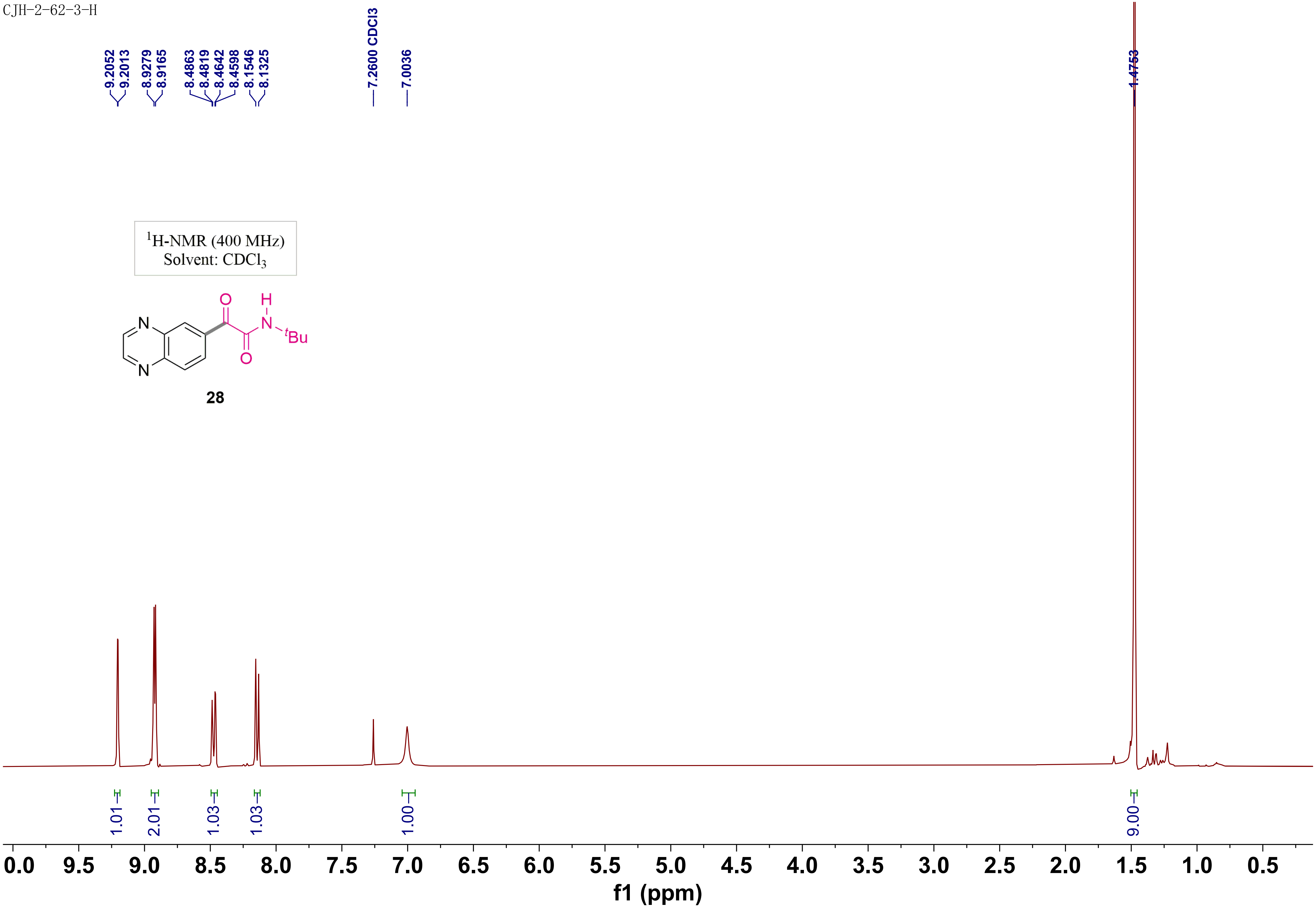
9.2052
9.2013
8.9279
8.9165
8.4863
8.4819
8.4642
8.4598
8.1546
8.1325
7.2600 CDCl3
7.0036
1.4753
^{1}H-NMR (400 MHz)
Solvent: $CDCl_3$
N
N
O
H
N
^{t}Bu
O
28
1.01
2.01
1.03
1.03
1.00
9.00
10.0 9.5 9.0 8.5 8.0 7.5 7.0 6.5 6.0 5.5 5.0 4.5 4.0 3.5 3.0 2.5 2.0 1.5 1.0 0.5
f1 (ppm)

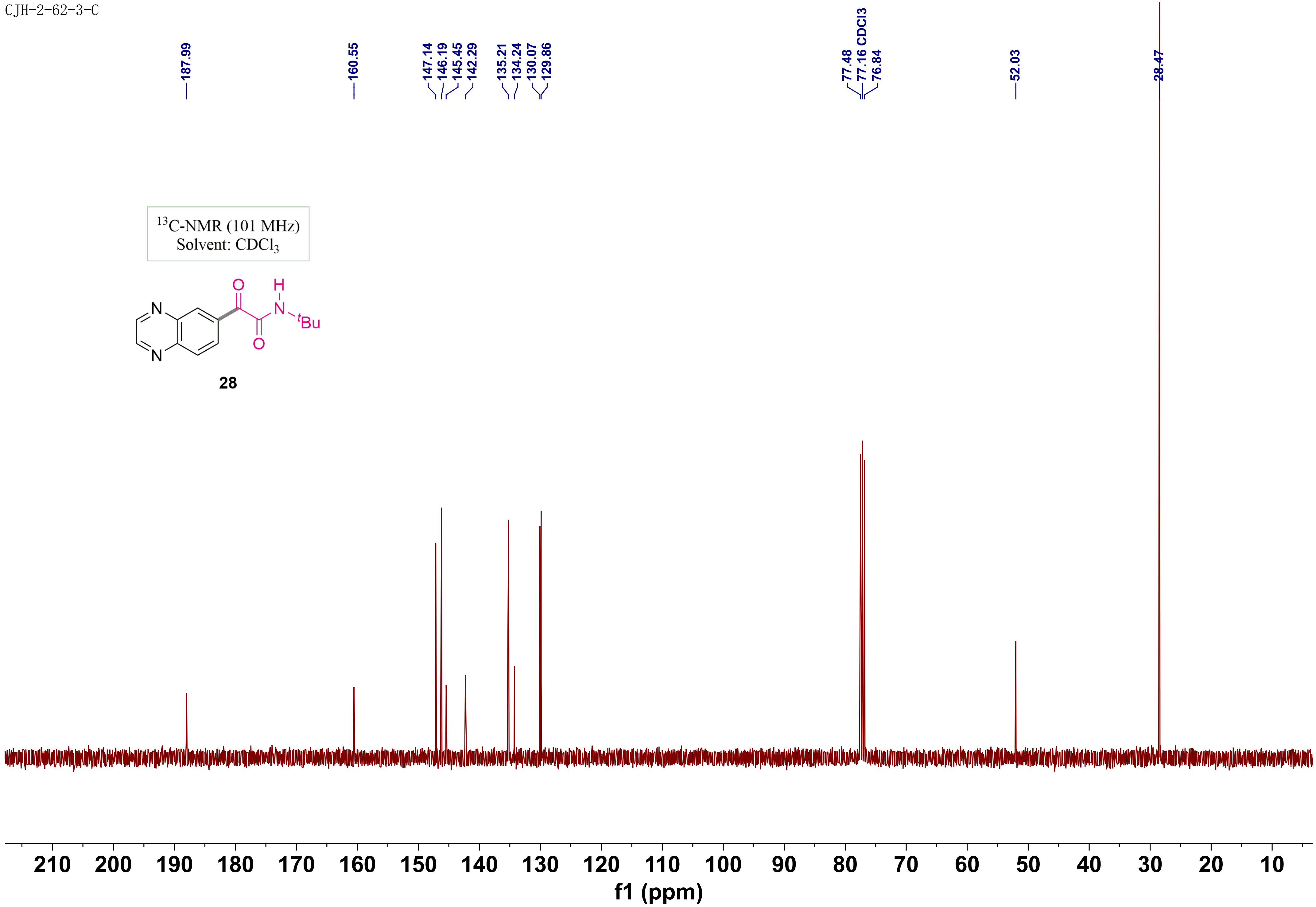
CJH-2-62-3-C
187.99
160.55
147.14
146.19
145.45
142.29
135.21
134.24
130.07
129.86
77.48
77.16 CDCl3
76.84
52.03
28.47
13C-NMR (101 MHz)
Solvent: CDCl3
N
N
O
H
N
tBu
O
28
210
200
190
180
170
160
150
140
130
120
110
100
90
80
70
60
50
40
30
20
10
f1 (ppm)

CJH-1-114-2-H

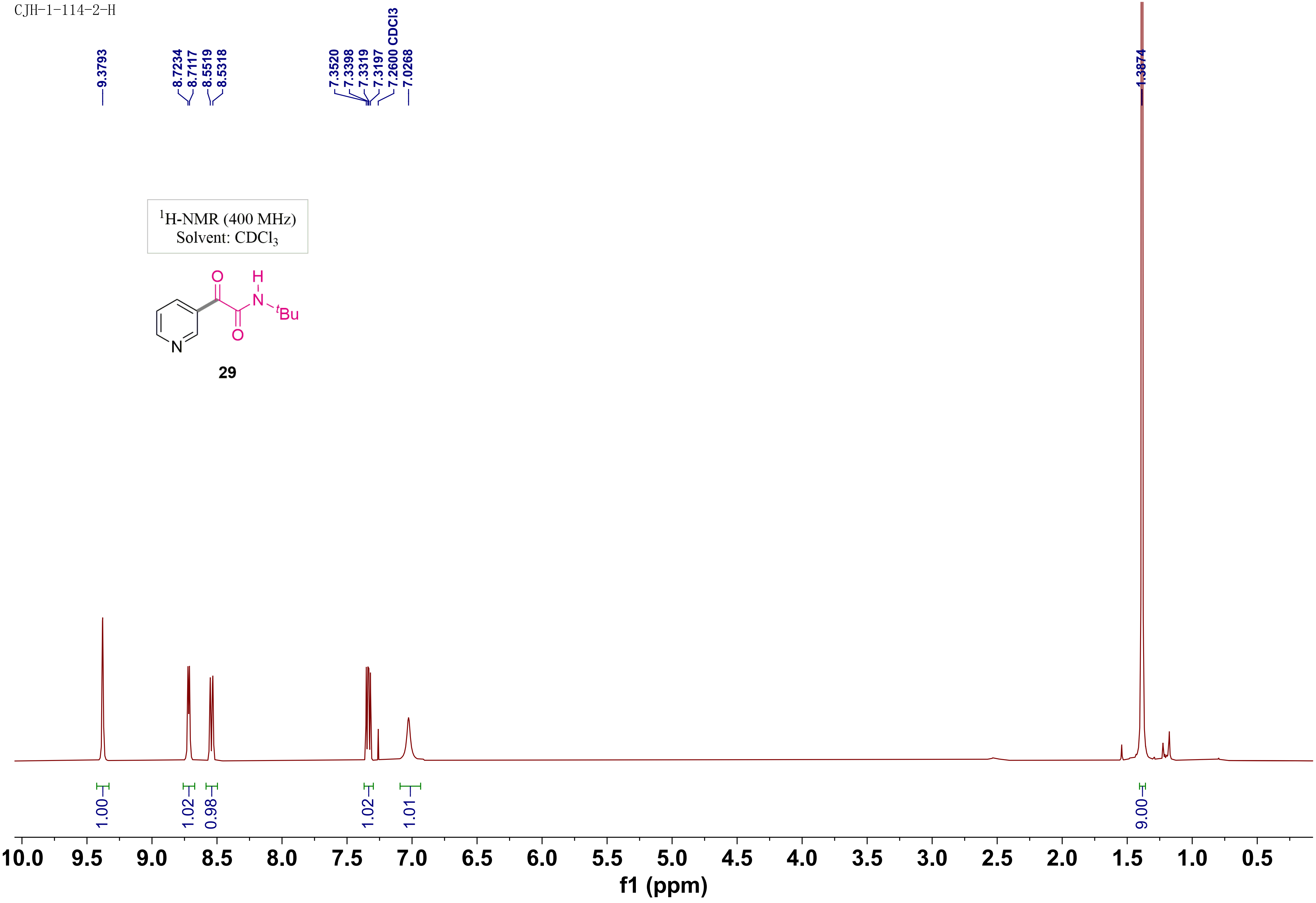
9.3793
8.7234
8.7117
8.5519
8.5318
7.3520
7.3398
7.3319
7.3197
7.2600 CDCl3
7.0268
1.3874
1H-NMR (400 MHz)
Solvent: CDCl3
O
H
N
tBu
O
N
29
1.00
1.02
0.98
1.02
1.01
9.00
10.0
9.5
9.0
8.5
8.0
7.5
7.0
6.5
6.0
5.5
5.0
4.5
4.0
3.5
3.0
2.5
2.0
1.5
1.0
0.5
f1 (ppm)

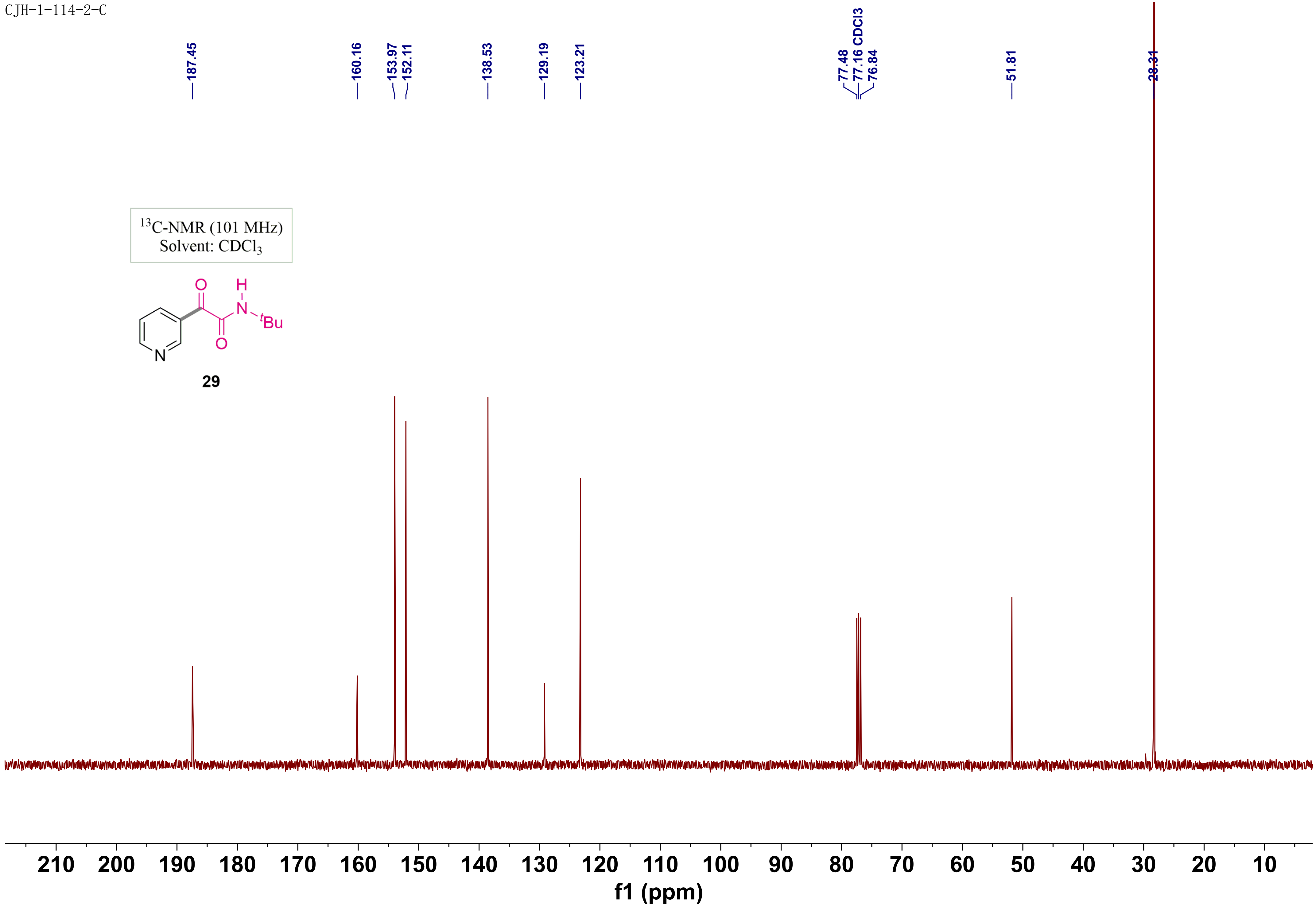
CJH-1-114-2-C
187.45
160.16
153.97
152.11
138.53
129.19
123.21
77.48
77.16 CDCl3
76.84
51.81
28.31
^{13}C-NMR (101 MHz)
Solvent: $CDCl_3$
O
H
N
^{t}Bu
O
N
29
210
200
190
180
170
160
150
140
130
120
110
100
90
80
70
60
50
40
30
20
10
f1 (ppm)

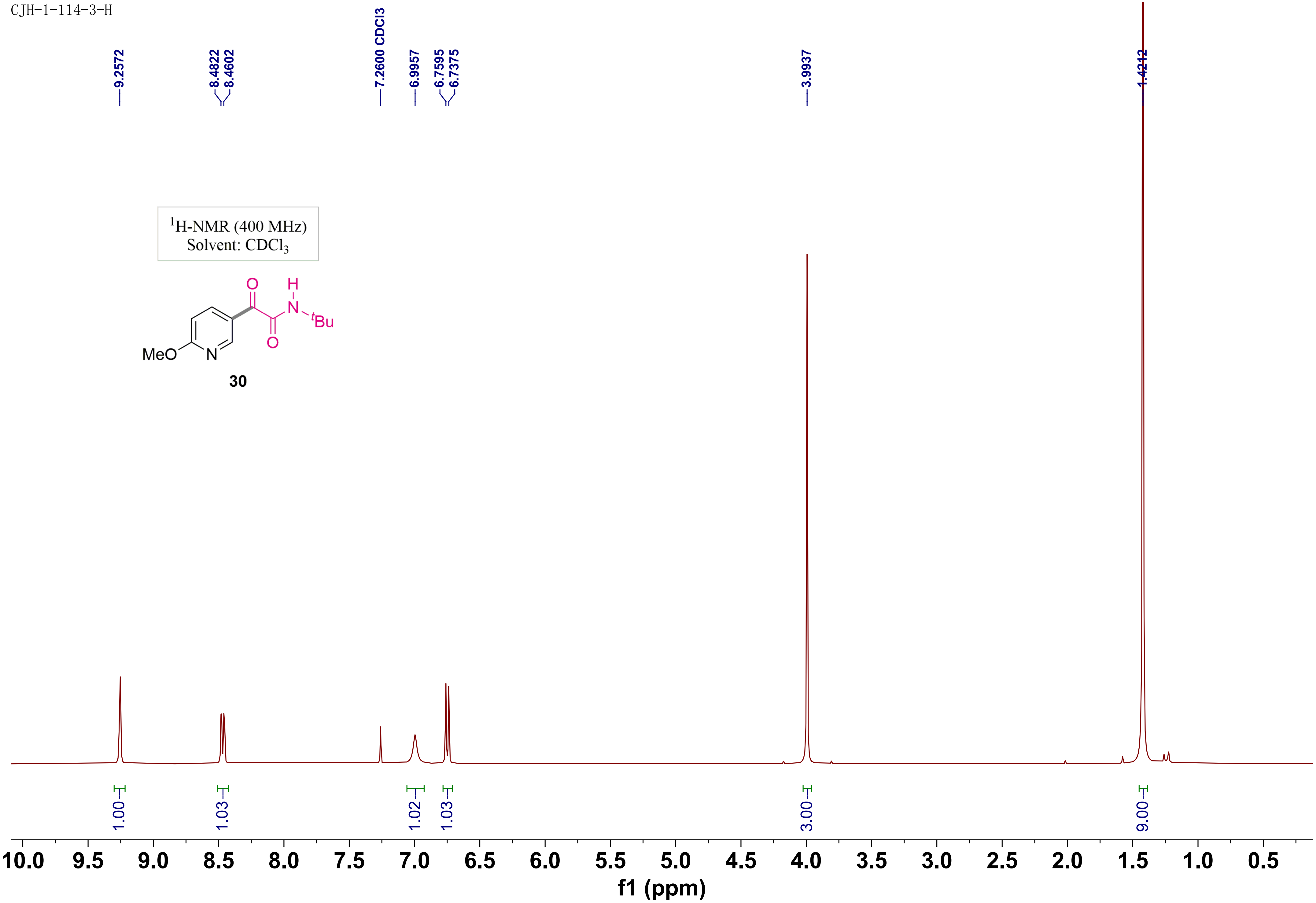
CJH-1-114-3-H
9.2572
8.4822
8.4602
7.2600 CDCl3
6.9957
6.7595
6.7375
3.9937
1.4212
^{1}H-NMR (400 MHz)
Solvent: $CDCl_3$
MeO
N
O
H
N
^{t}Bu
O
30
1.00
1.03
1.02
1.03
3.00
9.00
10.0
9.5
9.0
8.5
8.0
7.5
7.0
6.5
6.0
5.5
5.0
4.5
4.0
3.5
3.0
2.5
2.0
1.5
1.0
0.5
f1 (ppm)

CJH-1-114-3-C

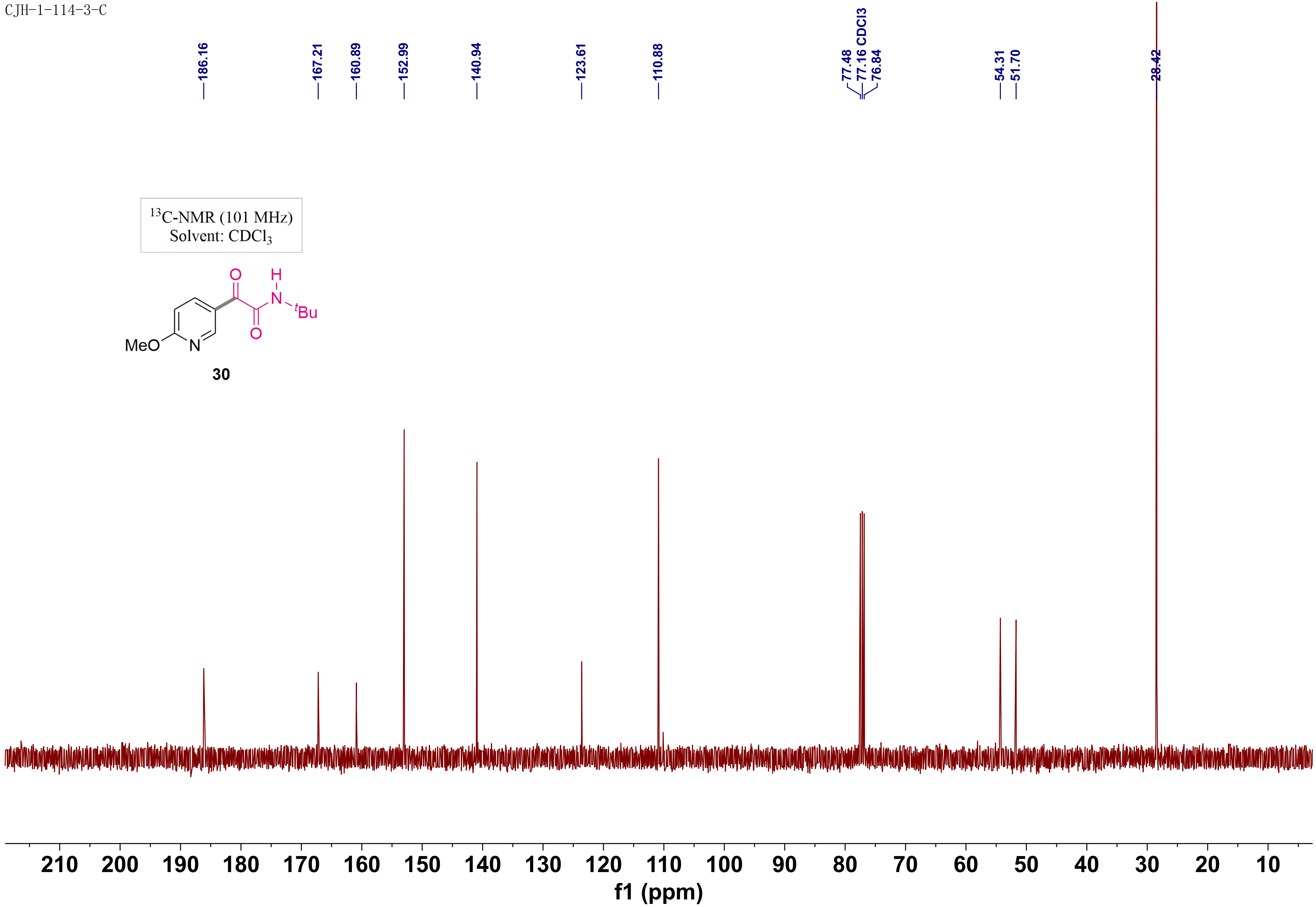
186.16
167.21
160.89
152.99
140.94
123.61
110.88
77.48
77.16 CDCl3
76.84
54.31
51.70
28.42
13C-NMR (101 MHz)
Solvent: CDCl3
MeO
N
O
H
N
tBu
O
30
210 200 190 180 170 160 150 140 130 120 110 100 90 80 70 60 50 40 30 20 10
f1 (ppm)

CJH-2-67-3-H

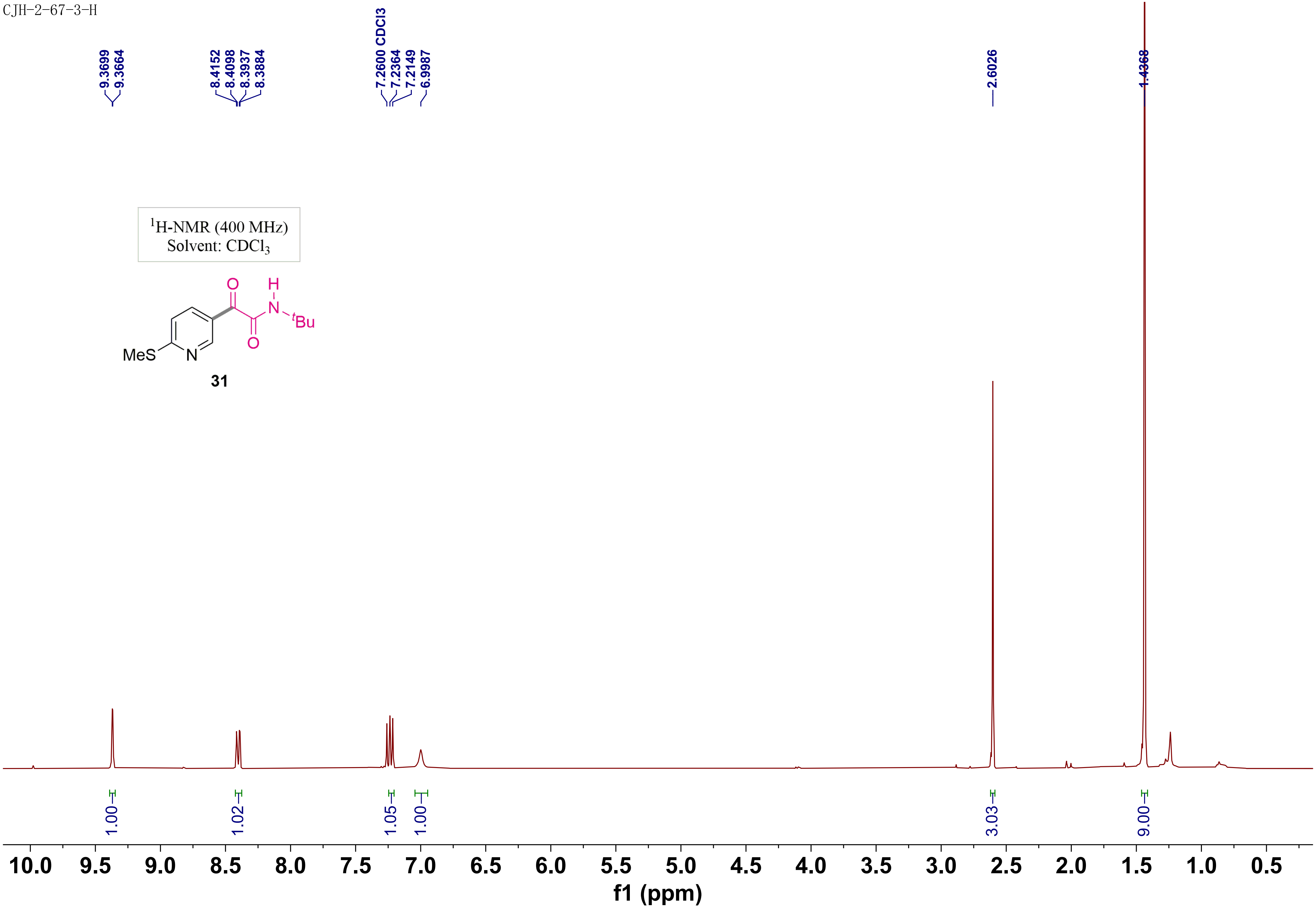

^{1}H-NMR (400 MHz)
Solvent: $CDCl_3$

31

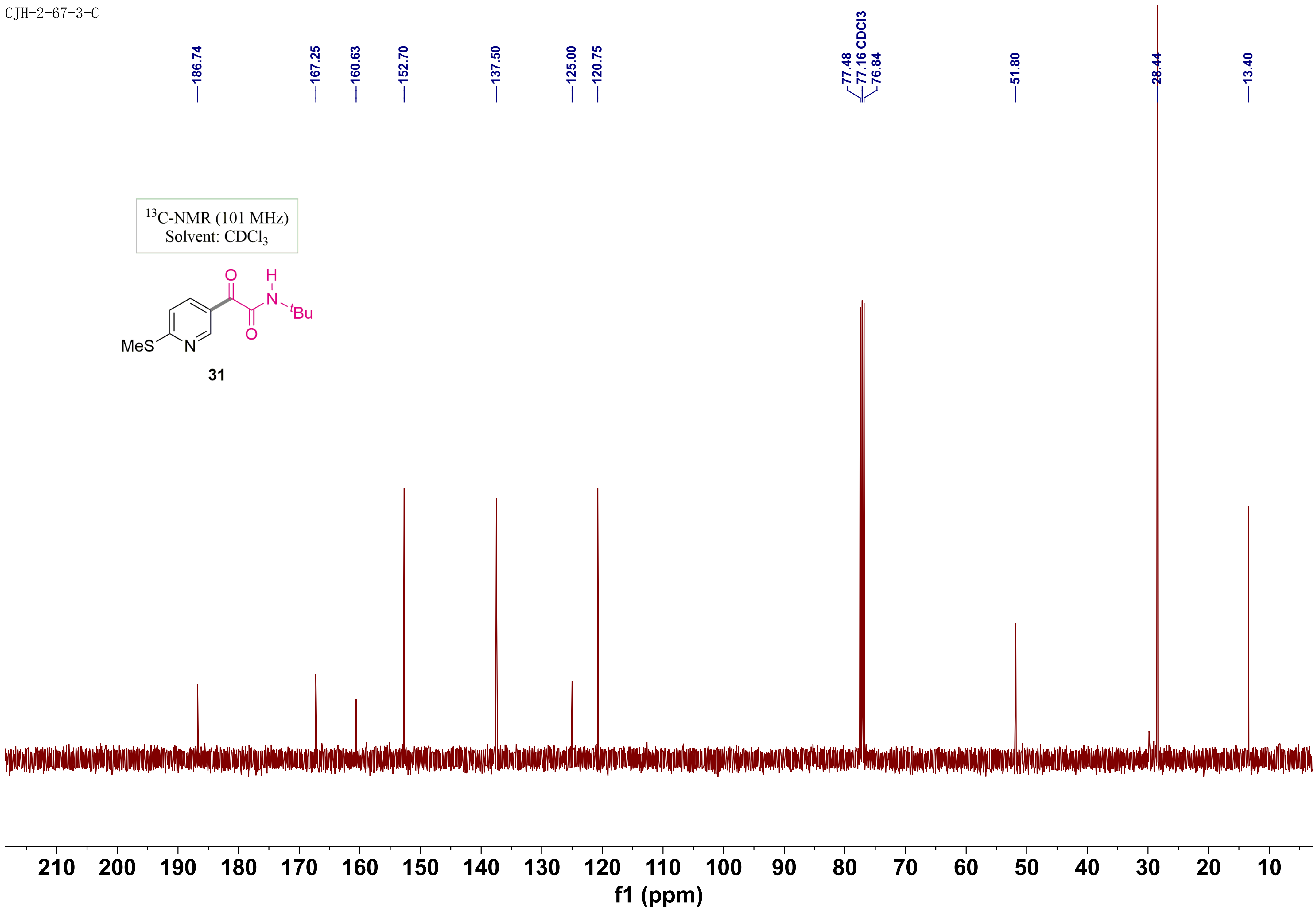

CJH-2-67-3-C
186.74
167.25
160.63
152.70
137.50
125.00
120.75
77.48
77.16 CDCl3
76.84
51.80
28.44
13.40
^{13}C-NMR (101 MHz)
Solvent: $CDCl_3$
O
H
N
tBu
MeS
N
O
31
210 200 190 180 170 160 150 140 130 120 110 100 90 80 70 60 50 40 30 20 10
f1 (ppm)

CJH-1-117-3-H

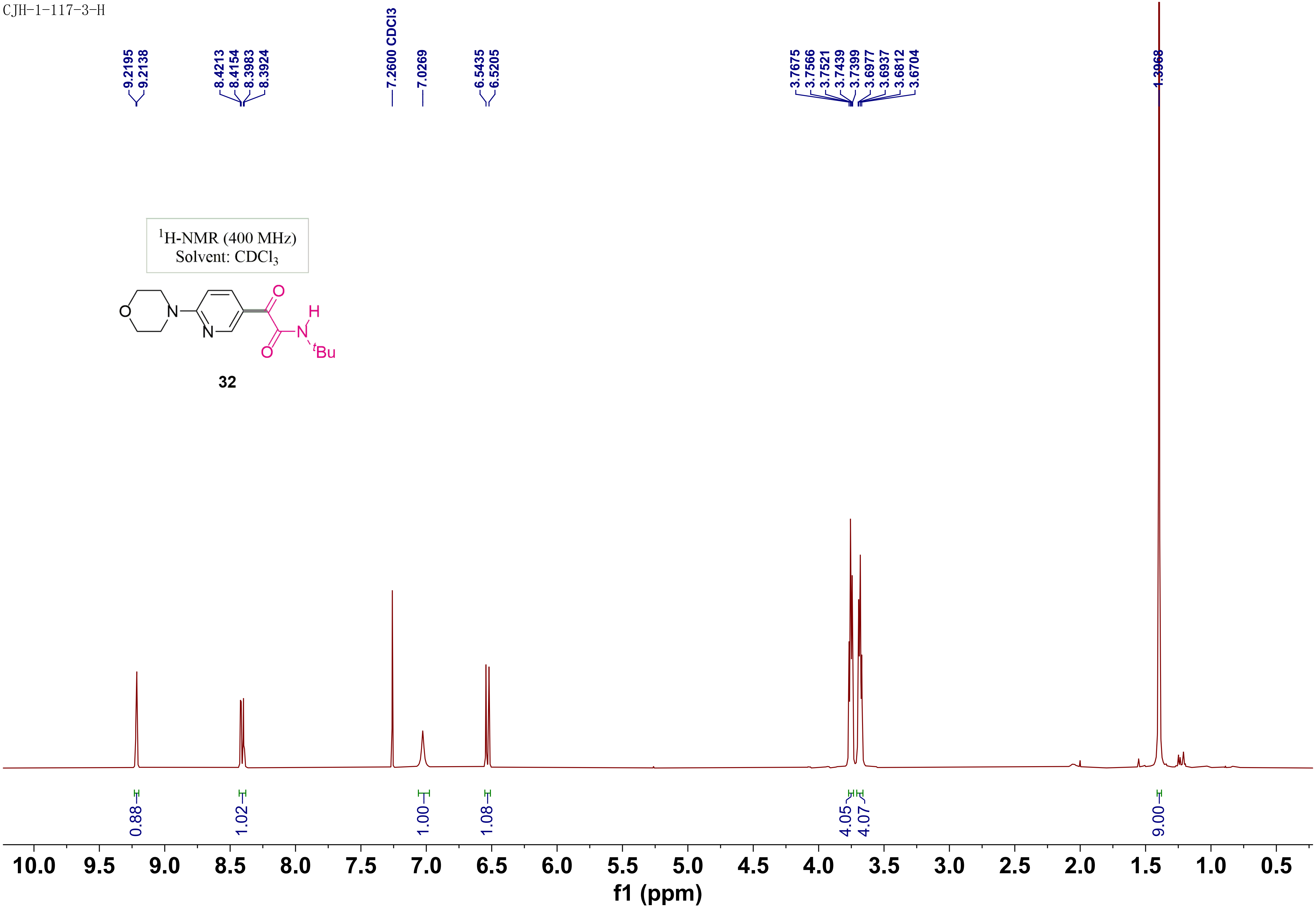

32

CJH-1-117-3-C

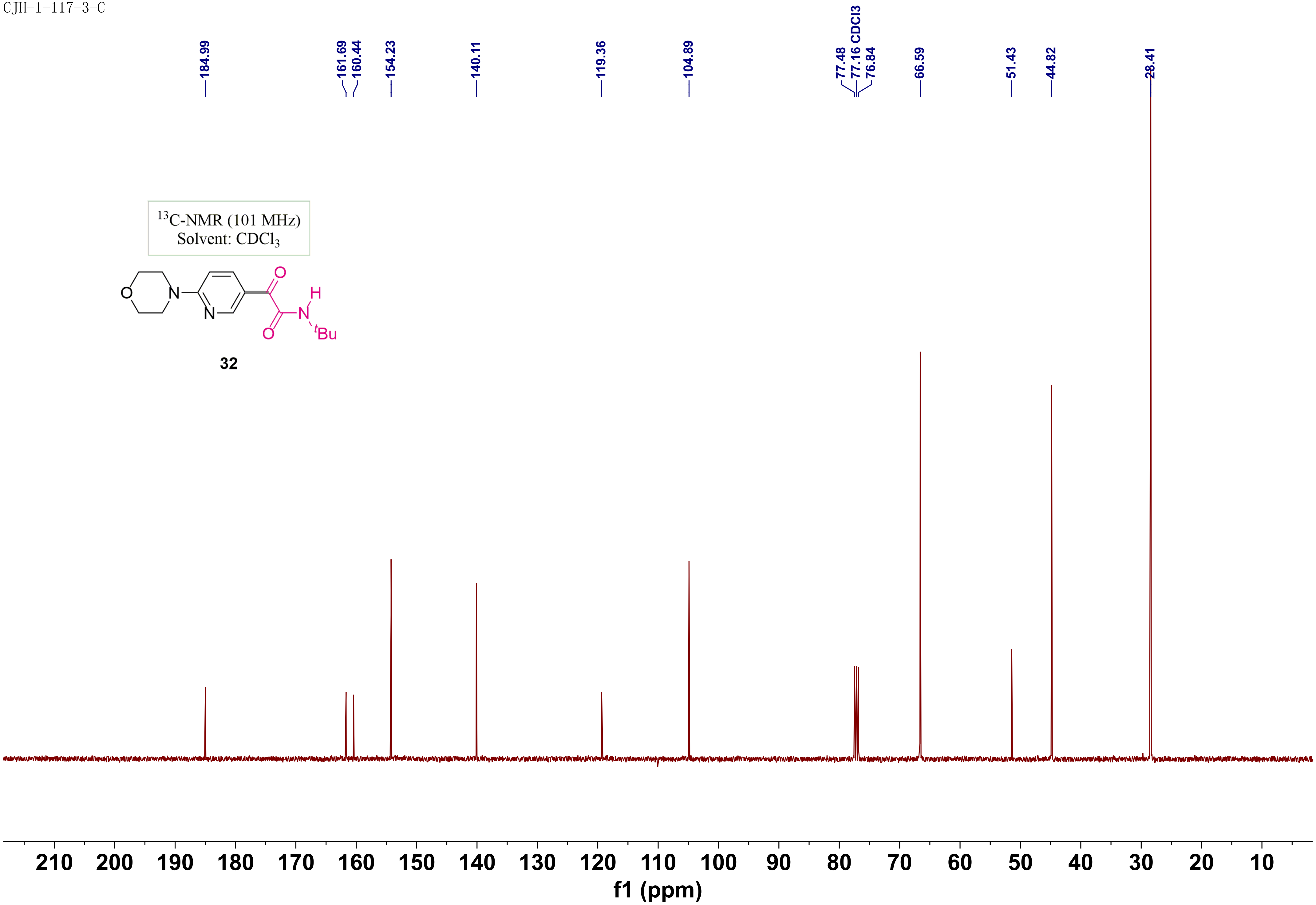
184.99
161.69
160.44
154.23
140.11
119.36
104.89
77.48
77.16 CDCl3
76.84
66.59
51.43
44.82
28.41
^{13}C-NMR (101 MHz)
Solvent: $CDCl_3$
O
N
N
O
H
N
O
^{t}Bu
32
210 200 190 180 170 160 150 140 130 120 110 100 90 80 70 60 50 40 30 20 10
f1 (ppm)

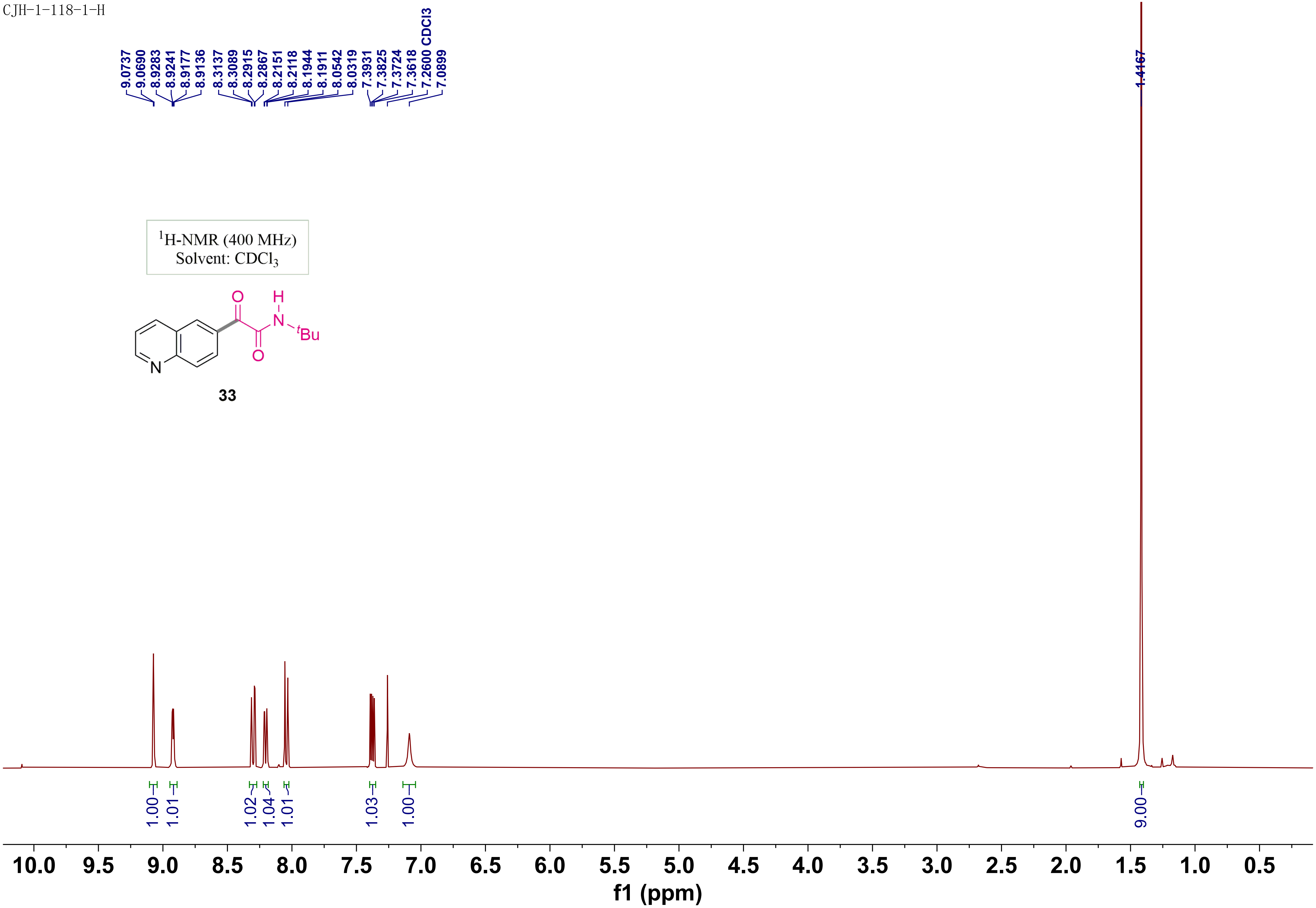
CJH-1-118-1-H
9.0737
9.0690
8.9283
8.9241
8.9177
8.9136
8.3137
8.3089
8.2915
8.2867
8.2151
8.2118
8.1944
8.1911
8.0542
8.0319
7.3931
7.3825
7.3724
7.3618
7.2600 CDCl3
7.0899
1.4167
^{1}H-NMR (400 MHz)
Solvent: $CDCl_3$
O
H
N
^{t}Bu
O
N
33
1.00
1.01
1.02
1.04
1.01
1.03
1.00
9.00
10.0
9.5
9.0
8.5
8.0
7.5
7.0
6.5
6.0
5.5
5.0
4.5
4.0
3.5
3.0
2.5
2.0
1.5
1.0
0.5
f1 (ppm)

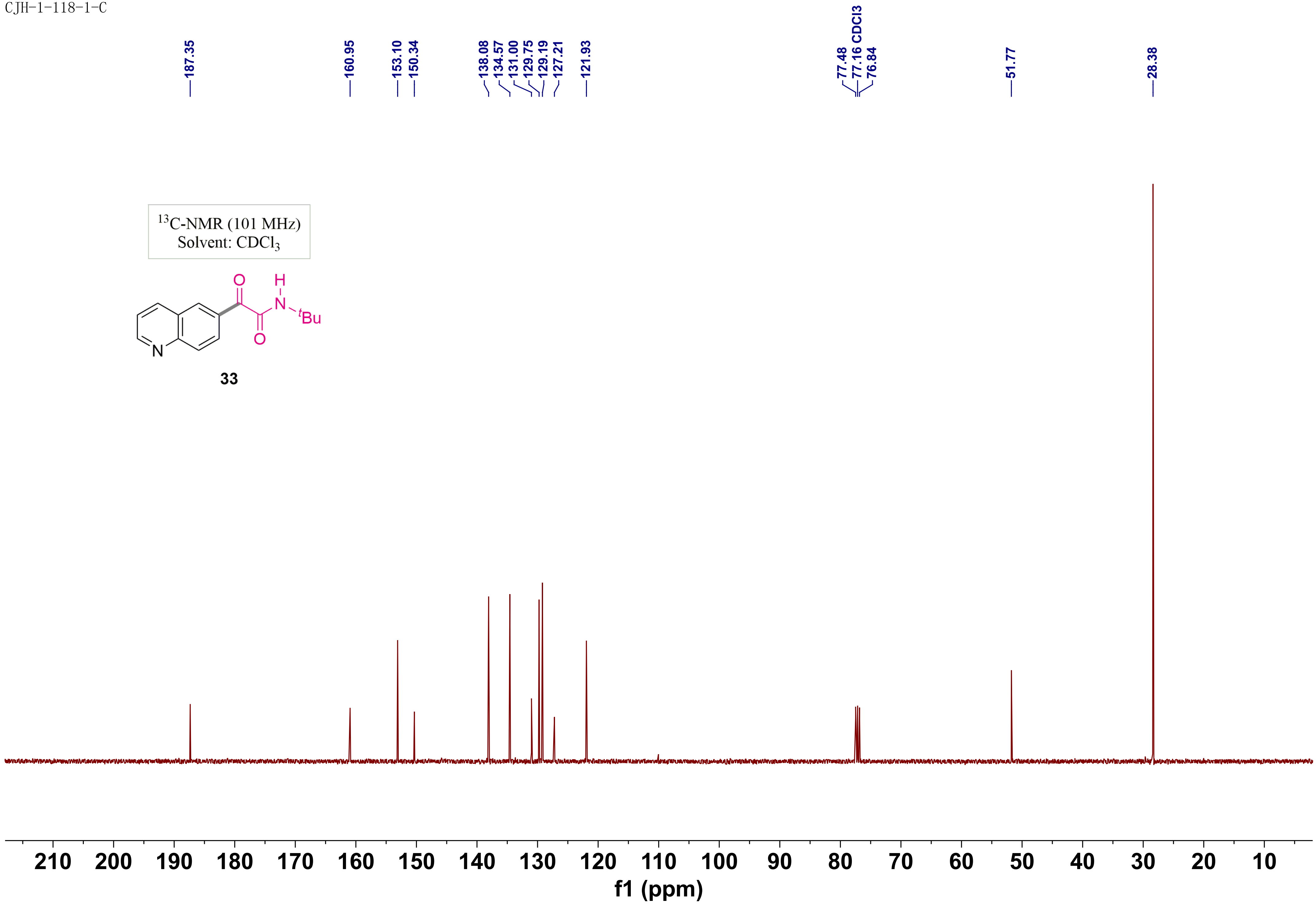
CJH-1-118-1-C
187.35
160.95
153.10
150.34
138.08
134.57
131.00
129.75
129.19
127.21
121.93
77.48
77.16 CDCl3
76.84
51.77
28.38
^{13}C-NMR (101 MHz)
Solvent: $CDCl_3$
O
H
N
tBu
O
N
33
210 200 190 180 170 160 150 140 130 120 110 100 90 80 70 60 50 40 30 20 10
f1 (ppm)

CJH-1-120-2-H

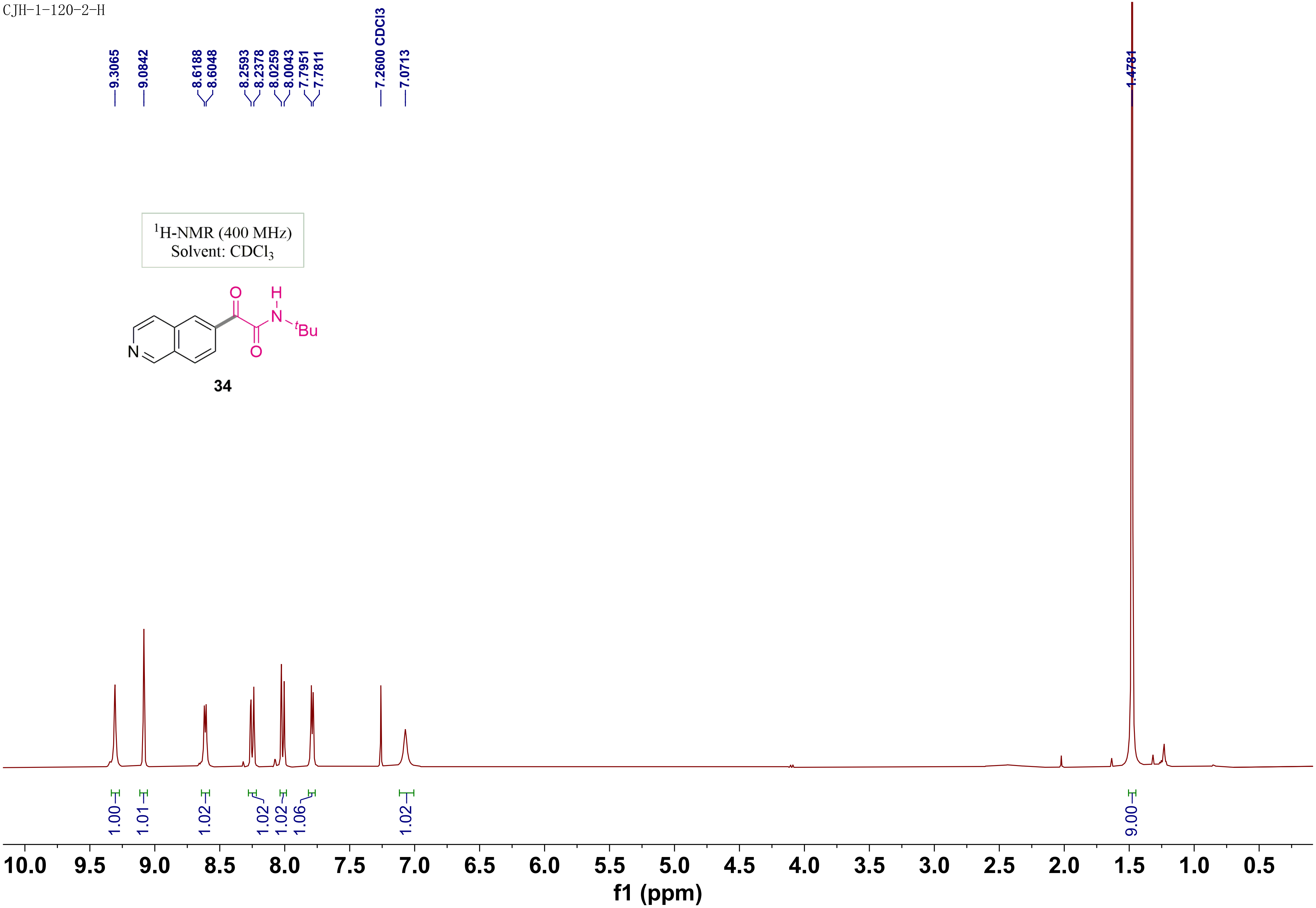

9.3065
9.0842
8.6188
8.6048
8.2593
8.2378
8.0259
8.0043
7.7951
7.7811
7.2600 CDCl3
7.0713
1.4781
¹H-NMR (400 MHz)
Solvent: CDCl3
34
1.00
1.01
1.02
1.02
1.02
1.06
1.02
9.00
10.0 9.5 9.0 8.5 8.0 7.5 7.0 6.5 6.0 5.5 5.0 4.5 4.0 3.5 3.0 2.5 2.0 1.5 1.0 0.5
f1 (ppm)

CJH-1-120-2-C

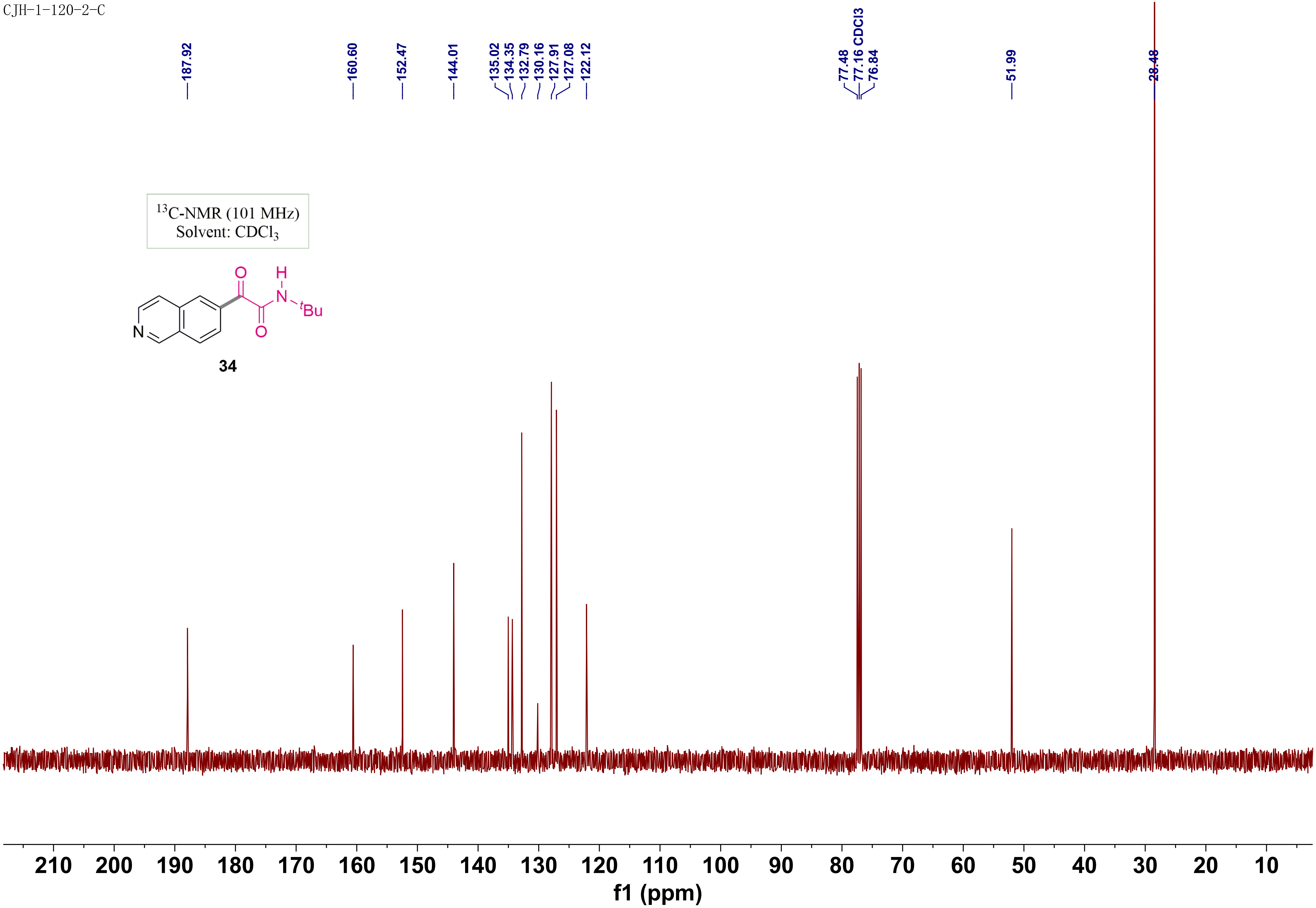

^{13}C-NMR (101 MHz)
Solvent: $CDCl_3$

34

CJH-1-111-3-H

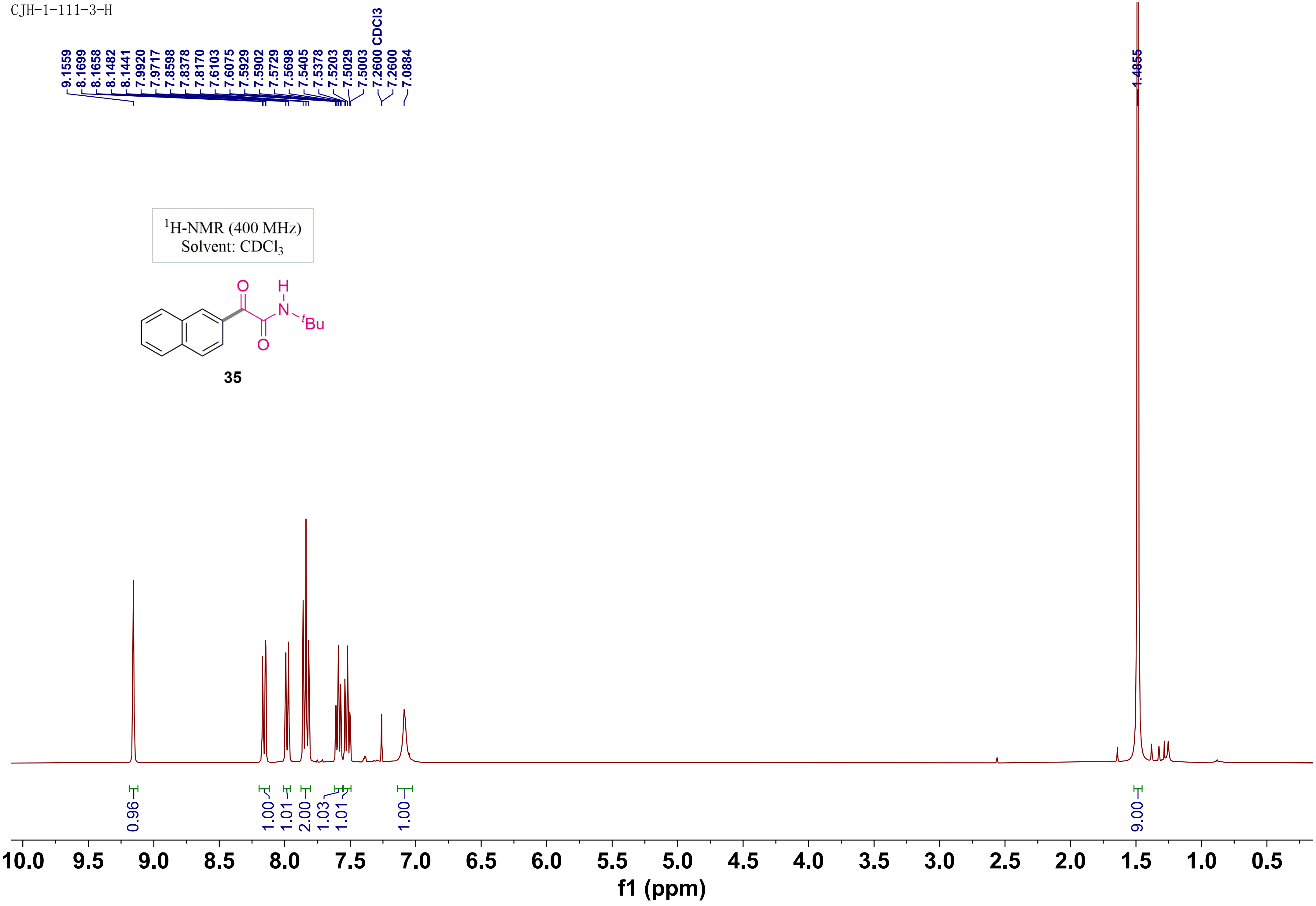
9.1559
8.1699
8.1658
8.1482
8.1441
7.9920
7.9717
7.8598
7.8378
7.8170
7.6103
7.6075
7.5929
7.5902
7.5729
7.5698
7.5405
7.5378
7.5203
7.5029
7.5003
7.2600 CDCl3
7.2600
7.0884
1.4855
^{1}H-NMR (400 MHz)
Solvent: $CDCl_3$
O
H
N
^{t}Bu
O
35
0.96
1.00
1.01
2.00
1.03
1.01
1.00
9.00
10.0
9.5
9.0
8.5
8.0
7.5
7.0
6.5
6.0
5.5
5.0
4.5
4.0
3.5
3.0
2.5
2.0
1.5
1.0
0.5
f1 (ppm)

CJH-1-111-3-C

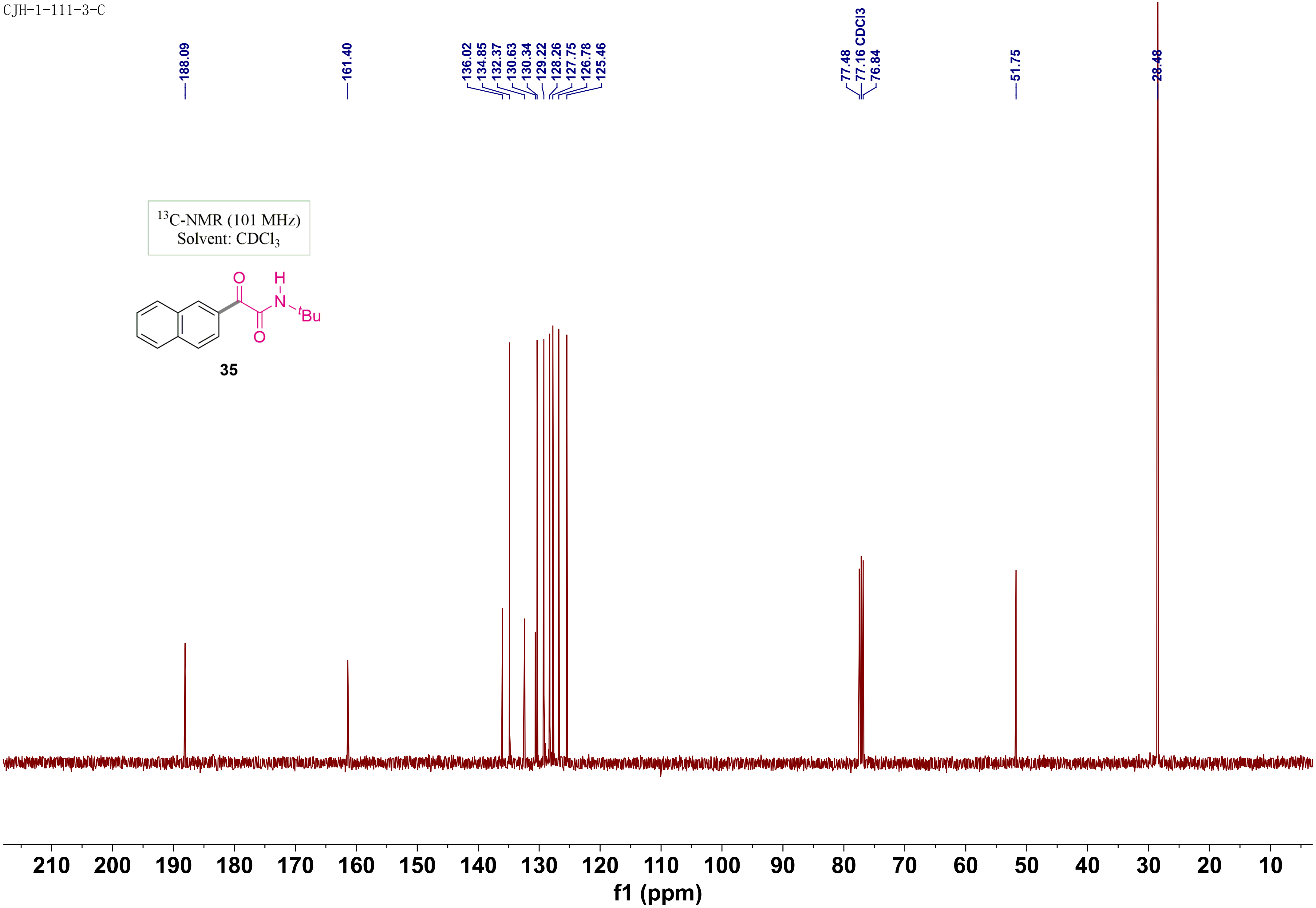

^{13}C-NMR (101 MHz)
Solvent: $CDCl_3$

35

CJH-2-61-1-H

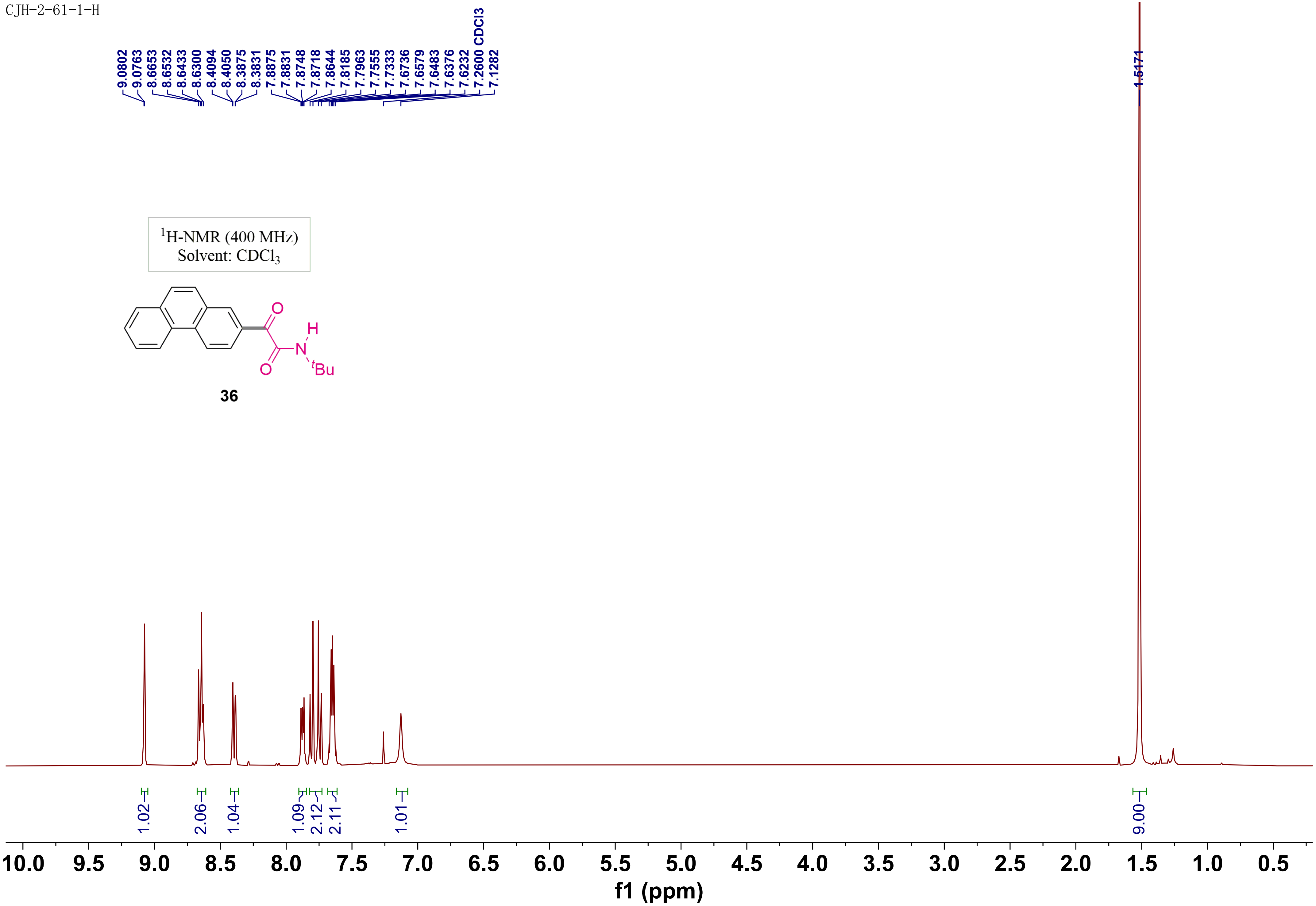
9.0802
9.0763
8.6653
8.6532
8.6433
8.6300
8.4094
8.4050
8.3875
8.3831
7.8875
7.8831
7.8748
7.8718
7.8644
7.8185
7.7963
7.7555
7.7333
7.6736
7.6579
7.6483
7.6376
7.6232
7.2600 CDCl3
7.1282
1.5171
^{1}H-NMR (400 MHz)
Solvent: $CDCl_3$
O
H
N
O
^{t}Bu
36
1.02
2.06
1.04
1.09
2.12
2.11
1.01
9.00
10.0
9.5
9.0
8.5
8.0
7.5
7.0
6.5
6.0
5.5
5.0
4.5
4.0
3.5
3.0
2.5
2.0
1.5
1.0
0.5
f1 (ppm)

CJH-2-61-1-C

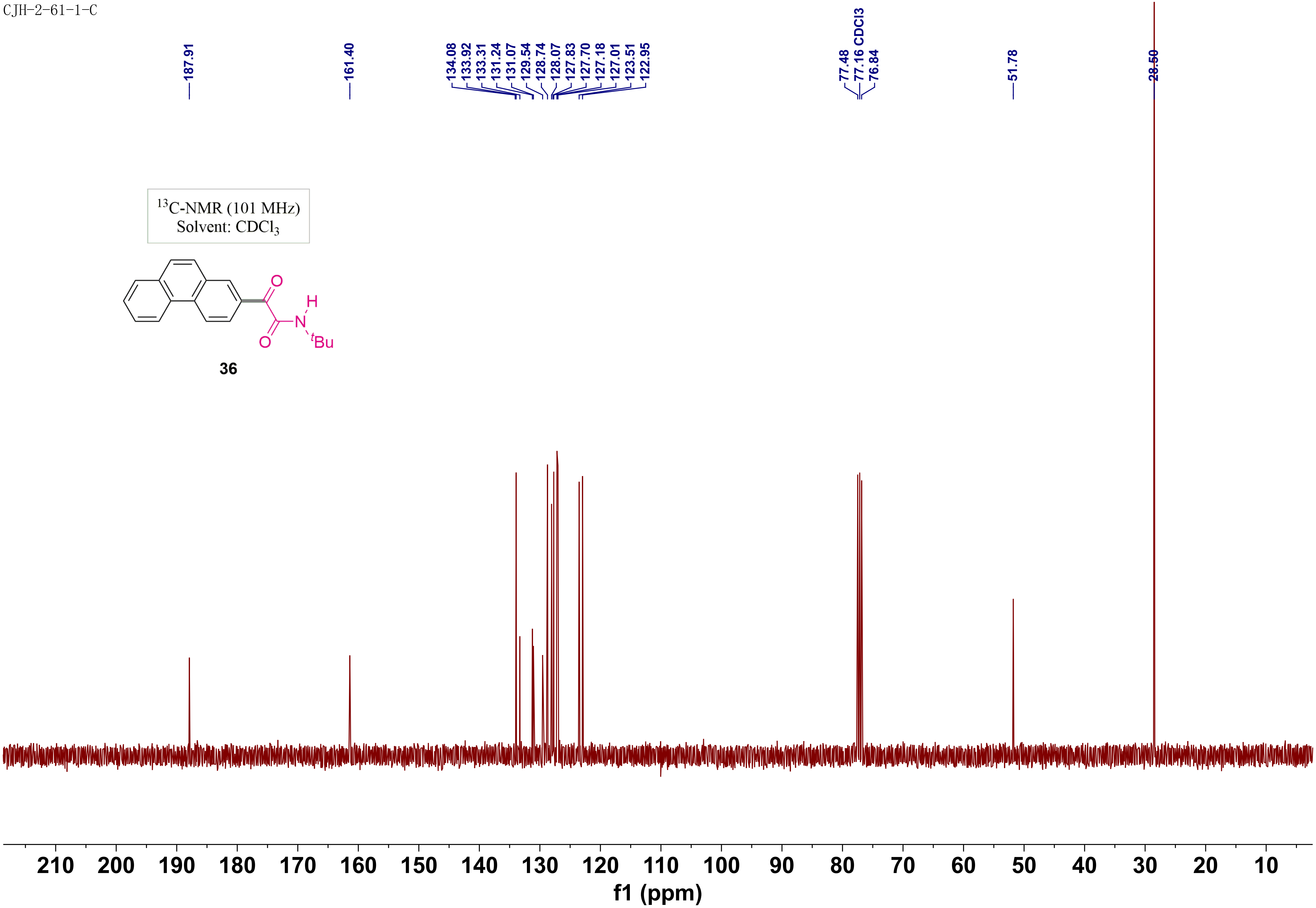

^{13}C-NMR (101 MHz)
Solvent: $CDCl_3$

36

CJH-2-62-2-H

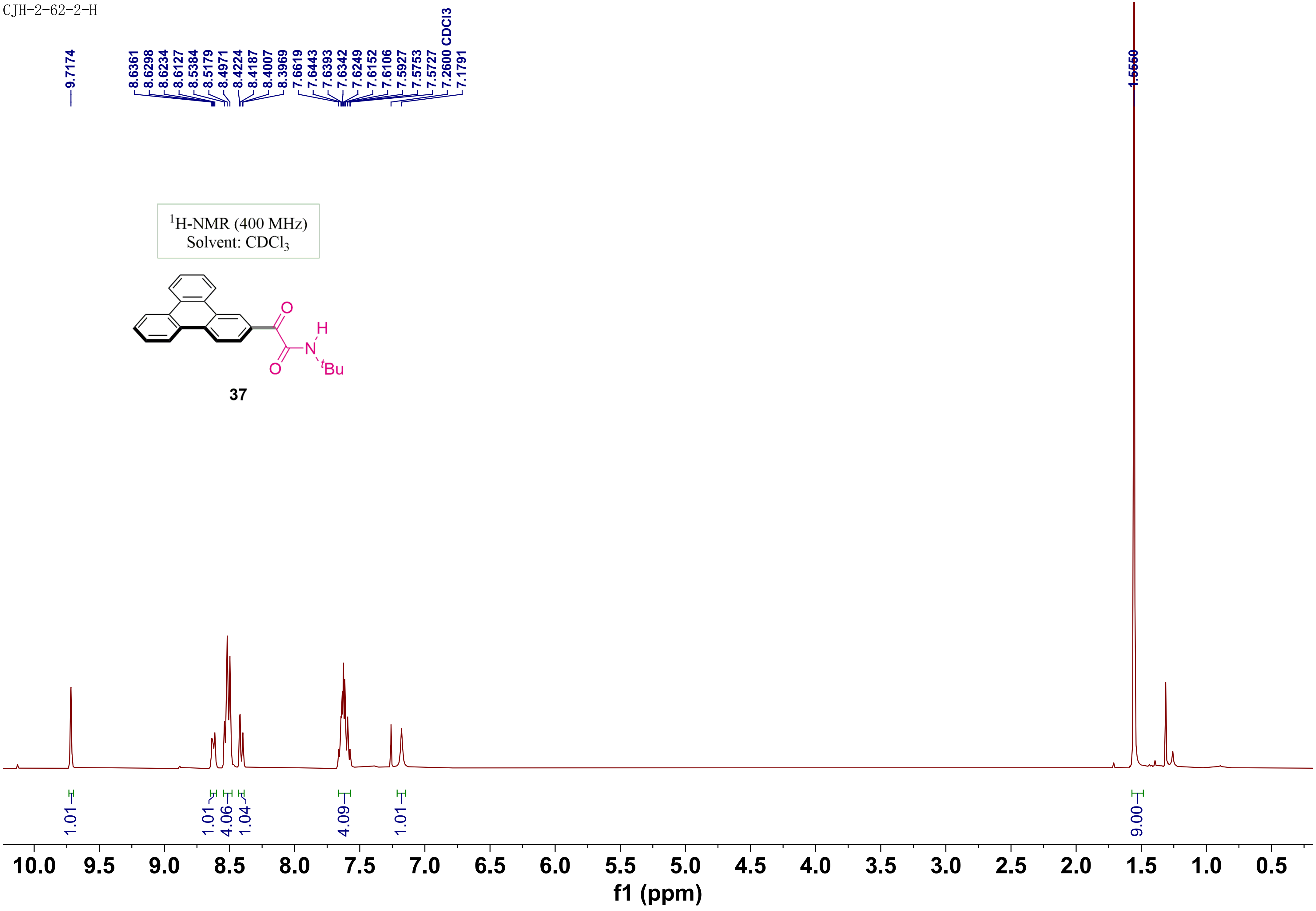
9.7174
8.6361
8.6298
8.6234
8.6127
8.5384
8.5179
8.4971
8.4224
8.4187
8.4007
8.3969
7.6619
7.6443
7.6393
7.6342
7.6249
7.6152
7.6106
7.5927
7.5753
7.5727
7.2600 CDCl3
7.1791
1.5550
^{1}H-NMR (400 MHz)
Solvent: $CDCl_3$
O
H
N
O
tBu
1.01
1.01
4.06
1.04
4.09
1.01
9.00
10.0
9.5
9.0
8.5
8.0
7.5
7.0
6.5
6.0
5.5
5.0
4.5
4.0
3.5
3.0
2.5
2.0
1.5
1.0
0.5
f1 (ppm)

37

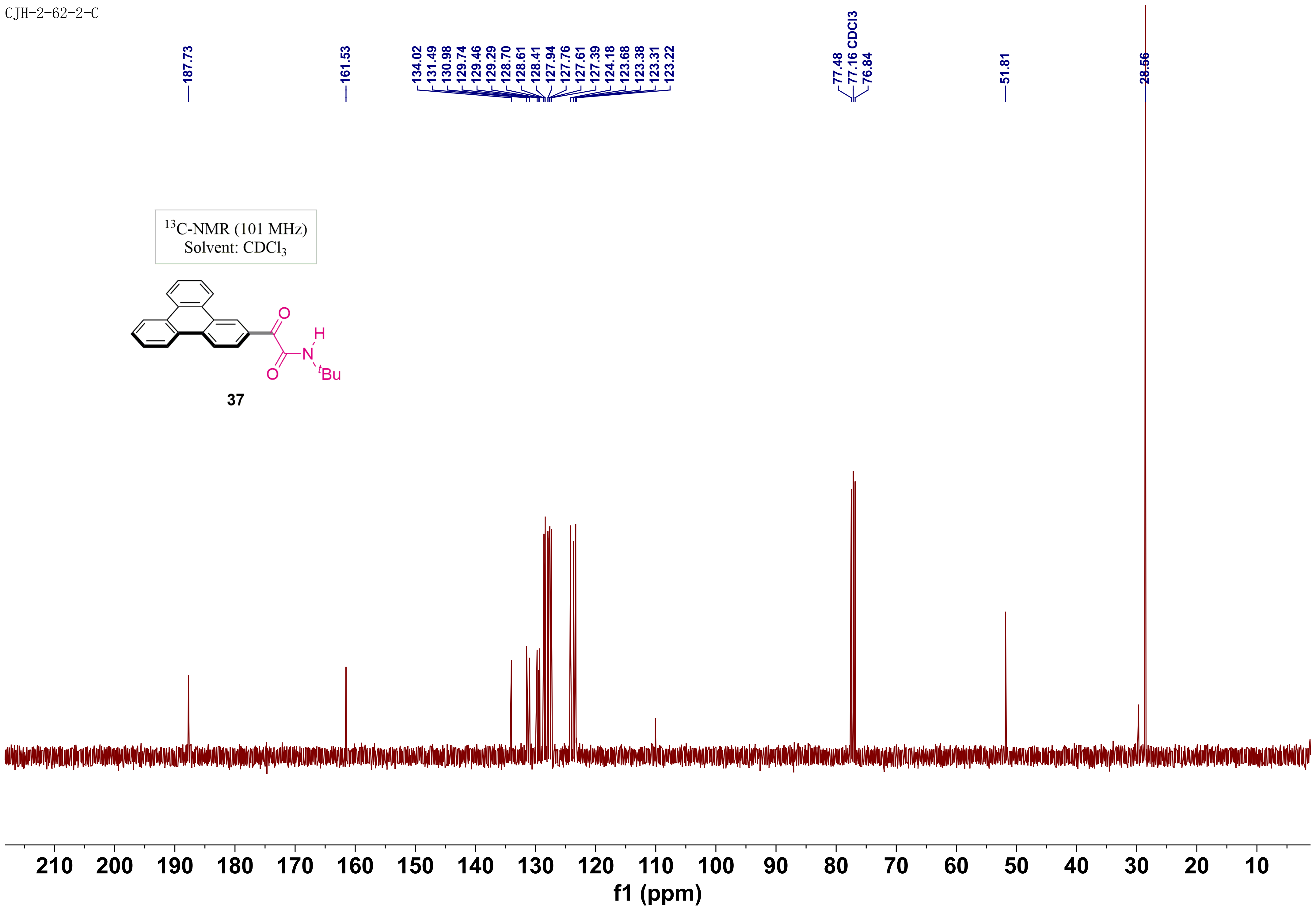
CJH-2-62-2-C
187.73
161.53
134.02
131.49
130.98
129.74
129.46
129.29
128.70
128.61
128.41
127.94
127.76
127.61
127.39
124.18
123.68
123.38
123.31
123.22
77.48
77.16 CDCl3
76.84
51.81
28.56
13C-NMR (101 MHz)
Solvent: CDCl3
O
H
N
O
tBu
37
210 200 190 180 170 160 150 140 130 120 110 100 90 80 70 60 50 40 30 20 10
f1 (ppm)

CJH-2-61-2-H

^{1}H-NMR (400 MHz)
Solvent: $CDCl_3$

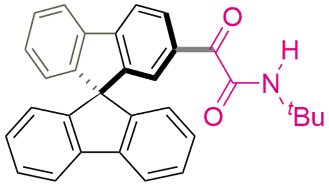

38

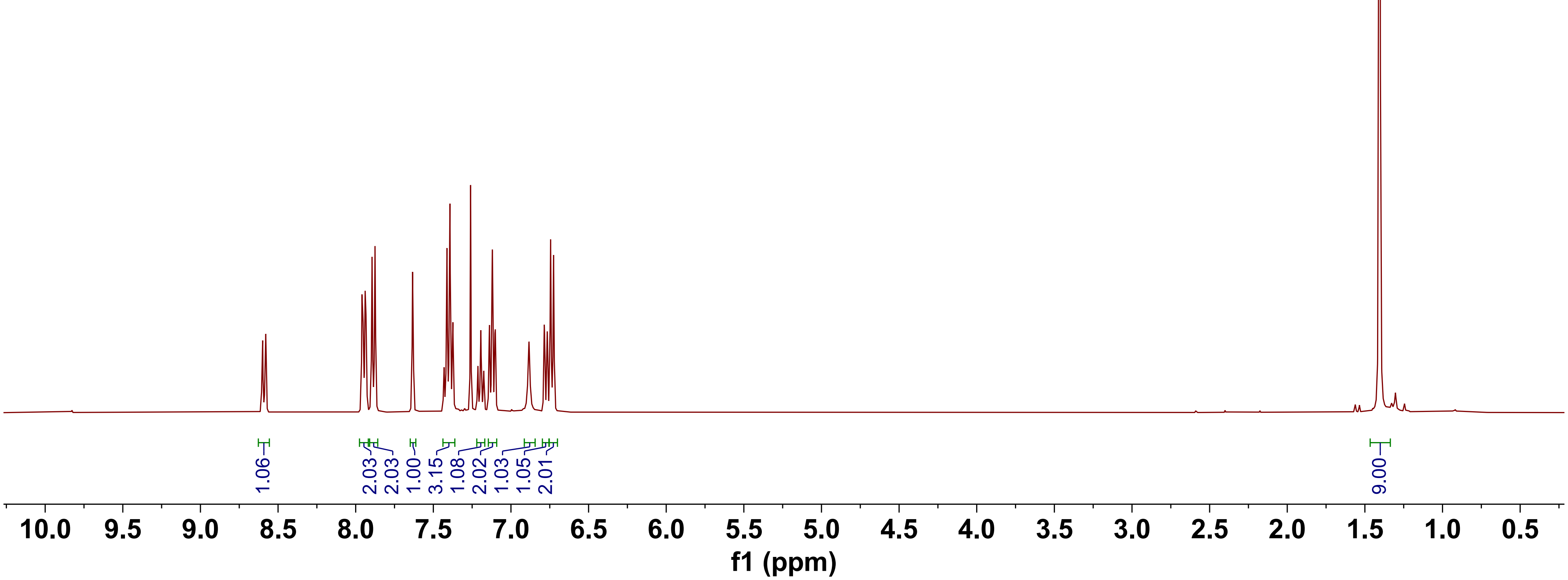

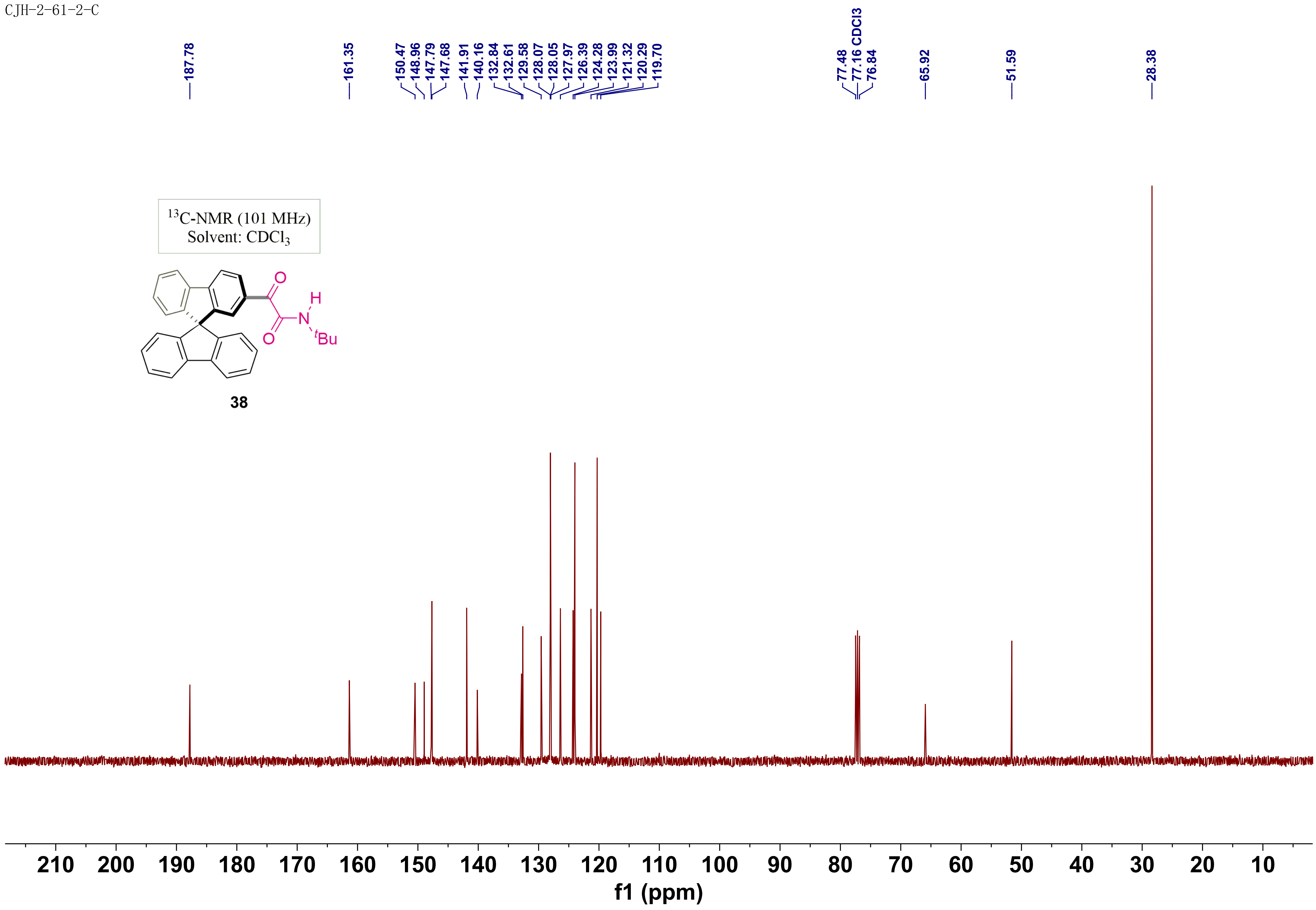
CJH-2-61-2-C
187.78
161.35
150.47
148.96
147.79
147.68
141.91
140.16
132.84
132.61
129.58
128.07
128.05
127.97
126.39
124.28
123.99
121.32
120.29
119.70
77.48
77.16 CDCl3
76.84
65.92
51.59
28.38
13C-NMR (101 MHz)
Solvent: CDCl3
O
H
N
O
tBu
38
210 200 190 180 170 160 150 140 130 120 110 100 90 80 70 60 50 40 30 20 10
f1 (ppm)

CJH-1-123-2-H

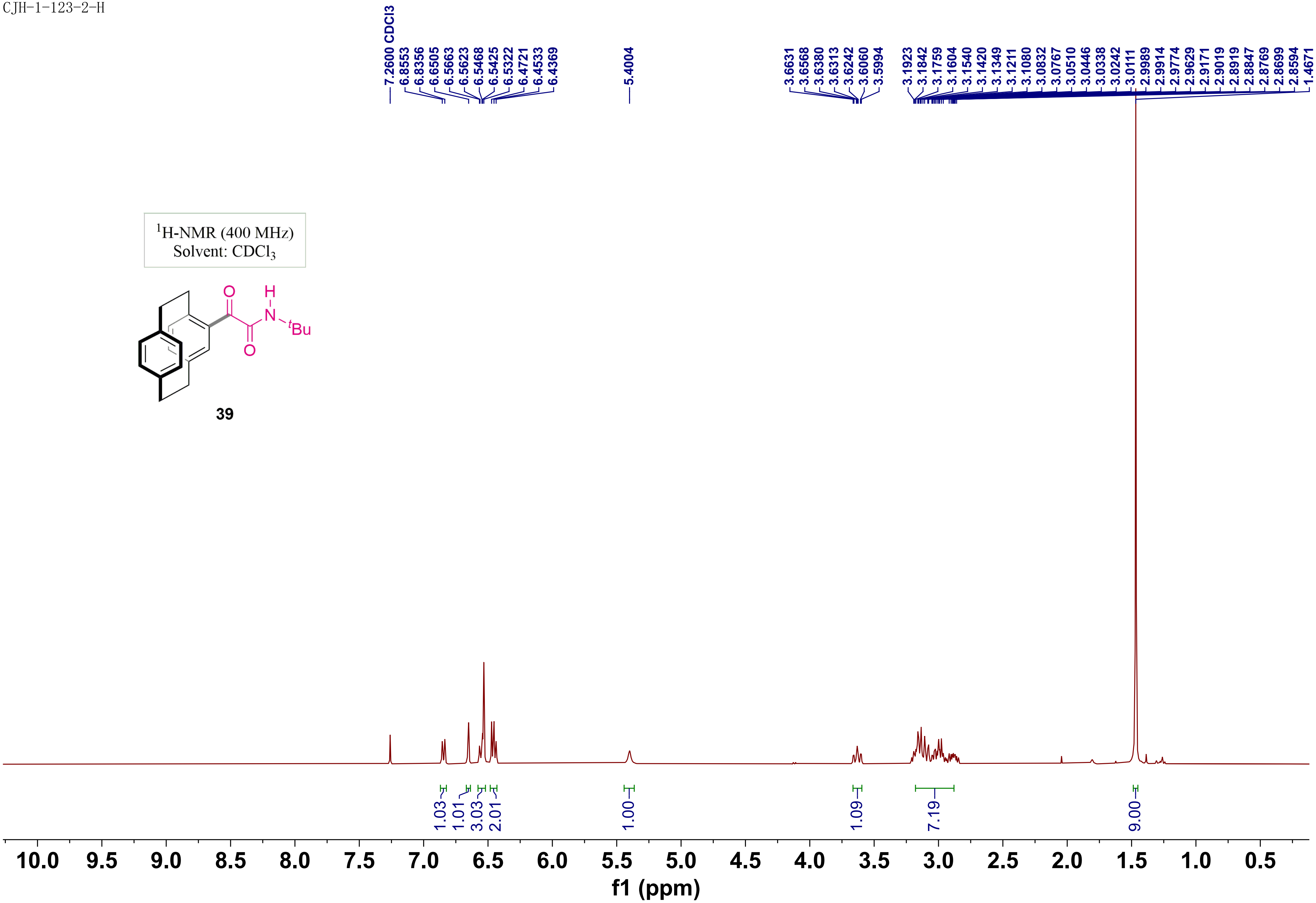

^{1}H-NMR (400 MHz)
Solvent: $CDCl_3$

39

CJH-1-123-2-C

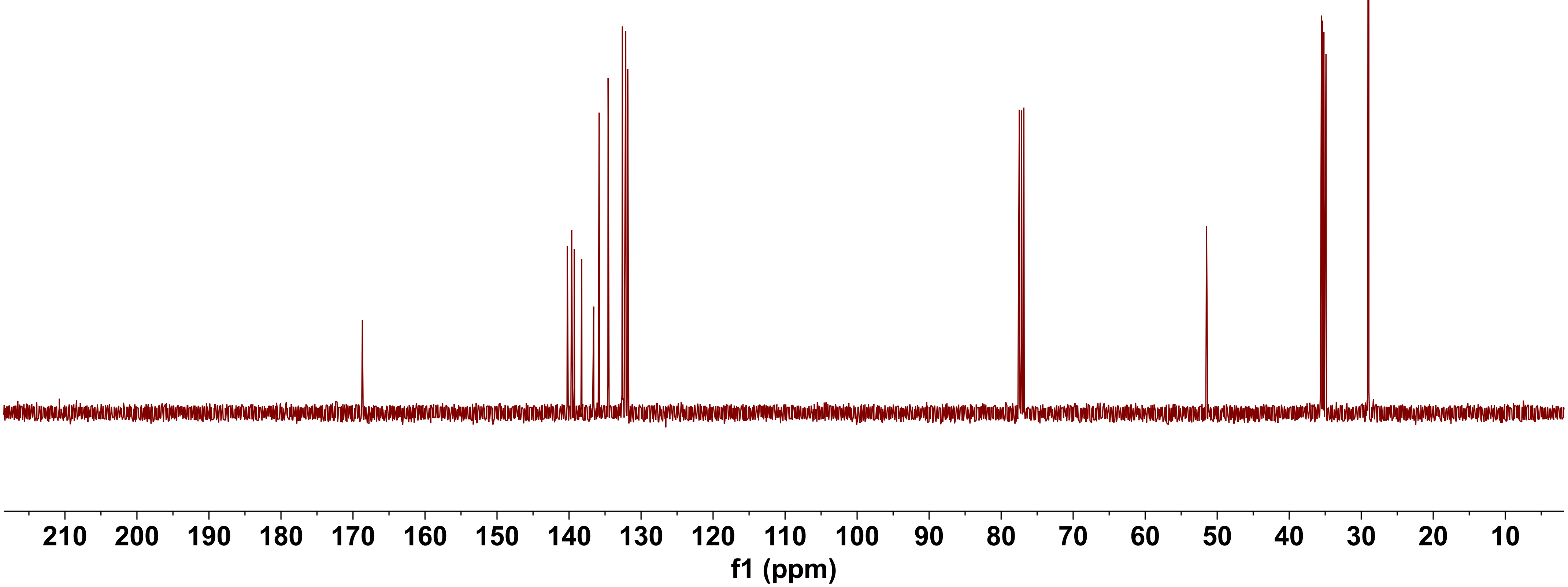

^{13}C-NMR (101 MHz)
Solvent: $CDCl_3$

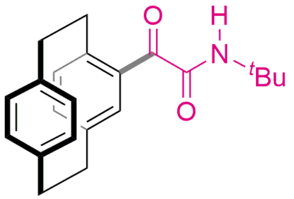

39

CJH-1-102-1-H

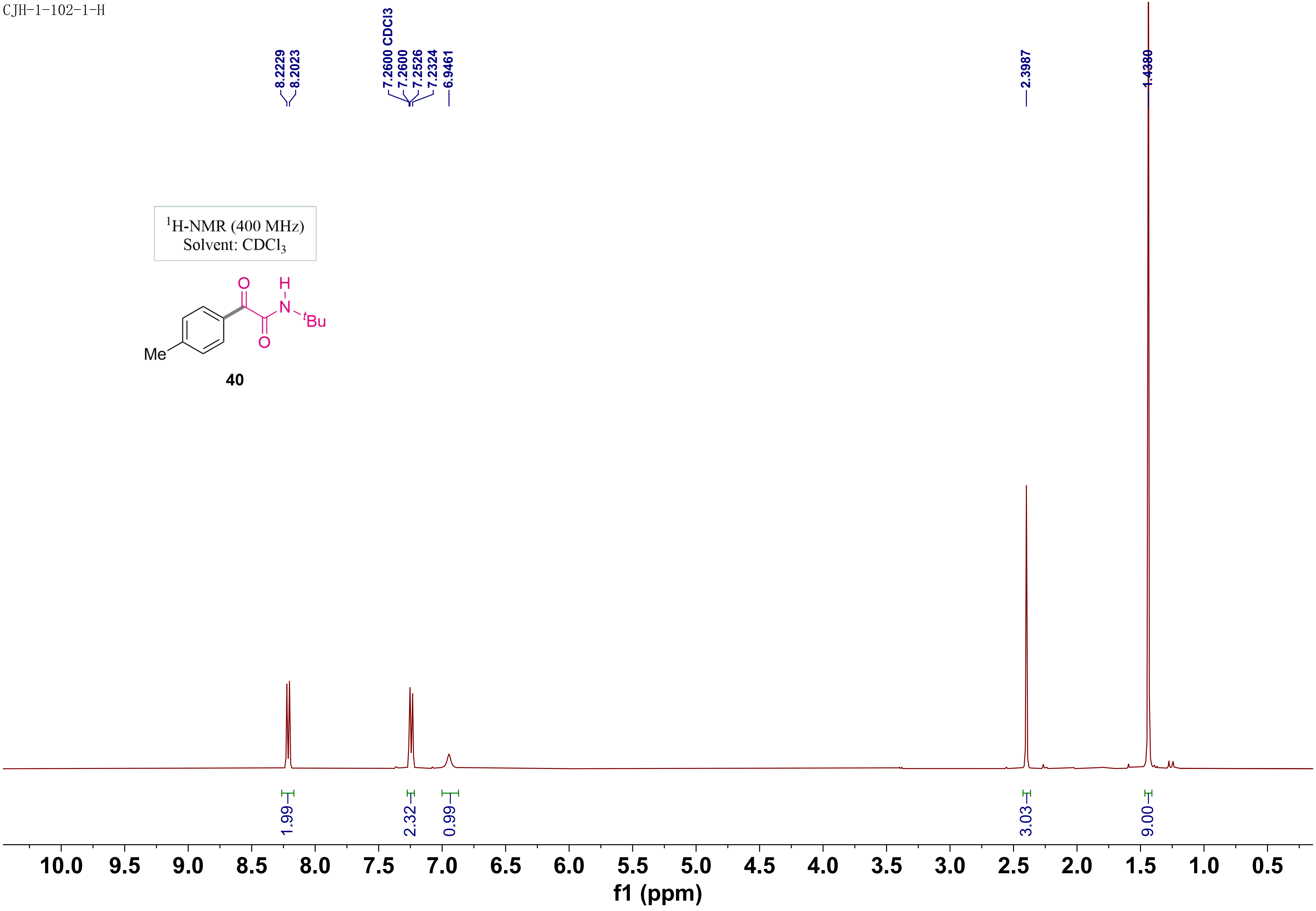
8.2229
8.2023
7.2600 CDCl3
7.2600
7.2526
7.2324
6.9461
2.3987
1.4380
^{1}H-NMR (400 MHz)
Solvent: $CDCl_3$
O
H
N
^{t}Bu
O
Me
40
1.99
2.32
0.99
3.03
9.00
10.0
9.5
9.0
8.5
8.0
7.5
7.0
6.5
6.0
5.5
5.0
4.5
4.0
3.5
3.0
2.5
2.0
1.5
1.0
0.5
f1 (ppm)

CJH-1-102-1-C

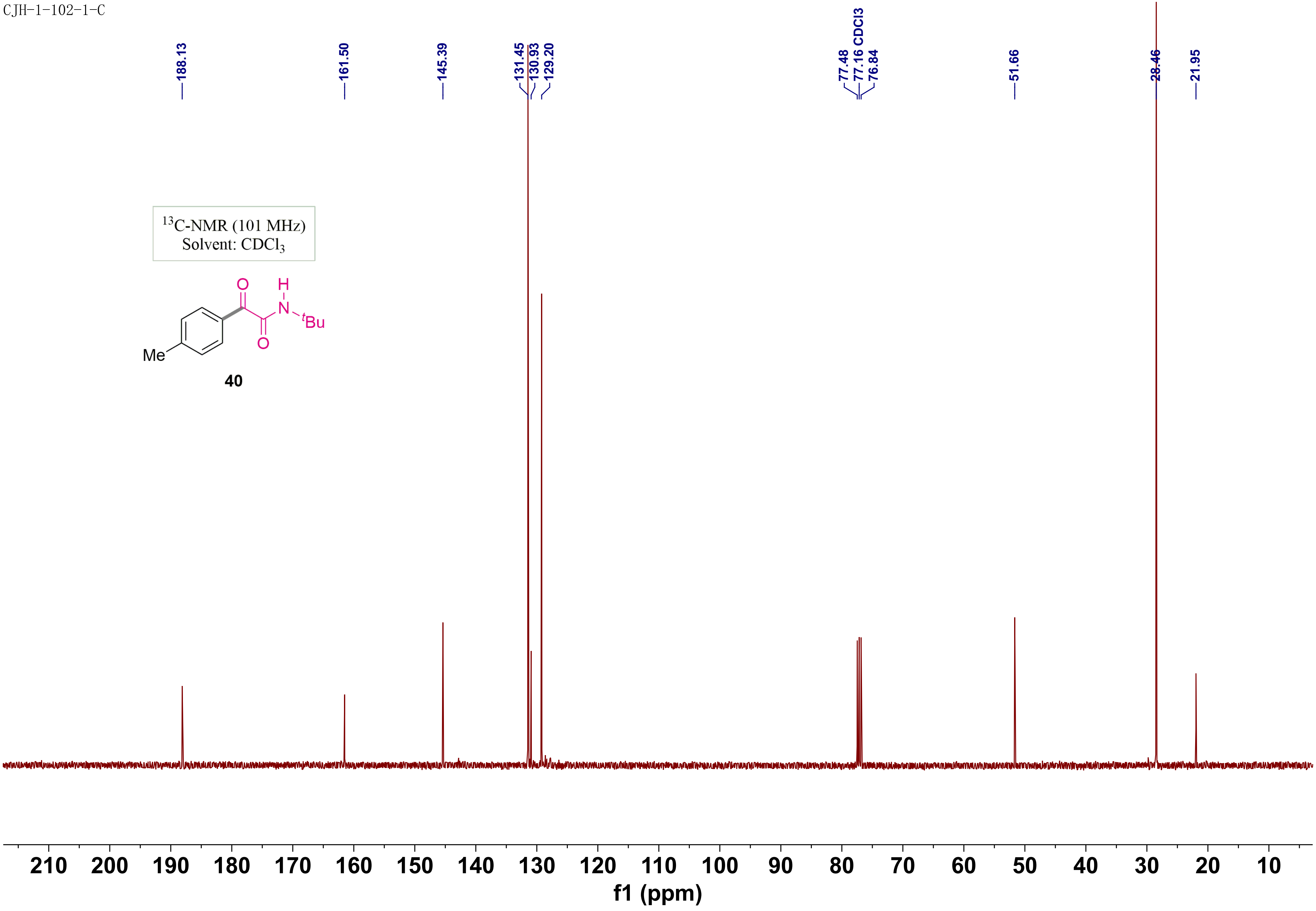
188.13
161.50
145.39
131.45
130.93
129.20
77.48
77.16 CDCl3
76.84
51.66
28.46
21.95
^{13}C-NMR (101 MHz)
Solvent: $CDCl_3$
O
H
N
tBu
O
Me
40
210 200 190 180 170 160 150 140 130 120 110 100 90 80 70 60 50 40 30 20 10
f1 (ppm)

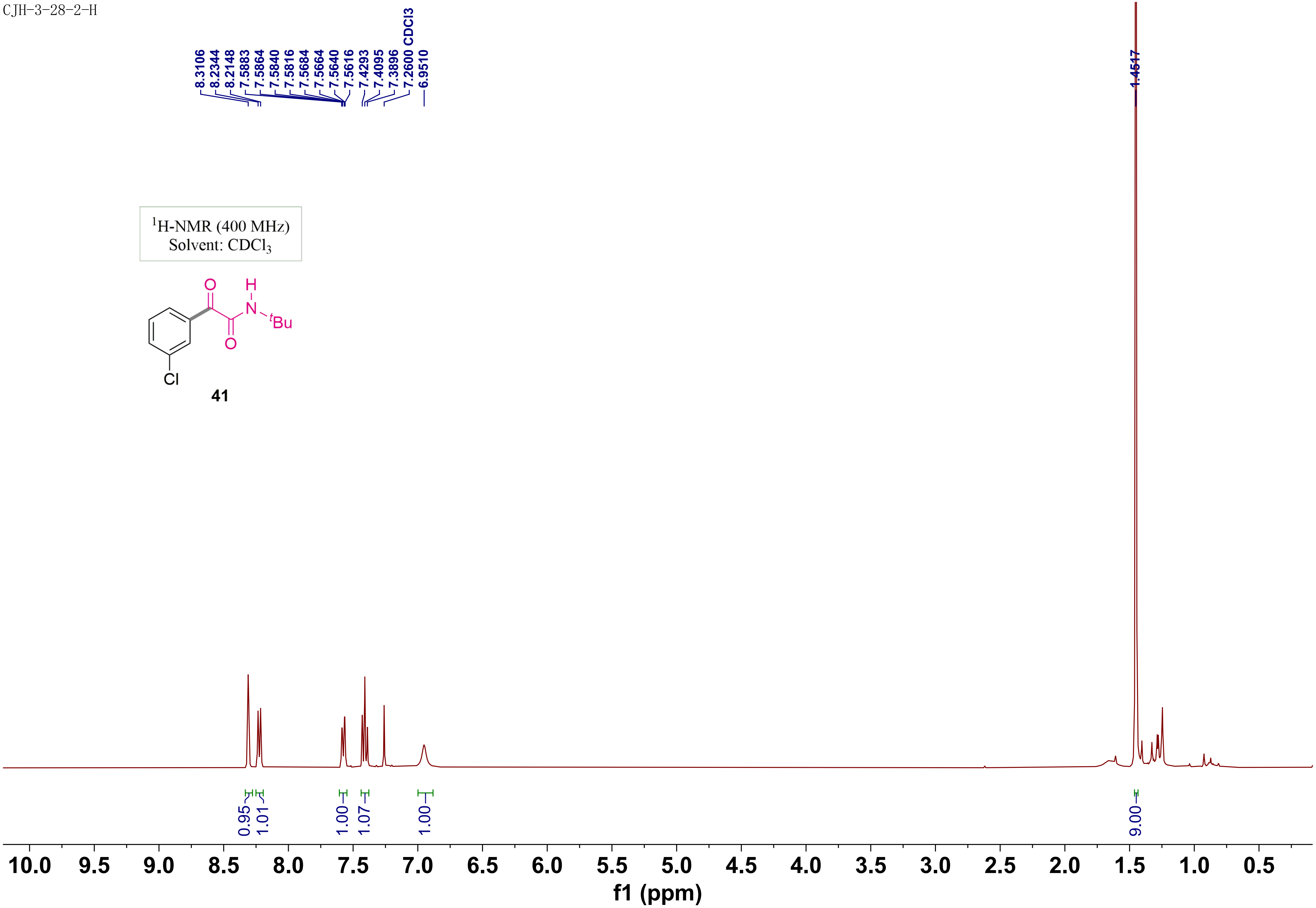
CJH-3-28-2-H
8.3106
8.2344
8.2148
7.5883
7.5864
7.5840
7.5816
7.5684
7.5664
7.5640
7.5616
7.4293
7.4095
7.3896
7.2600 CDCl3
6.9510
1.4517
^{1}H-NMR (400 MHz)
Solvent: $CDCl_3$
O
H
N
^{t}Bu
O
Cl
41
0.95
1.01
1.00
1.07
1.00
9.00
10.0
9.5
9.0
8.5
8.0
7.5
7.0
6.5
6.0
5.5
5.0
4.5
4.0
3.5
3.0
2.5
2.0
1.5
1.0
0.5
f1 (ppm)

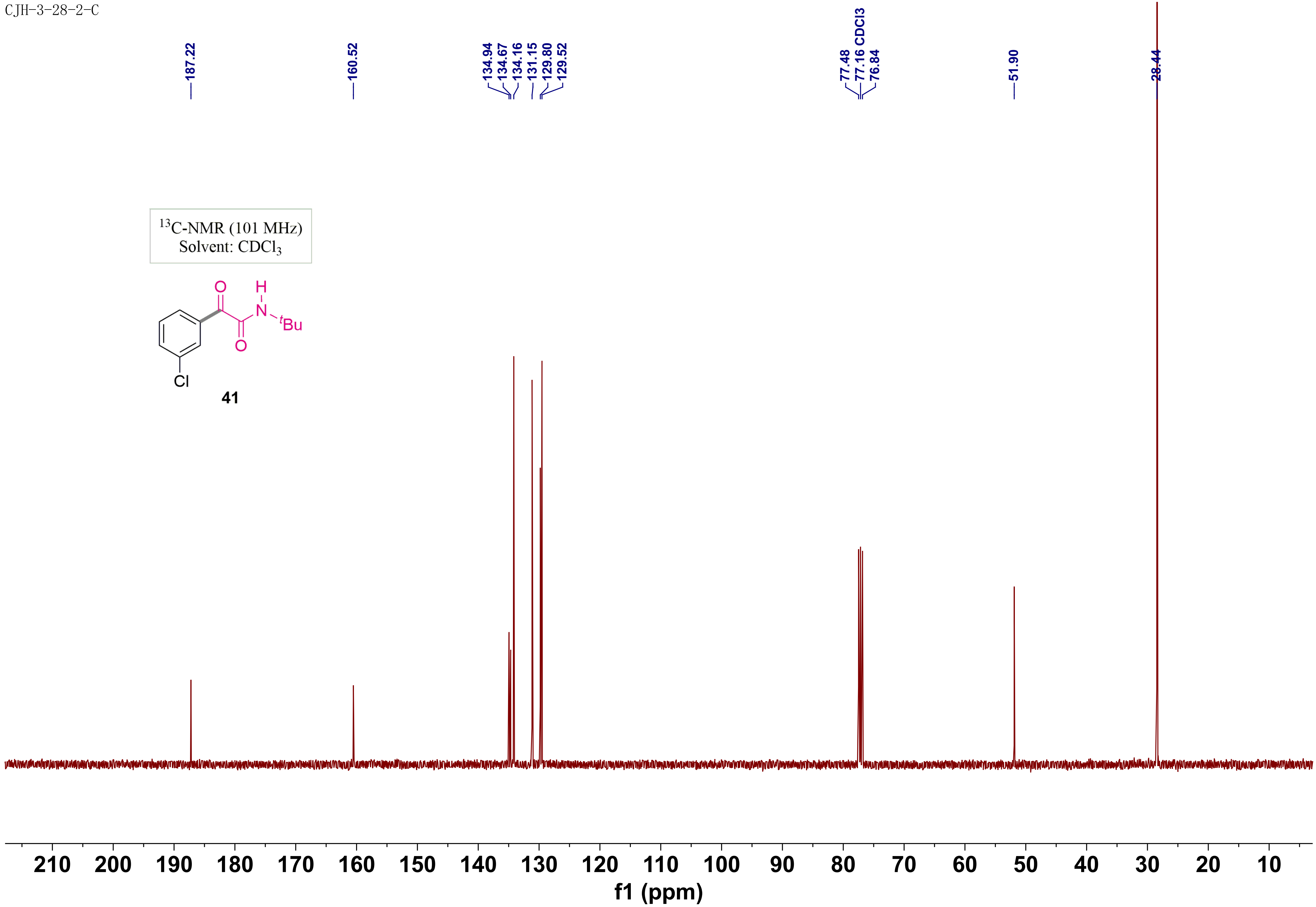
CJH-3-28-2-C
187.22
160.52
134.94
134.67
134.16
131.15
129.80
129.52
77.48
77.16 CDCl3
76.84
51.90
28.44
13C-NMR (101 MHz)
Solvent: CDCl3
O
H
N
tBu
O
Cl
41
210 200 190 180 170 160 150 140 130 120 110 100 90 80 70 60 50 40 30 20 10
f1 (ppm)

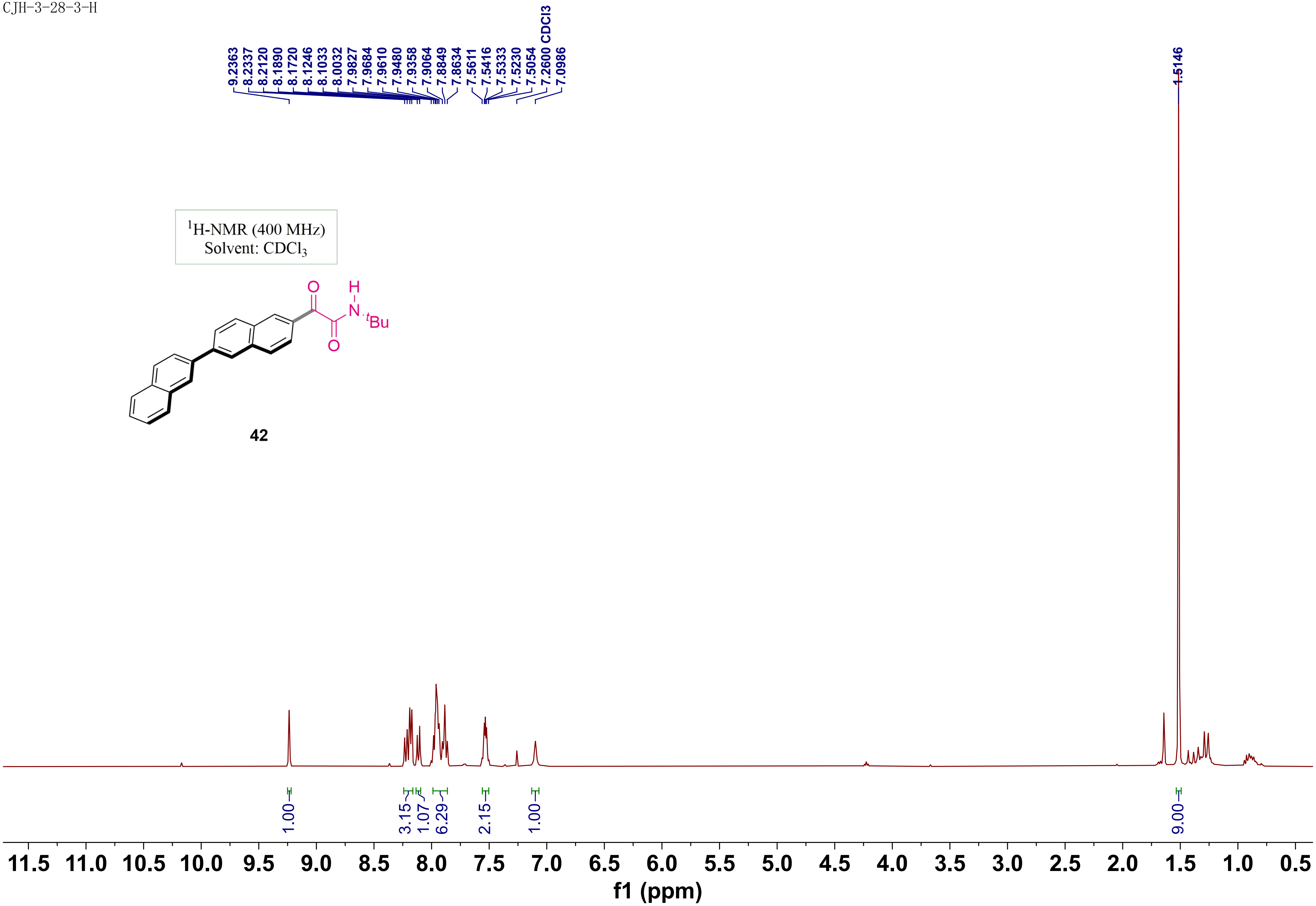
CJH-3-28-3-H
9.2363
8.2337
8.2120
8.1890
8.1720
8.1246
8.1033
8.0032
7.9827
7.9684
7.9610
7.9480
7.9358
7.9064
7.8849
7.8634
7.5611
7.5416
7.5333
7.5230
7.5054
7.2600 CDCl3
7.0986
1.5146
^{1}H-NMR (400 MHz)
Solvent: $CDCl_3$
O
H
N
^{t}Bu
O
42
1.00
3.15
1.07
6.29
2.15
1.00
9.00
11.5 11.0 10.5 10.0 9.5 9.0 8.5 8.0 7.5 7.0 6.5 6.0 5.5 5.0 4.5 4.0 3.5 3.0 2.5 2.0 1.5 1.0 0.5
f1 (ppm)

CJH-3-28-3-C

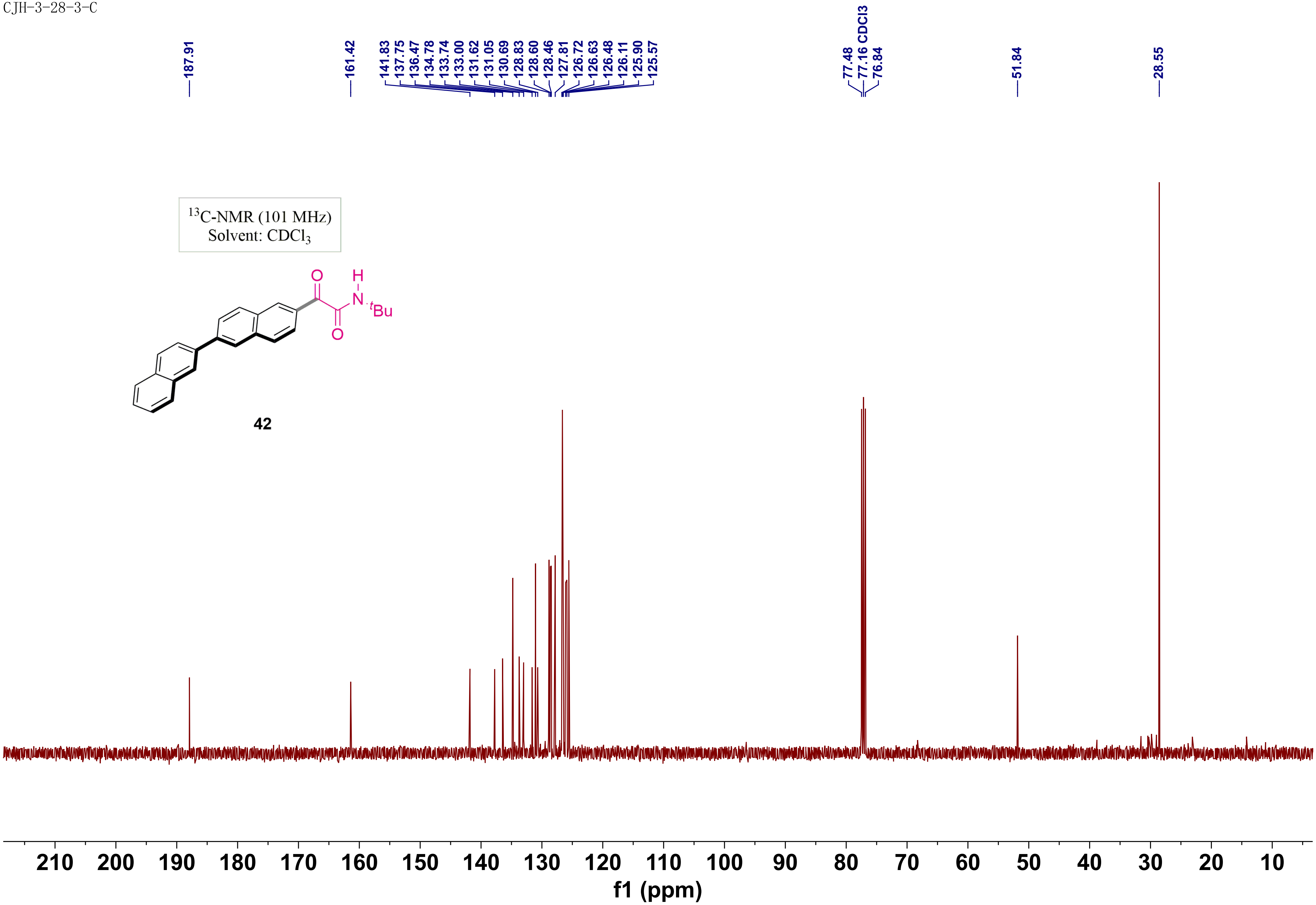
187.91
161.42
141.83
137.75
136.47
134.78
133.74
133.00
131.62
131.05
130.69
128.83
128.60
128.46
127.81
126.72
126.63
126.48
126.11
125.90
125.57
77.48
77.16 CDCl3
76.84
51.84
28.55
13C-NMR (101 MHz)
Solvent: CDCl3
O
H
N
tBu
O
42
210
200
190
180
170
160
150
140
130
120
110
100
90
80
70
60
50
40
30
20
10
f1 (ppm)

CJH-1-186-1-H

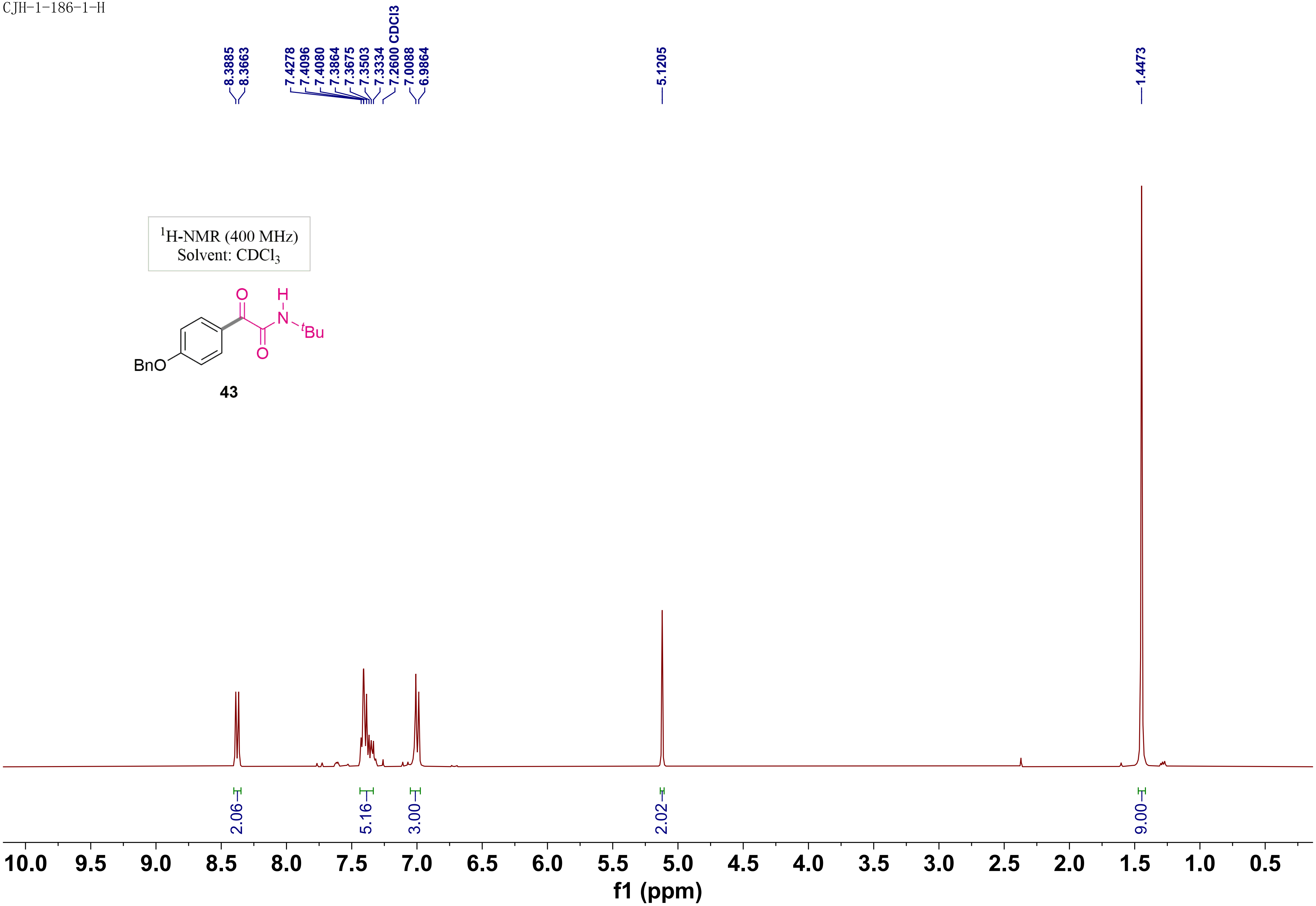

8.3885
8.3663
7.4278
7.4096
7.4080
7.3864
7.3675
7.3503
7.3334
7.2600 CDCl3
7.0088
6.9864
5.1205
1.4473
^{1}H-NMR (400 MHz)
Solvent: $CDCl_3$
BnO
43
2.06
5.16
3.00
2.02
9.00
10.0 9.5 9.0 8.5 8.0 7.5 7.0 6.5 6.0 5.5 5.0 4.5 4.0 3.5 3.0 2.5 2.0 1.5 1.0 0.5
f1 (ppm)

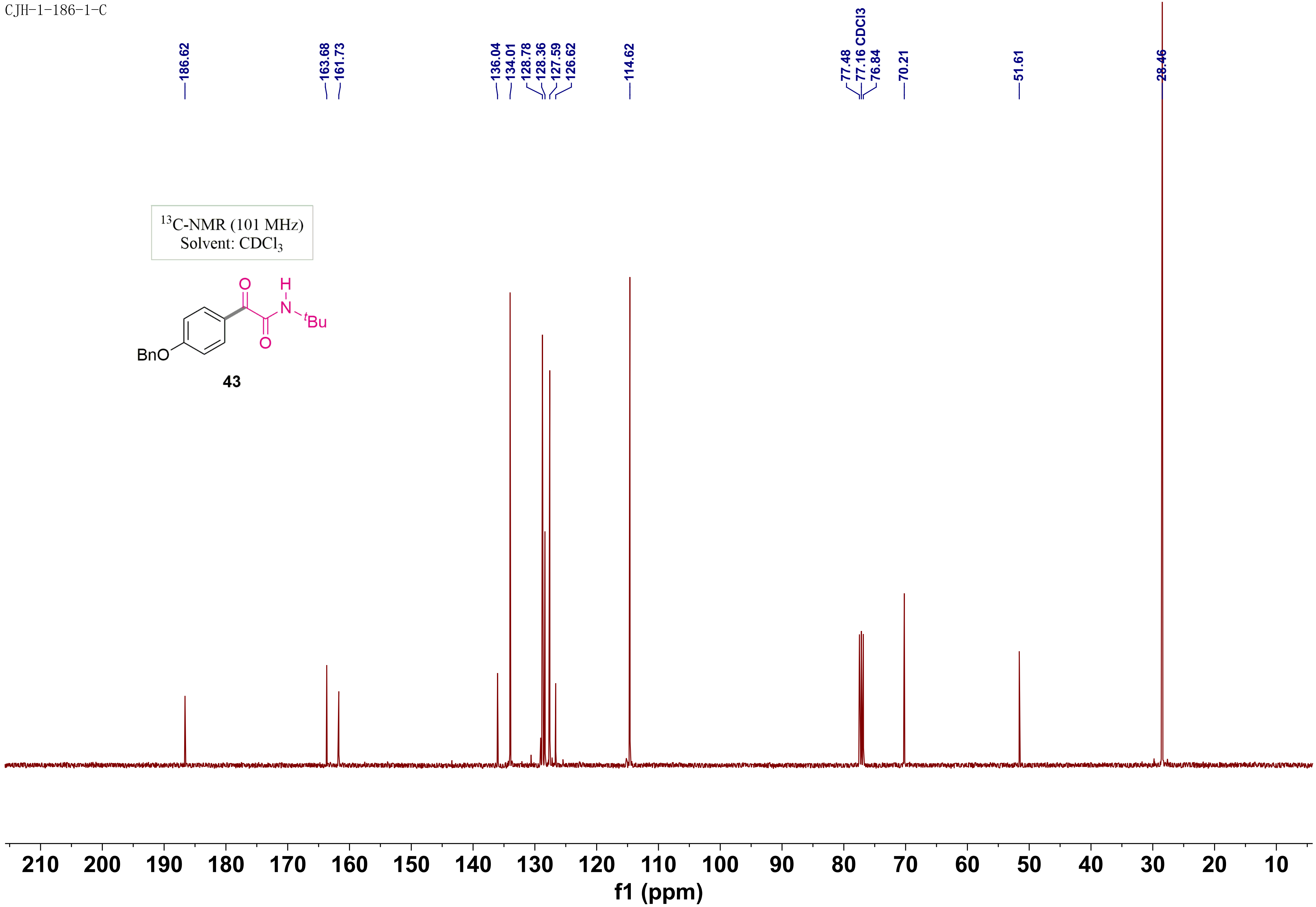
CJH-1-186-1-C
186.62
163.68
161.73
136.04
134.01
128.78
128.36
127.59
126.62
114.62
77.48
77.16 CDCl3
76.84
70.21
51.61
28.46
13C-NMR (101 MHz)
Solvent: CDCl3
O
H
N
tBu
O
BnO
43
210
200
190
180
170
160
150
140
130
120
110
100
90
80
70
60
50
40
30
20
10
f1 (ppm)

CJH-1-109-3-H

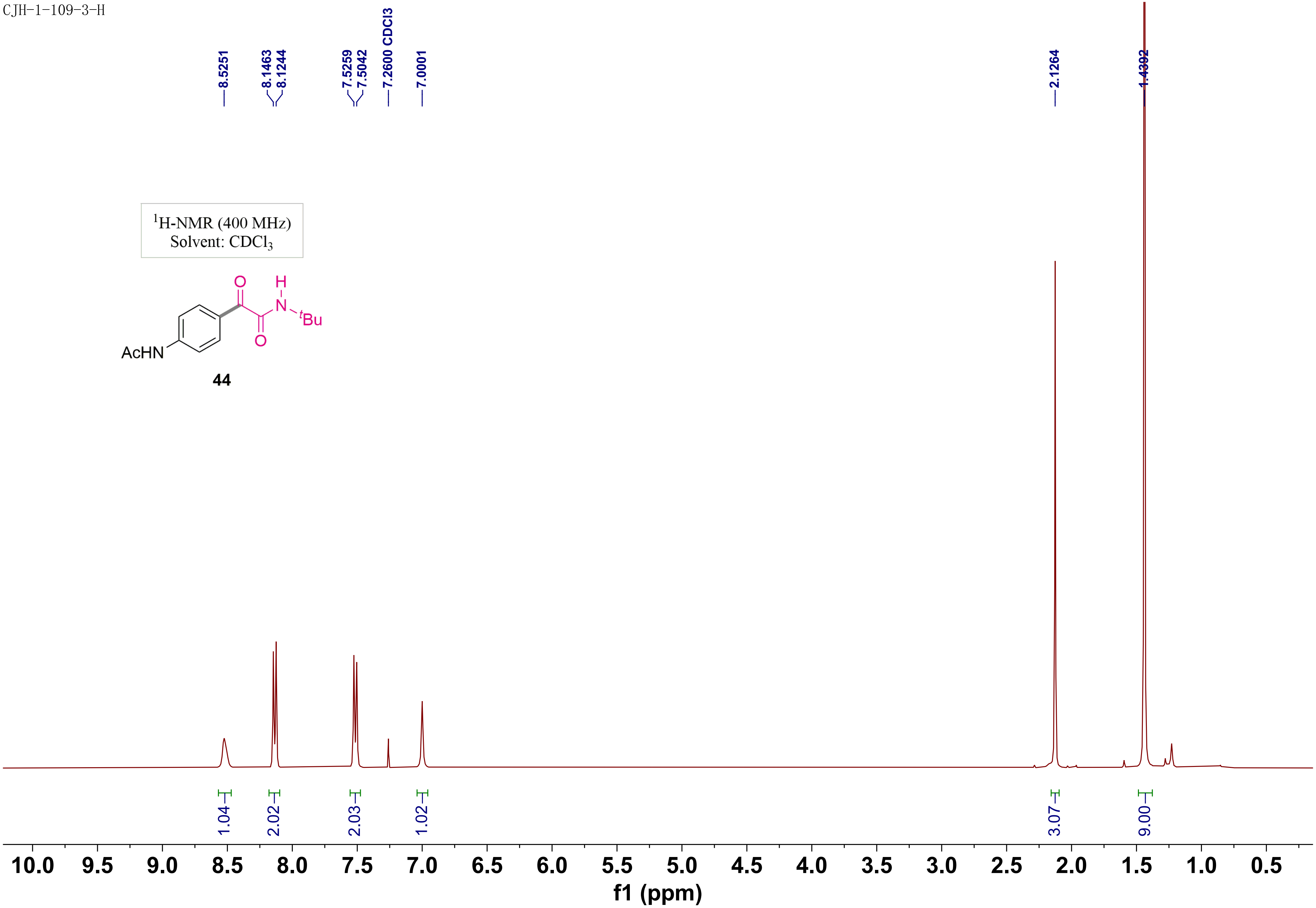
8.5251
8.1463
8.1244
7.5259
7.5042
7.2600 CDCl3
7.0001
2.1264
1.4392
^{1}H-NMR (400 MHz)
Solvent: $CDCl_3$
O
H
N
tBu
O
AcHN
44
1.04
2.02
2.03
1.02
3.07
9.00
10.0
9.5
9.0
8.5
8.0
7.5
7.0
6.5
6.0
5.5
5.0
4.5
4.0
3.5
3.0
2.5
2.0
1.5
1.0
0.5
f1 (ppm)

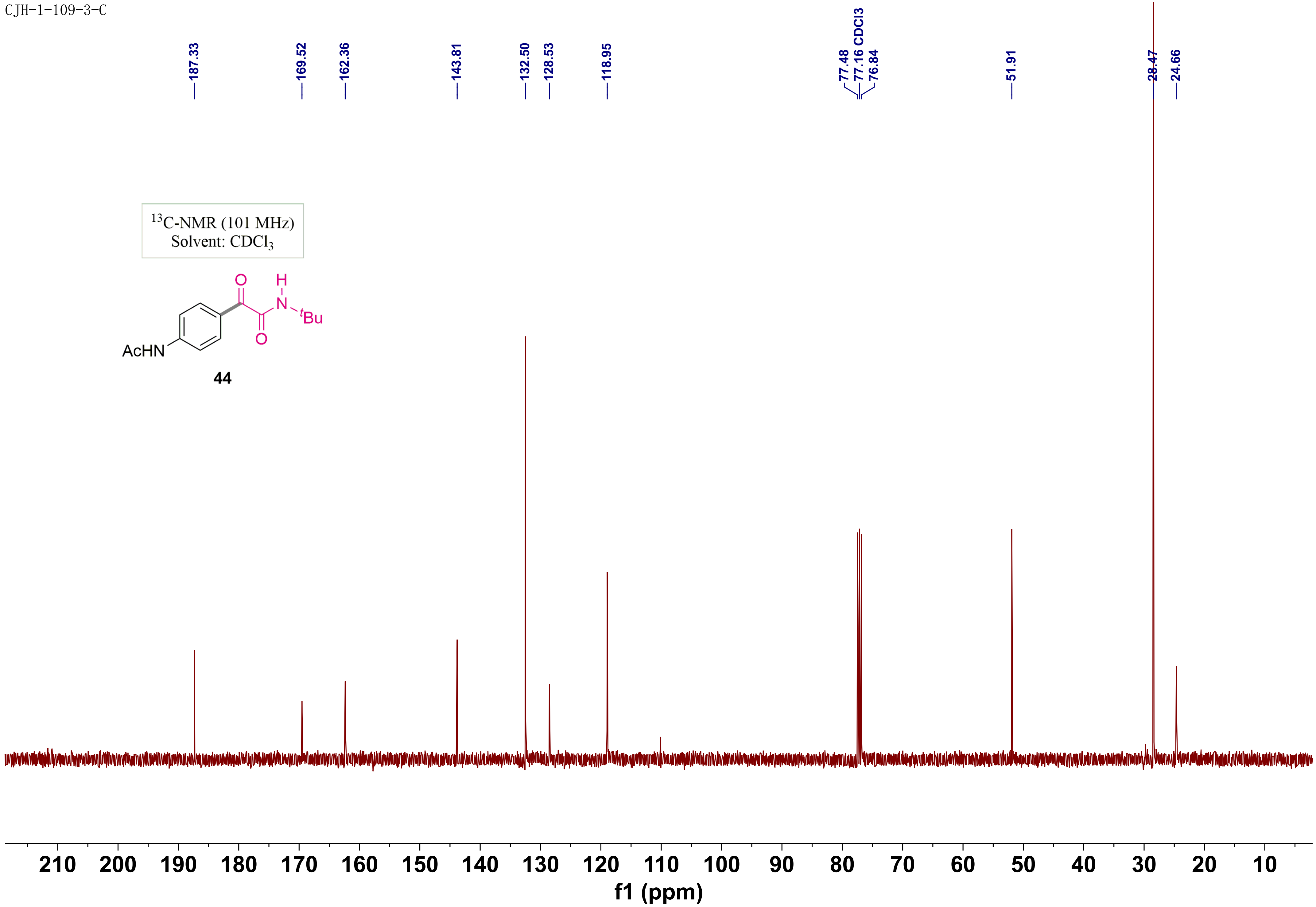
CJH-1-109-3-C
187.33
169.52
162.36
143.81
132.50
128.53
118.95
77.48
77.16 CDCl3
76.84
51.91
28.47
24.66
^{13}C-NMR (101 MHz)
Solvent: $CDCl_3$
O
H
N
^{t}Bu
O
AcHN
44
210
200
190
180
170
160
150
140
130
120
110
100
90
80
70
60
50
40
30
20
10
f1 (ppm)

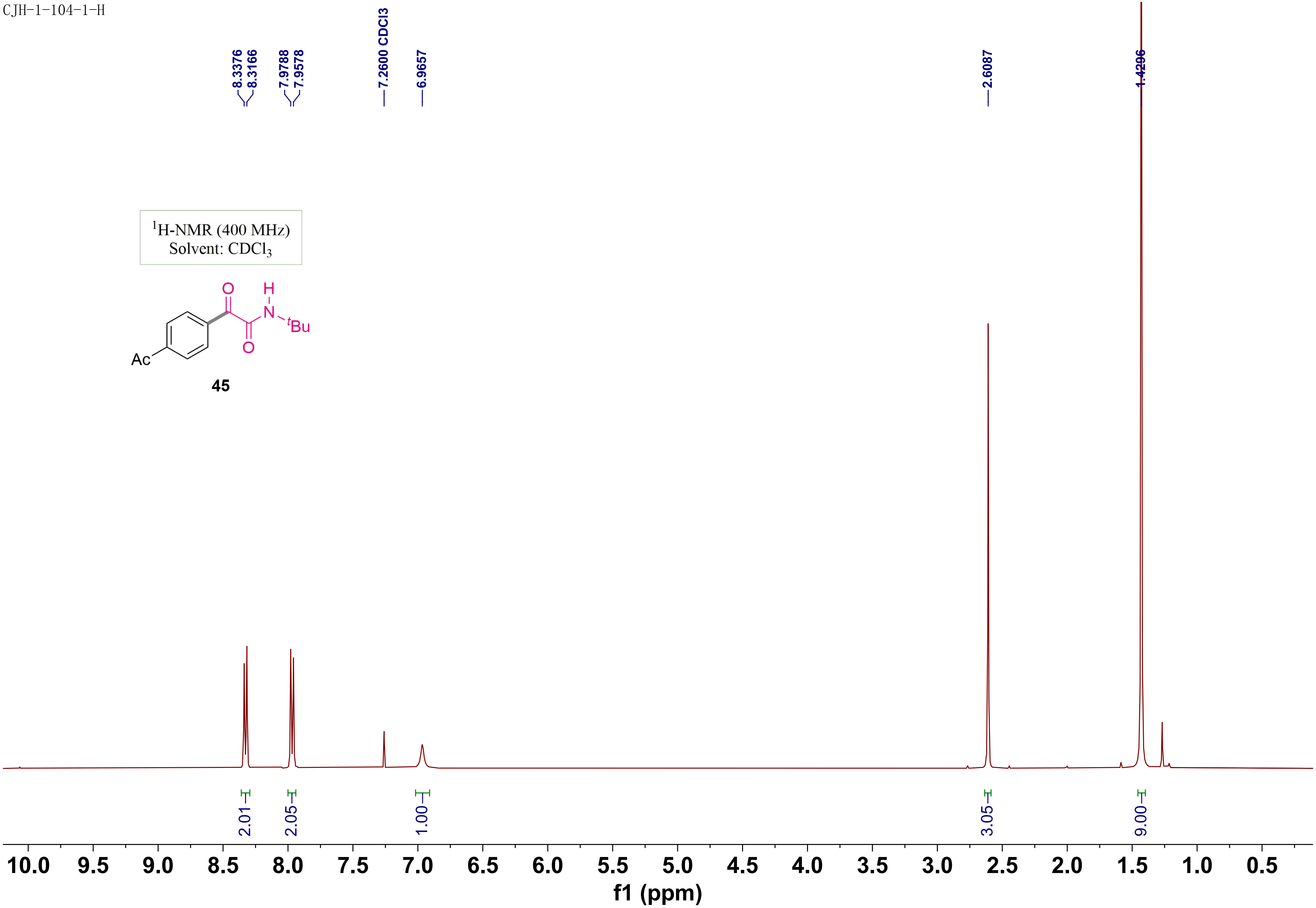
CJH-1-104-1-H
8.3376
8.3166
7.9788
7.9578
7.2600 CDCl3
6.9657
2.6087
1.4296
^{1}H-NMR (400 MHz)
Solvent: $CDCl_3$
O
H
N
^{t}Bu
O
Ac
45
2.01
2.05
1.00
3.05
9.00
10.0 9.5 9.0 8.5 8.0 7.5 7.0 6.5 6.0 5.5 5.0 4.5 4.0 3.5 3.0 2.5 2.0 1.5 1.0 0.5
f1 (ppm)

CJH-1-104-1-C

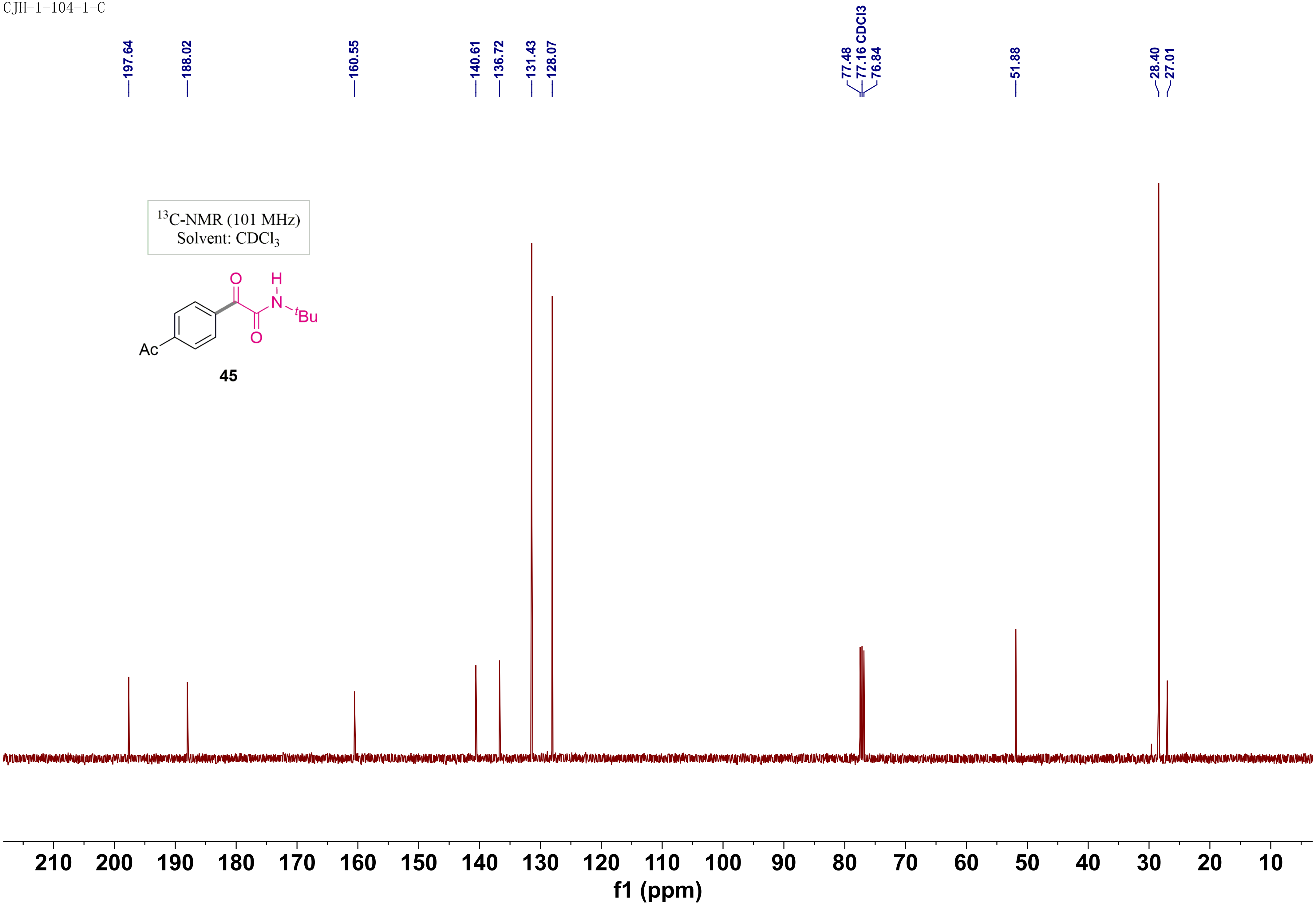

197.64
188.02
160.55
140.61
136.72
131.43
128.07
77.48
77.16 CDCl3
76.84
51.88
28.40
27.01
^{13}C-NMR (101 MHz)
Solvent: $CDCl_3$
O
H
N
tBu
O
Ac
45
210 200 190 180 170 160 150 140 130 120 110 100 90 80 70 60 50 40 30 20 10
f1 (ppm)

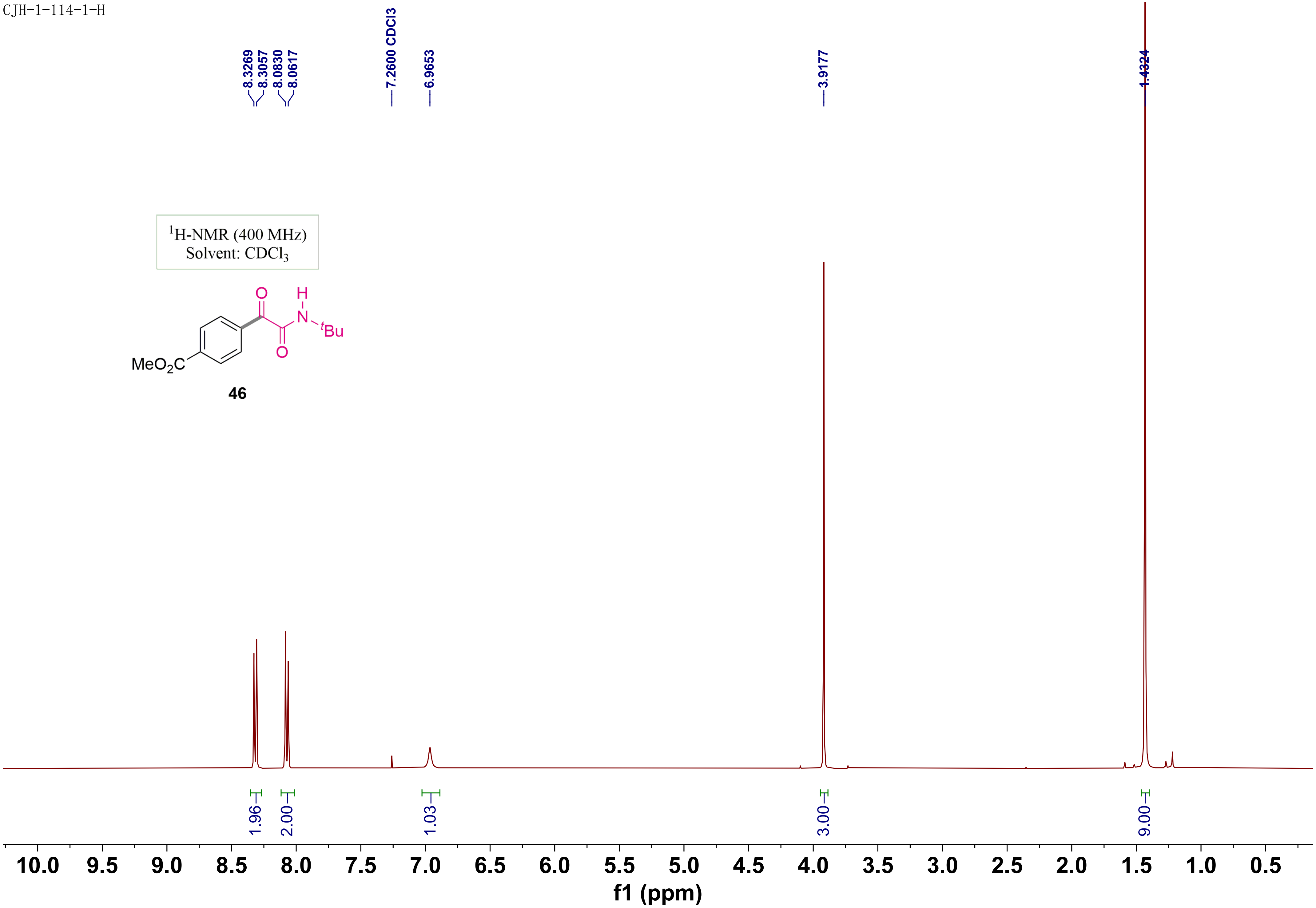
CJH-1-114-1-H
8.3269
8.3057
8.0830
8.0617
7.2600 CDCl3
6.9653
3.9177
1.4324
^{1}H-NMR (400 MHz)
Solvent: $CDCl_3$
O
H
N
^{t}Bu
O
MeO_2C
46
1.96
2.00
1.03
3.00
9.00
10.0 9.5 9.0 8.5 8.0 7.5 7.0 6.5 6.0 5.5 5.0 4.5 4.0 3.5 3.0 2.5 2.0 1.5 1.0 0.5
f1 (ppm)

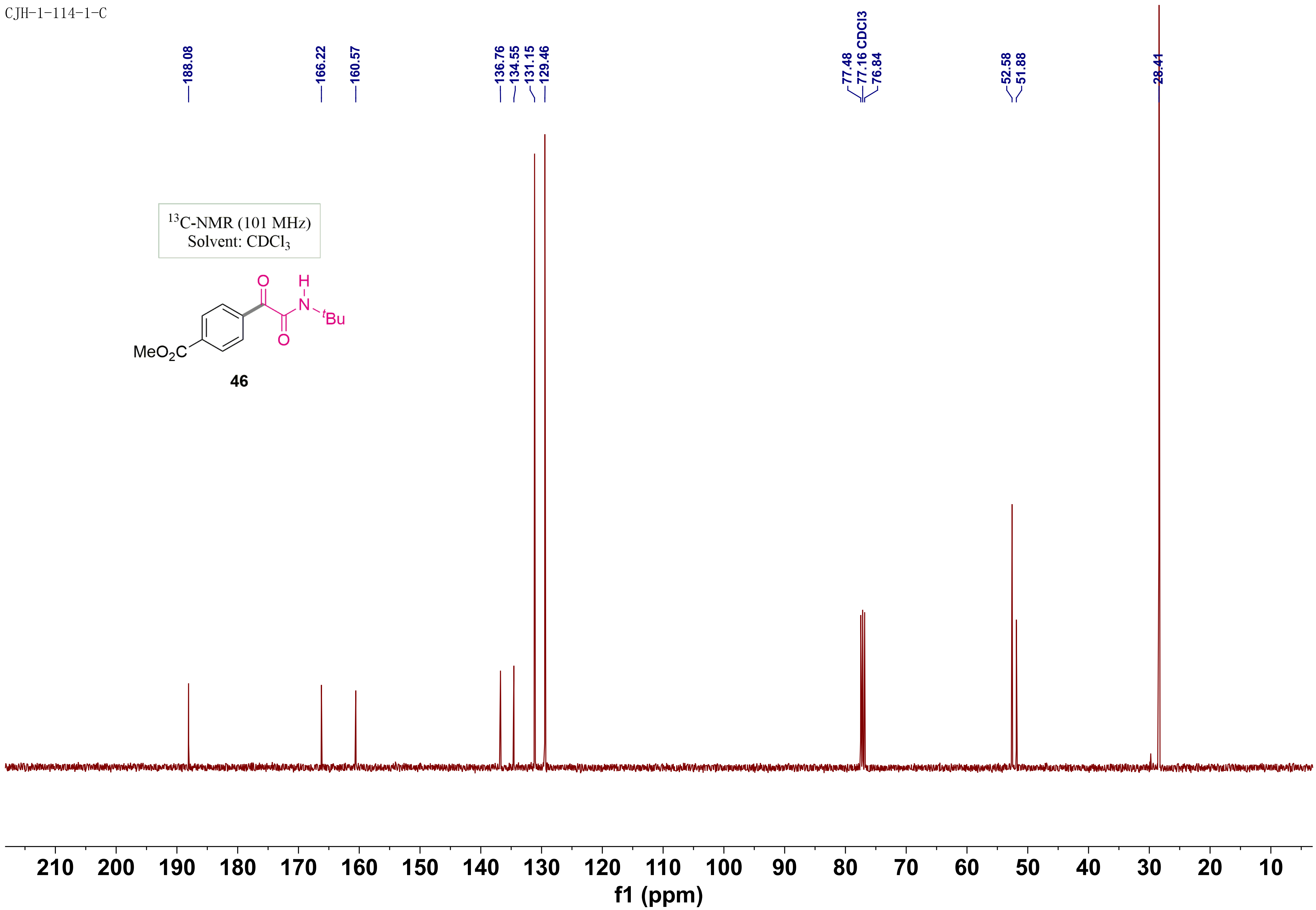
CJH-1-114-1-C
188.08
166.22
160.57
136.76
134.55
131.15
129.46
77.48
77.16 CDCl3
76.84
52.58
51.88
28.41
^{13}C-NMR (101 MHz)
Solvent: $CDCl_3$
MeO$_2$C
O
H
N
^{t}Bu
O
46
210 200 190 180 170 160 150 140 130 120 110 100 90 80 70 60 50 40 30 20 10
f1 (ppm)

CJH-1-107-2-H

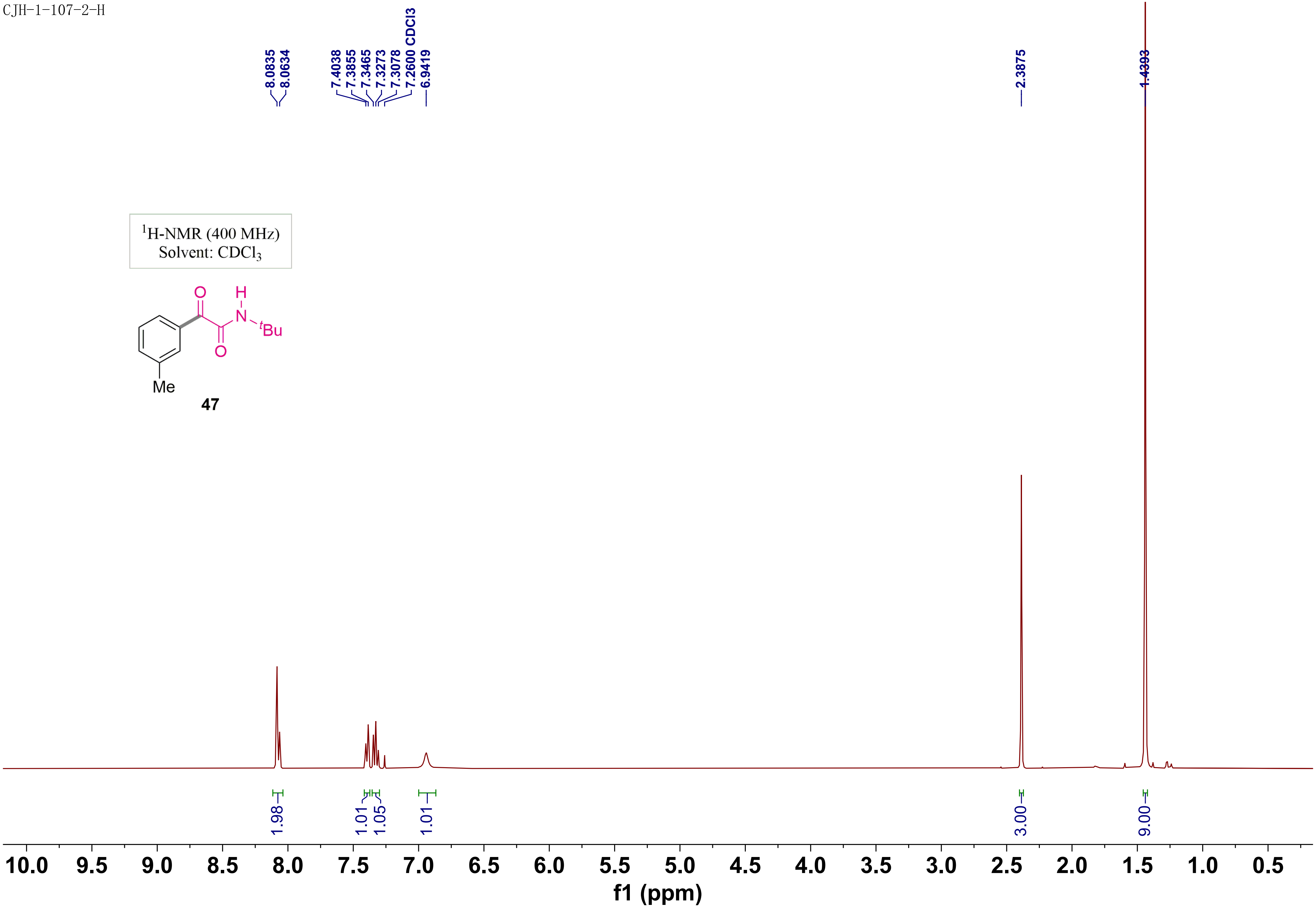

47

CJH-1-107-2-C

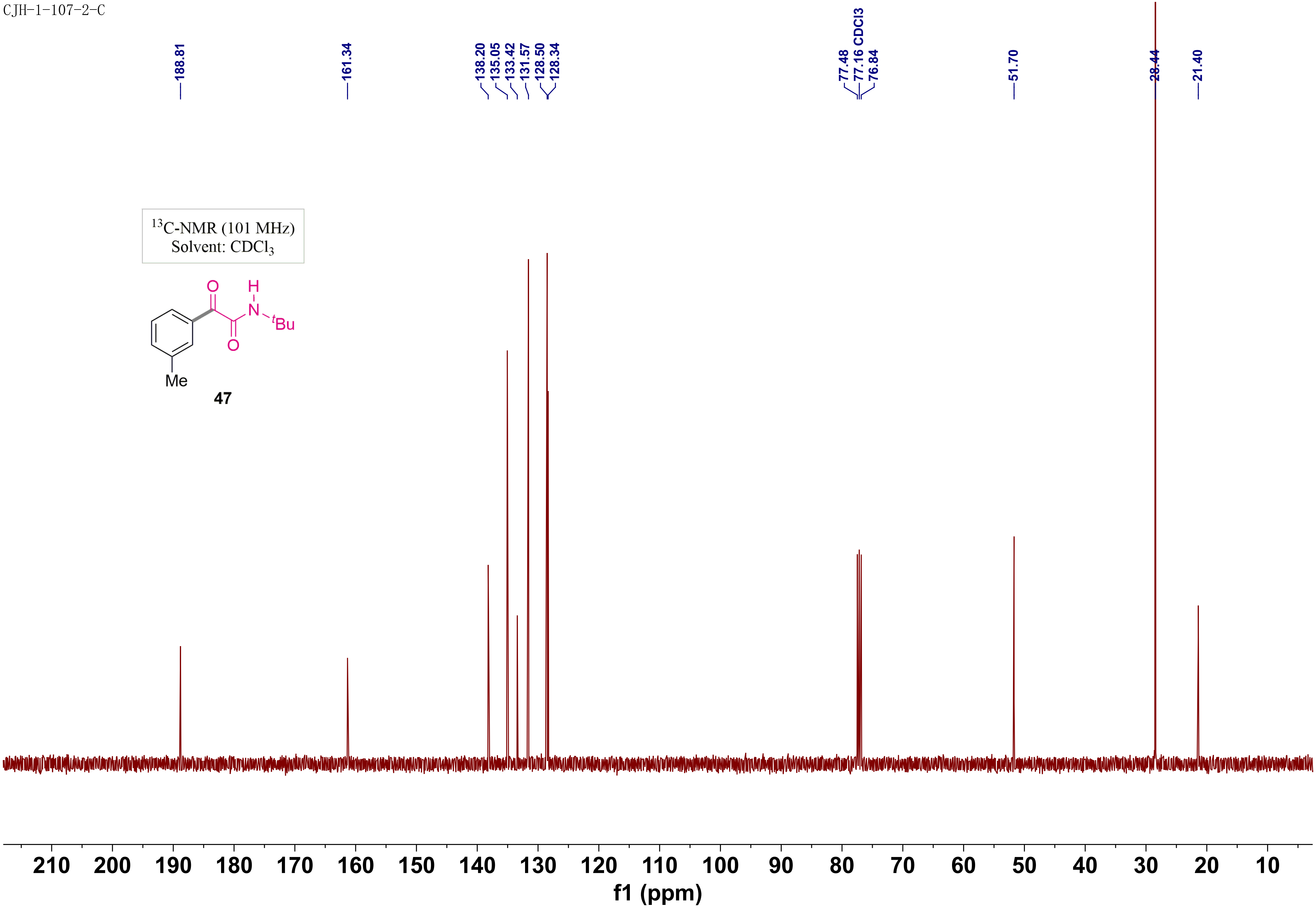

188.81
161.34
138.20
135.05
133.42
131.57
128.50
128.34
77.48
77.16 CDCl3
76.84
51.70
28.44
21.40
^{13}C-NMR (101 MHz)
Solvent: $CDCl_3$
O
H
N
tBu
O
Me
47
210
200
190
180
170
160
150
140
130
120
110
100
90
80
70
60
50
40
30
20
10
f1 (ppm)

CJH-1-107-3-H

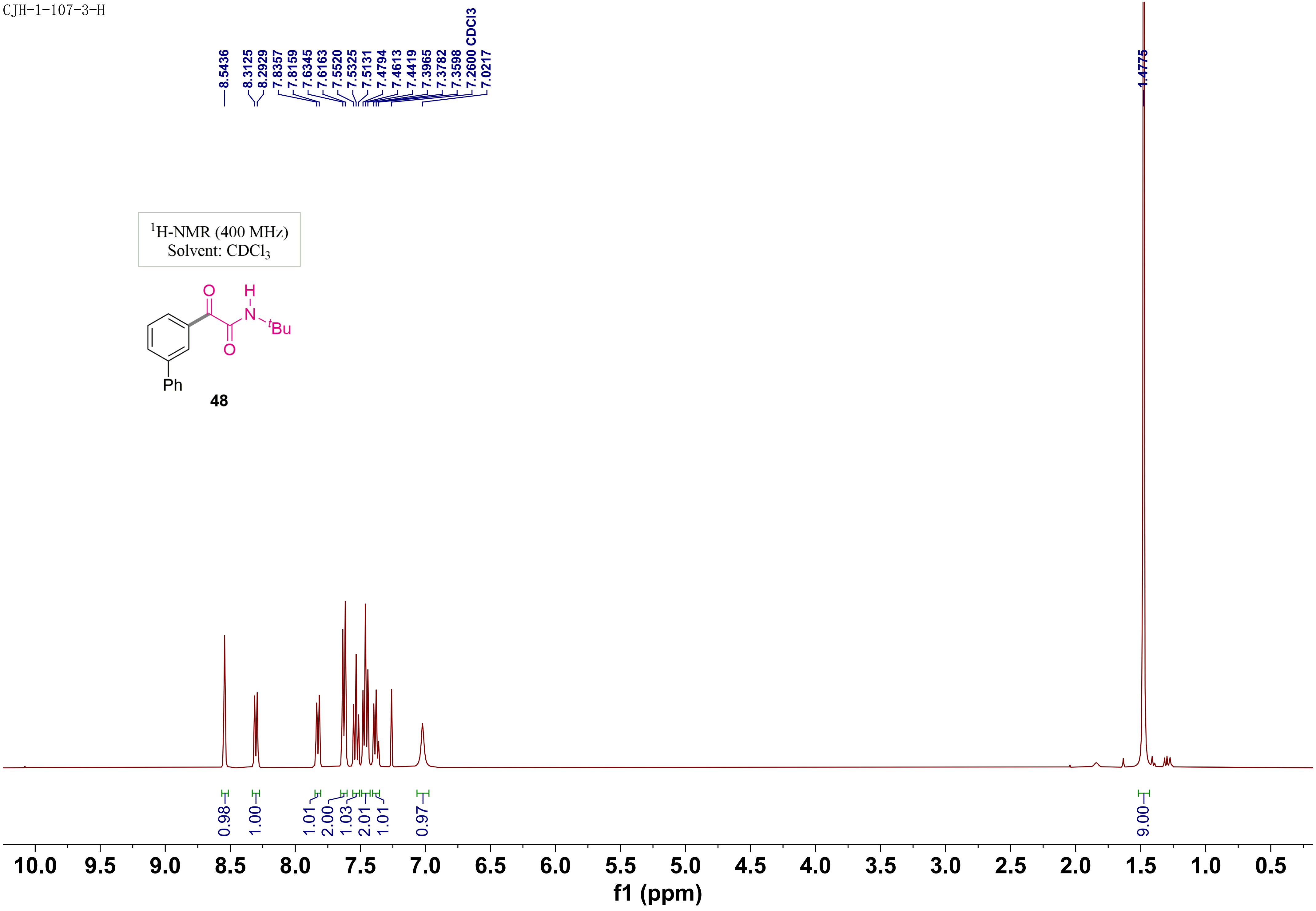
8.5436
8.3125
8.2929
7.8357
7.8159
7.6345
7.6163
7.5520
7.5325
7.5131
7.4794
7.4613
7.4419
7.3965
7.3782
7.3598
7.2600 CDCl3
7.0217
1.4775
^{1}H-NMR (400 MHz)
Solvent: $CDCl_3$
O
H
N
^{t}Bu
O
Ph
48
0.98
1.00
1.01
2.00
1.03
2.01
1.01
0.97
9.00
10.0
9.5
9.0
8.5
8.0
7.5
7.0
6.5
6.0
5.5
5.0
4.5
4.0
3.5
3.0
2.5
2.0
1.5
1.0
0.5
f1 (ppm)

CJH-1-107-3-C

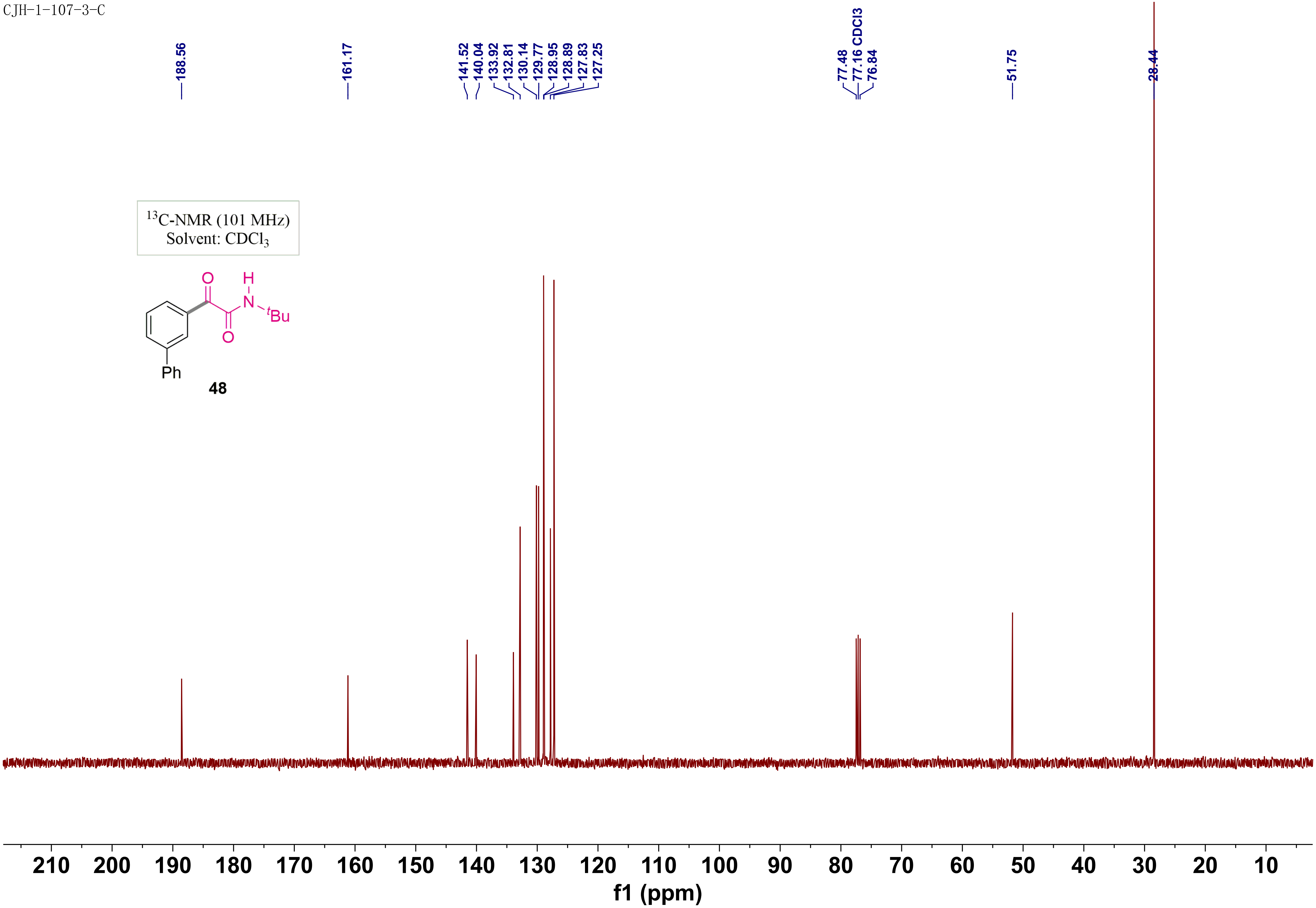
188.56
161.17
141.52
140.04
133.92
132.81
130.14
129.77
128.95
128.89
127.83
127.25
77.48
77.16 CDCl3
76.84
51.75
28.44
^{13}C-NMR (101 MHz)
Solvent: $CDCl_3$
O
H
N
tBu
O
Ph
48
210
200
190
180
170
160
150
140
130
120
110
100
90
80
70
60
50
40
30
20
10
f1 (ppm)

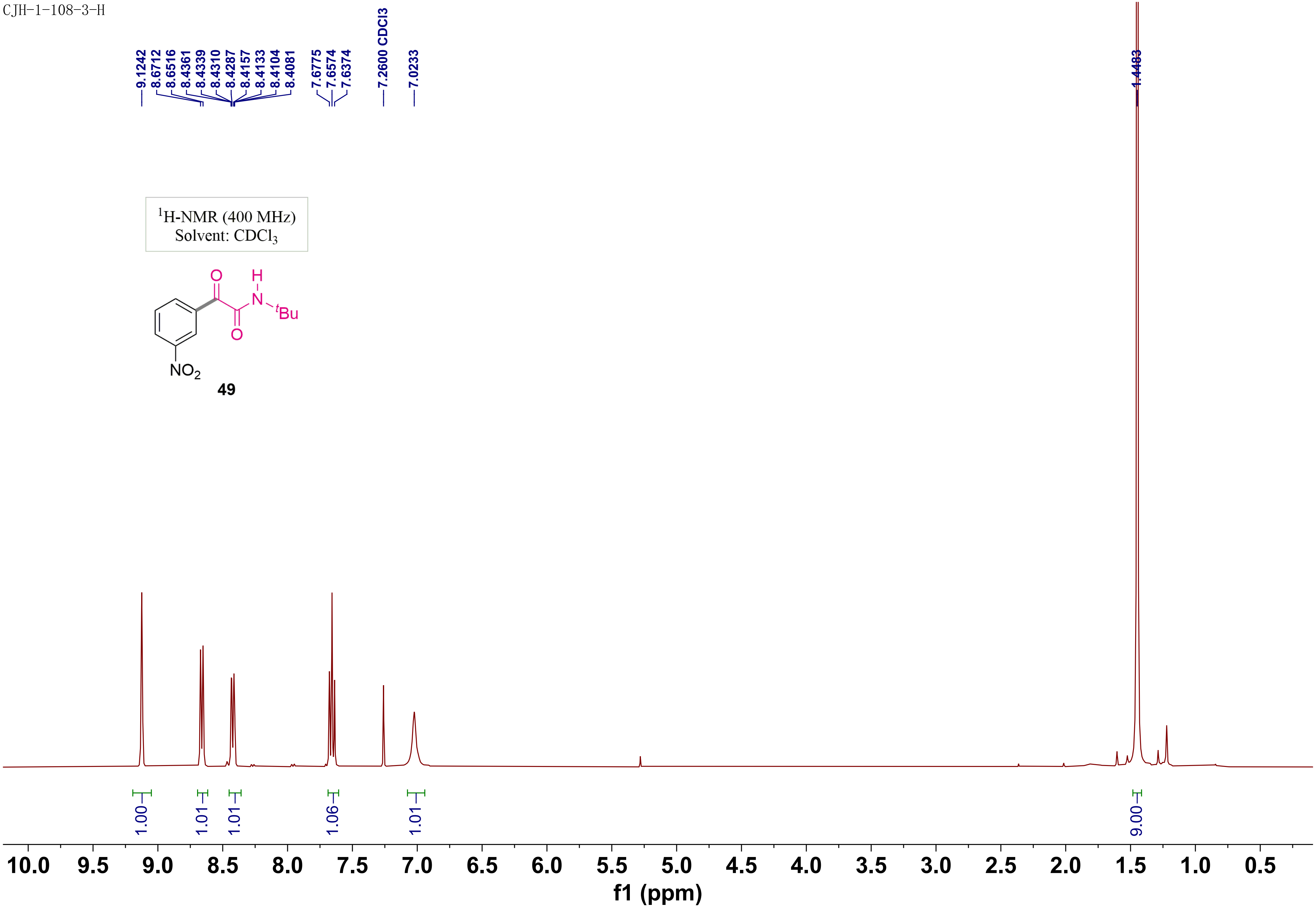
CJH-1-108-3-H
9.1242
8.6712
8.6516
8.4361
8.4339
8.4310
8.4287
8.4157
8.4133
8.4104
8.4081
7.6775
7.6574
7.6374
7.2600 CDCl3
7.0233
1.4483
1H-NMR (400 MHz)
Solvent: CDCl3
O
H
N
tBu
O
NO2
49
1.00
1.01
1.01
1.06
1.01
9.00
10.0
9.5
9.0
8.5
8.0
7.5
7.0
6.5
6.0
5.5
5.0
4.5
4.0
3.5
3.0
2.5
2.0
1.5
1.0
0.5
f1 (ppm)

CJH-1-108-3-C

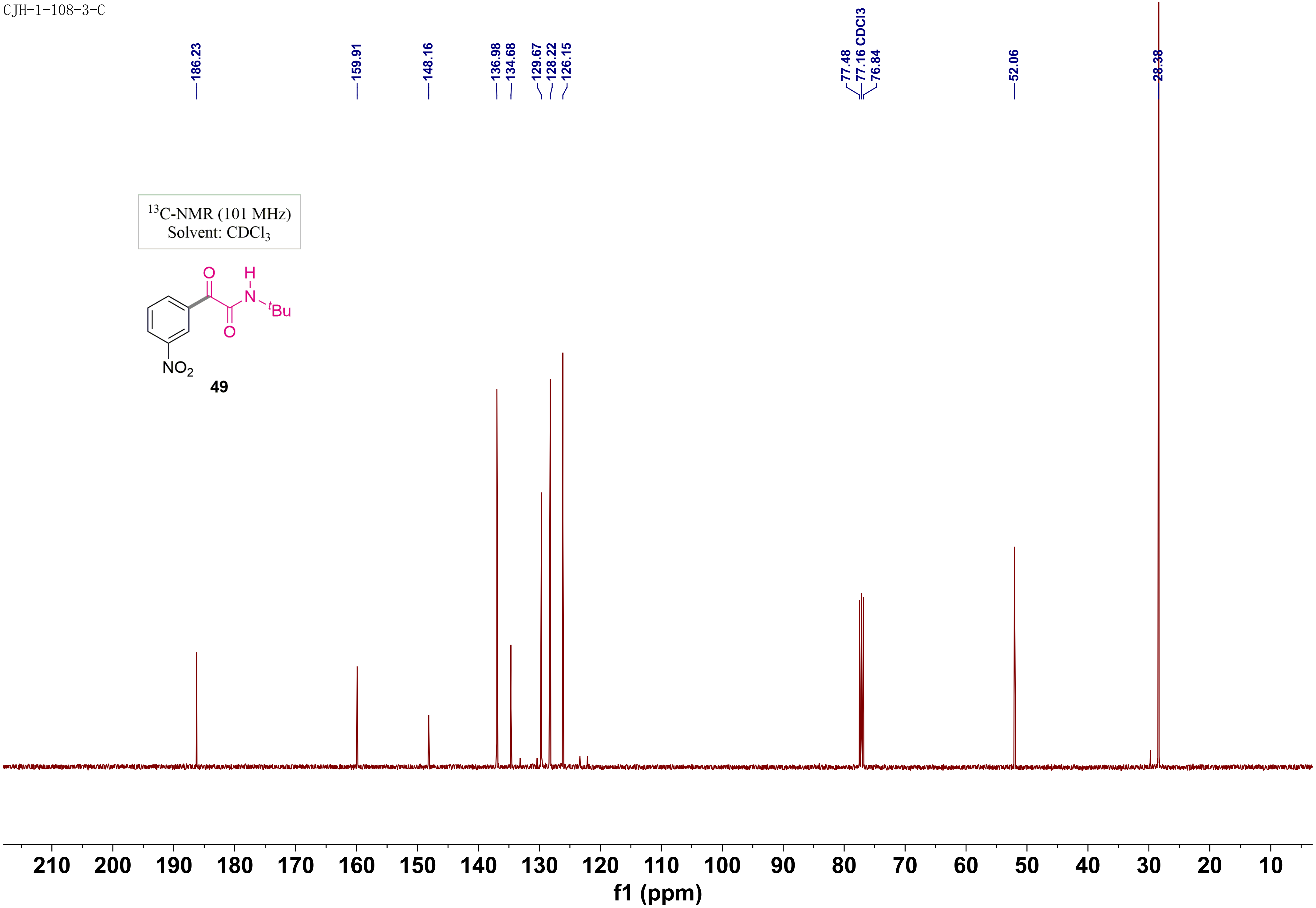

186.23
159.91
148.16
136.98
134.68
129.67
128.22
126.15
77.48
77.16 CDCl3
76.84
52.06
28.38
^{13}C-NMR (101 MHz)
Solvent: $CDCl_3$
O
H
N
^{t}Bu
O
NO_2
49
210 200 190 180 170 160 150 140 130 120 110 100 90 80 70 60 50 40 30 20 10
f1 (ppm)

CJH-1-116-1-H

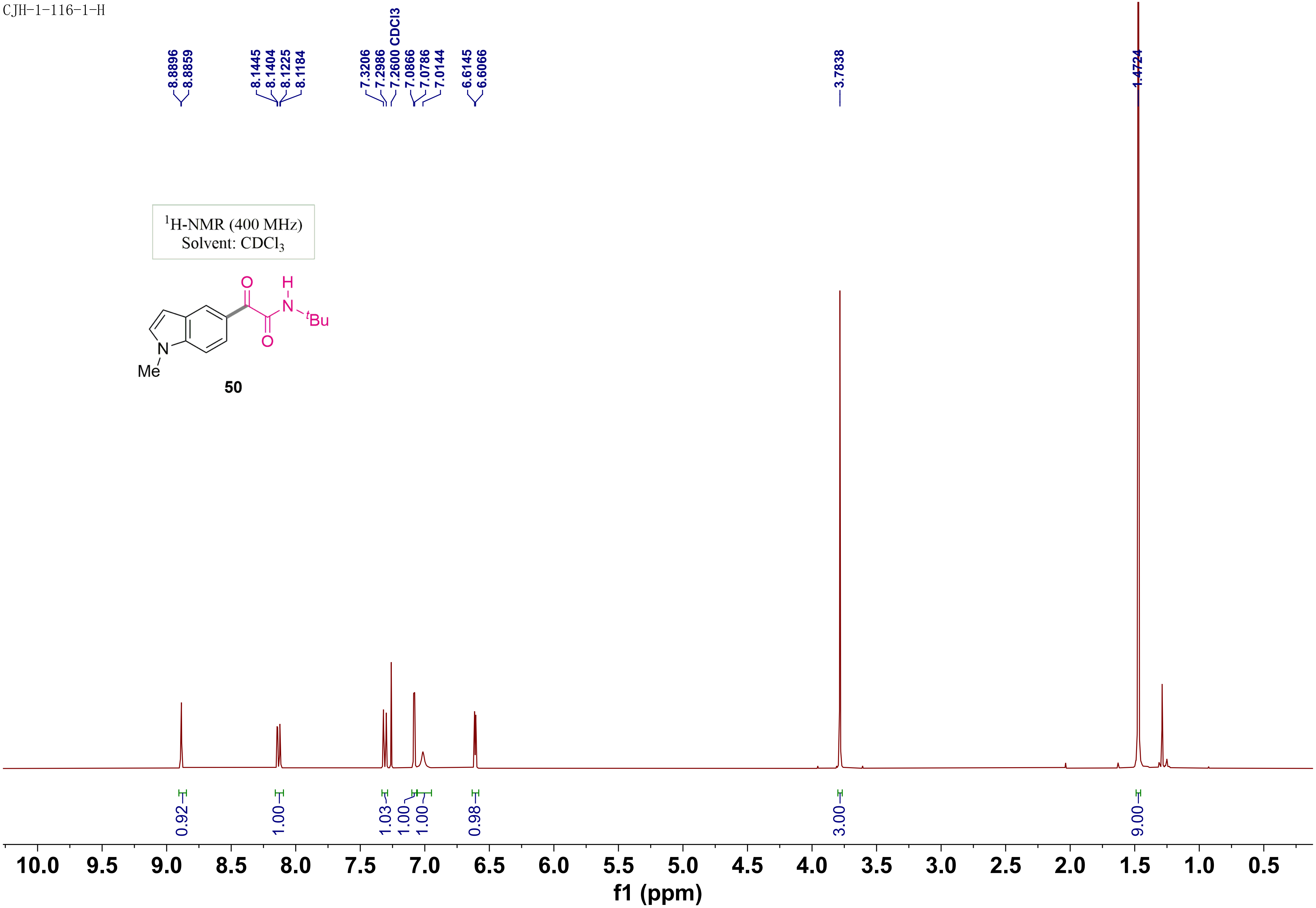
8.8896
8.8859
8.1445
8.1404
8.1225
8.1184
7.3206
7.2986
7.2600 CDCl3
7.0866
7.0786
7.0144
6.6145
6.6066
3.7838
1.4724
1H-NMR (400 MHz)
Solvent: CDCl3
50
0.92
1.00
1.03
1.00
1.00
0.98
3.00
9.00
f1 (ppm)

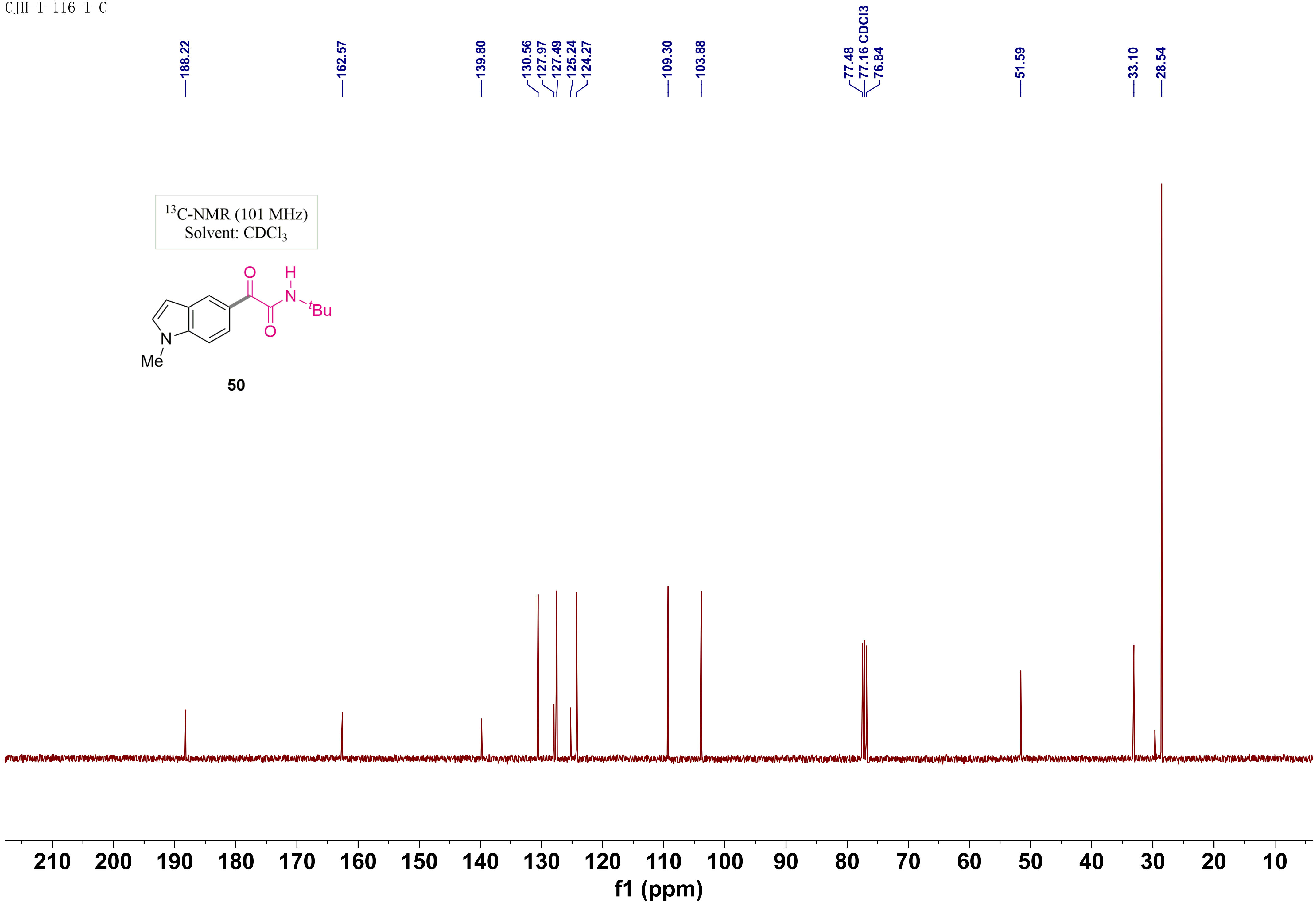
CJH-1-116-1-C
188.22
162.57
139.80
130.56
127.97
127.49
125.24
124.27
109.30
103.88
77.48
77.16 CDCl3
76.84
51.59
33.10
28.54
13C-NMR (101 MHz)
Solvent: CDCl3
O
H
N
tBu
O
N
Me
50
210 200 190 180 170 160 150 140 130 120 110 100 90 80 70 60 50 40 30 20 10
f1 (ppm)

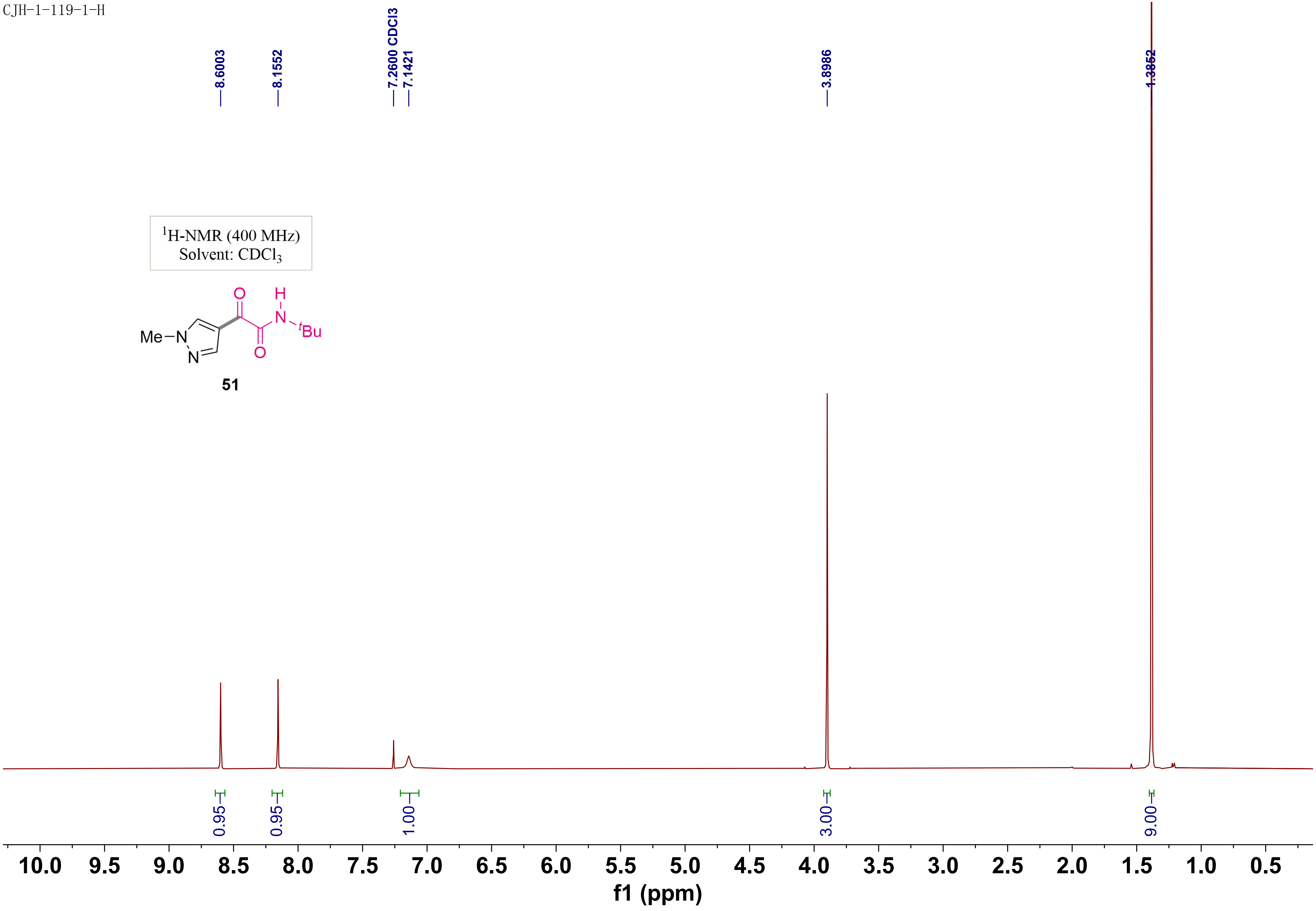
CJH-1-119-1-H
8.6003
8.1552
7.2600 CDCl3
7.1421
3.8986
1.3852
1H-NMR (400 MHz)
Solvent: CDCl3
Me
N
N
O
H
N
tBu
O
51
0.95
0.95
1.00
3.00
9.00
10.0
9.5
9.0
8.5
8.0
7.5
7.0
6.5
6.0
5.5
5.0
4.5
4.0
3.5
3.0
2.5
2.0
1.5
1.0
0.5
f1 (ppm)

CJH-1-119-1-C

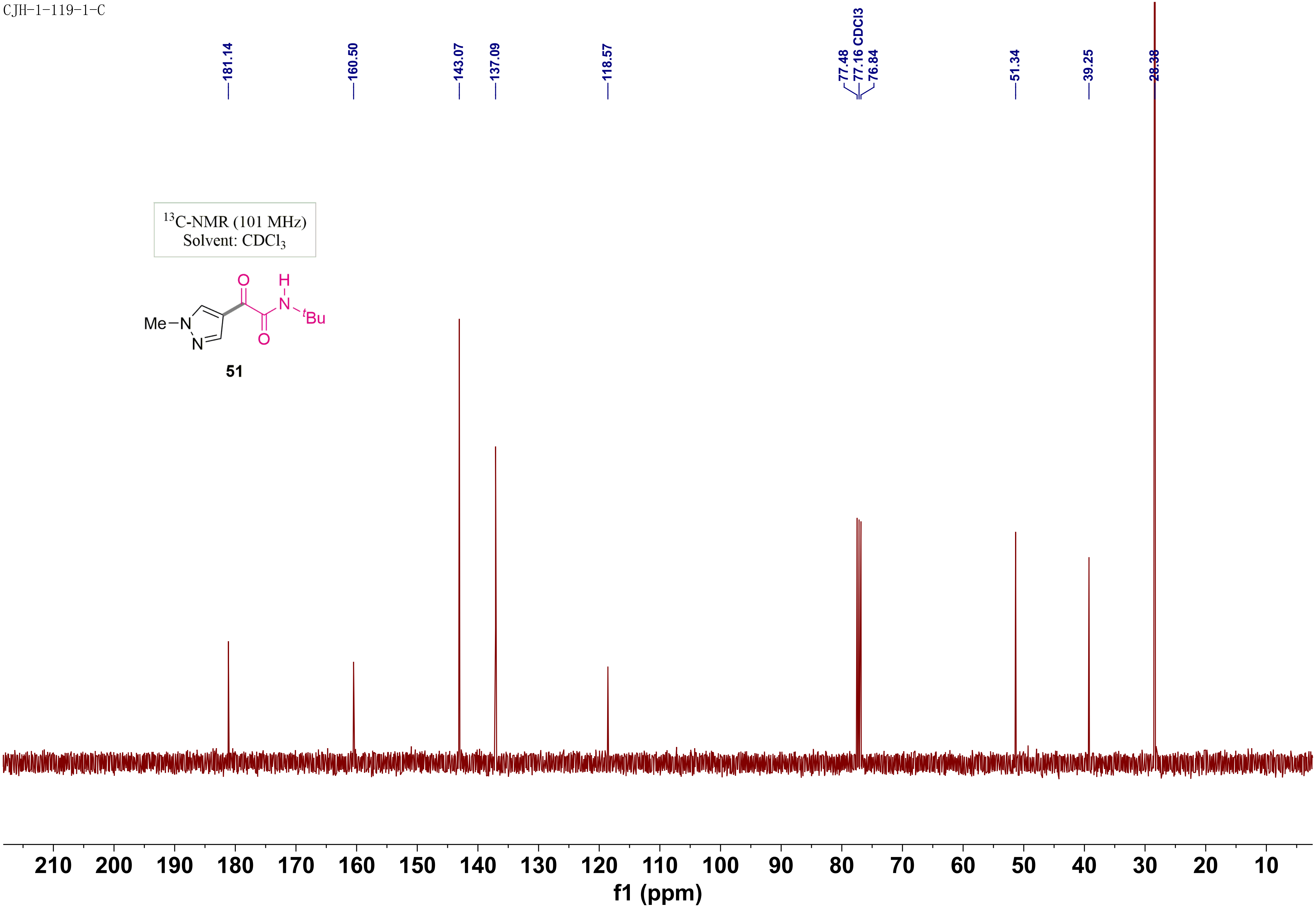
181.14
160.50
143.07
137.09
118.57
77.48
77.16 CDCl3
76.84
51.34
39.25
28.38
13C-NMR (101 MHz)
Solvent: CDCl3
Me
N
N
O
O
H
N
tBu
51
210
200
190
180
170
160
150
140
130
120
110
100
90
80
70
60
50
40
30
20
10
f1 (ppm)

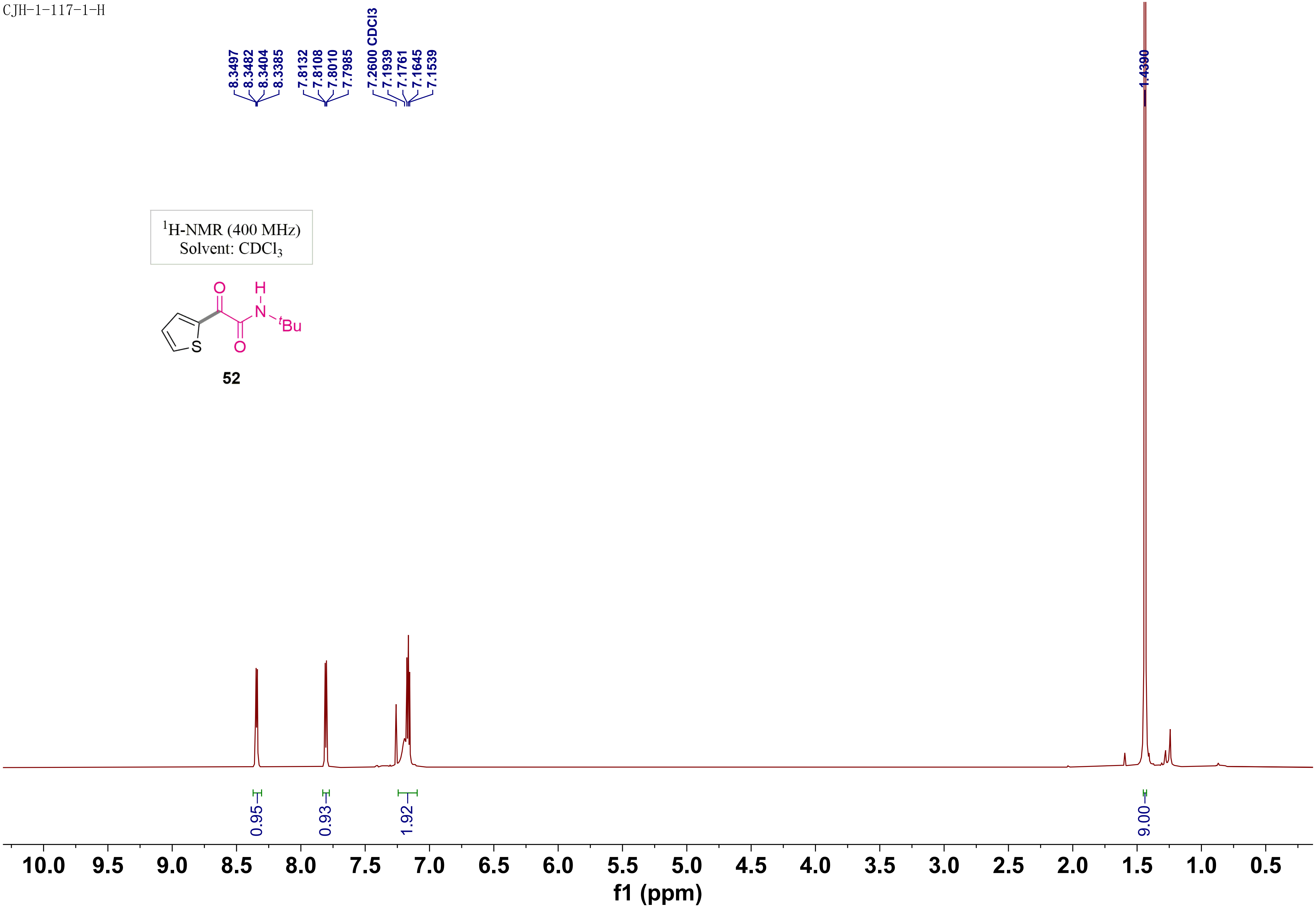
CJH-1-117-1-H
8.3497
8.3482
8.3404
8.3385
7.8132
7.8108
7.8010
7.7985
7.2600 CDCl3
7.1939
7.1761
7.1645
7.1539
1.4390
^{1}H-NMR (400 MHz)
Solvent: $CDCl_3$
O
H
N
^{t}Bu
S
O
52
0.95
0.93
1.92
9.00
10.0 9.5 9.0 8.5 8.0 7.5 7.0 6.5 6.0 5.5 5.0 4.5 4.0 3.5 3.0 2.5 2.0 1.5 1.0 0.5
f1 (ppm)

CJH-1-117-1-C

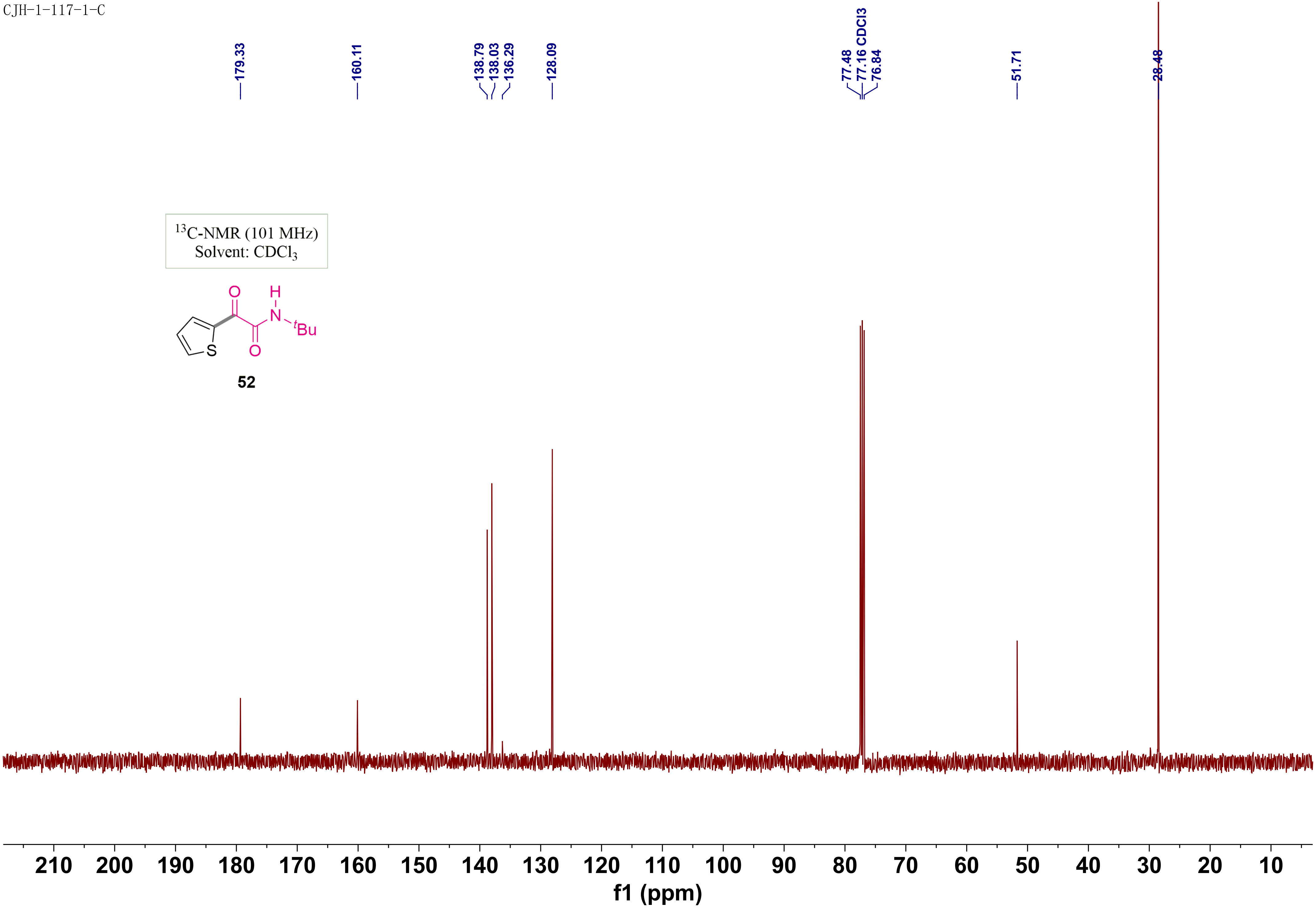

179.33
160.11
138.79
138.03
136.29
128.09
77.48
77.16 CDCl3
76.84
51.71
28.48
^{13}C-NMR (101 MHz)
Solvent: $CDCl_3$
O
H
N
tBu
S
O
52
210
200
190
180
170
160
150
140
130
120
110
100
90
80
70
60
50
40
30
20
10
f1 (ppm)

CJH-1-117-2-H

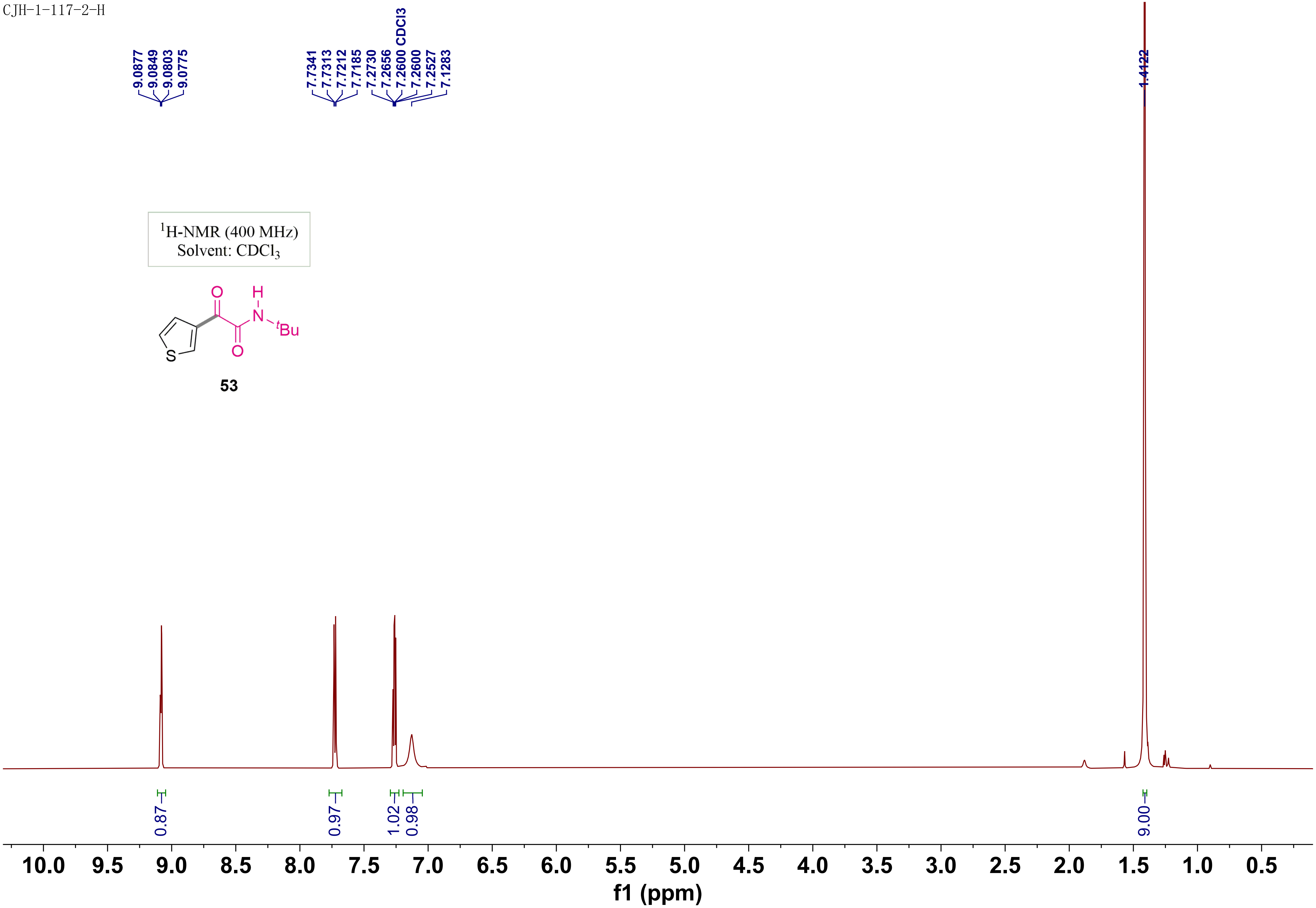

^{1}H-NMR (400 MHz)
Solvent: $CDCl_3$

53

CJH-1-117-2-C

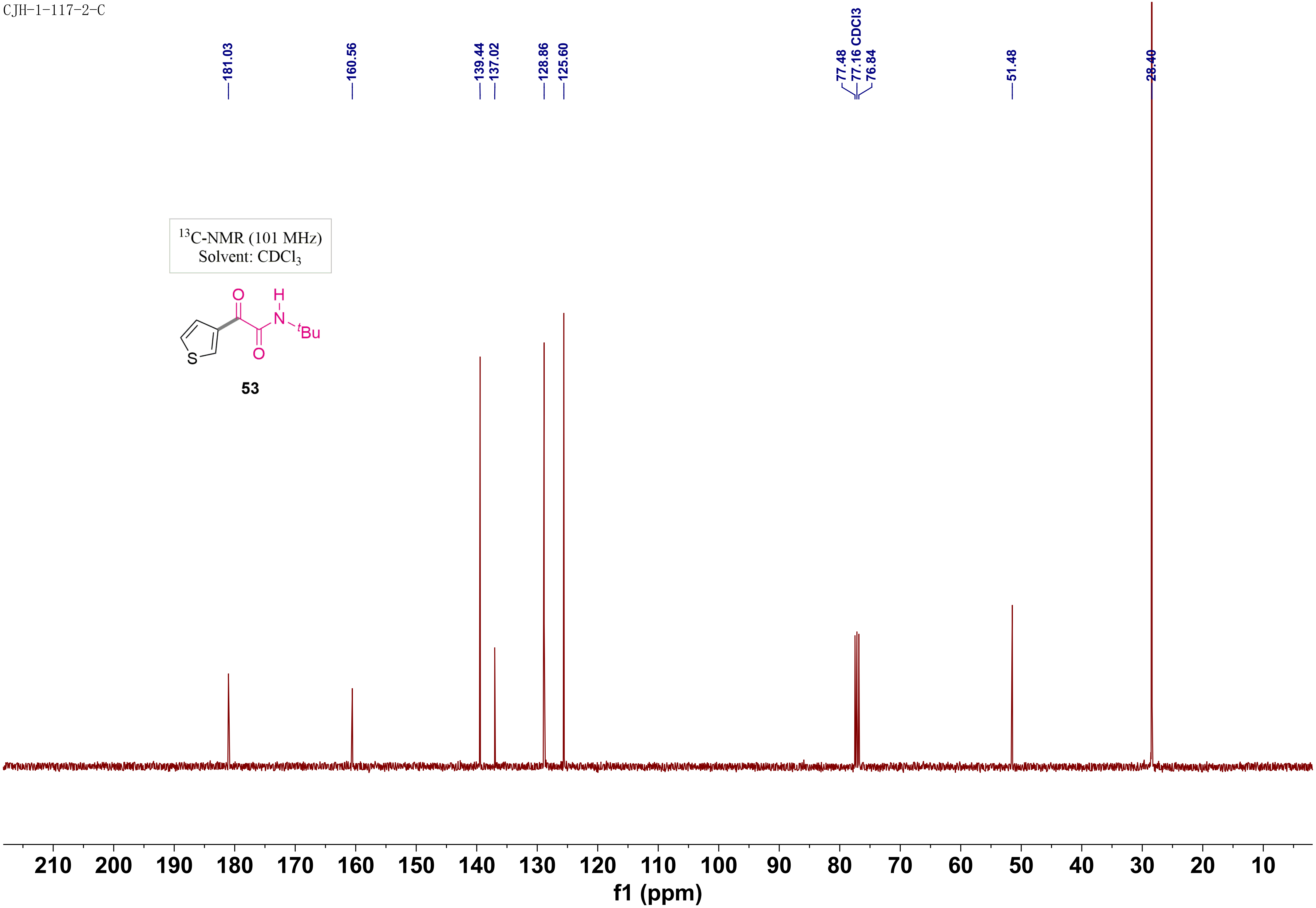

181.03
160.56
139.44
137.02
128.86
125.60
77.48
77.16 CDCl3
76.84
51.48
28.40
^{13}C-NMR (101 MHz)
Solvent: $CDCl_3$
O
H
N
^{t}Bu
O
S
53
210
200
190
180
170
160
150
140
130
120
110
100
90
80
70
60
50
40
30
20
10
f1 (ppm)

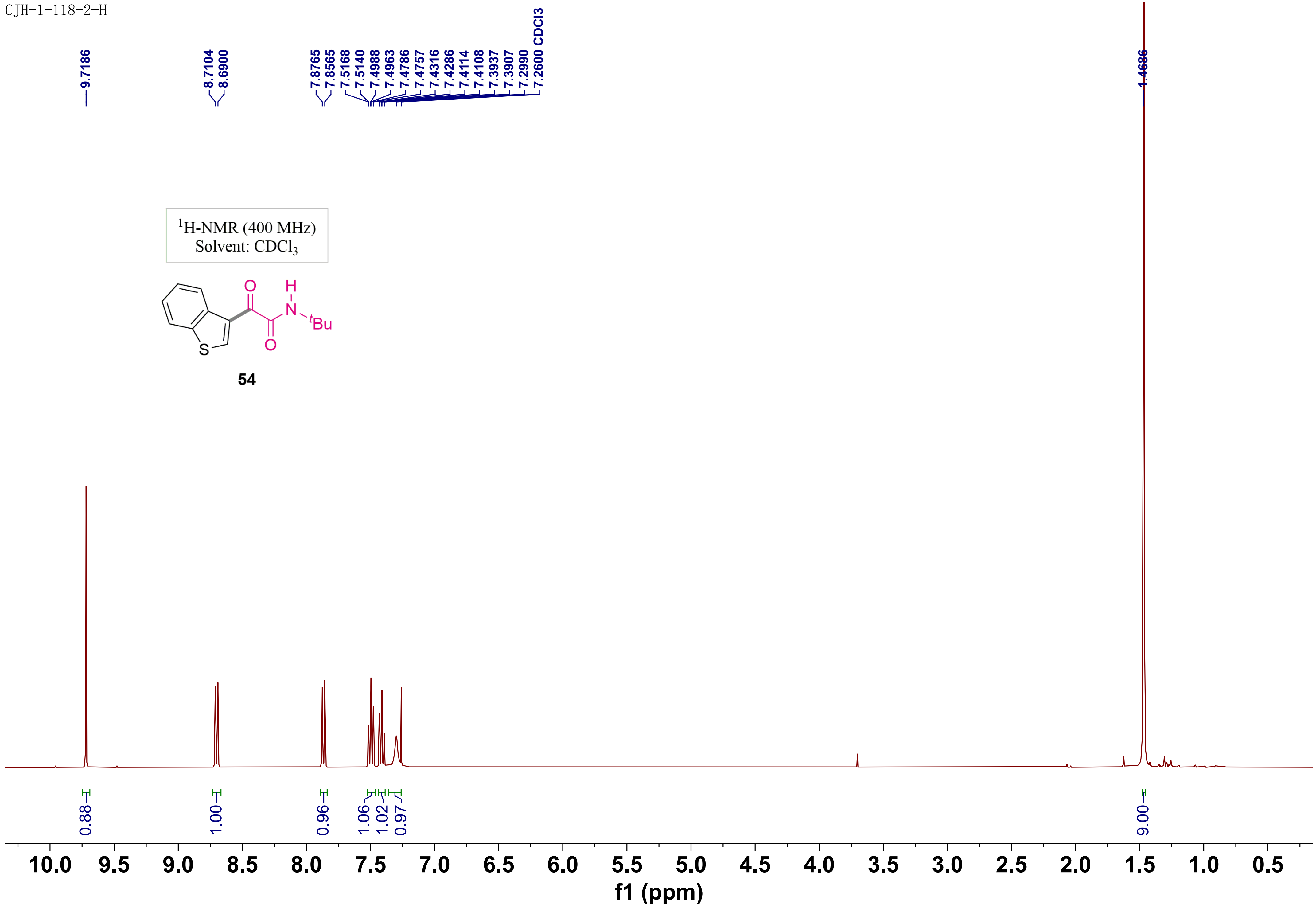
CJH-1-118-2-H
9.7186
8.7104
8.6900
7.8765
7.8565
7.5168
7.5140
7.4988
7.4963
7.4786
7.4757
7.4316
7.4286
7.4114
7.4108
7.3937
7.3907
7.2990
7.2600 CDCl3
1.4686
1H-NMR (400 MHz)
Solvent: CDCl3
O
H
N
tBu
S
O
54
0.88
1.00
0.96
1.06
1.02
0.97
9.00
10.0
9.5
9.0
8.5
8.0
7.5
7.0
6.5
6.0
5.5
5.0
4.5
4.0
3.5
3.0
2.5
2.0
1.5
1.0
0.5
f1 (ppm)

CJH-1-118-2-C

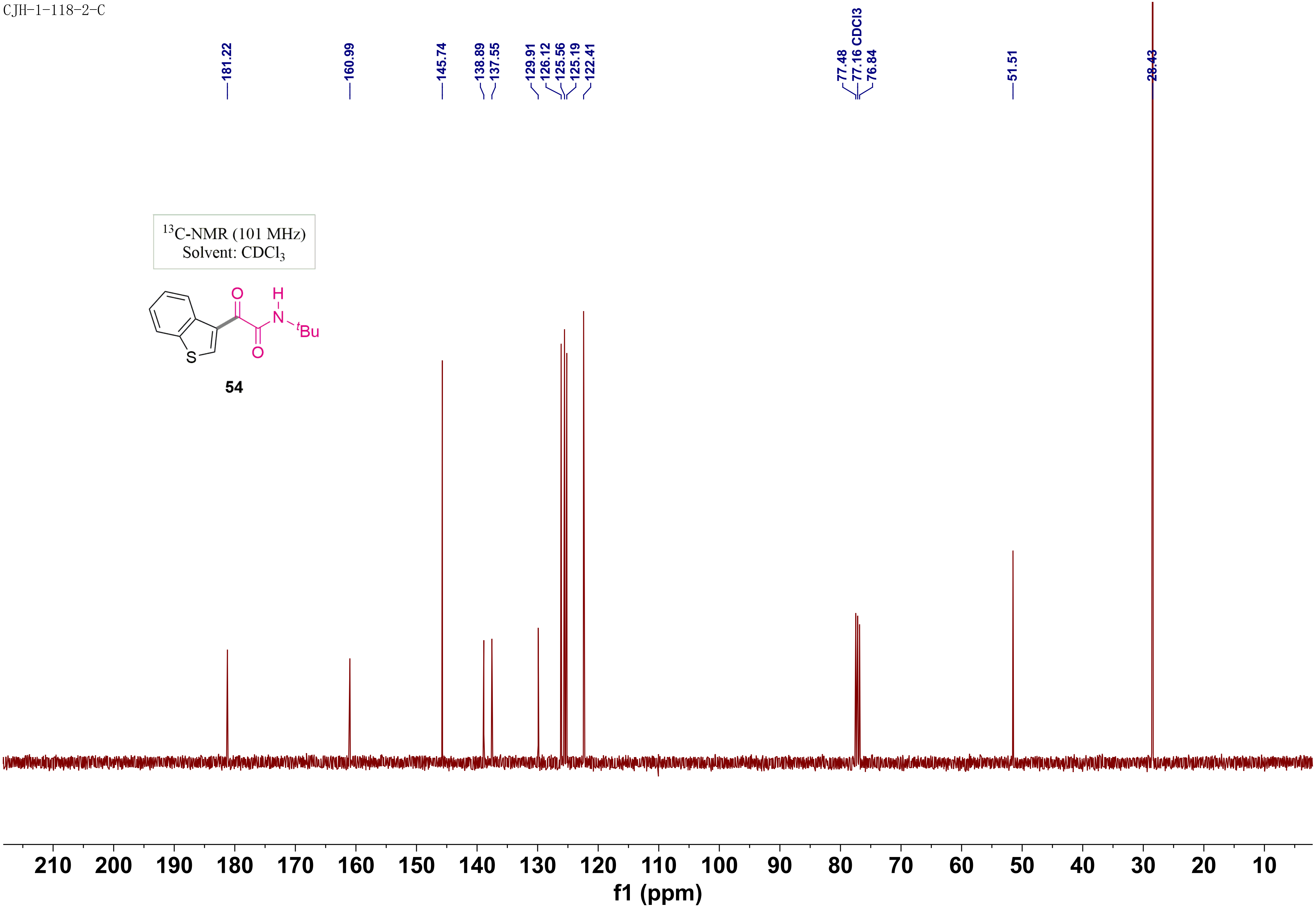

181.22
160.99
145.74
138.89
137.55
129.91
126.12
125.56
125.19
122.41
77.48
77.16 CDCl3
76.84
51.51
28.43
^{13}C-NMR (101 MHz)
Solvent: $CDCl_3$
O
H
N
tBu
O
S
54
210 200 190 180 170 160 150 140 130 120 110 100 90 80 70 60 50 40 30 20 10
f1 (ppm)

CJH-1-119-2-H

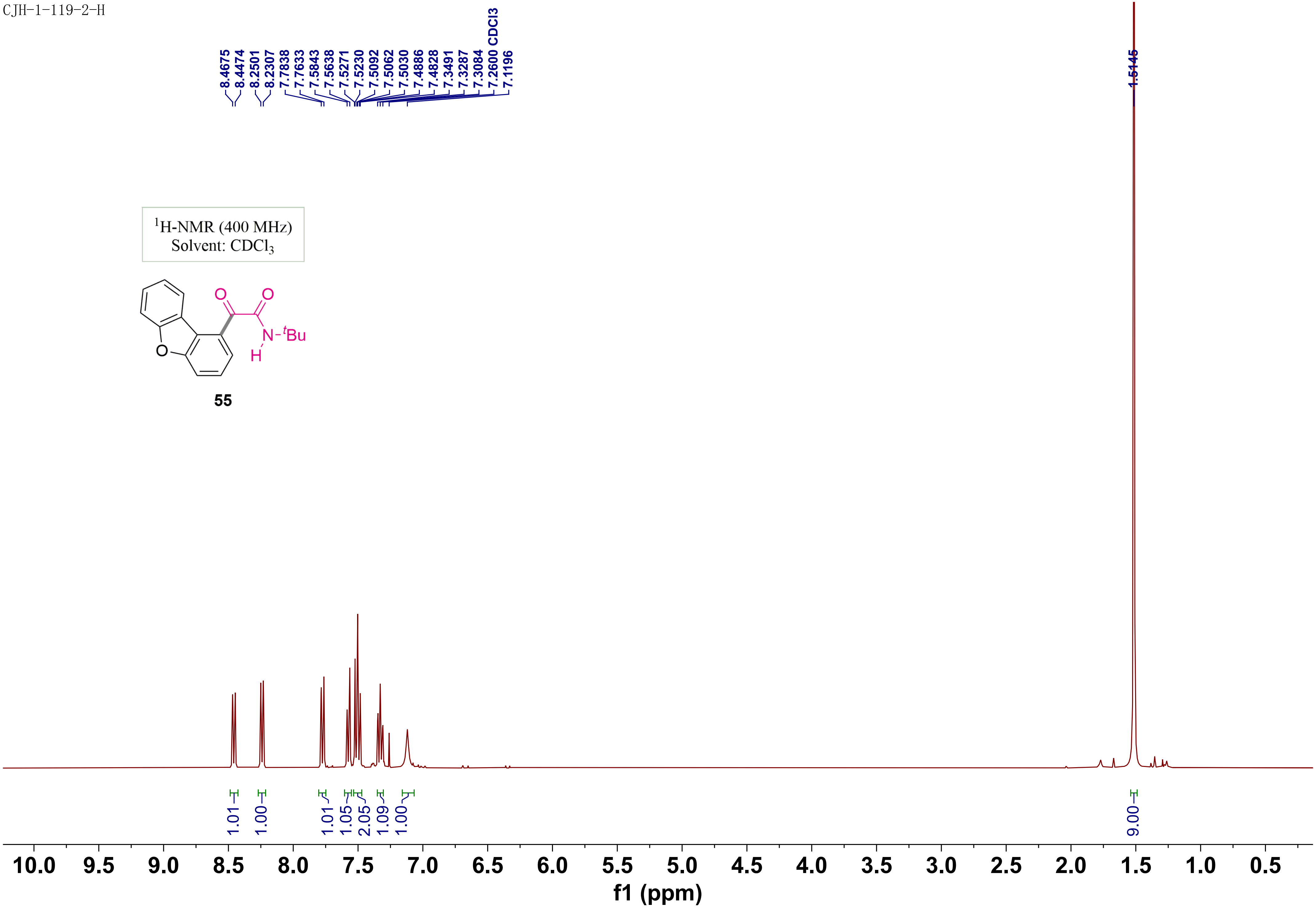

^{1}H-NMR (400 MHz)
Solvent: $CDCl_3$
55
8.4675
8.4474
8.2501
8.2307
7.7838
7.7633
7.5843
7.5638
7.5271
7.5230
7.5092
7.5062
7.5030
7.4886
7.4828
7.3491
7.3287
7.3084
7.2600 CDCl3
7.1196
1.5145
1.01
1.00
1.01
1.05
2.05
1.09
1.00
9.00
10.0
9.5
9.0
8.5
8.0
7.5
7.0
6.5
6.0
5.5
5.0
4.5
4.0
3.5
3.0
2.5
2.0
1.5
1.0
0.5
f1 (ppm)

CJH-1-119-2-C

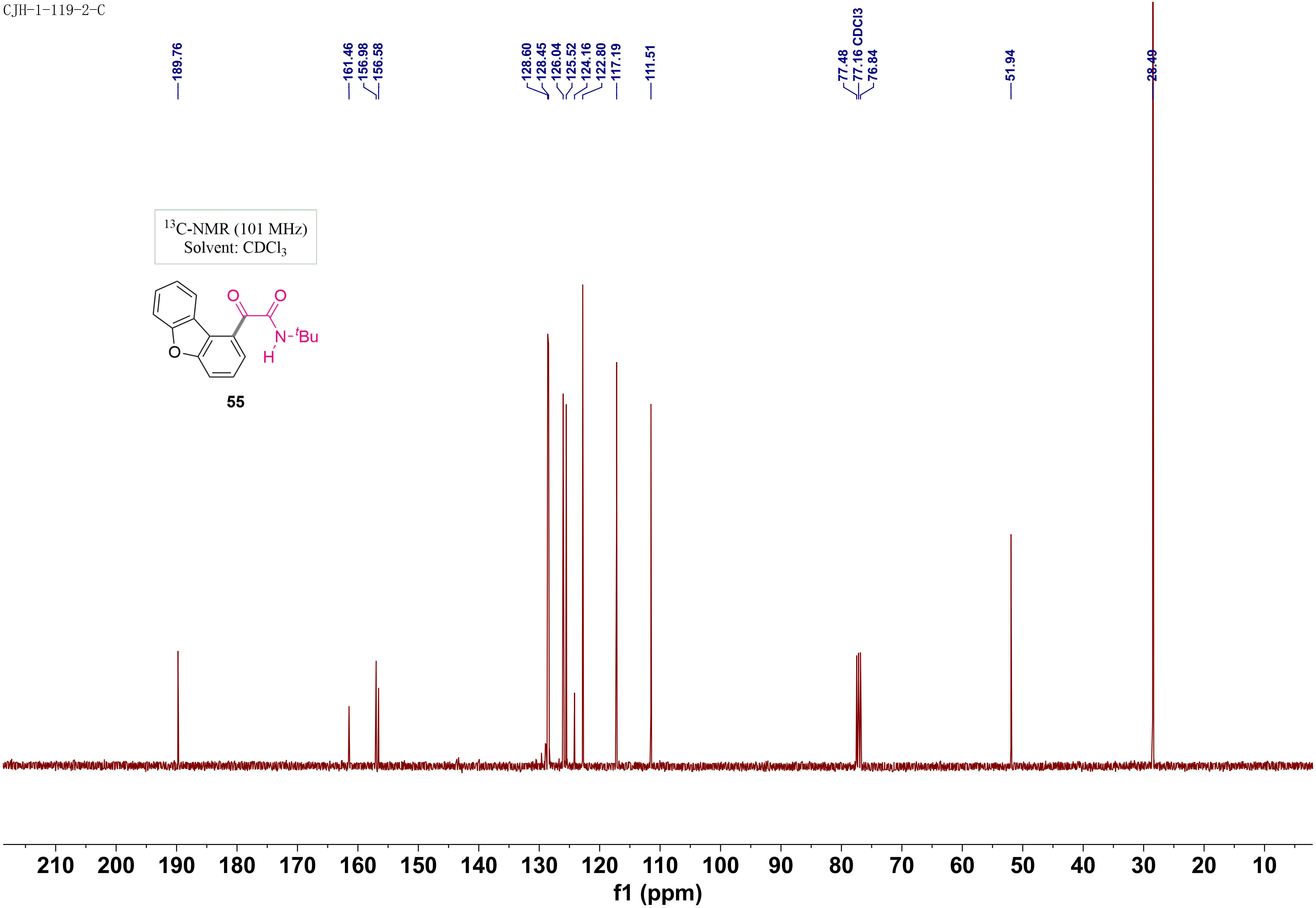

189.76
161.46
156.98
156.58
128.60
128.45
126.04
125.52
124.16
122.80
117.19
111.51
77.48
77.16 CDCl3
76.84
51.94
28.49
^{13}C-NMR (101 MHz)
Solvent: $CDCl_3$
O
O
N-^{t}Bu
H
O
55
210
200
190
180
170
160
150
140
130
120
110
100
90
80
70
60
50
40
30
20
10
f1 (ppm)

CJH-1-120-3-H

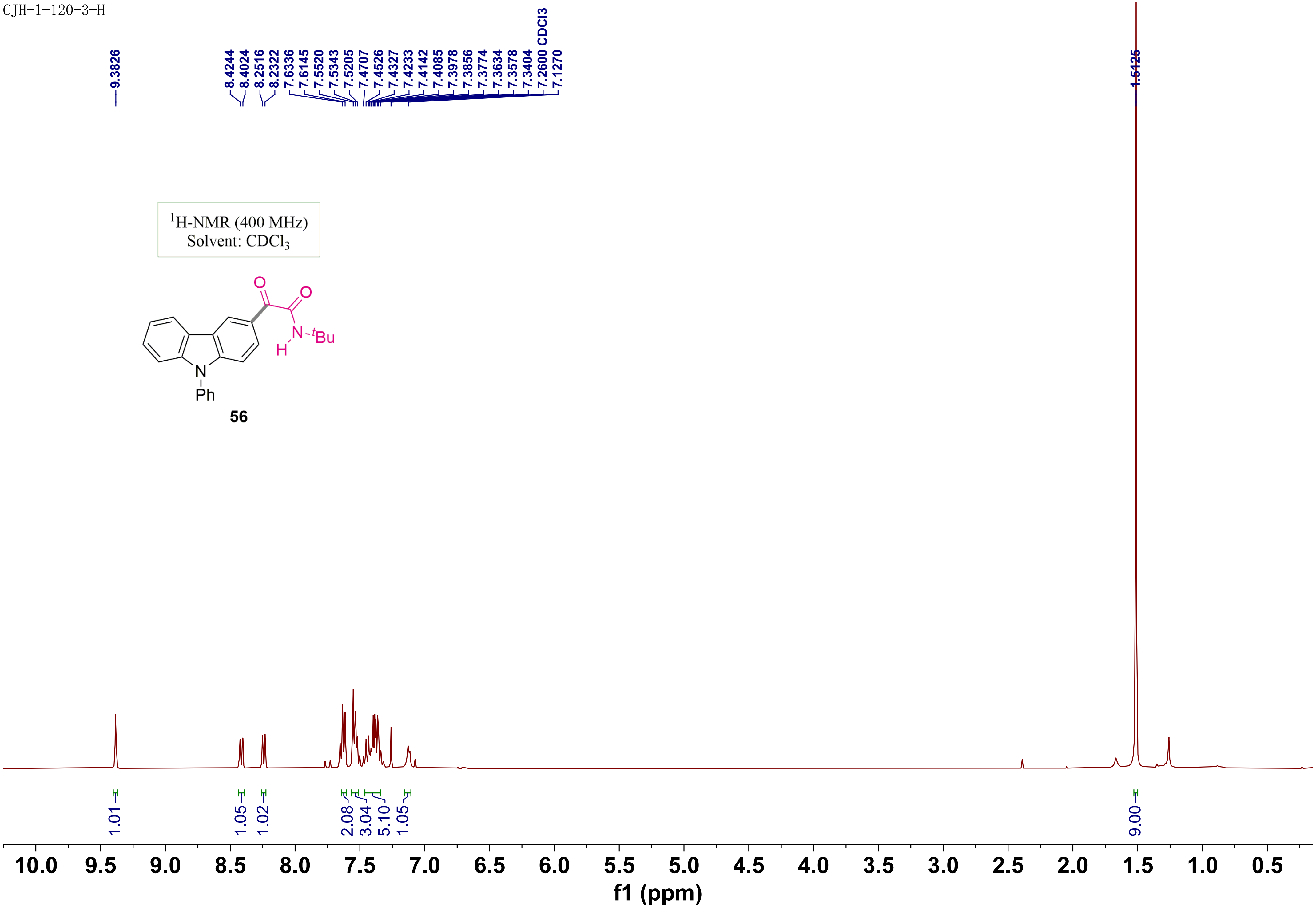
9.3826
8.4244
8.4024
8.2516
8.2322
7.6336
7.6145
7.5520
7.5343
7.5205
7.4707
7.4526
7.4327
7.4233
7.4142
7.4085
7.3978
7.3856
7.3774
7.3634
7.3578
7.3404
7.2600 CDCl3
7.1270
1.5125
^{1}H-NMR (400 MHz)
Solvent: $CDCl_3$
O
O
N
^{t}Bu
H
N
Ph
56
1.01
1.05
1.02
2.08
3.04
5.10
1.05
9.00
10.0
9.5
9.0
8.5
8.0
7.5
7.0
6.5
6.0
5.5
5.0
4.5
4.0
3.5
3.0
2.5
2.0
1.5
1.0
0.5
f1 (ppm)

CJH-1-120-3-C

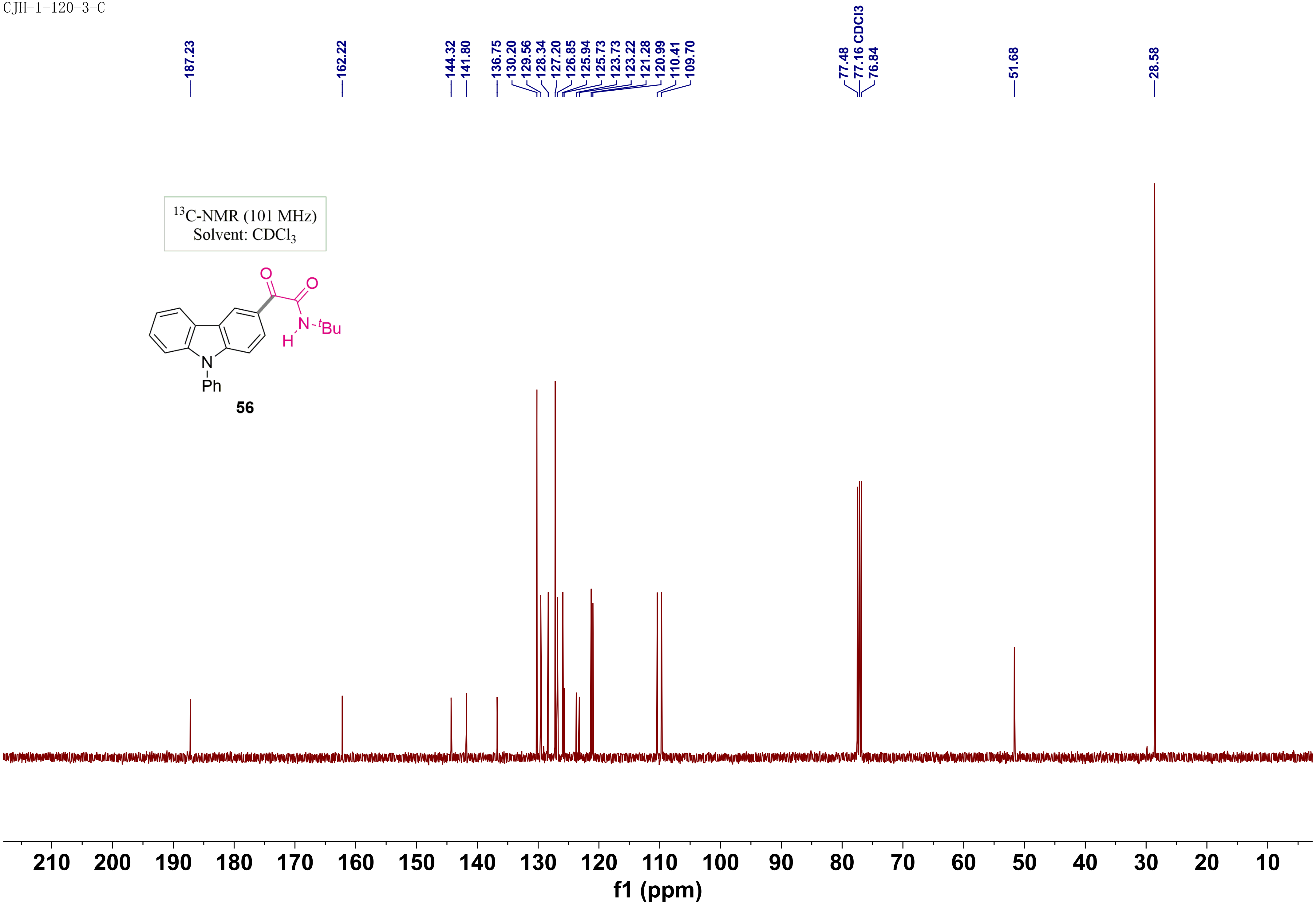
187.23
162.22
144.32
141.80
136.75
130.20
129.56
128.34
127.20
126.85
125.94
125.73
123.73
123.22
121.28
120.99
110.41
109.70
77.48
77.16 CDCl3
76.84
51.68
28.58
^{13}C-NMR (101 MHz)
Solvent: $CDCl_3$
O
O
N
H
^{t}Bu
N
Ph
56
210
200
190
180
170
160
150
140
130
120
110
100
90
80
70
60
50
40
30
20
10
f1 (ppm)

CJH-1-161-1-H

^{1}H-NMR (400 MHz)
Solvent: $CDCl_3$

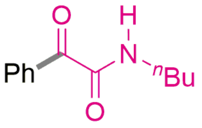

57

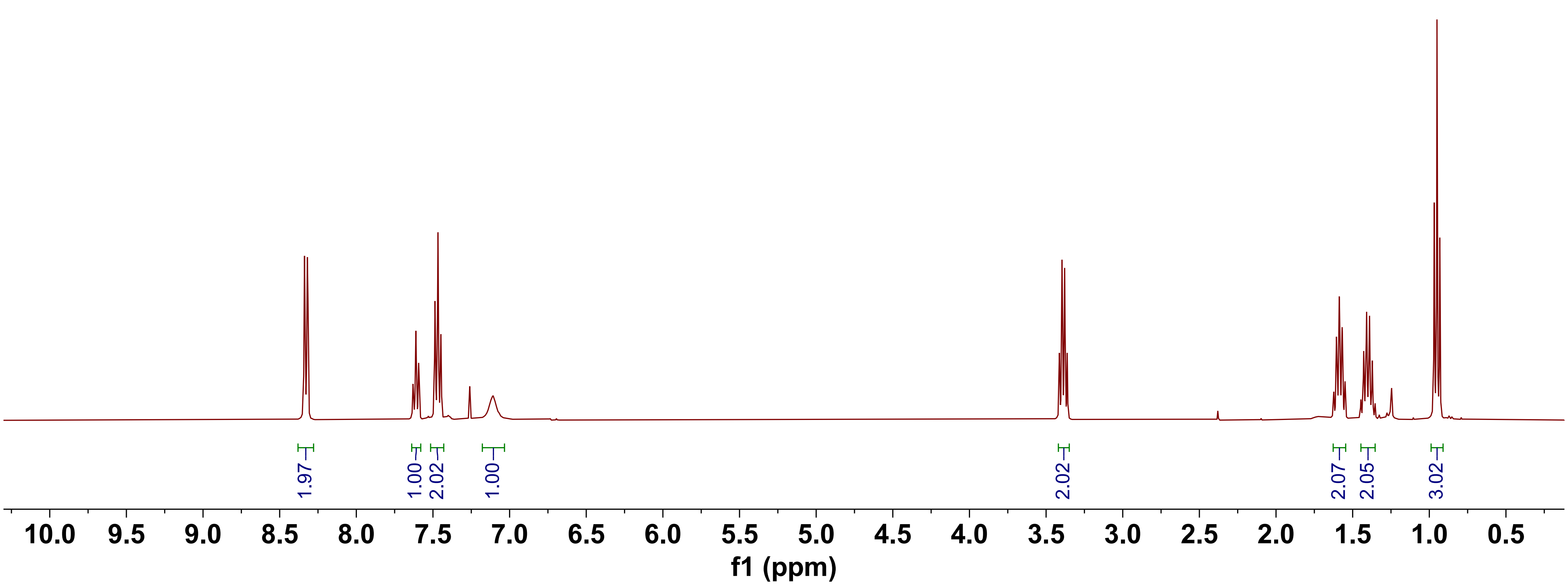

CJH-1-161-1-C

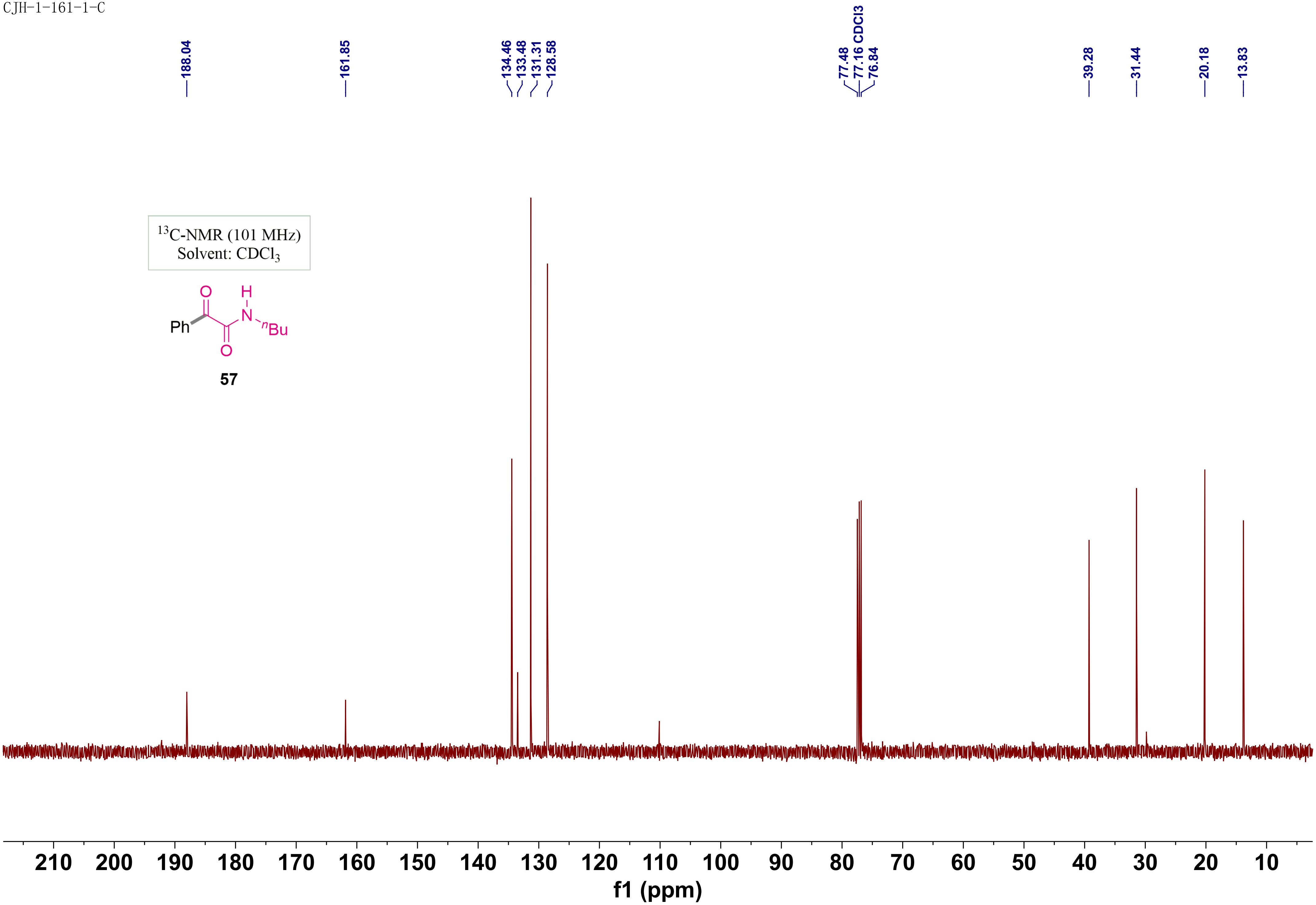
188.04
161.85
134.46
133.48
131.31
128.58
77.48
77.16 CDCl3
76.84
39.28
31.44
20.18
13.83
^{13}C-NMR (101 MHz)
Solvent: $CDCl_3$
O
H
N
Ph
^{n}Bu
O
57
210
200
190
180
170
160
150
140
130
120
110
100
90
80
70
60
50
40
30
20
10
f1 (ppm)

CJH-1-169-2-H

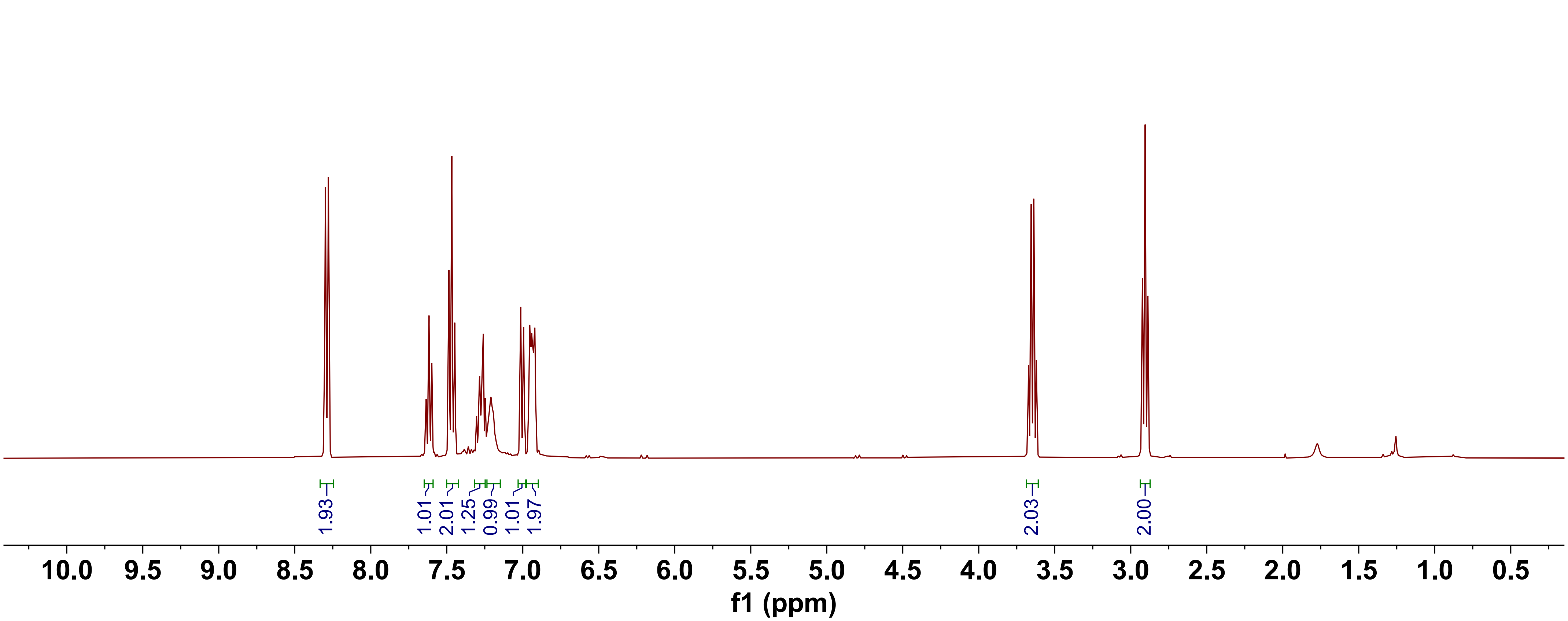

^{1}H-NMR (400 MHz)
Solvent: $CDCl_3$

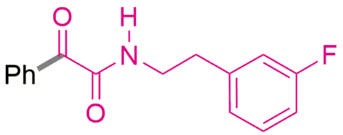

58

CJH-1-169-2-C

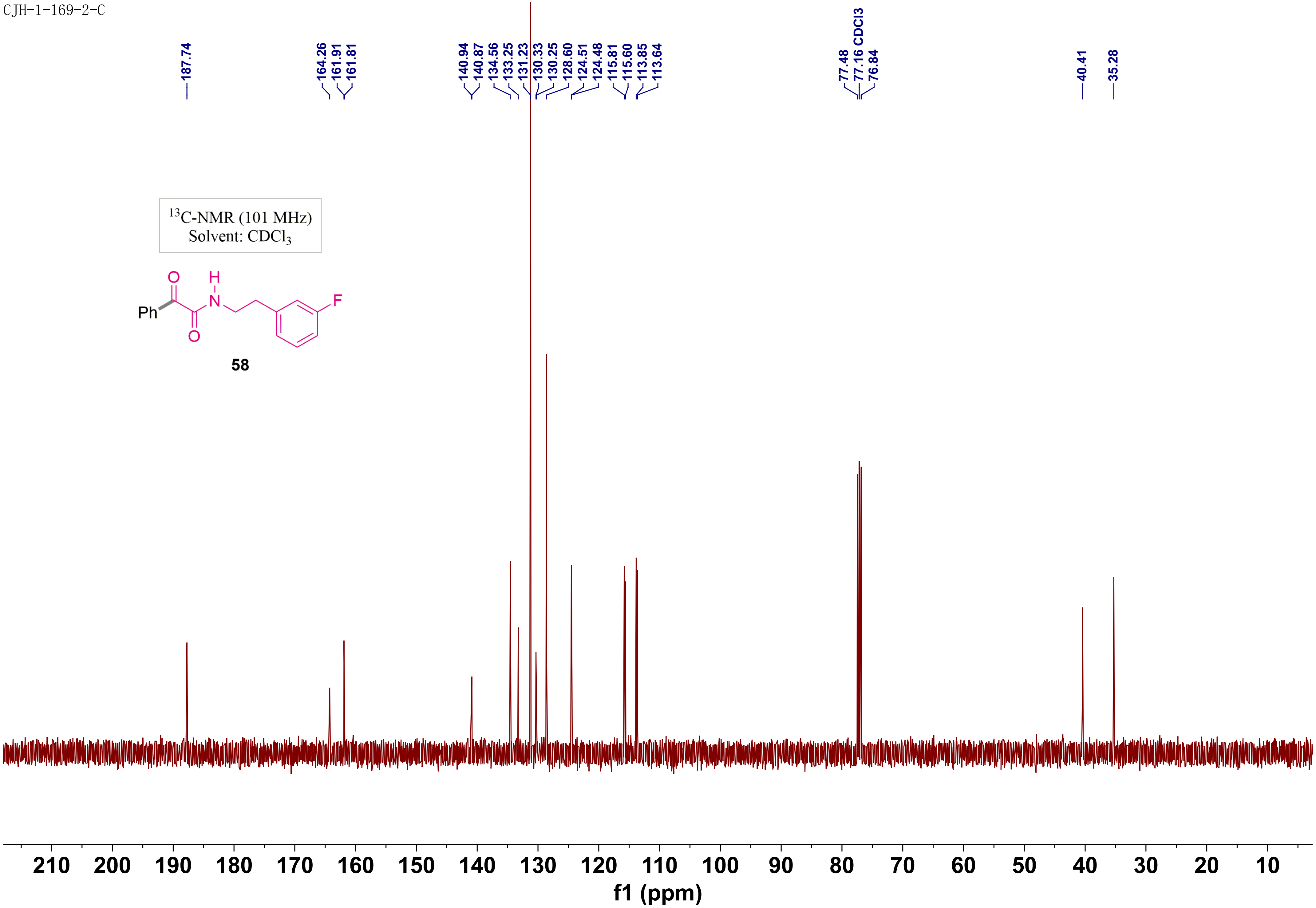

187.74
164.26
161.91
161.81
140.94
140.87
134.56
133.25
131.23
130.33
130.25
128.60
124.51
124.48
115.81
115.60
113.85
113.64
77.48
77.16 CDCl3
76.84
40.41
35.28
13C-NMR (101 MHz)
Solvent: CDCl3
Ph
O
H
N
F
O
58
210 200 190 180 170 160 150 140 130 120 110 100 90 80 70 60 50 40 30 20 10
f1 (ppm)

CJH-1-148-3-H

^{1}H-NMR (400 MHz)
Solvent: $CDCl_3$

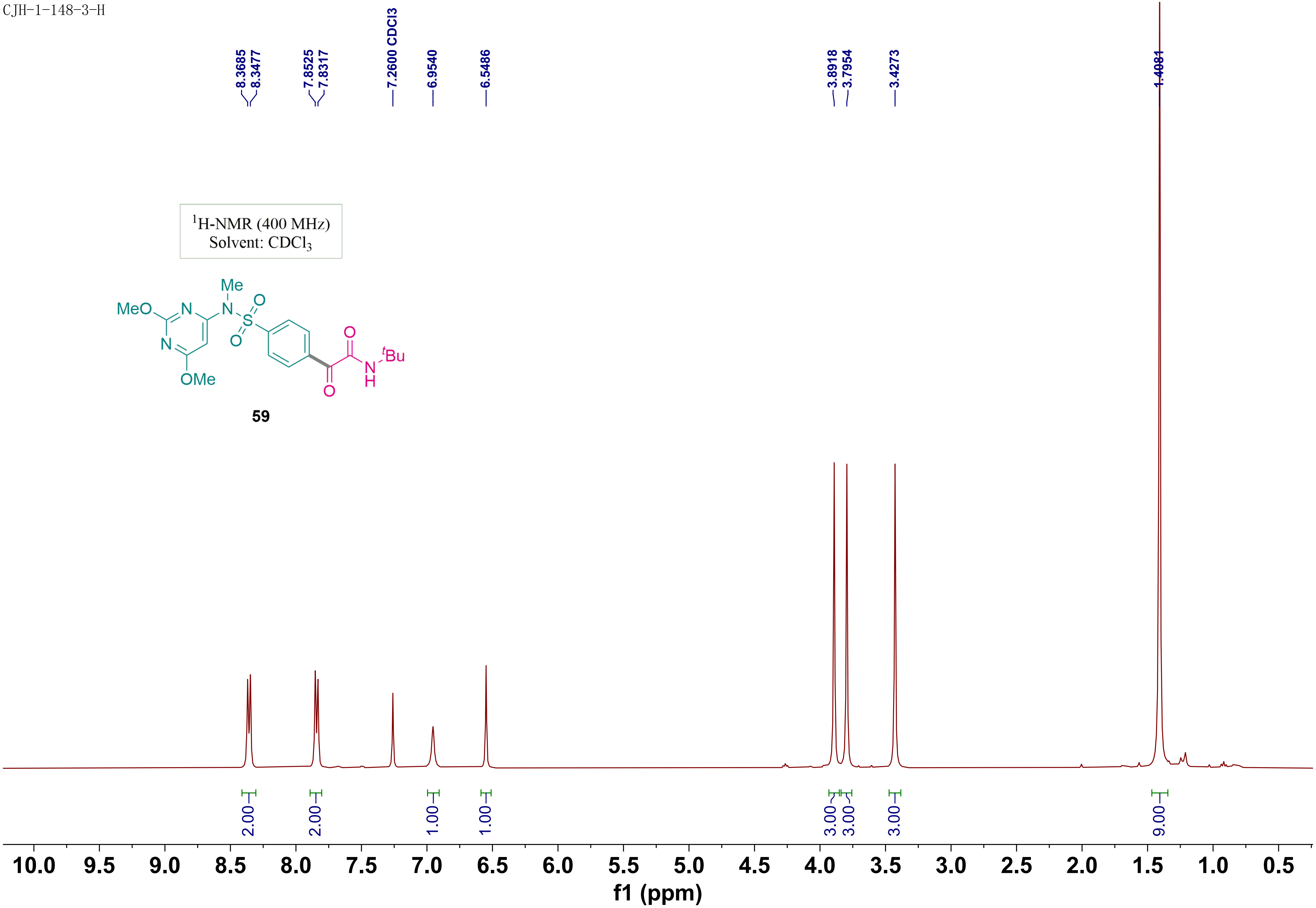

59

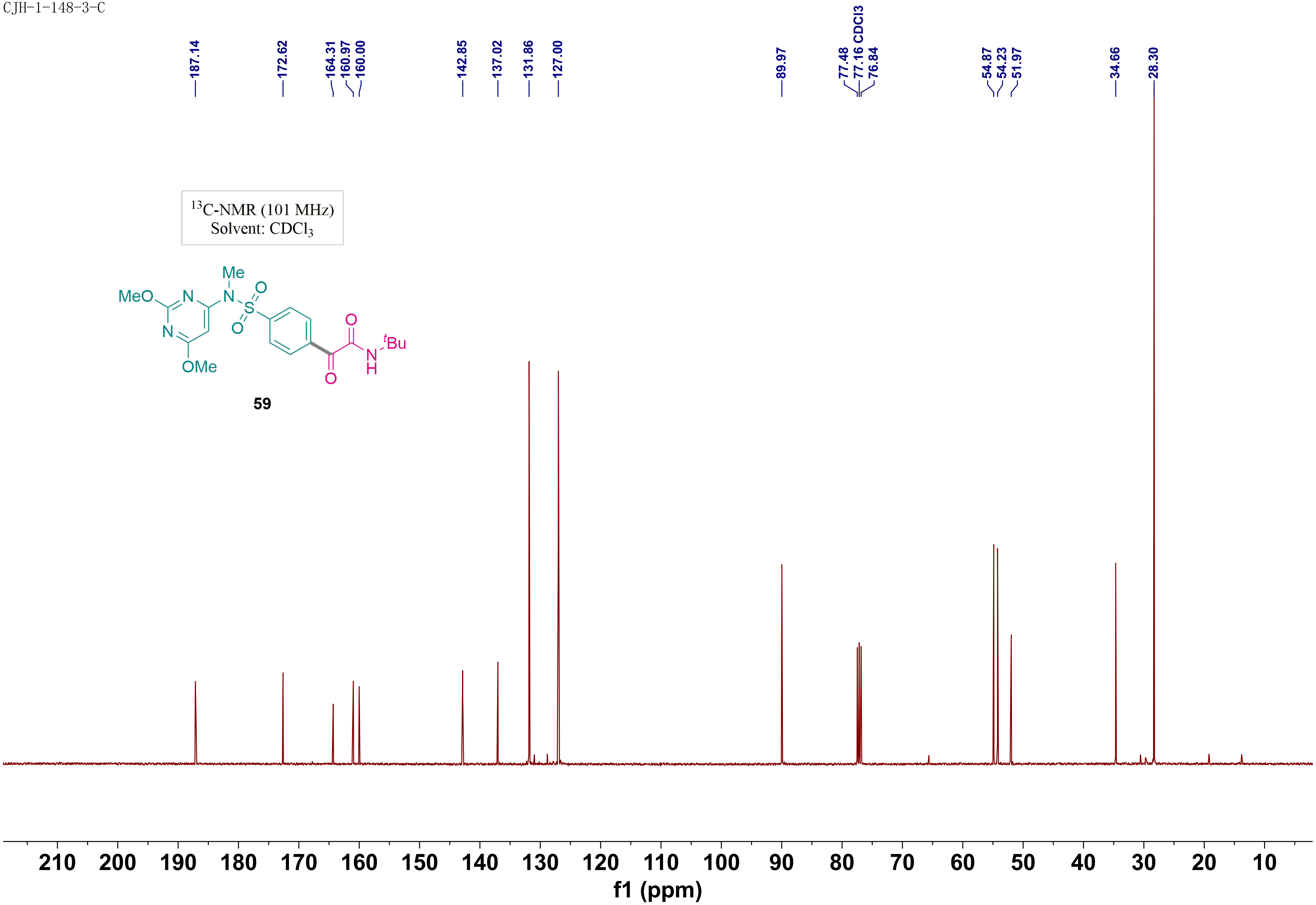
CJH-1-148-3-C
187.14
172.62
164.31
160.97
160.00
142.85
137.02
131.86
127.00
89.97
77.48
77.16 CDCl3
76.84
54.87
54.23
51.97
34.66
28.30
^{13}C-NMR (101 MHz)
Solvent: $CDCl_3$
MeO
N
N
OMe
Me
N
S
O
O
O
O
N
H
^{t}Bu
59
210 200 190 180 170 160 150 140 130 120 110 100 90 80 70 60 50 40 30 20 10
f1 (ppm)

CJH-1-140-2-H

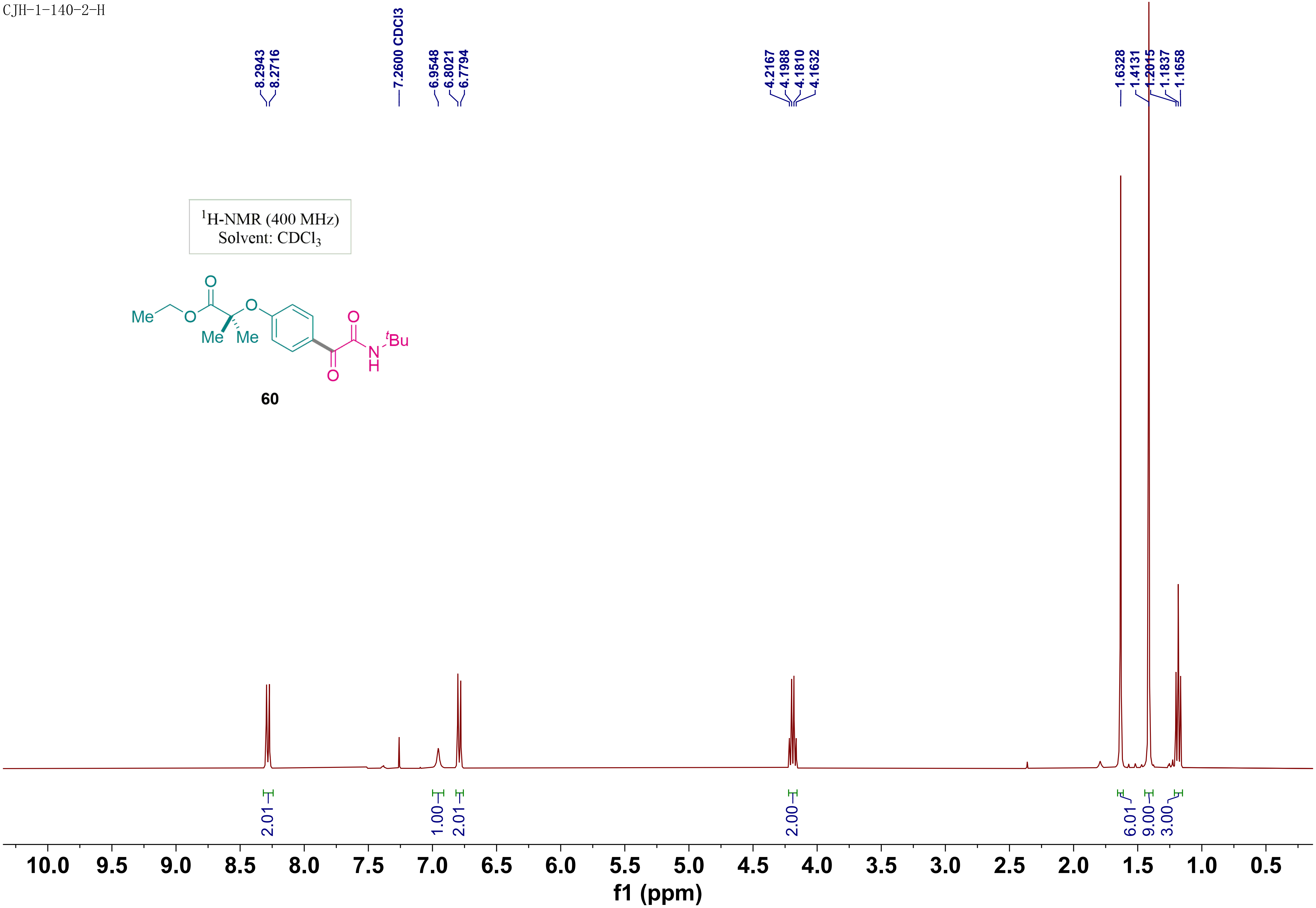

8.2943
8.2716
7.2600 CDCl3
6.9548
6.8021
6.7794
4.2167
4.1988
4.1810
4.1632
1.6328
1.4131
1.2015
1.1837
1.1658
^{1}H-NMR (400 MHz)
Solvent: $CDCl_3$
Me
O
O
O
Me
Me
O
N
H
tBu
O
60
2.01
1.00
2.01
2.00
6.01
9.00
3.00
10.0
9.5
9.0
8.5
8.0
7.5
7.0
6.5
6.0
5.5
5.0
4.5
4.0
3.5
3.0
2.5
2.0
1.5
1.0
0.5
f1 (ppm)

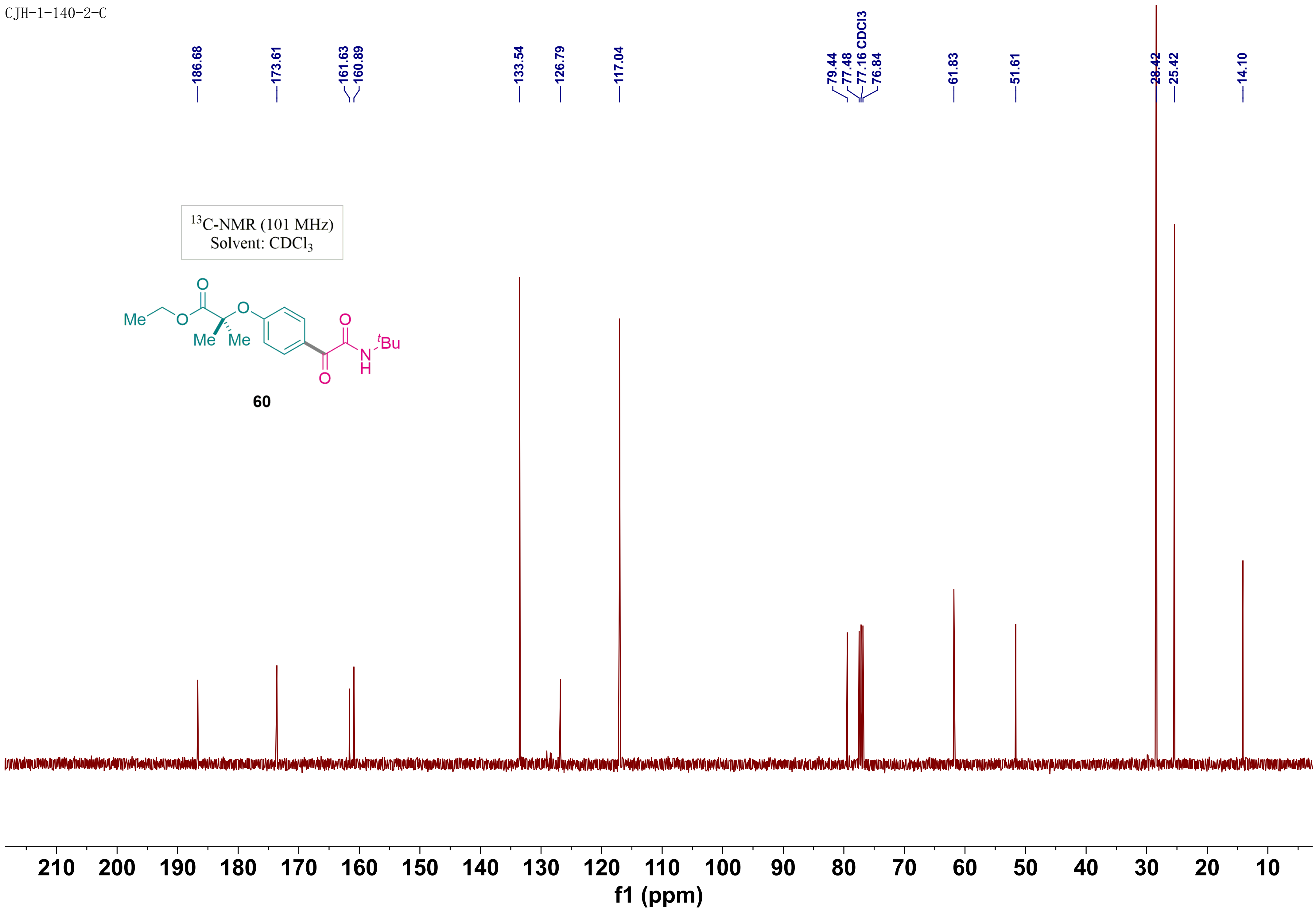
CJH-1-140-2-C
186.68
173.61
161.63
160.89
133.54
126.79
117.04
79.44
77.48
77.16 CDCl3
76.84
61.83
51.61
28.42
25.42
14.10
^{13}C-NMR (101 MHz)
Solvent: $CDCl_3$
60
210 200 190 180 170 160 150 140 130 120 110 100 90 80 70 60 50 40 30 20 10
f1 (ppm)

CJH-1-129-1-H

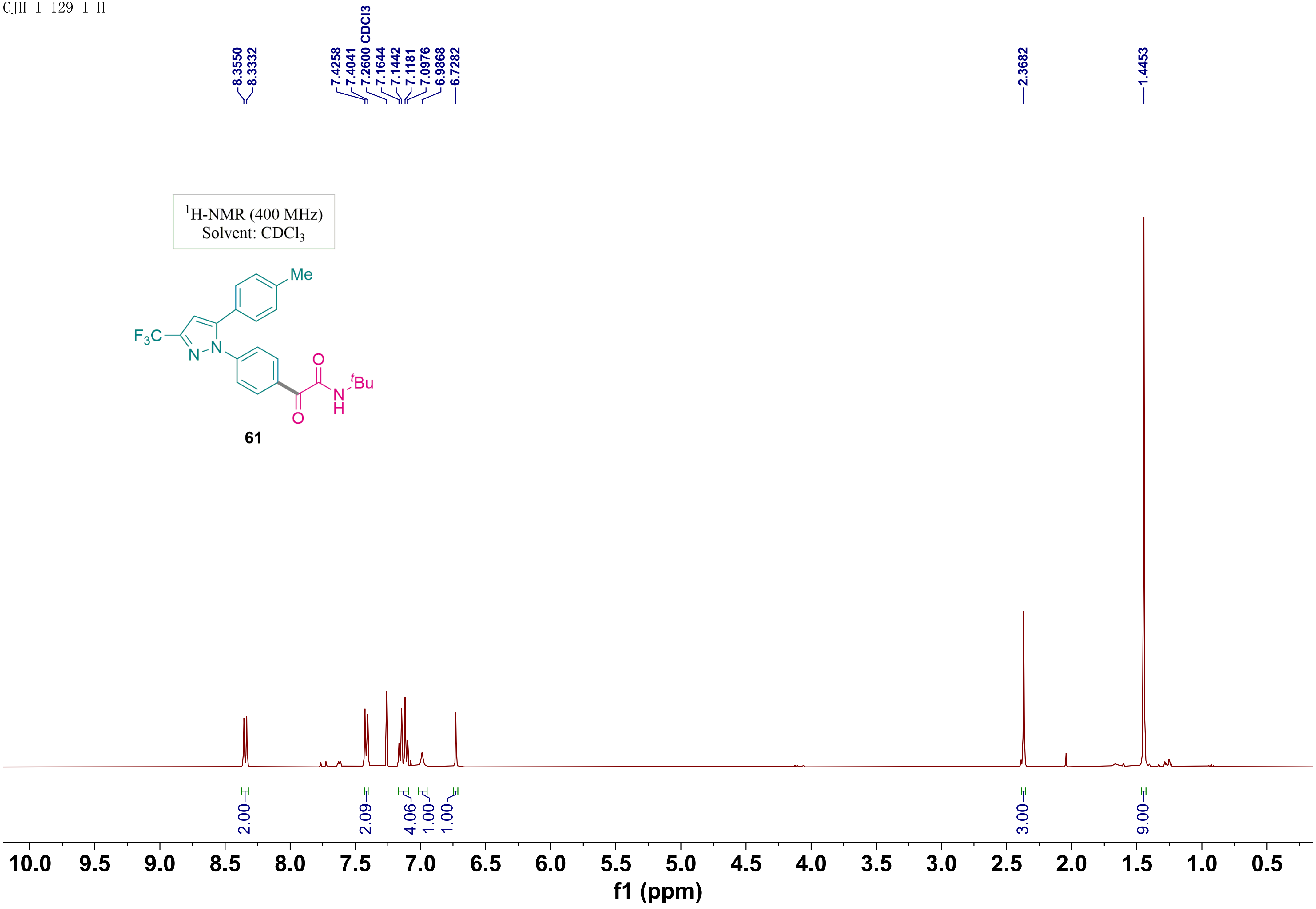

8.3550
8.3332
7.4258
7.4041
7.2600 CDCl3
7.1644
7.1442
7.1181
7.0976
6.9868
6.7282
2.3682
1.4453
¹H-NMR (400 MHz)
Solvent: CDCl₃
Me
F₃C
N
N
O
O
N
H
ᵗBu
61
2.00
2.09
4.06
1.00
1.00
3.00
9.00
10.0
9.5
9.0
8.5
8.0
7.5
7.0
6.5
6.0
5.5
5.0
4.5
4.0
3.5
3.0
2.5
2.0
1.5
1.0
0.5
f1 (ppm)

CJH-1-129-1-C

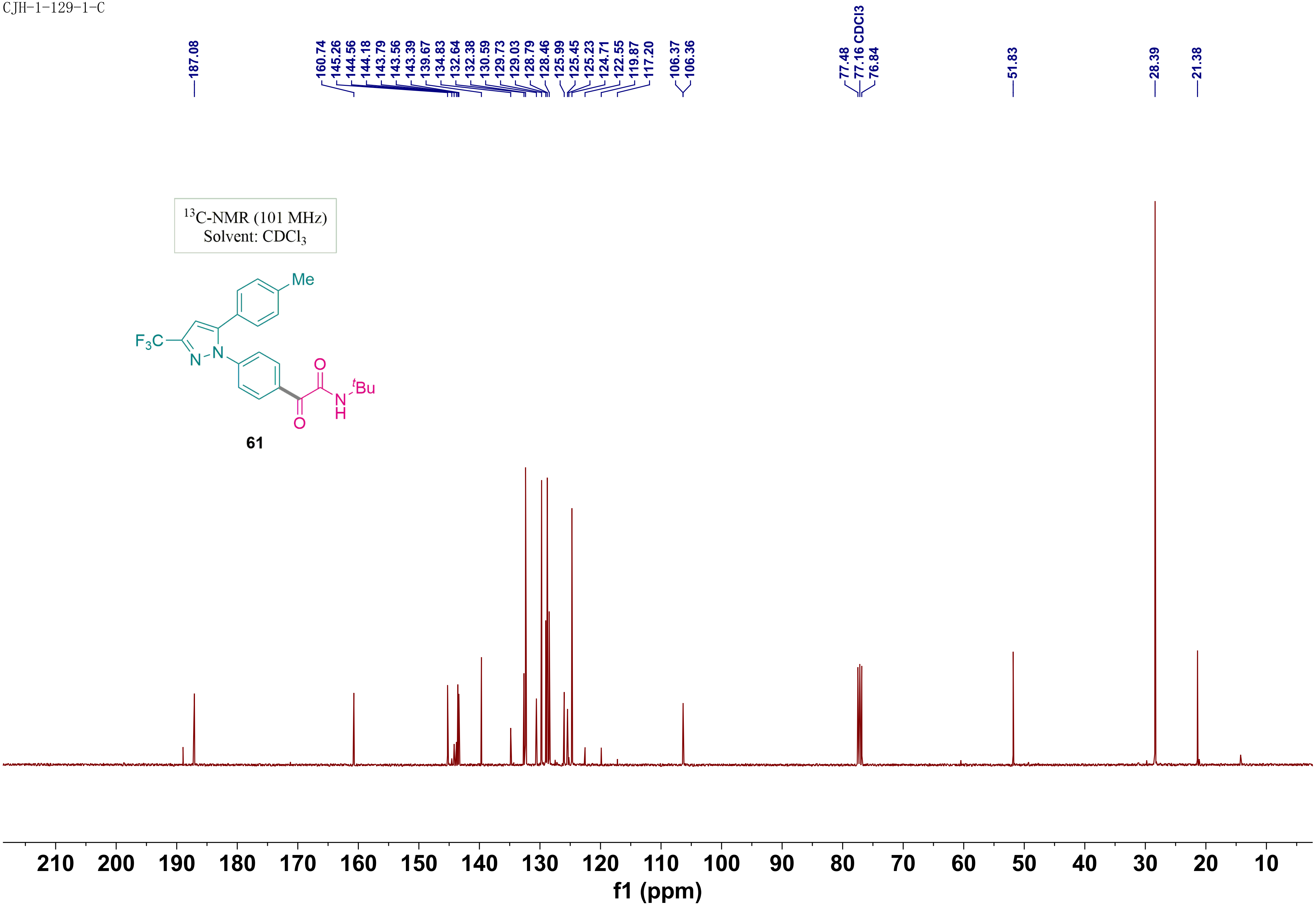
187.08
160.74
145.26
144.56
144.18
143.79
143.56
143.39
139.67
134.83
132.64
132.38
130.59
129.73
129.03
128.79
128.46
125.99
125.45
125.23
124.71
122.55
119.87
117.20
106.37
106.36
77.48
77.16 CDCl3
76.84
51.83
28.39
21.38
13C-NMR (101 MHz)
Solvent: CDCl3
Me
F3C
N
N
O
tBu
N
H
O
61
210 200 190 180 170 160 150 140 130 120 110 100 90 80 70 60 50 40 30 20 10
f1 (ppm)

CJH-1-130-1-H

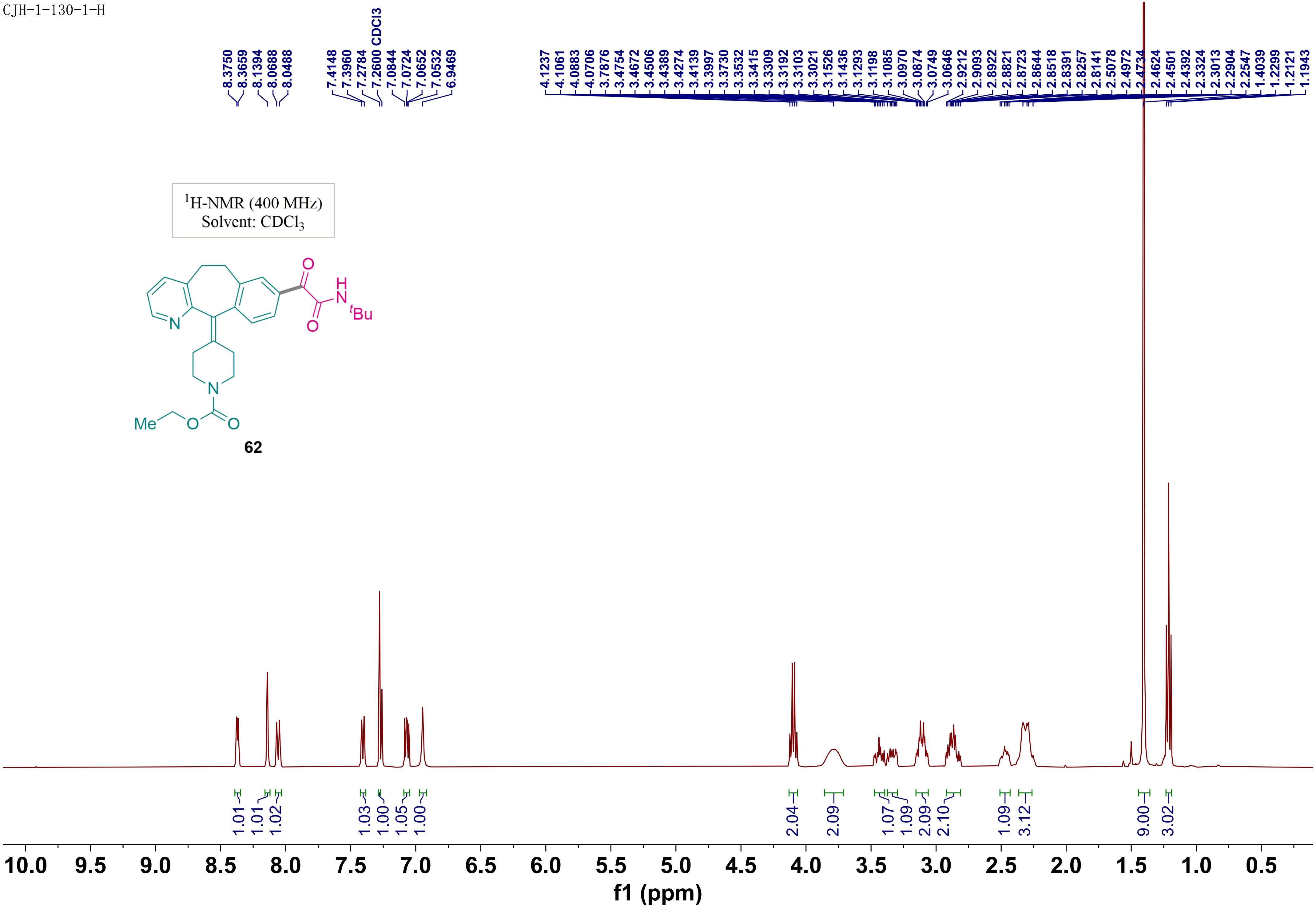

62

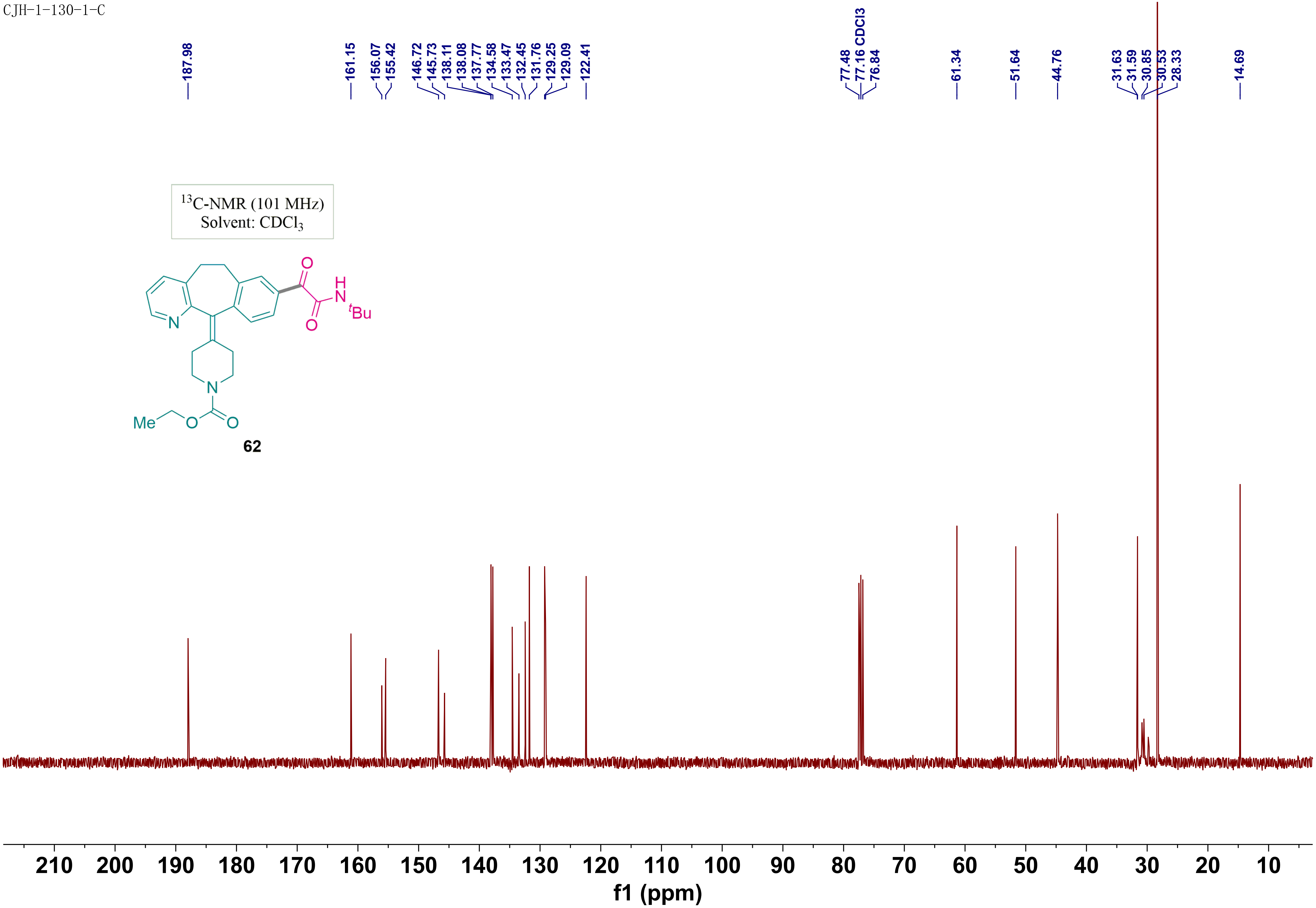
CJH-1-130-1-C
187.98
161.15
156.07
155.42
146.72
145.73
138.11
138.08
137.77
134.58
133.47
132.45
131.76
129.25
129.09
122.41
77.48
77.16 CDCl3
76.84
61.34
51.64
44.76
31.63
31.59
30.85
30.53
28.33
14.69
^{13}C-NMR (101 MHz)
Solvent: $CDCl_3$
O
H
N
^{t}Bu
O
N
N
Me
O
O
62
210 200 190 180 170 160 150 140 130 120 110 100 90 80 70 60 50 40 30 20 10
f1 (ppm)

CJH-1-149-3-H

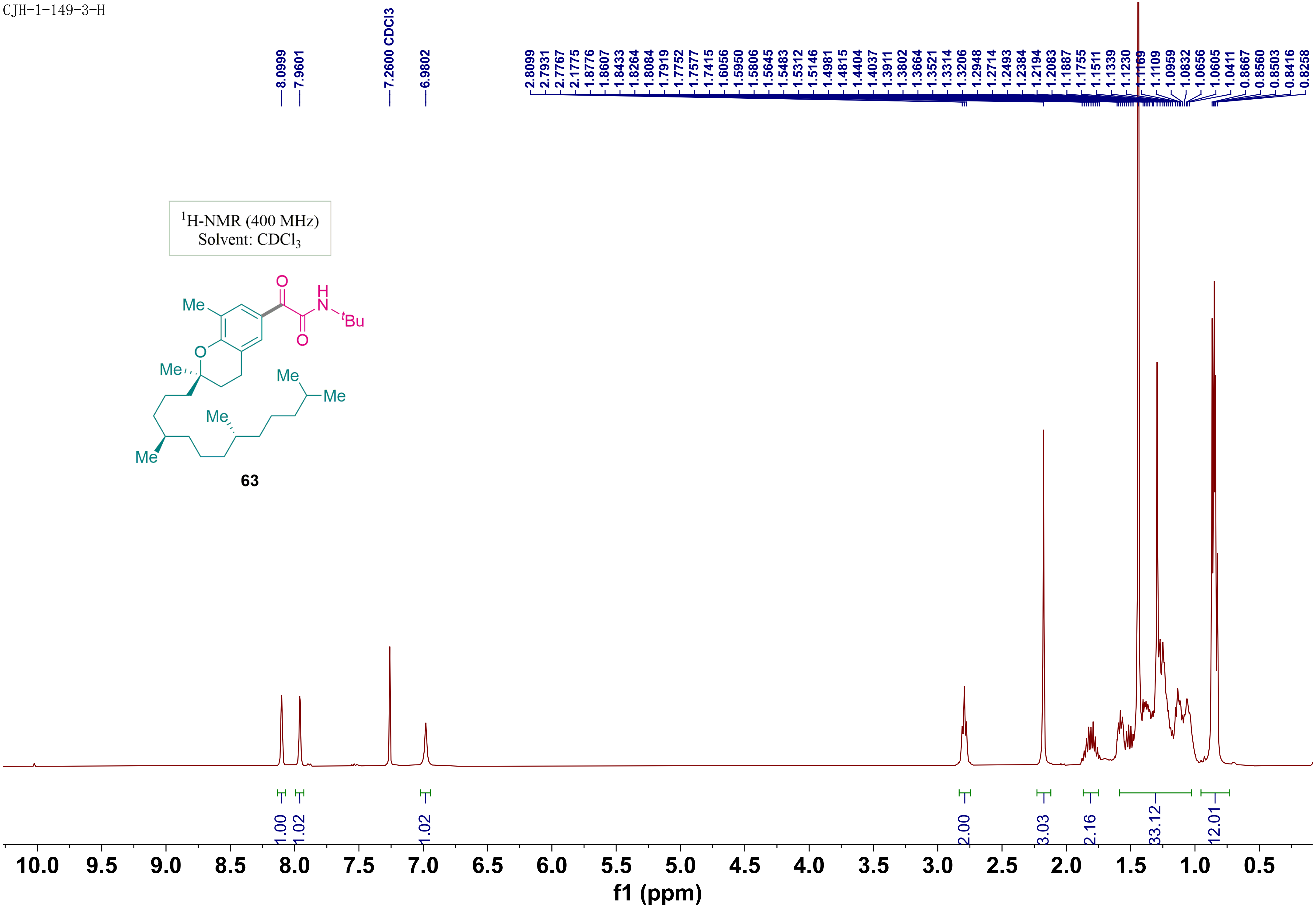

8.0999
7.9601
7.2600 CDCl3
6.9802
2.8099 2.7931 2.7767 2.1775 1.8776 1.8607 1.8433 1.8264 1.8084 1.7919 1.7752 1.7577 1.7415 1.6056 1.5950 1.5806 1.5645 1.5483 1.5312 1.5146 1.4981 1.4815 1.4404 1.4037 1.3911 1.3802 1.3664 1.3521 1.3314 1.3206 1.2948 1.2714 1.2493 1.2384 1.2194 1.2083 1.1887 1.1755 1.1511 1.1339 1.1230 1.1169 1.1109 1.0959 1.0832 1.0656 1.0605 1.0411 0.8667 0.8560 0.8503 0.8416 0.8258
1H-NMR (400 MHz)
Solvent: CDCl3
63
1.00
1.02
1.02
2.00
3.03
2.16
33.12
12.01
10.0 9.5 9.0 8.5 8.0 7.5 7.0 6.5 6.0 5.5 5.0 4.5 4.0 3.5 3.0 2.5 2.0 1.5 1.0 0.5
f1 (ppm)

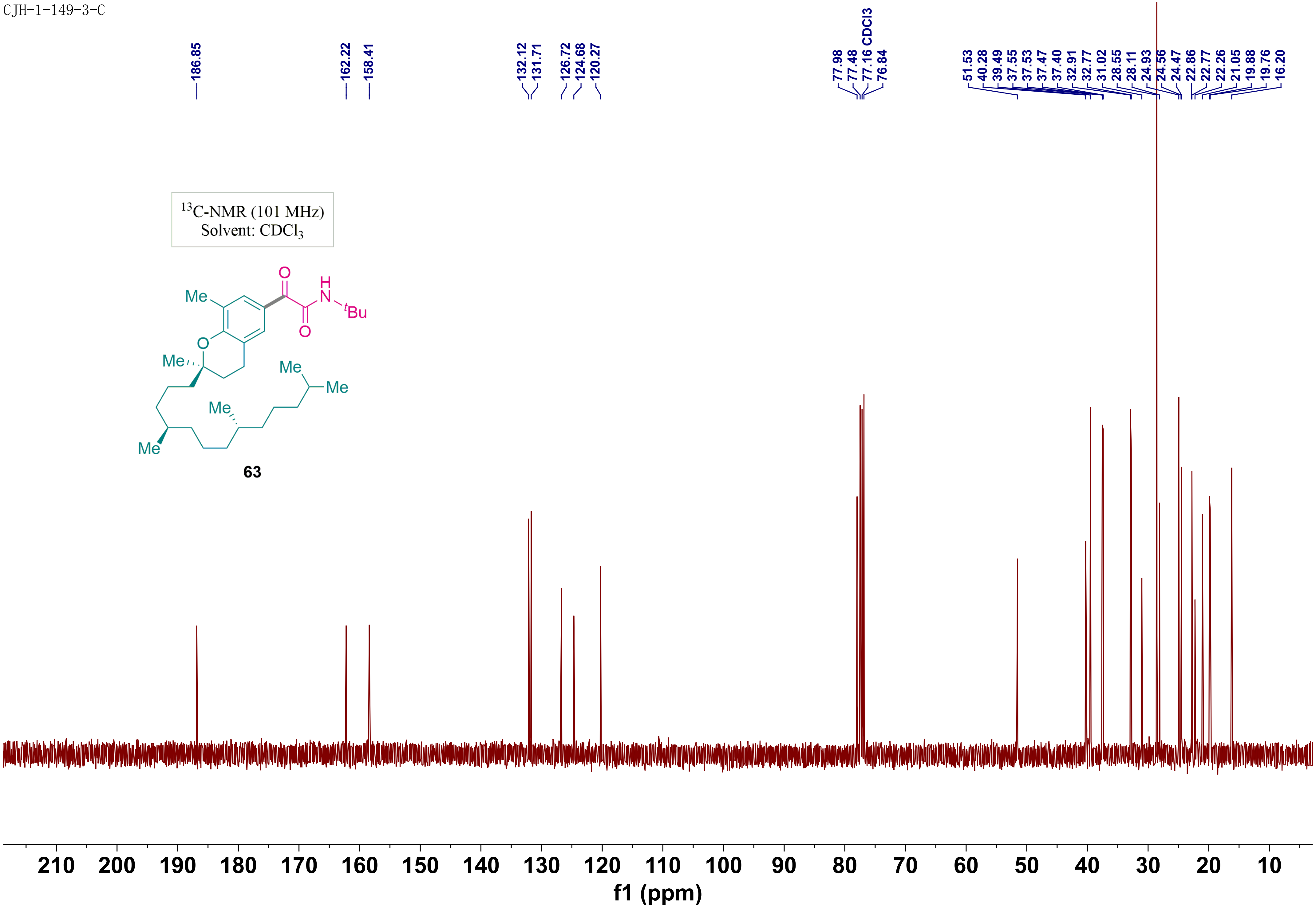
CJH-1-149-3-C
186.85
162.22
158.41
132.12
131.71
126.72
124.68
120.27
77.98
77.48
77.16 CDCl3
76.84
51.53
40.28
39.49
37.55
37.53
37.47
37.40
32.91
32.77
31.02
28.55
28.11
24.93
24.56
24.47
22.86
22.77
22.26
21.05
19.88
19.76
16.20
^{13}C-NMR (101 MHz)
Solvent: $CDCl_3$
Me
O
H
N
tBu
O
O
Me
Me
Me
Me
Me
63
210
200
190
180
170
160
150
140
130
120
110
100
90
80
70
60
50
40
30
20
10
f1 (ppm)

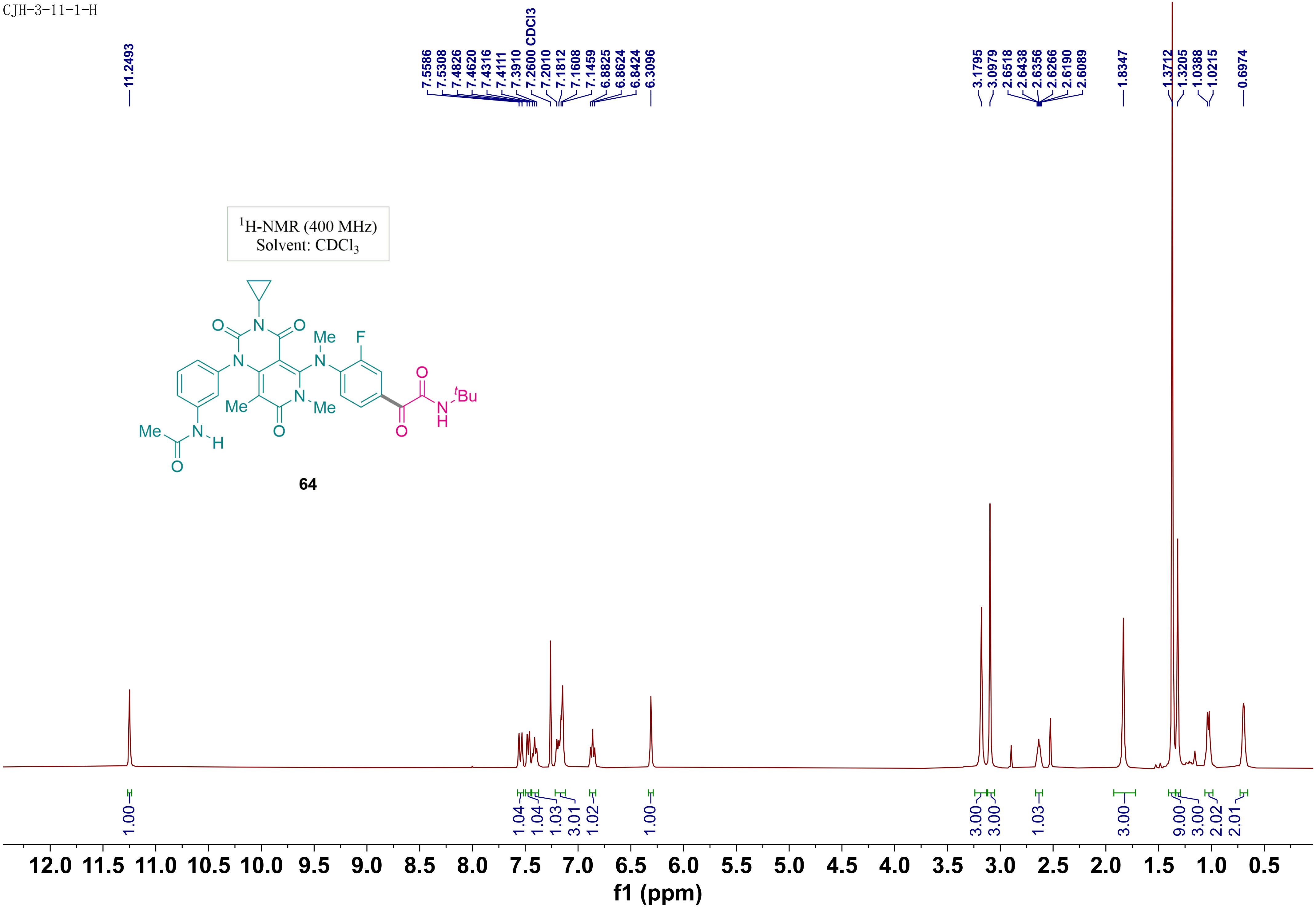
CJH-3-11-1-H
11.2493
7.5586
7.5308
7.4826
7.4620
7.4316
7.4111
7.3910
7.2600 CDCl3
7.2010
7.1812
7.1608
7.1459
6.8825
6.8624
6.8424
6.3096
3.1795
3.0979
2.6518
2.6438
2.6356
2.6266
2.6190
2.6089
1.8347
1.3712
1.3205
1.0388
1.0215
0.6974
1H-NMR (400 MHz)
Solvent: CDCl3
1.00
1.04
1.04
1.03
3.01
1.02
1.00
3.00
3.00
1.03
3.00
9.00
3.00
2.02
2.01
12.0 11.5 11.0 10.5 10.0 9.5 9.0 8.5 8.0 7.5 7.0 6.5 6.0 5.5 5.0 4.5 4.0 3.5 3.0 2.5 2.0 1.5 1.0 0.5
f1 (ppm)

64

CJH-3-11-1-C

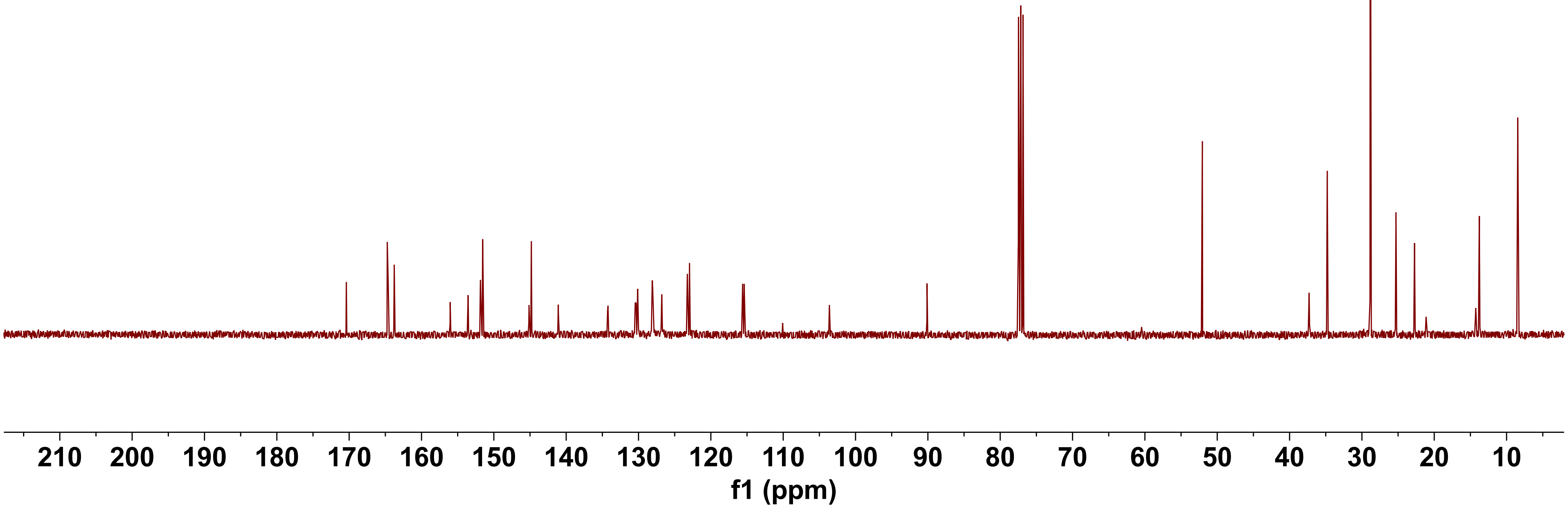

^{13}C-NMR (101 MHz)
Solvent: $CDCl_3$

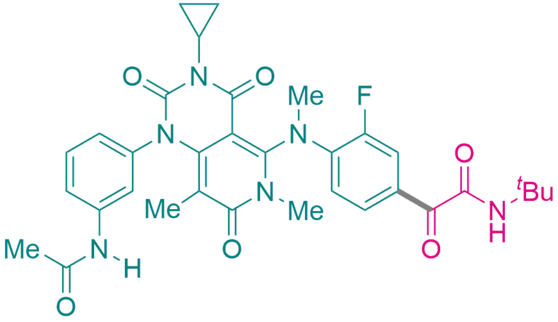

64

CJH-3-58-2-H

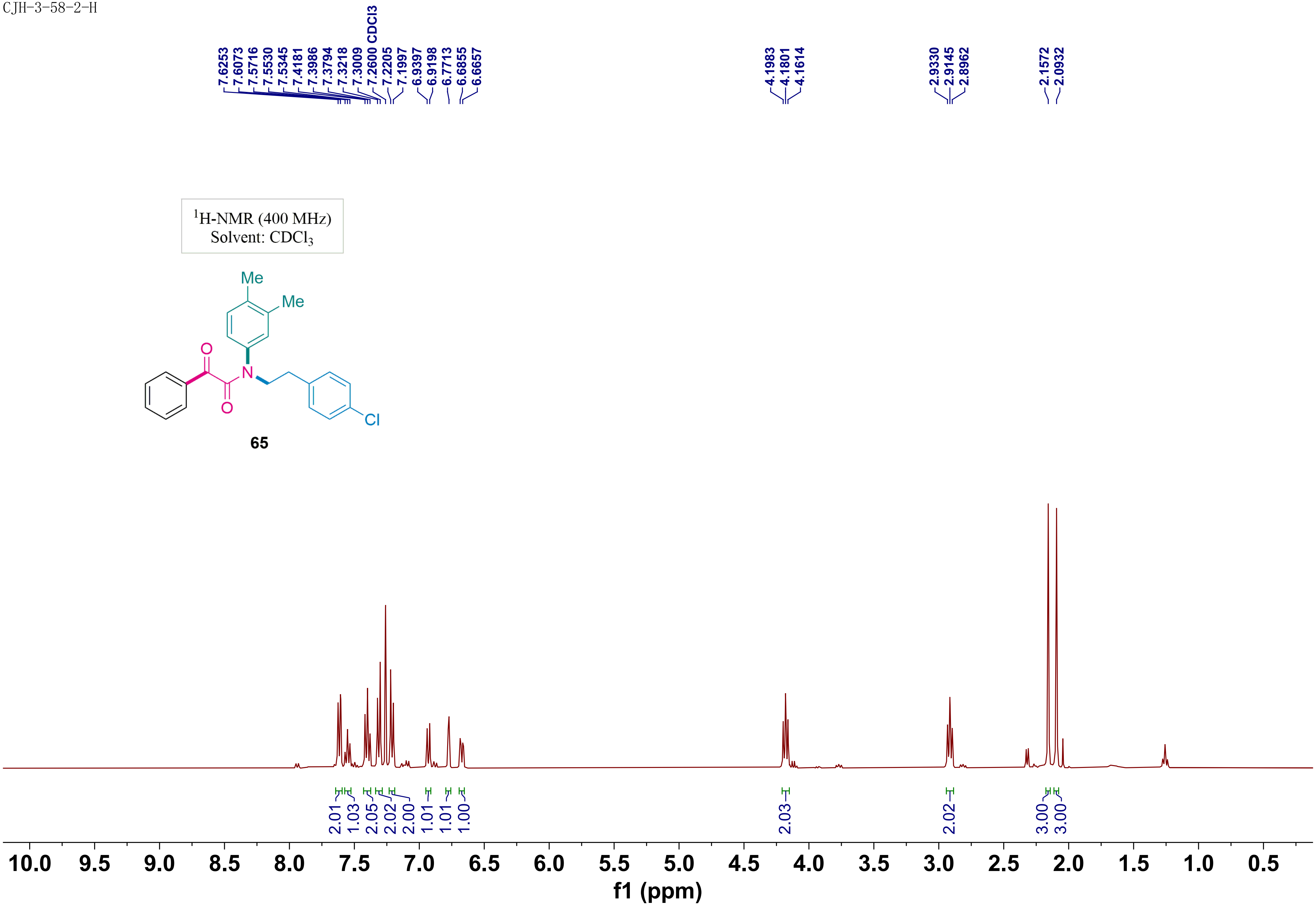

7.6253
7.6073
7.5716
7.5530
7.5345
7.4181
7.3986
7.3794
7.3218
7.3009
7.2600 CDCl3
7.2205
7.1997
6.9397
6.9198
6.7713
6.6855
6.6657
4.1983
4.1801
4.1614
2.9330
2.9145
2.8962
2.1572
2.0932
^{1}H-NMR (400 MHz)
Solvent: $CDCl_3$
Me
Me
O
O
N
Cl
65
2.01
1.03
2.05
2.02
2.00
1.01
1.01
1.00
2.03
2.02
3.00
3.00
10.0 9.5 9.0 8.5 8.0 7.5 7.0 6.5 6.0 5.5 5.0 4.5 4.0 3.5 3.0 2.5 2.0 1.5 1.0 0.5
f1 (ppm)

CJH-3-58-2-C

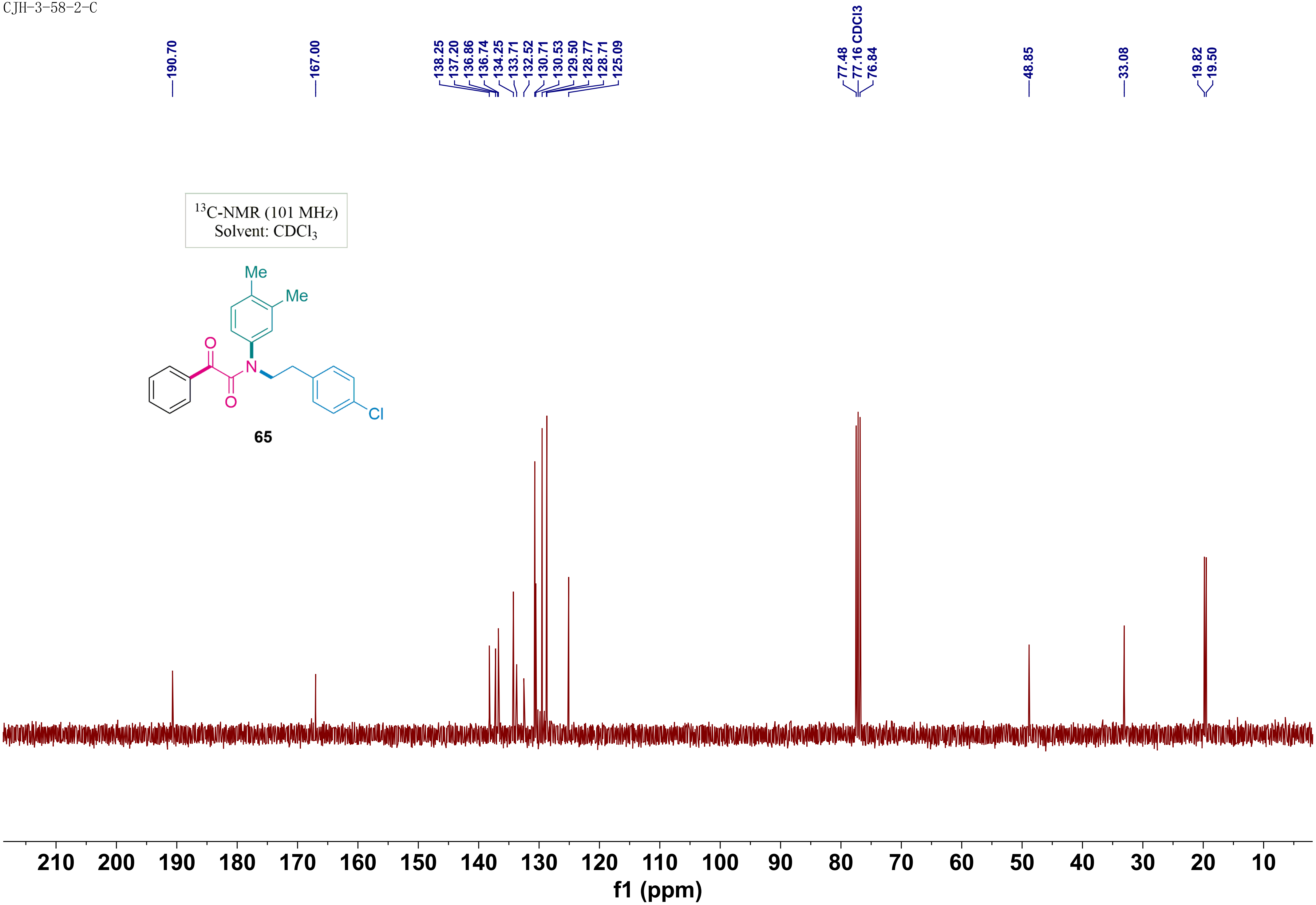
190.70
167.00
138.25
137.20
136.86
136.74
134.25
133.71
132.52
130.71
130.53
129.50
128.77
128.71
125.09
77.48
77.16 CDCl3
76.84
48.85
33.08
19.82
19.50
^{13}C-NMR (101 MHz)
Solvent: $CDCl_3$
Me
Me
O
N
O
Cl
65
210
200
190
180
170
160
150
140
130
120
110
100
90
80
70
60
50
40
30
20
10
f1 (ppm)

CJH-1-188-3-H

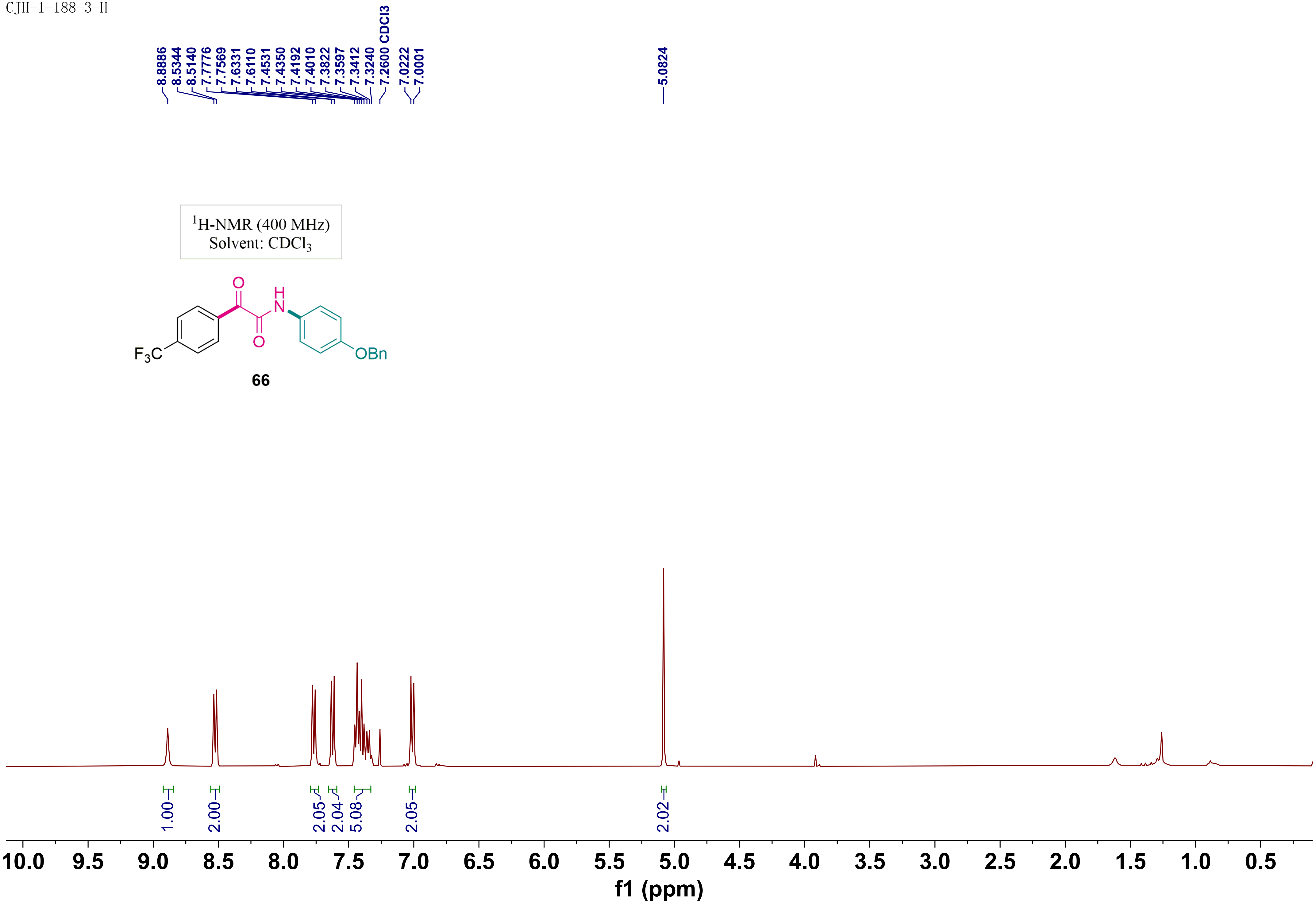
8.8886
8.5344
8.5140
7.7776
7.7569
7.6331
7.6110
7.4531
7.4350
7.4192
7.4010
7.3822
7.3597
7.3412
7.3240
7.2600 CDCl3
7.0222
7.0001
5.0824
^{1}H-NMR (400 MHz)
Solvent: $CDCl_3$
O
H
N
O
F_3C
OBn
66
1.00
2.00
2.05
2.04
5.08
2.05
2.02
10.0
9.5
9.0
8.5
8.0
7.5
7.0
6.5
6.0
5.5
5.0
4.5
4.0
3.5
3.0
2.5
2.0
1.5
1.0
0.5
f1 (ppm)

CJH-1-188-3-C

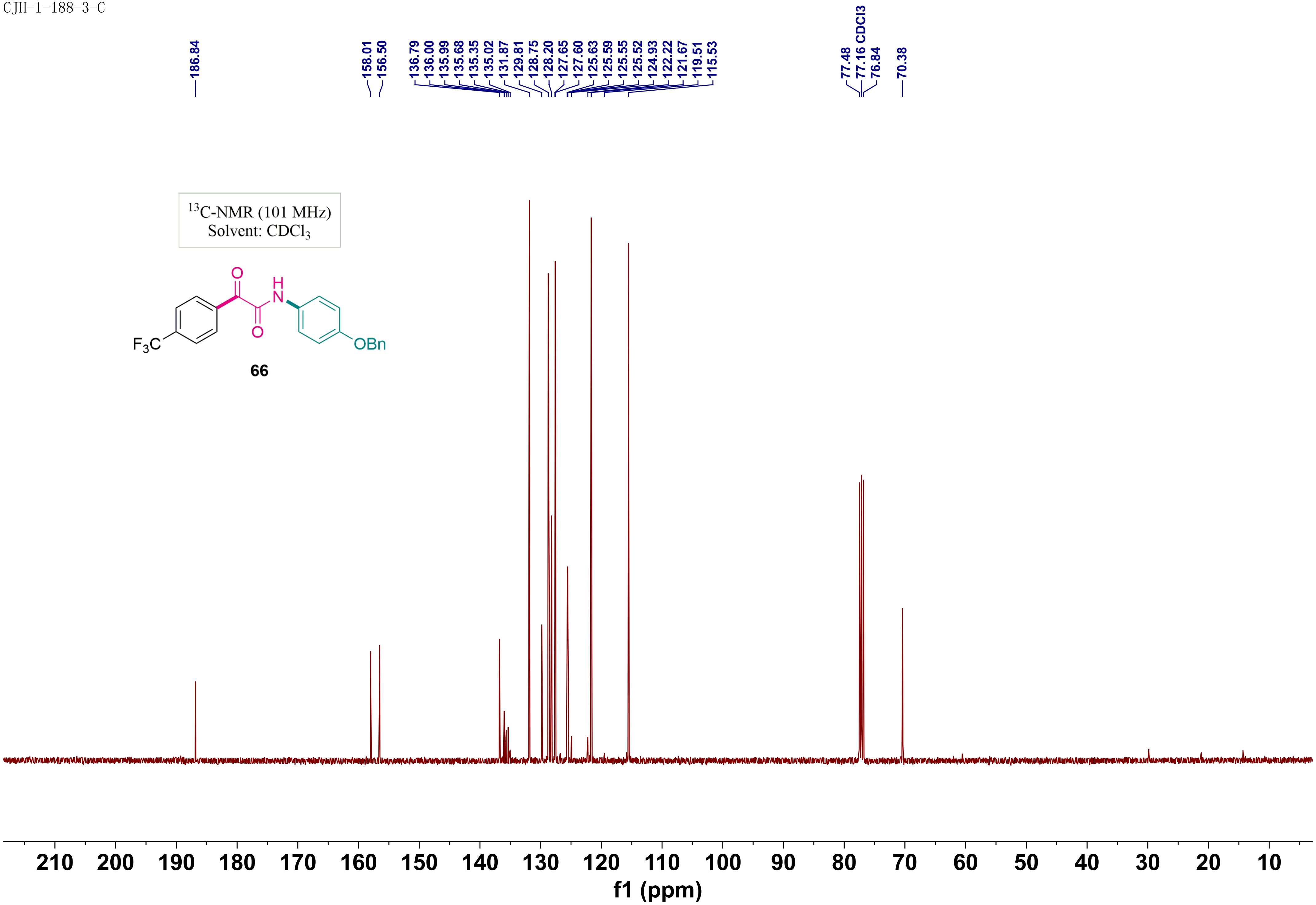
186.84
158.01
156.50
136.79
136.00
135.99
135.68
135.35
135.02
131.87
129.81
128.75
128.20
127.65
127.60
125.63
125.59
125.55
125.52
124.93
122.22
121.67
119.51
115.53
77.48
77.16 CDCl3
76.84
70.38
13C-NMR (101 MHz)
Solvent: CDCl3
O
H
N
O
F3C
OBn
66
210
200
190
180
170
160
150
140
130
120
110
100
90
80
70
60
50
40
30
20
10
f1 (ppm)

CJH-2-52-1-H

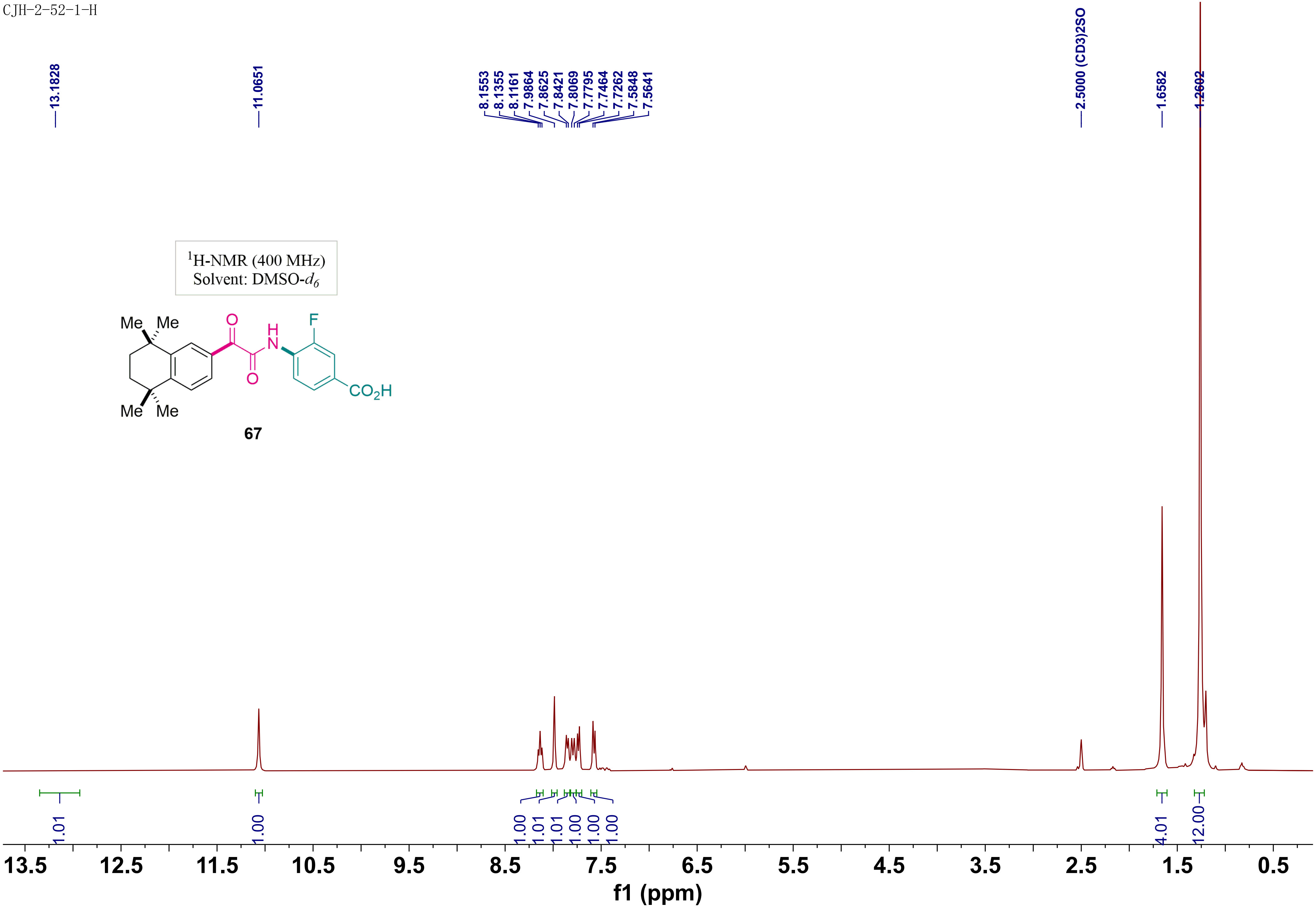

13.1828
11.0651
8.1553
8.1355
8.1161
7.9864
7.8625
7.8421
7.8069
7.7795
7.7464
7.7262
7.5848
7.5641
2.5000 (CD3)2SO
1.6582
1.2602
^{1}H-NMR (400 MHz)
Solvent: DMSO-d_6
Me
Me
Me
Me
O
O
H
N
F
CO_2H
67
1.01
1.00
1.00
1.01
1.01
1.00
1.00
1.00
4.01
12.00
13.5
12.5
11.5
10.5
9.5
8.5
7.5
6.5
5.5
4.5
3.5
2.5
1.5
0.5
f1 (ppm)

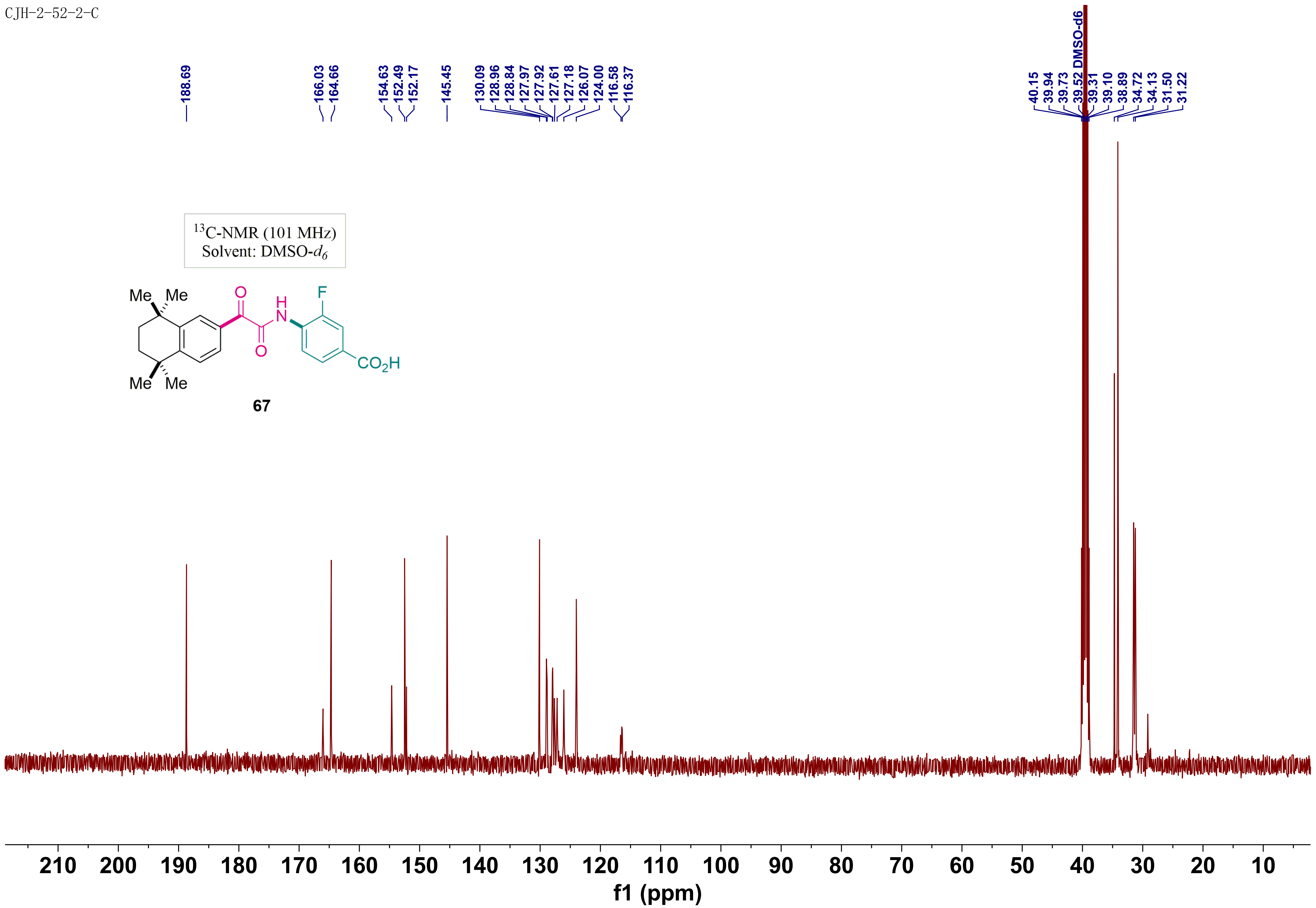
CJH-2-52-2-C
188.69
166.03
164.66
154.63
152.49
152.17
145.45
130.09
128.96
128.84
127.97
127.92
127.61
127.18
126.07
124.00
116.58
116.37
40.15
39.94
39.73
39.52 DMSO-d6
39.31
39.10
38.89
34.72
34.13
31.50
31.22
13C-NMR (101 MHz)
Solvent: DMSO-d6
Me
Me
Me
Me
O
O
H
N
F
CO2H
67
210 200 190 180 170 160 150 140 130 120 110 100 90 80 70 60 50 40 30 20 10
f1 (ppm)

CJH-3-57-1-H

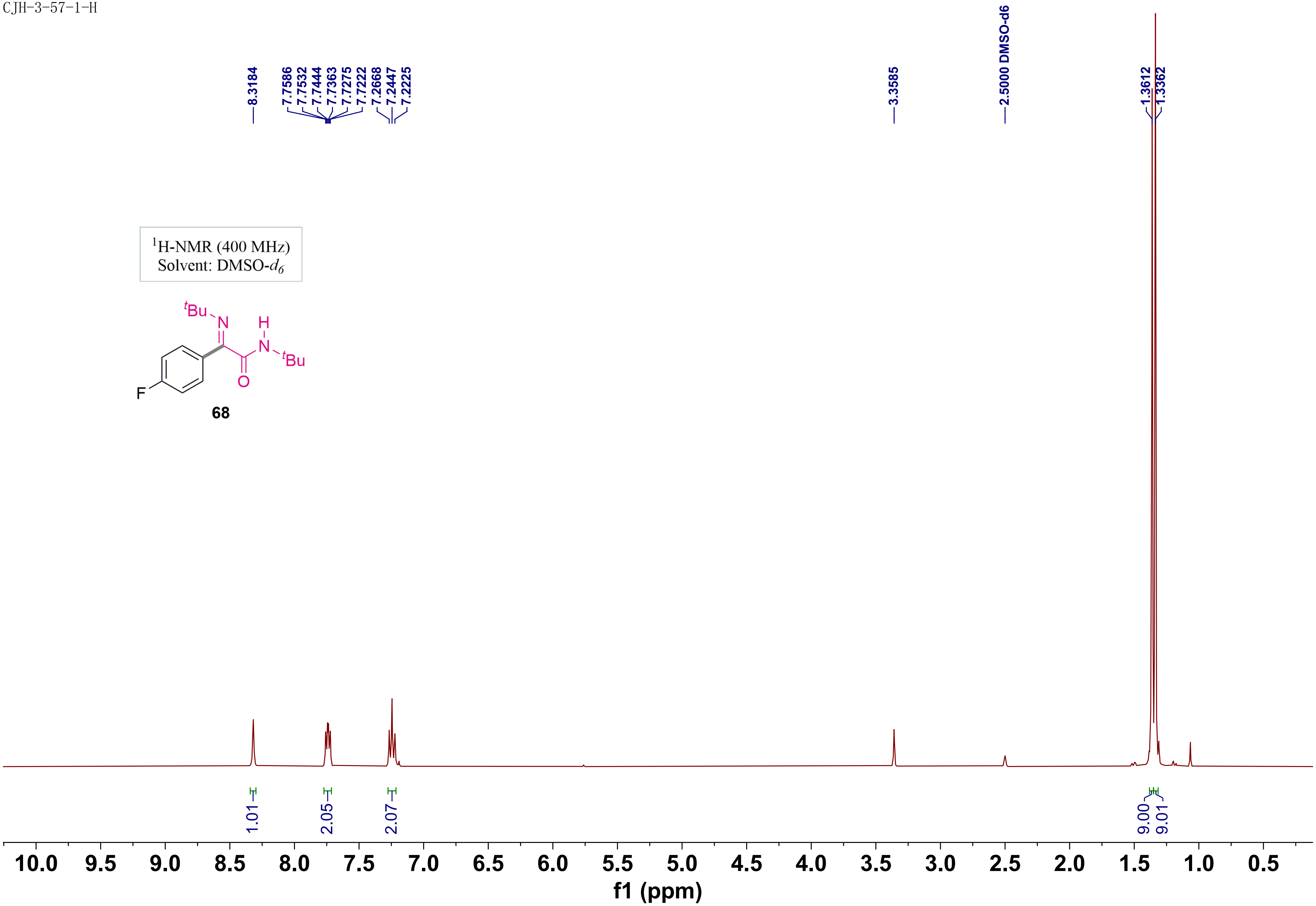

^{1}H-NMR (400 MHz)
Solvent: DMSO-d_6

68

CJH-3-57-1-C

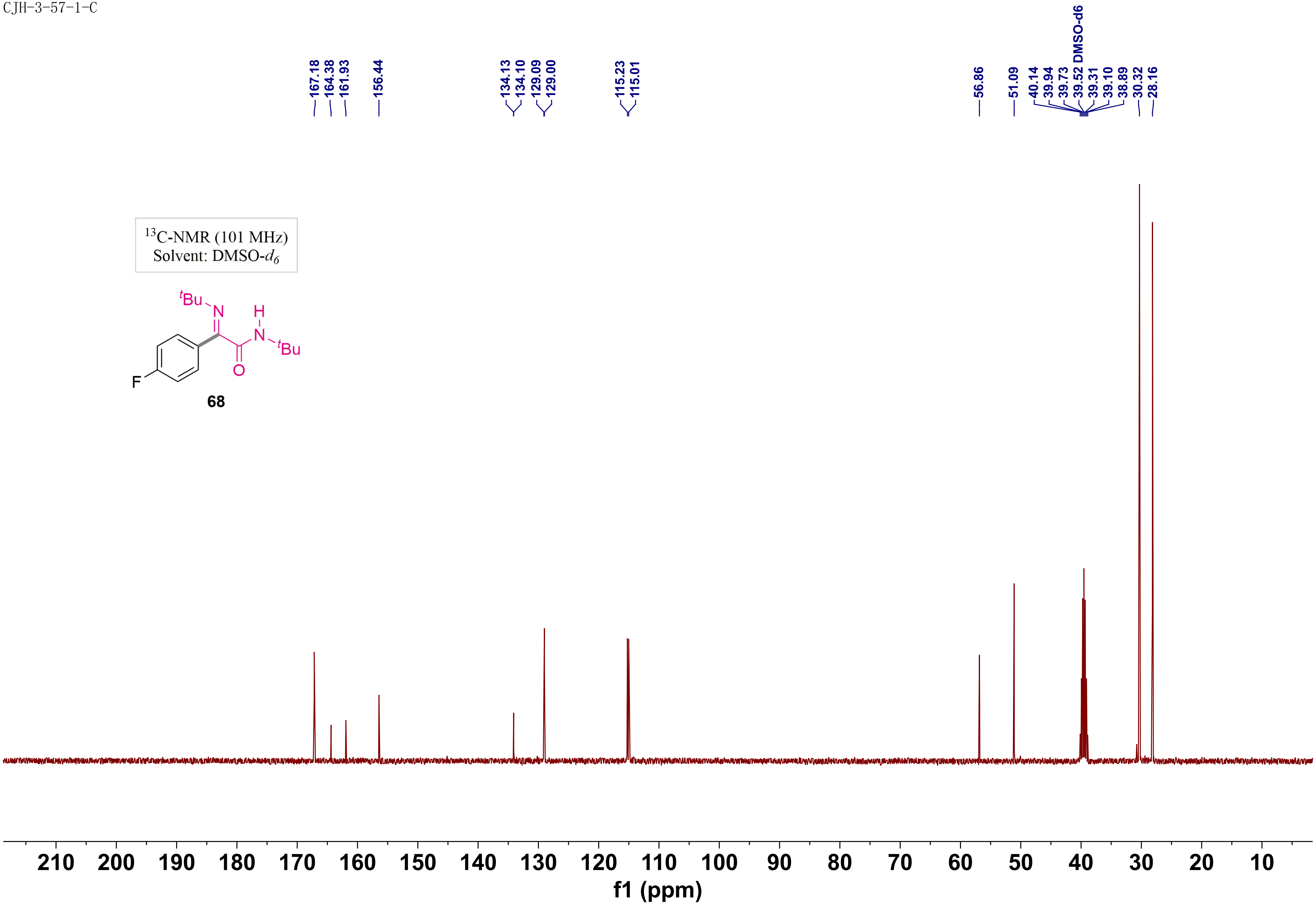

^{13}C-NMR (101 MHz)
Solvent: DMSO-d_6

68

CJH-3-57-1-F

^{19}F-NMR (376 MHz)
Solvent: DMSO-d_6

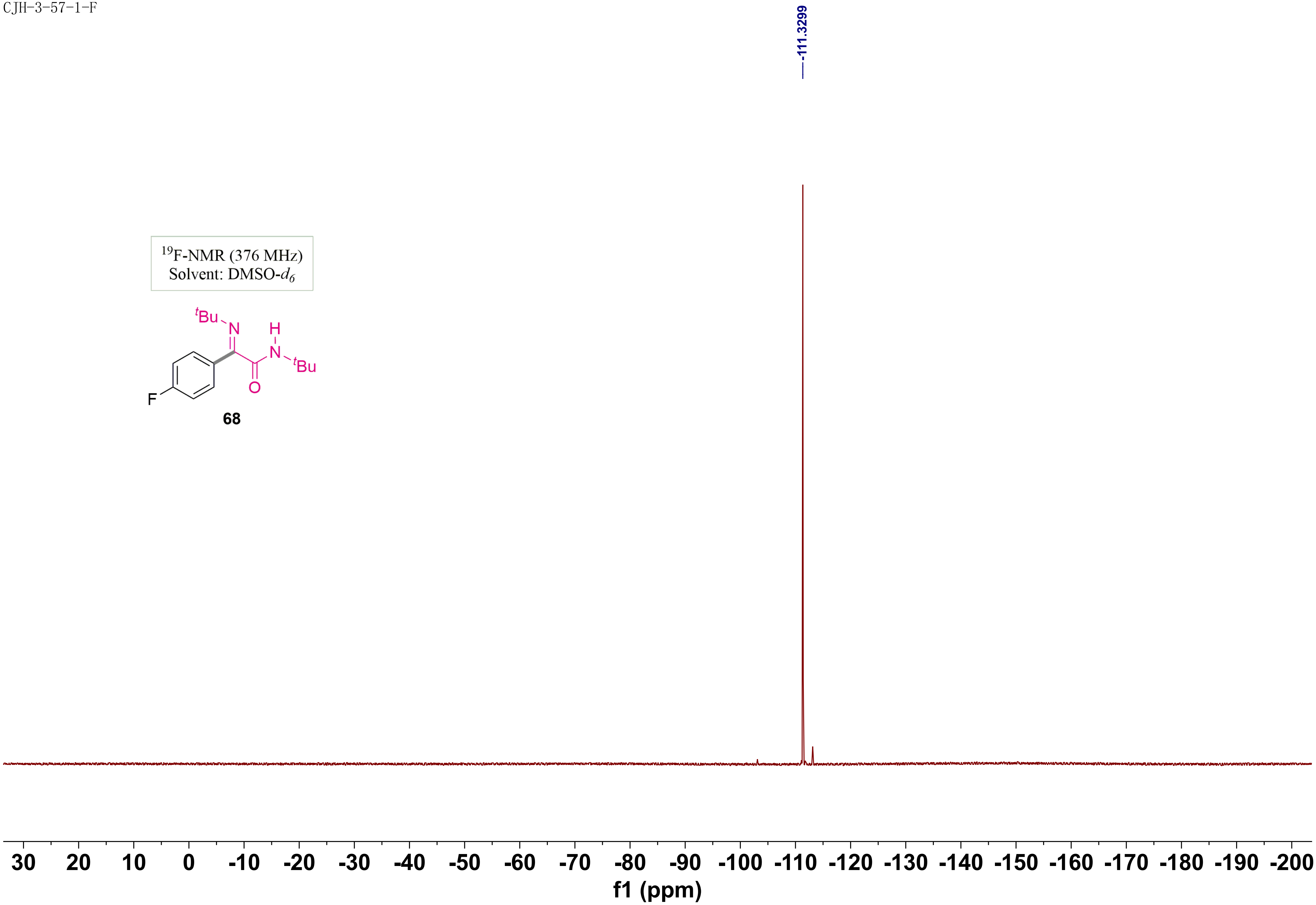

68

CJH-3-130-1-H

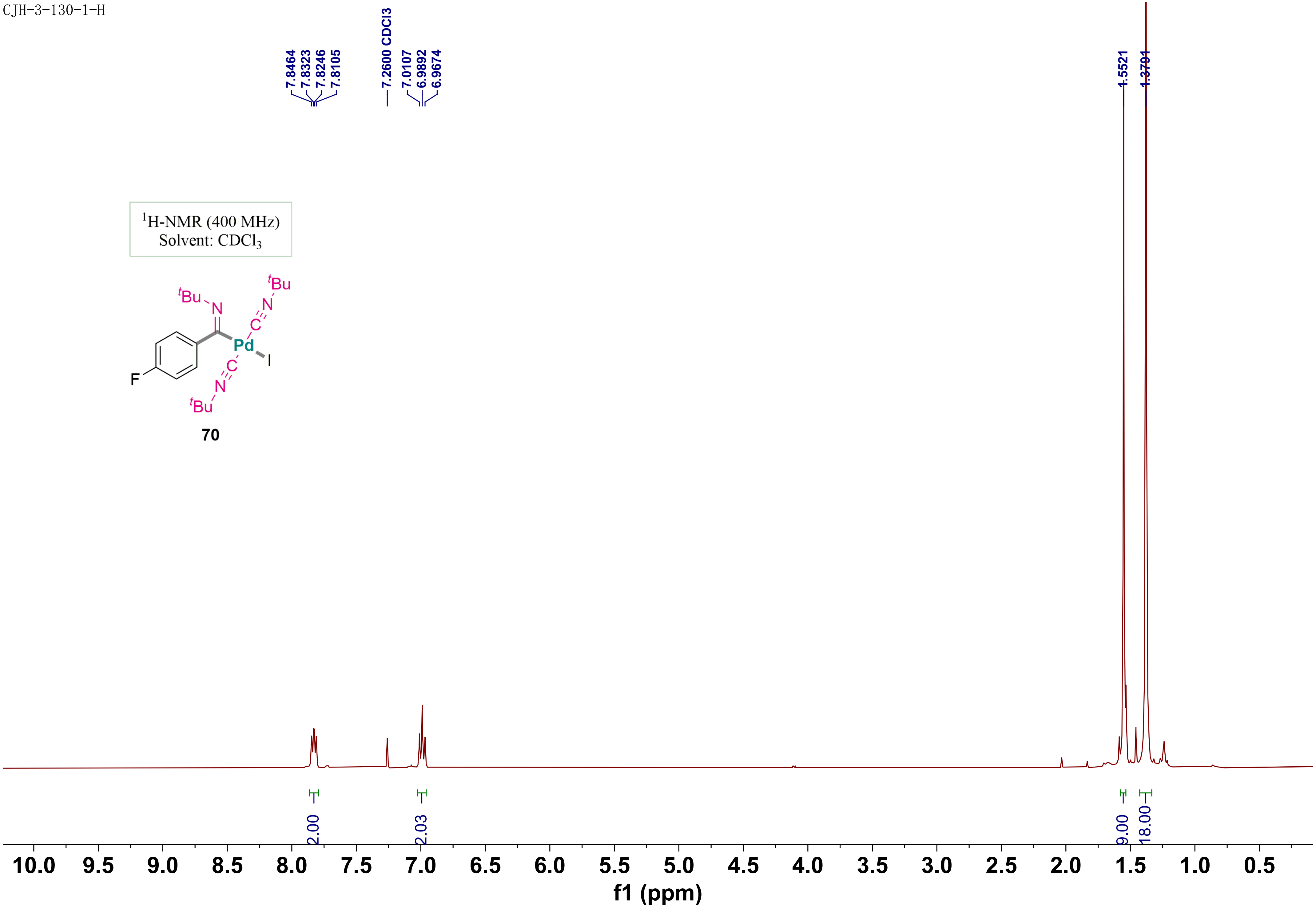
7.8464
7.8323
7.8246
7.8105
7.2600 CDCl3
7.0107
6.9892
6.9674
1.5521
1.3791
1H-NMR (400 MHz)
Solvent: CDCl3
tBu
N
C
Pd
I
F
70
2.00
2.03
9.00
18.00
10.0
9.5
9.0
8.5
8.0
7.5
7.0
6.5
6.0
5.5
5.0
4.5
4.0
3.5
3.0
2.5
2.0
1.5
1.0
0.5
f1 (ppm)

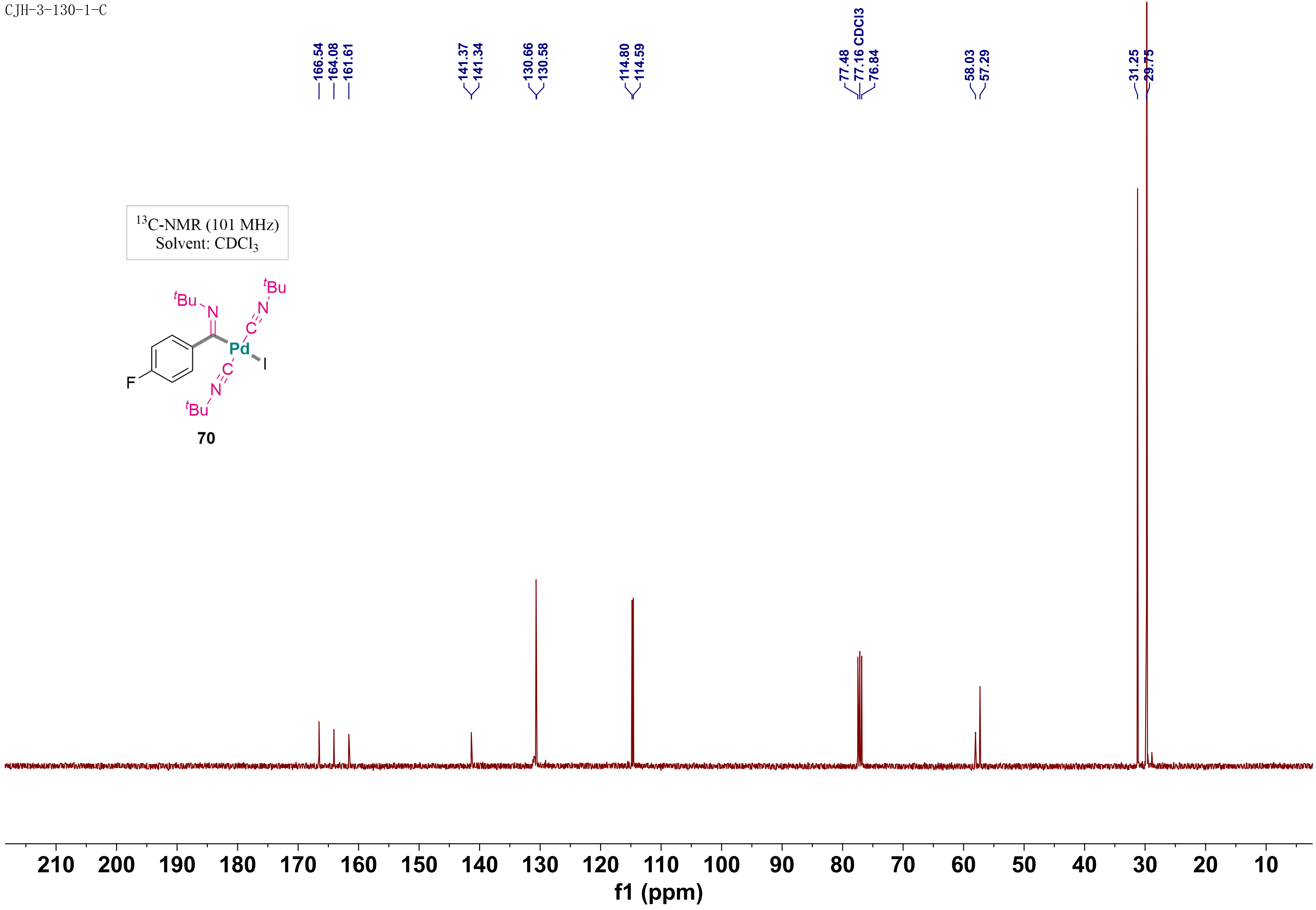
CJH-3-130-1-C
166.54
164.08
161.61
141.37
141.34
130.66
130.58
114.80
114.59
77.48
77.16 CDCl3
76.84
58.03
57.29
31.25
29.75
^{13}C-NMR (101 MHz)
Solvent: $CDCl_3$
^{t}Bu
N
^{t}Bu
N
C
Pd
I
F
C
N
^{t}Bu
70
210 200 190 180 170 160 150 140 130 120 110 100 90 80 70 60 50 40 30 20 10
f1 (ppm)

CJH-3-130-1-F

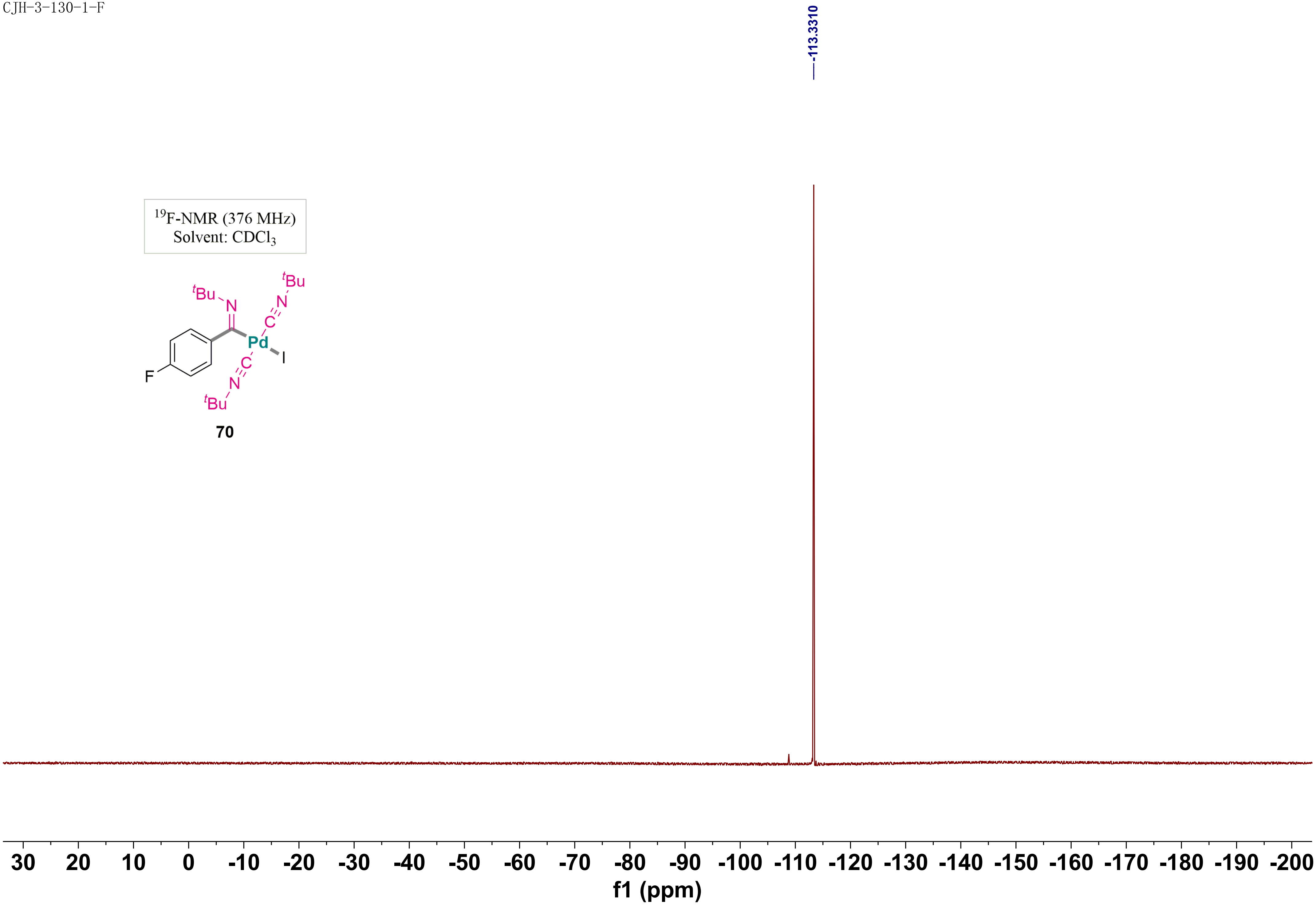

^{19}F-NMR (376 MHz)
Solvent: $CDCl_3$

70
